# The GATA transcription factor BcWCL2 regulates citric acid secretion to maintain redox homeostasis and full virulence in *Botrytis cinerea*

**DOI:** 10.1128/mbio.00133-24

**Published:** 2024-05-30

**Authors:** Weiheng Ren, Chen Qian, Dandan Ren, Yunfei Cai, Zhaohui Deng, Ning Zhang, Congcong Wang, Yiwen Wang, Pinkuan Zhu, Ling Xu

**Affiliations:** 1 School of Life Sciences, East China Normal University, Shanghai, China; Max Planck Institute for Terrestrial Microbiology, Marburg, Germany

**Keywords:** BcWCL2, citric acid, redox homeostasis, virulence, *Botrytis cinerea*

## Abstract

*Botrytis cinerea* is a typical necrotrophic plant pathogenic fungus which can deliberately acidify host tissues and trigger oxidative bursts therein to facilitate its virulence. The white collar complex (WCC), consisting of BcWCL1 and BcWCL2, is recognized as the primary light receptor in *B. cinerea*. Nevertheless, the specific mechanisms through which the WCC components, particularly BcWCL2 as a GATA transcription factor, control virulence are not yet fully understood. This study demonstrates that deletion of BcWCL2 results in the loss of light-sensitive phenotypic characteristics. Additionally, the Δ*bcwcl2* strain exhibits reduced secretion of citrate, delayed infection cushion development, weaker hyphal penetration, and decreased virulence. The application of exogenous citric acid was found to restore infection cushion formation, hyphal penetration, and virulence of the Δ*bcwcl2* strain. Transcriptome analysis at 48 h post-inoculation revealed that two citrate synthases, putative citrate transporters, hydrolytic enzymes, and reactive oxygen species scavenging-related genes were down-regulated in Δ*bcwcl2*, whereas exogenous citric acid application restored the expression of the above genes involved in the early infection process of Δ*bcwcl2*. Moreover, the expression of *Bcvel1*, a known regulator of citrate secretion, tissue acidification, and secondary metabolism, was down-regulated in Δ*bcwcl2* but not in Δ*bcwcl1*. ChIP-qPCR and electrophoretic mobility shift assays revealed that BcWCL2 can bind to the promoter sequences of *Bcvel1*. Overexpressing *Bcvel1* in Δ*bcwcl2* was found to rescue the mutant defects. Collectively, our findings indicate that BcWCL2 regulates the expression of the global regulator *Bcvel1* to influence citrate secretion, tissue acidification, redox homeostasis, and virulence of *B. cinerea*.

**IMPORTANCE:**

This study illustrated the significance of the fungal blue light receptor component BcWCL2 protein in regulating citrate secretion in *Botrytis cinerea*. Unlike BcWCL1, BcWCL2 may contribute to redox homeostasis maintenance during infection cushion formation, ultimately proving to be essential for full virulence. It is also demonstrated that BcWCL2 can regulate the expression of *Bcvel1* to influence host tissue acidification, citrate secretion, infection cushion development, and virulence. While the role of organic acids secreted by plant pathogenic fungi in fungus-host interactions has been recognized, this paper revealed the importance, regulatory mechanisms, and key transcription factors that control organic acid secretion. These understanding of the pathogenetic mechanism of plant pathogens can provide valuable insights for developing effective prevention and treatment strategies against fungal diseases.

## INTRODUCTION

*Botrytis cinerea* is a ubiquitous plant pathogenic fungus that infects over 1,400 plant species. It is the primary cause of post-harvest losses of fruits and vegetables, resulting in significant yield reductions in global agricultural production (1–3). The conidia of *B. cinerea* attach to and germinate on plant surfaces, subsequently differentiate into germ tubes or elongated hyphae, which then form simple appressoria or multicellular infection cushions, which invade the host tissue by releasing diverse virulence factors (4). As a necrotrophic fungus, *B. cinerea* promotes virulence by inducing oxidative bursts and hypersensitive cell death in its host plants (5, 6). Additionally, the fungus secretes a range of phytotoxic proteins, metabolites, and small RNAs that contribute to enhancing host invasion (7–9). Moreover, *B. cinerea* is capable of secreting organic acids to acidify plant tissues, thereby potentially increasing its lytic enzymes’ activity and enhancing host infection (10). Some studies have indicated that oxalic acid is secreted by *B. cinerea* and *Sclerotinia sclerotiorum* to support its virulence (11–13), whereas others have reported that *B. cinerea* mainly produces citrate (and succinate), which might be more important for host infestation (14). Müller et al. have reported that the VELVET complex, a global regulatory factor, may influence the activity of acid hydrolases by controlling the secretion of citric acid, thereby enhancing the virulence of *B. cinerea* (15).

Environmental factors are known to significantly affect the growth and virulence of fungal pathogens (16–19). Light, for example, is a major environmental signal influencing different life processes of fungi, including spore production, phototropism, sexual and asexual reproduction, secondary metabolism, circadian rhythm, and virulence (20). However, excessive exposure to light can lead to photooxidative stress and damage in fungal cells (21, 22). Thus, it can be expected that fungi might utilize information on the different facets of light, such as its intensity and quality, and light-induced reactive oxygen species (ROS) to monitor their environment and make key decisions regarding growth, development, and host infection. This assumption is supported by the observation that *B. cinerea* encodes 11 potential photoreceptors for sensing near-UV, blue, green, and the ratio between red and far-red light (23). Fungi are known to perceive and respond to light via complex light-sensing systems (20, 24), among which is the widely conserved white collar complex (WCC) blue light receptor (25), which contributes to the regulation of growth, development, and secondary metabolism (26). In *Neurospora crassa*, the WCC comprises WC-1 and WC-2, both of which are GATA-type transcription factors (27, 28). WC-1 contains a light-sensitive light oxygen voltage (LOV) domain that binds to the flavin chromophore and forms a heterodimeric complex with WC-2 through their common Per ARNT Sim (PAS) domains (28, 29). In response to light, the WCC complex is activated to regulate the expression of light-responsive genes (30, 31). The light-dependent regulatory role of WCC has been found to be conserved among Ascomycota, Mucoromycota, and Basidiomycota (32–37). However, the WCC components show functional differentiation among different classes of fungal pathogens. In *Magnaporthe oryzae*, MgWC-1 has been found to negatively regulate virulence in a light-dependent manner (38), whereas in *Fusarium graminearum*, the virulence of the FgWC-1 deletion mutant did not differ from that of the wild type (WT) (39). In contrast, the deletion of FaWC-1 in *F. asiaticum* resulted in a reduction in virulence (40). Currently, research on the role of the blue light receptor WCC in fungal pathogens is focusing mainly on WC-1. However, since certain clues indicated that WC-1 and WC-2, in addition to forming a WCC for light sensing, could possess independent functions, additional efforts are deserved for the investigation of the transcriptional regulation functions of WC-2 (41–43).

As in other fungi, light sensing of *B. cinerea* is primarily mediated by the BcWCL1 and BcWCL2 protein complex. The interaction between BcWCL1 and BcWCL2 proteins occurs through their PAS domains, independent of light conditions (44), and BcWCL1 and BcWCL2 could both be located in the nuclei (45). The loss of BcWCL1 was found to be associated with increased sporulation and reduced secretion of oxalic acid, resulting in a decreased ability to acidify culture mediums during light-dark (LD) conditions. This, in turn, affected virulence under light conditions and the regulation of light-responsive genes, such as *Bcltf1*, *Bcltf2*, and *Bcltf3* (46). Among these genes, BcLTF1 transcription factor plays a crucial role in maintaining ROS homeostasis, regulating secondary metabolism, and influencing virulence (47). However, only some of the light-inducing genes in *B. cinerea* were found to rely on the presence of BcWCL1 (48), leaving open the question whether and how BcWCL2 targets unique downstream genes that are associated with virulence characteristics of this necrotrophic pathogen.

In this study, we found that the absence of BcWCL2 in *B. cinerea* led to reductions in citrate secretion, acidification capacity, tolerance to oxidative stress, and infection cushion formation, whereas the provision of citric acid from an external source could restore these mutant defects. Furthermore, the Δ*bcwcl2* strain showed down-regulation of genes responsible for citrate synthesis, citrate transport, and acid hydrolysis. We also established that direct regulation of *Bcvel1* transcription by BcWCL2 influences the secretion of citrate, which acts as an antioxidant agent and is essential for maintaining cellular oxidative homeostasis and full virulence. Collectively, our findings reveal that BcWCL2 contributes to the regulation of citrate secretion, redox homeostasis, and virulence of *B. cinerea*.

## RESULTS

### The effects of BcWCL2 on virulence and oxidative stress tolerance are different from BcWCL1

Fungal GATA transcription factors can be divided based on the number of ZnF GATA domains and zinc finger motifs. *B. cinerea* encodes seven GATA transcription factors, namely, BcWCL1, BcWCL2, BcSNF5, BcLTF1, BcAREA, BcAREB, and BIRl (Fig. S1). As the WC-1 and WC-2 proteins may independently retain their unique downstream genes besides forming WCC (26, 49), this study revisited the functions of BcWCL1 and BcWCL2 with specific focus on phenotypic differences due to their mutations. Gene expression analysis with WT and the single mutants Δ*bcwcl1* and Δ*bcwcl2* revealed that there was no significant difference neither in *Bcwcl2* expression levels between the WT and Δ*bcwcl1* strains nor in *Bcwcl1* expression levels between the WT and Δ*bcwcl2* strains (Fig. S3E and F), suggesting that the transcription of *Bcwcl1* and *Bcwcl2* is not likely interdependent on the presence of each other.

**Fig 1:**
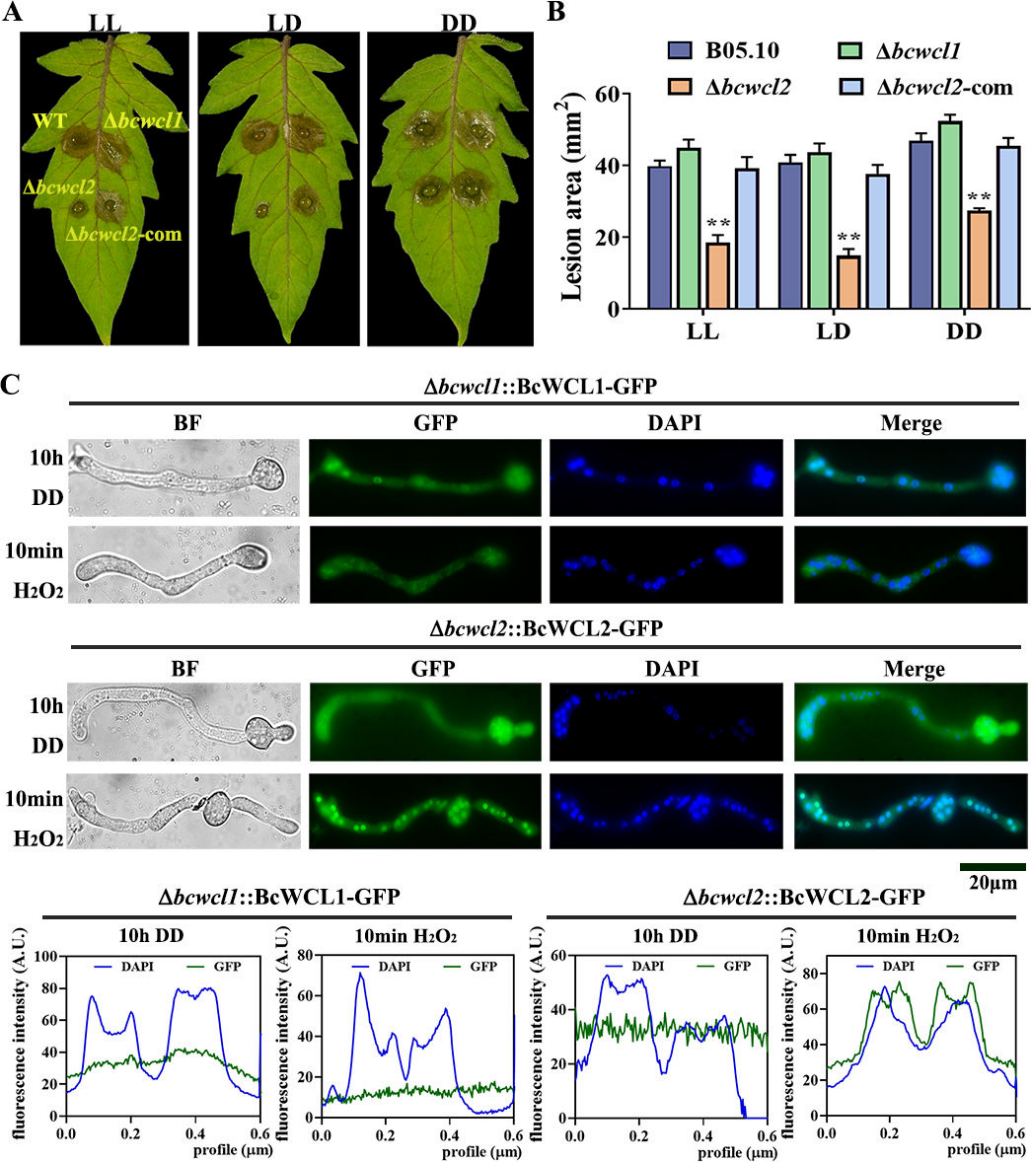
BcWCL2 and BcWCL1 demonstrate inconsistent functions and responses to oxidative stimulus. (**A**) The virulence of the WT, Δ*bcwcl1*, Δ*bcwcl2*, and ∆*bcwcl2*-com strains on detached tomato leaves. The inoculated leaves were maintained under different light conditions at 23°C for 48 h. (**B**) Lesion areas on tomato leaves formed under different light conditions. (**C**) The subcellular localization of BcWCL1*-*GFP in Δ*bcwcl1*::BcWCL1*-*GFP and BcWCL2*-*GFP in Δ*bcwcl2*::BcWCL2-GFP strains in response to 1 mM H_2_O_2_ treatment. Nuclei were visualized using the fluorescent dye Hoechst 33342 (DAPI). Scale bars = 5 µm. The mean values were calculated using data obtained from three independent biological replicates. * denotes that values were significantly different from the WT control values at the *P* < 0.05 level.

Our results further revealed that the Δ*bcwcl2* mutant exhibited reduced virulence when infecting detached tomato (*Solanum lycopersicum*) leaves (Fig. 1), and the differences between WT and ∆*bcwcl2* lesion areas were more significant under LL and LD compared with DD condition. Additionally, the Δ*bcwcl2* mutant showed increased sensitivity to H_2_O_2_ compared with the WT (Fig. S4). In contrast, the Δ*bcwcl1* did not show significant differences in virulence on detached tomato leaves when compared to the WT (Fig. 1A and B). It has been established that BcWCL1 and BcWCL2 could interact with each other and co-localize in the nuclei (45). Interestingly, we found that the ectopically expressed BcWCL2-GFP fusion protein driven by the native promoter of *Bcwcl2* could well complement the virulence phenotypes of ∆*bcwcl2* mutant (Fig. 1A and B), and the fluorescent signal of BcWCL2-GFP responded to H_2_O_2_ treatment by movement from a more cytoplasmic localization into the nuclei. In contrast, the signal of BcWCL1-GFP did not change by H_2_O_2_ treatment towards increased nuclear localization (Fig. 1C). Based on these observations, we propose that BcWCL2 could function either in conjunction with BcWCL1 to regulate light sensing responses, or independently to regulate its unique downstream targets which are not discovered yet.

### BcWCL2 regulates growth, development, and virulence of *B. cinerea*

**Fig 2:**
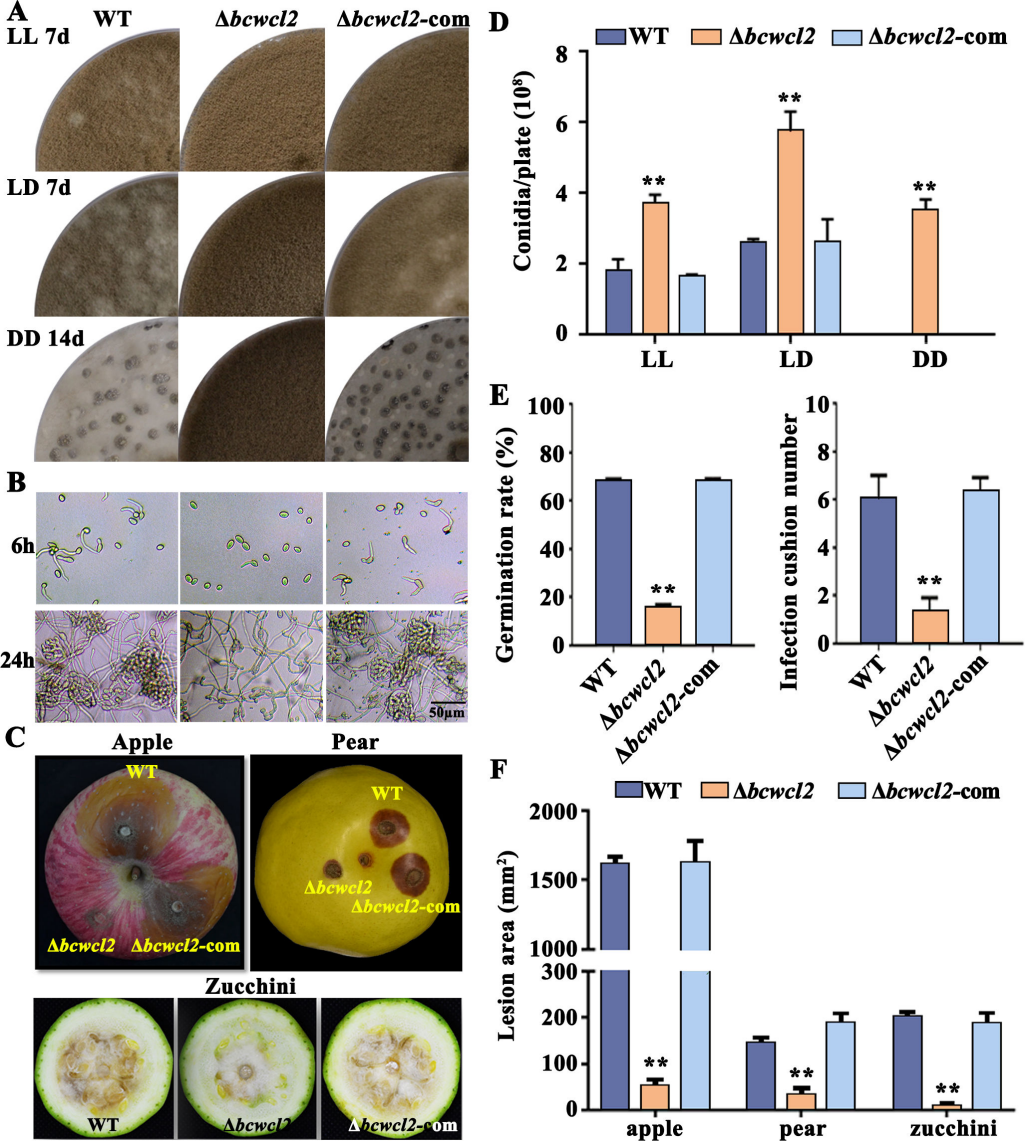
BcWCL2 regulates the development and virulence of *B. cinerea*. (**A**) Conidiation and sclerotium formation strains under different light conditions. *B. cinerea* strains were cultured on CM under LL (constant light), LD (12 h light, 12 h dark), and DD (constant dark) conditions. (**B**) Conidial germination and infection cushion development. (**C**) Virulence on apple, pear, and zucchini fruit incubated at 23℃ under DD conditions for 3 days. (**D**) Sporulation under LL, LD, and DD conditions. (**E**) Spore germination rate and infection cushion formation. The number of infection cushions produced after 24 h were quantified in fields of view measuring 200 µm × 100 µm (*n* = 20). (**F**) Lesion areas on apple, pear, and zucchini. Data represent the means ± standard deviations (SDs) of three independent experiments. Data were analyzed by one-way ANOVA. * and ** denote that the values obtained for Δ*bcwcl2* were significantly different from those of the WT at *P* < 0.05 and *P* < 0.01.

To establish the role of BcWCL2 in *B. cinerea*, we determined mycelial growth of the WT, *∆bcwcl2*, and complemented transformant (Δ*bcwcl2*-com) strains on solid complete medium (CM) under LL, LD, and DD conditions. The ∆*bcwcl2* mutant was unable to form sclerotia under DD but constitutively produced conidia under all assessed conditions, and its sporulation was higher than that of the WT under LL and LD conditions (Fig. 2A and D). These light/dark-regulated reproductive phenotypes due to loss of BcWCL2 are consistent with those found in the ∆*bcwcl1* mutant (Fig. S3A), which is in agreement with the earlier studies (46), thus confirming that these two components of WCC are indeed both required for light sensing in *B. cinerea*. Moreover, the germination rate of the conidia and the number of infection cushions formed on glass slides were strongly reduced in the *∆bcwcl2* mutant (Fig. 2B and E). Inoculation assays with apple, pear, and zucchini fruit under DD conditions verified that the Δ*bcwcl2* mutant was less virulent than the WT, while its virulence defect was rectified by complementation with the intact *Bcwcl2* gene (Fig. 2C and F), suggesting that *Bcwcl2* is required for full virulence of *B. cinerea* on varied plant materials

### The virulence defect in ∆*bcwcl2* is associated with reduced citrate secretion

**Fig 3:**
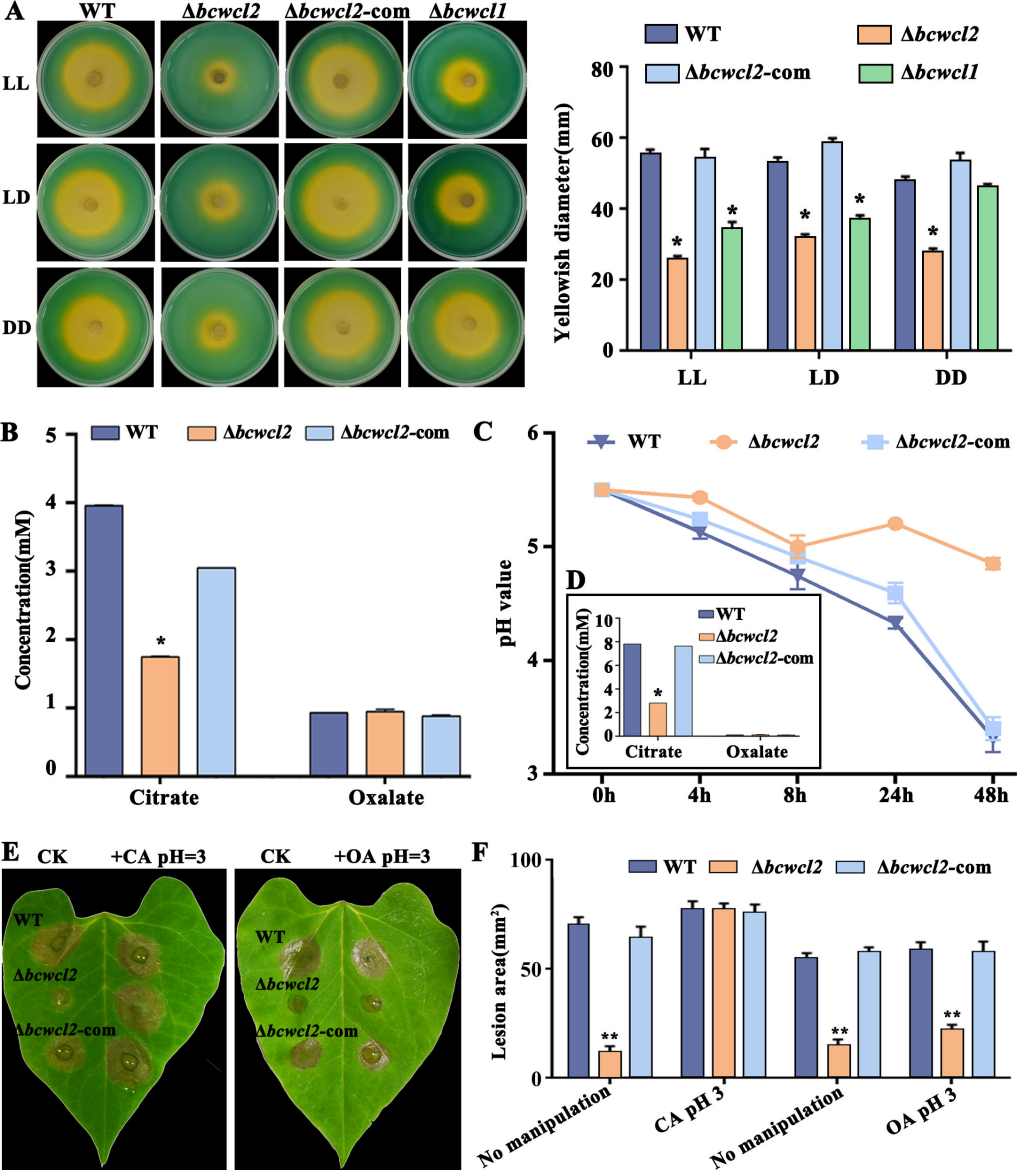
BcWCL2 regulates citrate secretion and affects virulence. (**A**) The ability of *B. cinerea* to acidify the medium. The medium exhibits a green coloration at pH values of approximately 7, whereas a yellowish coloration is observed at pH values below 6. The indicated strains were grown on CM supplemented with bromophenol blue (pH 7.0) for 72 h under LL (constant light), LD (12 h light and 12 h dark), and DD (constant dark) conditions. (**B**) BcWCL2-dependent secretion of citrate and independent secretion of oxalate into CM medium. (**C**) A line graph of pH changes in inoculated droplets on green bean leaves. (**D**) Secretion of citrate and oxalate into the infection droplets on detached green bean leaves. (**E**) Lesion formation of the WT, Δ*bcwcl2*, and ∆*bcwcl2*-com strains on detached green bean leaves with exogenous application of citric acid (CA) or oxalic acid (OA). (**F**) Lesion areas on the detached green bean leaves at 48 h post-inoculation. * and ** denote values that are significantly different from the WT control values at the *P* < 0.05 and *P* < 0.01 levels, respectively.

The acidification ability of *B. cinerea* on medium was influenced by BcWCL1 and BcWCL2. The Δ*bcwcl2* mutant showed decreased citrate secretion when incubated in CM compared to the WT strain, independent on the light conditions (Fig. 3A). On the other hand, there were no significant differences in oxalic acid secretion between the Δ*bcwcl2* mutant and the WT strain (Fig. 3B). The acidification capacity of the Δ*bcwcl1* mutant was lower under LL and LD conditions compared to the WT strain, but not significantly reduced under DD conditions (Fig. 3A). When droplets with conidial suspensions in CM were inoculated on detached leaves, the WT acidified the droplets to pH 3.5, whereas the Δ*bcwcl2* acidified only to pH 5.0 and secreted less citrate than the WT, while equally low amounts of oxalic acid were secreted by Δ*bcwcl2* and the WT (Fig. 3C and D).

To examine how acidification affects the virulence of the Δ*bcwcl2* strain, suspensions of spores in Gamborg B5 medium at pH 5.5 were inoculated on green bean leaves. For pH manipulation, oxalic acid or citric acid was added to the inoculation droplets to reduce the pH to 3. The results revealed that pH adjustment with oxalic and citric acids had no significant effects on the full virulence of the WT, but partially and fully restored Δ*bcwcl2* virulence, respectively (Fig. 3E and F). These findings indicated that the reduced virulence of Δ*bcwcl2* could be attributed to a defect in citrate secretion at the site of infection.

**Fig 4:**
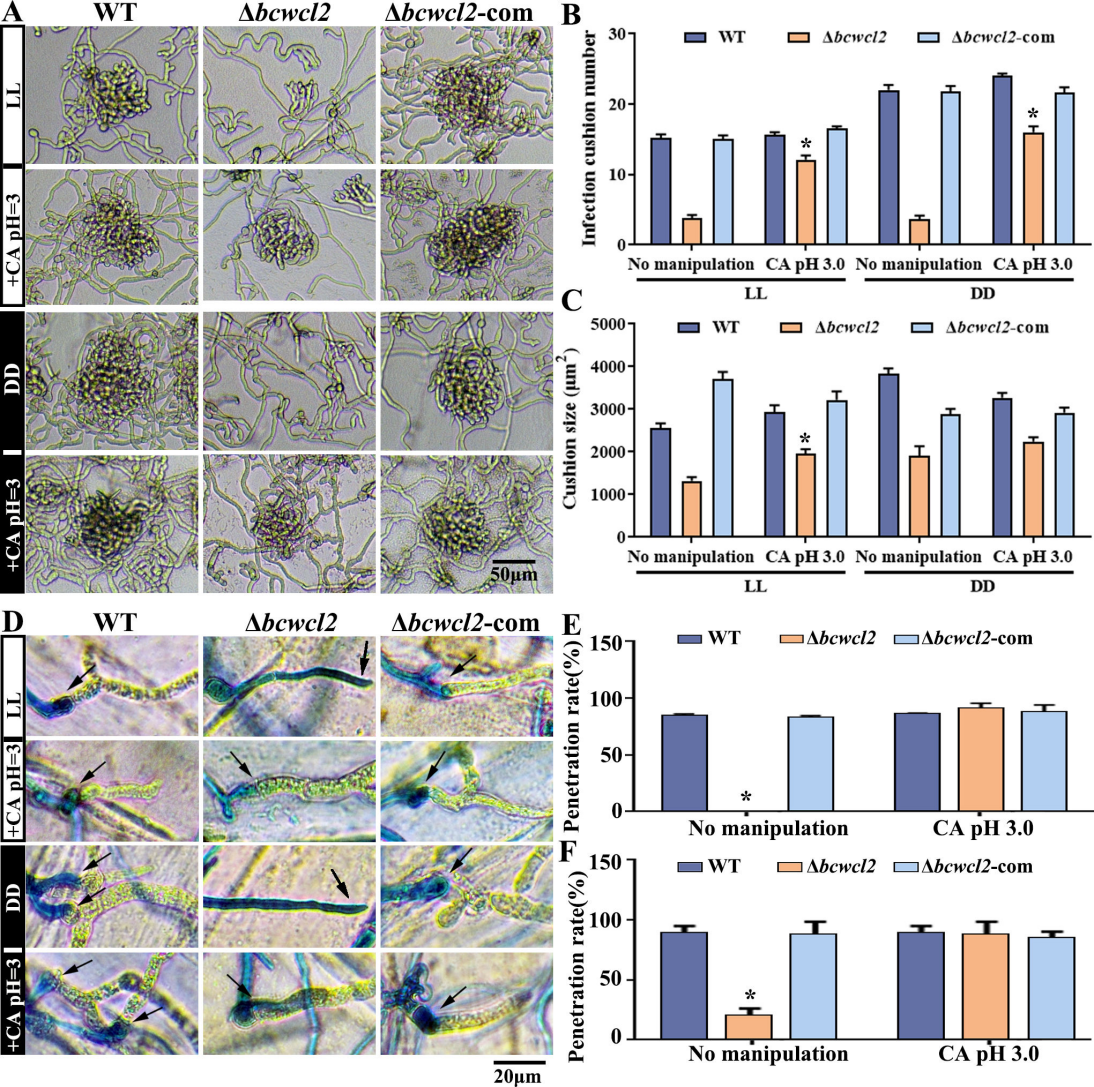
BcWCL2 regulates infection cushion formation and plant penetration by mediating citrate secretion. (**A**) Formation of infection cushions by *B. cinerea* strains (after 16 h). LL, constant light; DD, constant dark; CA, exogenous application of citric acid. (**B and C**) Quantification of the numbers and sizes of infection cushions produced by the indicated strains in the absence or presence of CA in a field of view measuring 200 µm × 100 µm (*n* = 20). (**D**) Hyphal penetration of *B. cinerea* strains inoculated on onion inner epidermis and maintained under LL and DD conditions for 16 h, followed by lactophenol blue staining to visualize the infection hyphae. (**E and F**) Quantification of the penetration rates of the test strains on onion epidermis in the absence or presence of exogenous CA under LL and DD conditions, respectively. Data represent the means ± standard deviations (SDs) from three independent experiments. *, significant difference at *P* < 0.05.

Under both light and dark conditions, the Δ*bcwcl2* strain showed significant defects in the formation of infection cushions. Intriguingly, the application of citric acid (pH 3) partially restored infection cushion development (Fig. 4A through C). The functionality of infection structures (appressoria and infection cushions) can be tested on heat-killed onion epidermis layers. Interestingly, the epidermis penetration capacity of the Δ*bcwcl2* mutant was strongly impaired both under constant light or dark conditions but could be restored by the application of exogenous citric acid (pH 3) (Fig. 4D through F). These results, thus, indicate that BcWCL2 contributes to the regulation of infection cushion development and hyphal penetration of the host tissue and that this regulation is associated with the secretion of citrate.

### BcWCL2 regulates the tolerance to oxidative stress

DAB (3,3ʹ-diaminobenzidine) staining indicated that the levels of reactive oxygen species (ROS) in the infected zones of the ∆*bcwcl2* strain were lower than those in tissues infected with the WT (Fig. S4A). Whereas growth of the Δ*bcwcl2* strain was slightly inhibited on media supplemented with 0.7 M sorbitol and 0.7 M NaCl, this strain was significantly more inhibited than the WT in the presence of 8 mM H_2_O_2_ and 3 mM FeSO_4_. However, we detected no significant differences between Δ*bcwcl2* and WT strains in growth inhibition by the oxidative stress inducer menadione [an artificial source of superoxide radicals (O^−^_2_·)] (Fig. S4B). *In vitro* culture revealed lower levels of ROS in the exudates of the ∆*bcwcl2* strain, whereas this mutant appeared to accumulate more ROS in the mycelium (Fig. S4C). These findings indicate that the loss of BcWCL2 may result in excessive accumulation of ROS in mycelia and endogenous oxidative stress, leading to hypersensitive to additionally exogenous ROS which is produced during host plant defense responses.

### Loss of BcWCL2 causes large-scale transcriptional changes in *B. cinerea in planta*

**Fig 5:**
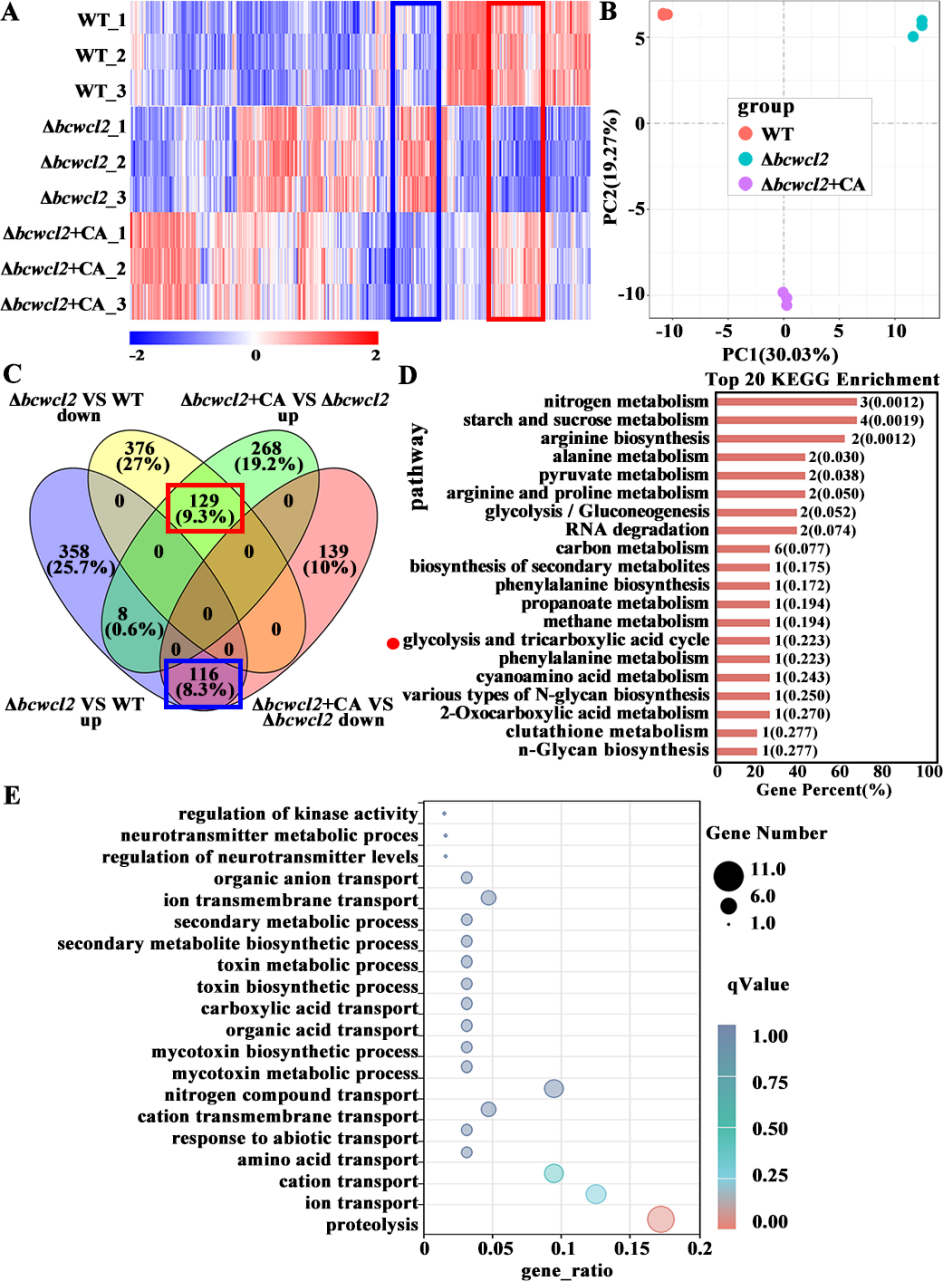
Analysis of global transcriptome genes regulated by BcWCL2. (**A**) Gene expression heatmap of three sample groups: WT, Δ*bcwcl2*, and Δ*bcwcl2* + CA. (**B**) Principal component analysis of WT, Δ*bcwcl2*, and Δ*bcwcl2* + CA samples. (**C**) A Venn diagram of the differentially expressed genes (DEGs) obtained from the comparisons between Δ*bcwcl2* and WT and Δ*bcwcl2* + CA and Δ*bcwcl2*. The 116 DEGs (in blue box) that were up-regulated in Δ*bcwcl2* compared with the WT, but suppressed to WT levels in the Δ*bcwcl2* + CA sample, were grouped in Cluster1. The 129 DEGs (in red box) that were down-regulated in Δ*bcwcl2* compared with WT, but recovered to WT levels in the Δ*bcwcl2* + CA sample, were grouped in Cluster2. (**D**) The most significantly enriched Kyoto Encyclopedia of Genes and Genomes metabolic pathways of the Cluster1 and 2 DEGs. Numbers adjacent to the bars indicate the *q*-values. (**E**) The enrichment analysis identified the most significantly enriched GO terms for Cluster2 DEGs. The gene ratio was calculated as the ratio of the number of differential genes annotated to the GO number to the total number of differential genes.

To assess transcriptional changes in the WT and Δ*bcwcl2* strains of *B. cinerea* during host infection, we performed RNA sequencing-based transcriptome analyses of these strains, as well as the Δ*bcwcl2* strain supplemented with citric acid, at 48 h after infection of green bean leaves under LD conditions. Among the 11,701 genes annotated in the *B. cinerea* B05.10 genome (50), we detected a large number of differentially expressed genes (DEGs) (defined as showing a |log2 Fold change| > 1 and padj < 0.05). Compared with the WT, 116 genes were up-regulated in Δ*bcwcl2* and down-regulated in Δ*bcwcl2* + citric acid (CA) (Cluster1 genes), and 129 genes were down-regulated in Δ*bcwcl2* and up-regulated in Δ*bcwcl2* + CA (Cluster2 genes; Fig. 5A and C). Principal component clustering analysis indicated that the Δ*bcwcl2* + CA group was situated between the WT and Δ*bcwcl2* groups in the first principal component (PC1) dimension (Fig. 5B). Kyoto Encyclopedia of Genes and Genomes (KEGG) pathway enrichment analysis revealed that Cluster1 and Cluster2 are both significantly enriched in glycolysis and the tricarboxylic acid (TCA) cycle functions (Fig. 5D). Furthermore, Gene Ontology (GO) functional clustering analysis revealed that the products of the DEGs contained secreted proteases and membrane transport proteins encoding genes as most highly enriched GO terms (Fig. 5E).

**Fig 6:**
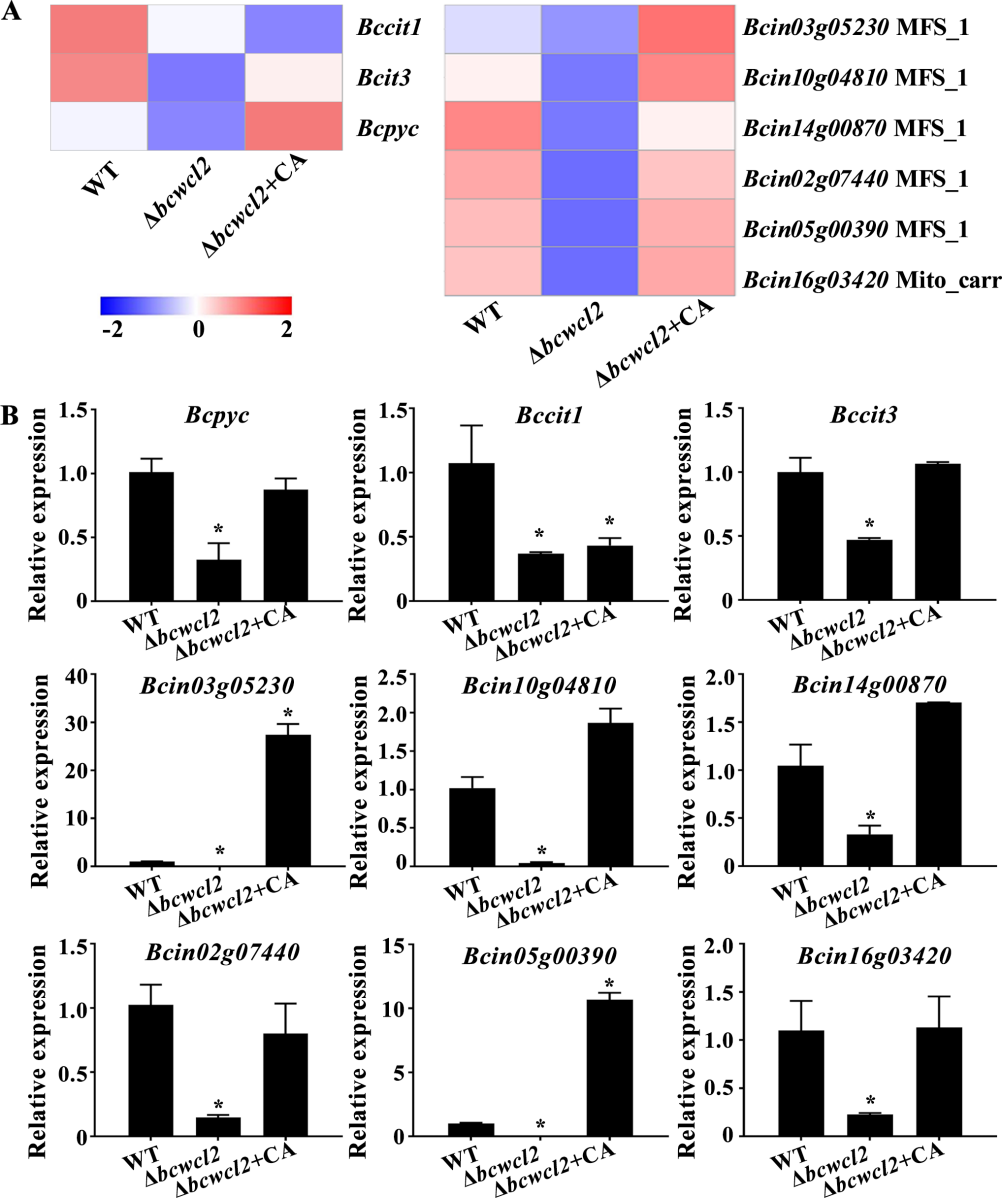
Transcript levels of citrate synthesis- and transport-related genes are regulated by BcWCL2. (**A**) Expression profiles derived from the transcriptome data of the key genes associated with citrate synthesis and transport in *B. cinerea*. (**B**) qRT-PCR analysis confirmed the expression levels of the selected genes (*Bccit1*, *Bccit3*, *Bcpyc*, *Bcin03g05230*, *Bcin10g04810*, *Bcin14g00870*, *Bcin02g07440*, *Bcin05g00390*, and *Bcin16g03420*). Data are presented as the means and standard errors of three biological replicates (*n* = 9). *, significant difference at *P* < 0.05.

Among the genes showing significantly down-regulated expression levels in the Δ*bcwcl2* mutant were two genes encoding two isoforms of citrate synthases, *Bccit1* (*Bcin02g02750*) and *Bccit3* (*Bcin09g00650*), and pyruvate carboxylase *Bcpyc* (*Bcin01g09950*), which are key enzyme involved in the synthesis of citrate. However, upon exogenous application of citric acid (pH 3), we detected an elevation of *Bccit3* and *Bcpyc* expression in the Δ*bcwcl2* strain to levels comparable with the WT. In Δ*bcwcl2*, the expression levels of major facilitator superfamily (MFS) transporter genes were also down-regulated. Similar to the previously mentioned citrate synthesis genes, the expression levels of these genes in Δ*bcwcl2* increased to or even surpassed, WT levels in response to the application of citric acid (pH 3) (Fig. 6A). qRT-PCR analysis confirmed that genes associated with citrate were significantly down-regulated in the Δ*bcwcl2* strain, and up-regulated, except for *Bccit1*, upon application of citric acid (Fig. 6B). Collectively, these findings indicate that BcWCL2 regulates genes involved in citrate synthesis and transport.

### Expression of the global regulator BcVEL1 is regulated by BcWCL2

In *B. cinerea*, BcVEL1, together with the other Velvet complex proteins BcVEL2 and BcLAE1, has been established to serve as a core regulator of organic acid secretion, host tissue acidification, and secretion of hydrolytic proteins (15, 51, 52). BcVEL1 mutation could result in down-regulation of the virulence-related CAZymes, proteases, and other lytic enzymes (15). Interestingly, we observed downregulation in the expression levels of several protease and CAZyme-coding genes in Δ*bcwcl2*, and exogenous application of citric acid could recover the expression levels of these genes to comparable or even higher levels as observed in the WT (Fig. S5). Because of the similarity of Δ*bcvel1* and Δ*bcwcl2* phenotypes, we wondered whether BcWCL2 could influence acid secretion via regulation of *Bcvel1*.

**Fig 7:**
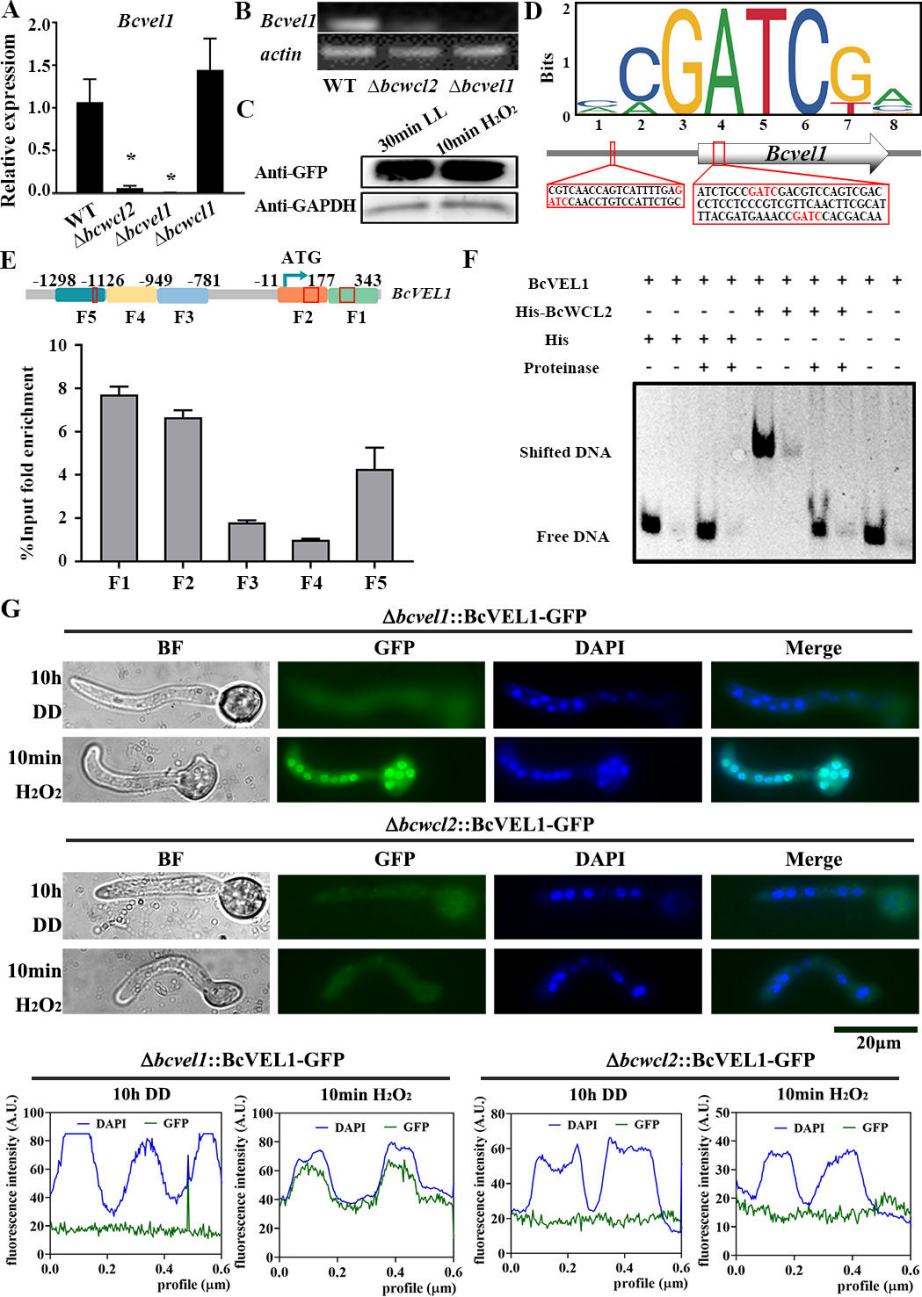
BcWCL2 directly regulates *Bcvel1*. (**A**) Changes in *Bcvel1* expression in WT, Δ*bcwcl2*, Δ*bcvel1,* and Δ*bcwcl1* strains were confirmed by quantitative RT-PCR analyses. * denotes that values were significantly different from the WT control values at the *P* < 0.05 level. (**B**) Changes in *Bcvel1* expression in WT, Δ*bcwcl2*, and Δ*bcvel1* strains confirmed by semi-quantitative RT-PCR analyses. (**C**) Detection of BcWCL2-GFP levels in the Δ*bcwcl2*::BcWCL2-GFP strain using western blotting. The Δ*bcwcl2*::BcWCL2-GFP strain was cultured in liquid CM for 48 h and then subjected to H_2_O_2_ treatment for 10 min or light treatment for 30 min. Proteins were detected using the anti-GFP antibody. Antibodies against GAPDH were employed as loading controls. (**D**) The JASPAR transcription factor binding site database (http://jaspar.genereg.net) was used to determine the degree of sequence conservation, which is indicated by the relative height of each nucleotide (shown in the 5′ to 3′ direction on the *x*-axis) with a maximal score of 2 (as indicated on the *y*-axis). GATC motifs in the BcWCL2 binding sites of *Bcvel1* are shown in the red box. (**E**) ChIP-qPCR assays demonstrated the presence of GFP-tagged BcWCL2 at the promoter of the *Bcvel1* gene. (**F**) Electrophoretic mobility shift assays verified the binding of BcWCL2 to the *Bcvel1* promoter region. Purified His-BcWCL2 fusion protein was incubated with *Bcvel1* promoter DNA fragment containing GATC motif, and His tag was used as control. (**G**) The subcellular localization of BcVEL1*-*GFP in Δ*bcvel1*::BcVEL1*-*GFP and Δ*bcwcl2*::BcVEL1-GFP strains in response to 1 mM H_2_O_2_ treatment. Nuclei were visualized using the fluorescent dye Hoechst 33342 (DAPI). Scale bars = 5 µm.

Analysis of *Bcvel1* expression levels in the Δ*bcwcl2*, Δ*bcwcl1,* and WT strains using qRT-PCR revealed that *Bcvel1* was significantly down-regulated compared to WT at the initial stage of infection cushion formation in the Δ*bcwcl2* strain. In contrast, the loss of BcWCL1 did not affect the expression levels of *Bcvel1* (Fig. 7A and B). Both the Δ*bcwcl2* and Δ*bcvel1* strains formed significantly fewer infection cushions than WT after a 16 h culture in CM, PDA, GB5, and MM liquid media under DD conditions. However, the application of exogenous citric acid restored the infection cushion formation capacity of the Δ*bcwcl2* and Δ*bcvel1* strains. The formation of infection cushions by the Δ*bcwcl1* strain was similar to that of WT (Fig. S6).

In addition, it was found that the promoter region of *Bcvel1* contains the GATC motif of the BcWCL2-target sites (Fig. 7D). Chromatin immunoprecipitation and quantitative PCR (ChIP-qPCR) and electrophoretic mobility shift assay analyses revealed binding of BcWCL2 to the *Bcvel1* promoter (Fig. 7E and F). BcVEL1-GFP was found to be distributed throughout the cytoplasm in the hyphae of the Δ*bcvel1*::BcVEL1-GFP complemented strain when cultured in dark, whereas in response to treatment with 1 mM H_2_O_2_ for 10 min, BcVEL1-GFP was mainly located within the nuclei. Deletion of BcWCL2 resulted in a weaker BcVEL1-GFP signal and the inability of BcVEL1-GFP to translocate into the nuclei (Fig. 7C and G). Collectively, these findings provide evidence that BcWCL2 may directly regulate the expression of *Bcvel1* to influence infection cushion formation and virulence.

### Citrate production regulated by BcWCL2 plays antioxidant roles during the infectious development of *B. cinerea*

**Fig 8:**
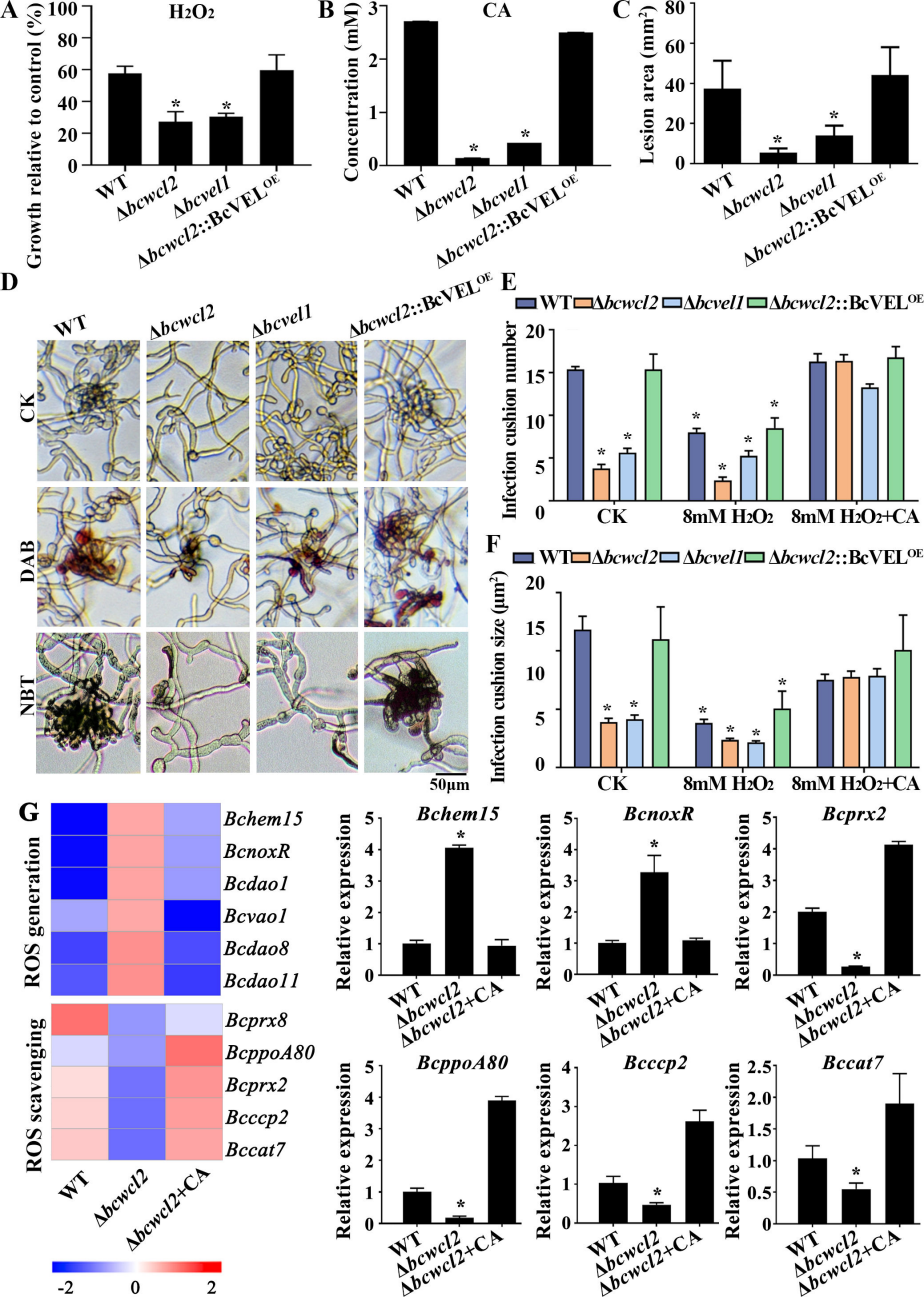
BcWCL2 regulates citrate secretion and maintains redox homeostasis. (**A**) The colony diameters of WT, Δ*bcwcl2*, Δ*bcvel1*, and Δ*bcwcl2*::BcVEL1^OE^ strains grown on CM supplemented with oxidative stress agents were compared with their growth on CM without oxidative stress after 48 h. Relative growth was measured on CM plates supplemented with 8 mM H_2_O_2_ compared to growth on unsupplemented CM plates. (**B**) Citric acid (CA) secretion of the WT, Δ*bcwcl2*, Δ*bcvel1*, and Δ*bcwcl2*::BcVEL1^OE^ strains cultured in liquid CM for 48 h. (**C**) Lesion areas of the WT, Δ*bcwcl2*, Δ*bcvel1*, and Δ*bcwcl2*::BcVEL1^OE^ strains on green bean leaves. (**D**) ROS production during infection-related *in vitro* development of the WT, Δ*bcwcl2*, Δ*bcvel1*, and Δ*bcwcl2*::BcVEL1^OE^ strains detected by DAB or NBT staining. Disruption of *Bcwcl2* or *Bcvel1* reduced infection cushion development, and ROS accumulation was detected by DAB or NBT staining. (**E**) Effects of exogenous application of 8 mM H_2_O_2_ and CA on infection cushion numbers in the WT, Δ*bcwcl2*, Δ*bcvel1*, and Δ*bcwcl2*::BcVEL1^OE^ strains in a field of view measuring 200 µm × 100 µm (*n* = 20). (**F**) Effects of exogenous application of 8 mM H_2_O_2_ and CA on infection cushion size in the WT, Δ*bcwcl2*, Δ*bcvel1*, and Δ*bcwcl2*::BcVEL1^OE^ strains. (**G**) Relative expression of ROS generation- and scavenging-associated genes in the WT, Δ*bcwcl2*, and Δ*bcwcl2* + CA strains. Data represent the means ± standard deviations (SDs) of three independent experiments. *, significant difference at *P* < 0.05.

To evaluate the role of BcWCL2 and BcVEL1 in the early stages of *B. cinerea* infection, we compared mycelial growth, citrate secretion, and virulence of the Δ*bcwcl2*, Δ*bcvel1*, and Δ*bcwcl2*::BcVEL1^OE^ (*Bcvel1* overexpressed in a Δ*bcwcl2* background) strains. The vegetative growth of Δ*bcwcl2* and Δ*bcvel1* on CM was significantly more inhibited than the growth of WT by exogenous application of 8 mM H_2_O_2_ (Fig. 8A). Intriguingly, the secretion of citrate by the Δ*bcwcl2* and Δ*bcvel1* strains was lower than that of the WT and Δ*bcwcl2*::BcVEL1^OE^ strains (Fig. 8B). When the *B. cinerea* strains were inoculated on green bean leaves, both Δ*bcwcl2* and Δ*bcvel1* showed markedly reduced virulence compared to WT and Δ*bcwcl2*::BcVEL1^OE^ strains (Fig. 8C). ROS production in these strains was detected by DAB and nitroblue tetrazolium (NBT) staining, which revealed that ROS had accumulated in the infection cushions of the WT and Δ*bcwcl2*::BcVEL1^OE^ strains, whereas the hyphae of Δ*bcwcl2* and Δ*bcvel1*, characterized by considerably fewer infection cushions, were observed to show lower ROS staining intensities (Fig. 8D). Collectively, these findings indicate that *Bcwcl2* and its downstream target *Bcvel1* are required for coping with elevated levels of ROS, which may accumulate during the formation of infection cushions.

Moreover, H_2_O_2_ treatment retarded infection cushion formation in the WT, Δ*bcwcl2*, Δ*bcvel1*, and Δ*bcwcl2*::BcVEL1^OE^ strains, whereas the application of citric acid could restore the defects caused by H_2_O_2_ treatment (Fig. 8E and F). Similar to the results obtained for ROS production, we detected no significant difference among the WT, Δ*bcwcl1*, and Δ*bcwcl2*::BcVEL1^OE^ strains with respect to infection cushion numbers, and application of antioxidative agents (3 mM ascorbic acid or 10 mM sodium phenylpropionate) also restored infection cushion formation capacity in Δ*bcwcl2* and Δ*bcvel1* (Fig. S7). Subsequently, we examined the expression of genes associated with ROS synthesis and scavenging. In the Δ*bcwcl2* strain, the expression of the ROS generation-related gene *BcnoxR*, a regulatory subunit of NOX, was up-regulated. Conversely, the expression of genes encoding ROS scavenging enzymes, *Bcprx2*, *BcppoA80*, *Bcccp2*, and *Bccat7* was down-regulated in the mutant. However, we detected no significant differences between the Δ*bcwcl2* + CA and WT strains regarding the expression of these genes (Fig. 8G). These findings, thus, tend to indicate that the production of citrate regulated by BcWCL2 and BcVEL1 may play antioxidant roles in *B. cinerea* to maintain oxidative homeostasis during infection cushion development and host invasion.

## DISCUSSION

Light signals play essential roles in regulating the morphology and physiology of fungi (20). One example, in this regard, is the well-conserved fungal blue light receptor WCC, consisting of the two GATA-type transcription factors WC-1 and WC-2 (26). The regulatory mechanisms of these WCC components could be either dependent on a photoreceptor complex, or on their contributions as individual transcription regulators. In this study, we demonstrated that the BcWCL2 protein regulates citrate secretion in the gray mold fungus *B. cinerea*, thereby possibly contributing to the maintenance of redox homeostasis during infection cushion formation, and is, thus, required for full virulence of this fungus.

It has been established that fungal pathogens can enhance their infectivity by up- or down-regulation of the pH of host tissues, which they achieve by releasing organic acids or ammonia. Based on this activity, these fungi can be classified into two groups, namely, acidic and alkaline (53). Among the acidic fungi are certain post-harvest fungal pathogens besides *B. cinerea*, including *Penicillium digitatum*, *P. italicum*, *P. expansum*, and *S. sclerotiorum*, which have been observed to secrete organic acids, such as oxalic, malic, and citric acids, during infection (54, 55), and the acidification of host tissues plays a key role in promoting infection of these pathogenic species (9). The findings of some early studies in this regard have indicated that *B. cinerea* acidifies host tissues by secreting oxalic acid to enhance its virulence (56). However, later studies showed that the oxalic acid-deficient Δ*bcoahA* mutant induced only slightly reduced acidification of tomato leaf tissue and marginally reduced in virulence (15). In another study, it was found that *B. cinerea* secretes citrate during the early infection stages of sunflower cotyledons, whereas oxalic acid was detected only during later infection stages (14). Previous studies with the Δ*bcwcl1* and OE::*bcwcl1 + bcwcl2* mutants have shown varying abilities to acidify the pH 7.5 CM medium over a 7-day period, suggesting that the WCC may play a role in regulating oxalic acid production. Furthermore, when an antioxidant (ascorbic acid) was added, it was able to restore the light-dependent reduction in growth observed in Δ*bcwcl1* under both LL and LD conditions, bringing it back to levels similar to the wild-type. This suggests that the WCC may function as response to oxidative stress induced by excessive exposure to light (46).

As for virulence, it is reported that disruption of *Bcwcl1* does not significantly impact on virulence of *B. cinerea* in the dark but notably reduce the fungal virulence under light conditions (46). However, the Δ*bcwcl1* mutant in the present study showed no significant attenuation in virulence on tomato (M82) leaves under either light or dark conditions. The discrepancy may be due to the different host materials being used for virulence assessment, as the early study deployed *Arabidopsis thaliana* and green bean leaves (46). On the other hand, it is worth noting that loss of *Bcwcl2* resulted in significantly reduced virulence of *B. cinerea* on varied host materials irrespective of light conditions. These observations led us to hypothesize that *Bcwcl1* and *Bcwcl2* may function differentially to affect the virulence of *B. cinerea*.

It is well recognized that acidification of host tissue by *B. cinerea* could facilitate its successful infection progression (15). Nonetheless, the precise connection between impaired acidification and alterations in organic acid secretion and gene expression remains unclear and warrants further investigation (15, 57). In the present study, we confirmed that *B. cinerea* primarily secretes citrate during the early stages of growth and infection of *Phaseolus* leaves and that ROS accumulate intracellularly during the development of infection cushions. The formation of infection cushions was attenuated in the BcWCL2-deletion mutant, whereas the application of citric acid restored the defective processes in the mutant and also facilitated the recovery of ROS tolerance in Δ*bcwcl2*. Accordingly, we speculate that the citrate produced by *B. cinerea* plays a potential role in maintaining redox homeostasis during the stage of infection cushion development.

To gain a more in-depth understanding of the mechanisms underlying the restoration of virulence in the Δ*bcwcl2* in response to exogenous citric acid, we conducted a transcriptome analysis with *Phaseolus* leaves infected with both the WT and Δ*bcwcl2* strains. Transcriptome analysis also revealed that genes for citrate synthases (*Bccit3/Bccit1*) and pyruvate carboxylase (*Bcin09g02790*), which promotes synthesis of the citrate precursor oxaloacetate, were significantly down-regulated in Δ*bcwcl2*. The expression of these genes was restored upon supplementation with citric acid. One of these genes is *BccexA* (*Bcin03g05230*), which is an ortholog of the primary plasma membrane transporter of citrate in *A. niger*. Overexpression *A. niger cexA* results in a significant increase in citrate secretion (58). Additionally, *Bcin14g00870* and *Bcin05g00390* are homologous of *Bcin03g05230*, indicating their potential involvement in the expulsion of organic acids. These findings suggest that BcWCL2 might regulate the transport and efflux of citrate and other metabolites.

ROS plays a dual role in the life cycle of *B. cinerea*. On one hand, they can induce hypersensitive response-mediated cell death in plant hosts, facilitating the pathogen’s necrotrophic invasion (6, 59). On the other hand, the fungus must prevent excessive accumulation of ROS from damaging its own metabolism and mitochondria (60). Thus, *B. cinerea* has adapted to survive, while making use of, the oxidative burst during its infestation of host plants, and under such hostile conditions, the fungus may form multicellular infection cushions (57). It has been recently found that infection cushions secrete many fungal virulent effectors to aid in plant penetration and colonization, and they produce significant amounts of ROS (4). In the present study, we observed that deletion of *Bcwcl2* in *B. cinerea* resulted in hyperaccumulation of ROS in the fungal mycelium, potentially leading to elevated endogenous oxidative stress. However, the application of exogenous citric acid or the antioxidant ascorbic acid, along with the acidic preservative sodium benzoate, to the Δ*bcwcl2* strain resulted in the restoration of complete infection cushion development. Similarly, when applied during germination of the WT strain, hydrogen peroxide led to a suppression of infection cushion development, which was again restored by the application of exogenous citric acid. Furthermore, we demonstrated that the loss of BcWCL2 resulted in the down-regulated expression of the ROS scavenging genes *Bccat7*, *Bcccp2*, *Bcprx2*, and *BcppoA80*, but in up-regulation of ROS synthesis, such as *BcnoxR* (the regulatory subunit of NOX) (61), lipoxygenase *Bclox*, and iron chelatase *Bchem15*. Again, in response to the addition of exogenous citric acid, the expression levels of these genes returned to levels comparable to those observed in the WT strain. These findings accordingly indicate that citrate secretion, regulated by BcWCL2, plays a pivotal role in maintaining the balance of ROS during the spore germination, infection cushion formation, and early infection stages of *B. cinerea* pathogenesis.

The global regulator BcVEL1 (together with BcVEL2 and BcLAE1) of *B. cinerea* has been established to be involved in the regulation of reproductive development, secondary metabolism, and virulence (15, 51). BcVEL1 plays a role in the secretion of citrate via large-scale transcriptional regulation during the infection process. It contributes to maintaining a low pH environment, thereby promoting the expression of virulence factors such as acid hydrolase although the specific regulatory mechanisms of citrate secretion have yet to be clarified (15). Loss of BcVEL1 and BcWCL1/2 proteins seems to result in similar phenotypes, including constitutive sporulation, inability to form sclerotia in the dark, impaired acidification, oxidative tolerance, and virulence (15, 46, 51). In *Aspergillus nidulatus*, VeA and LreB have been found to interact with the red light phytochrome receptor FphA, playing a role in red light-dependent regulation (62). In the present study, we discovered that BcWCL2 transcriptionally regulates *Bcvel1* expression levels. In contrast, the earlier report showed that the expression level of *Bcwcl2* in the Δ*bcvel1* mutant was comparable to the WT strain (52). Furthermore, our genetic data demonstrated that overexpression of *Bcvel1* in the Δ*bcwcl2* strain could restore the phenotypic defects including the secretion of citrate, maintenance of redox homeostasis, infection cushion development, and virulence. Collectively, BcWCL2 is likely epistatic to BcVEL1 in the regulation of citrate synthesis and virulence in *B. cinerea*.

However, ectopically overexpressing *Bcvel1* in the Δ*bcwcl2* mutant failed to restore photoresponsive defects of the mutant, including no sclerotium formation and always sporulation phenotypes, which could be due to deregulation of some other downstream target genes of WCC, like the BcLTFs that are responsible for determining the light responsive phenotypes (47, 63, 64). Otherwise, it is also quite important to note that the restoration of light-responsive phenotypes in Δ*bcwcl1* mutant requires adding-back the copy at its native genomic location, while virulence phenotypes can be restored also by a copy from another location (46). In the future study, it is definitely worth evaluating the effects of integration sites of overexpressing target genes, like *Bcvel1*, on the resulted phenotypes.

Collectively, this study has revealed that BcWCL2 plays a pivotal role in regulating oxidative homeostasis, infection cushion formation, the TCA cycle, citrate synthesis, and virulence of *B. cinerea*. The secretion of citrate, regulated by BcWCL2 transcription, may contribute to promoting *B. cinerea* survival against the oxidative stress encountered during the early infection process, ultimately facilitating the development of host plant disease. These findings provide significant clues for understanding of the mechanisms of acidification by *B. cinerea* and its role for pathogenesis, and probably of other acidifying necrotrophic pathogens that deserve further investigations.

## MATERIALS AND METHODS

### Fungal strains and culture conditions

The *B. cinerea* strain B05.10 used in this study was kindly donated by Prof. Matthias Hahn of the Kaiserslautern University, Germany. The fungal strains used in the study (listed in Table S1) were cultured on potato dextrose agar (PDA) medium or complete medium (CM) at 23°C under three conditions of continuous light (LL), alternating light and dark (12 h/12 h, LD), and continuous darkness (DD). Under light conditions, sporulation occurs within 7 days, whereas sclerotia form within 14 days under dark conditions. Spores were collected in sterile water, and the total number of spores was calculated using a hemocytometer. Having determined spore numbers, the spore suspensions were centrifuged at 4,500 rpm for 10 min. The spore pellets were resuspended in potato dextrose broth (PDB), CM, Gamborg B5 (brand name, or GB5), and minimal medium (MM) liquid medium. The concentration of the spores was adjusted to 2 × 10^5^ spores/mL for further analysis of spore germination.

### Phylogenetical analysis and multiple alignment

GATA TFs protein sequences were aligned using ClustalW in MEGA7.0 software using default parameters. The consensus neighbor-joining (NJ) tree represents 1,000 bootstrap replications (65). The protein sequences of the GATA TFs were downloaded from Fungal Transcription Factor Database (FTFD). The GATA TFs are classified into seven subgroups in the NJ tree. Comparative analysis of the structural domains in WC2 proteins from different fungal species using the Conserved Domain Architecture Retrieval Tool (https://www.ncbi.nlm.nih.gov/Structure/lexington/lexington.cgi), as verified using the SMART protein database (http://smart.embl-heidelberg.de). Comparative analysis of the WC2 zinc finger structural domains in *B. cinerea* and their homologs using DNAMAN.

### Fungal transformation

For the purposes of deleting target genes in B05.10, we performed polyethylene glycol (PEG)-mediated protoplast transformation (66). The mutant strain obtained was identified by diagnostic PCR using specific primer pairs (Fig. S2), which are listed in Table S2. The GFP-fusion proteins were ectopically expressed in the mutant strains. Molecular diagnostic PCR assays using both DNA and cDNA as templates were carried out to confirm the presence and expression of the GFP-fusion cassettes, respectively.

### Assay of conidial germination and infection cushion formation

To determine spore germination and infection cushion formation in both the WT (B05.10) and mutant strains, respective conidial suspensions (2 × 10^5^ spores/mL) were dropped onto sterilized glass slides and incubated in humidified plastic boxes at 23°C under the LL and DD conditions. After incubation for 6 h, observations were made at 16 or 24 h for the detection of conidial germination and infection cushion formation. For each treatment, three replicates of 300 conidia were randomly selected for statistical analysis, and conidial germination rates were determined using ImageJ software (National Institutes of Health, USA).

### Virulence assay

Fungal spores were suspended in GB5 liquid medium (pH 5.5), supplemented with 2% glucose, and adjusted to 2 × 10^5^ spores/mL to inoculate detached green bean leaves (*Phaseolus vulgaris*) (52), tomato leaves (*Solanum lycopersicum* M82), apple fruit (*Malus pumila*), pear fruit (*Pyrus* spp.), and zucchini fruit (*Cucurbita pepo* L.). At each inoculation point in these plants, 10 µL of the spore suspension was applied, and thereafter, the inoculated leaves and fruits were placed in a moist chamber at 23°C, under LL, LD, and DD conditions. For each fungal strain, we inoculated 30 leaves and fruits, and disease development was recorded at 2 or 4 days. The area of plant tissue decay was measured using ImageJ software. The experiment was repeated five times.

### Analysis of acid production

To evaluate acid production, fresh hyphal plugs of the strains were cultured on CM plates supplemented with bromophenol blue (1 mg/mL). After culturing at 23°C under LL conditions for 3 days, the change in color of the culture medium was photographed. Fresh conidia of the strains were harvested from CM plates with ddH_2_O and the conidial suspension was inoculated into liquid CM, followed by incubation at 23°C with shaking at 180 rpm. The pH of cultures incubated for different lengths of time was monitored daily using a pH electrode (PB-30; Sartorius Corporate, Gottingen, Germany) to determine acid production by the strains. The assay was repeated three times with a set of four replicates.

### Measurement of citric and oxalic acid

To determine the contents of citric and oxalic acids secreted by the different fungal strains *in vitro* and at the sites of infection, fungal mycelia (0.1 g) of the WT and mutant strains were mixed with 1 mL of lysis buffer (Solarbio, Beijing, China), ice ground, and then centrifuged at 11,000 × *g* and 4°C for 10 min. The absorbance of the supernatant thus obtained was measured at 545 nm using a citric acid and oxalic acid detection kit BC2150 (Solarbio, Beijing, China), and the contents of citric and oxalic acids were accordingly calculated.

### Cytological assay

The preparation of conidia and onion epidermal cell layers and lactophenol blue staining of samples were conducted using previously described methods (67), and infected samples were observed under a microscope after 16 h.

### Detection of reactive oxygen species

Quantification of ROS production during conidial germination and infection cushion formation was performed as described previously (68), using ImageJ software.

### Radicals scavenging activity

The ·DPPH radical was used to measure the free radical scavenging capacity of the strains. Fresh conidia of the strains were harvested from CM plates using ddH_2_O, and the conidial suspension was then inoculated into liquid CM. The mixture was incubated at 23°C with shaking at 180 rpm. After incubation, the final solutions were reacted in the dark at room temperature for 30 min. The absorbance value of the solutions was measured at 515 nm. The ·DPPH free radical scavenging capacity kit was purchased from Solarbio Technology Co., Ltd.

### Exogenous acid treatment

To investigate the effects of acidification on spore germination, infection cushion development, and the expression of virulence, we prepared spore suspensions using a 0.1 M citric acid solution (pH 3) or oxalic acid (pH 3). Dilute the configuration of citric acid (excluding citrate) and oxalic acid with ddH_2_O. Soybean leaves were initially treated with this suspension, and at 12 h post-inoculation, the spore suspension at the inoculation site was replaced with a spore-free citric acid solution. As a control, we conducted a similar inoculation using spore-free citric acid solution. This process was repeated at 4 h intervals until 48 h. Green bean plants were propagated in a controlled greenhouse environment at a temperature of 23°C, with a 12 h light and 12 h dark cycle. The virulence test was carried out by inoculating 3-week-old green bean leaves in plastic boxes placed in a humid environment at the same temperature. The area of the lesion formed was measured using ImageJ software. Measurements of the pH of inoculation droplets were performed using a flat pH electrode (PB-30; Sartorius Corporate, Gottingen, Germany). Each treatment was performed in triplicate, and the experiment was repeated three times.

### RNA extraction and qRT-PCR analysis

For RNA isolation, 2-day-old mycelia were obtained from cellophane-covered PDA media, immediately frozen in liquid nitrogen, and ground to a powder. Total RNAs were isolated using TRIzol reagent (Invitrogen, USA) according to a previously described protocol (26) and then purified using a Prime Script RT reagent kit (Perfect Real Time) (Takara Biotechnology, Co., Dalian, China). Real-time PCR amplifications were performed using a CFX96TM RealTime System (BIO-BAD, Inc., California, USA) with Takara SYBR Premix Ex Taq (Takara Biotechnology, Co., Dalian, China). Threshold cycle (Ct) values were obtained during the annealing period of the qRT-PCR amplification cycles. The *actin* gene was selected as the reference gene and was used to normalize the expression levels of the target gene. The relative expression levels were calculated using the 2^−ΔΔCt^ method (69). Each sample was assessed with three biological replicates.

### RNA-seq analysis

For transcriptome data analysis, RNA was extracted following the described method and sequenced using Illumina (150 bp paired reads) at Novagene (Beijing, China). Clean reads were obtained by removing adaptors, reads containing poly-N, and low-quality reads, which were trimmed using cutadapt (v1.16). The trimmed reads were aligned to the B05.10 genome using hisat2 software (v2.0.5). StringTie software (v1.3.3b) was utilized for new transcript assembly and gene prediction. To account for sequencing depth and gene length, the expression value of RNA-seq data is commonly represented by FPKM rather than read count. FPKM corrects for sequencing depth and gene length in a successive manner. Differentially expressed genes (DEGs) (fold change ≥ 2, adjusted *P*-value ≤ 0.05) were identified using DEseq2 software (v1.20.0). Subsequently, these DEGs were subjected to Gene Ontology (GO) and Kyoto Encyclopedia of Genes and Genomes (KEGG) enrichment analysis using clusterProfiler software (v.3.8.1).

### Chromatin immunoprecipitation-qPCR analyses

Chromatin immunoprecipitation (ChIP) was performed based on a previously published protocol with additional modifications (70, 71). Fresh mycelia of the WT and mutant strains were prepared using the aforementioned method and were individually cross-linked with 1% formaldehyde for 10 min. The reaction was stopped by the addition of 1.375 M glycine. Subsequently, the samples were ground in liquid nitrogen and suspended in protease inhibitor-containing lysis buffer for 15 min. After isolating the nuclei, the crosslinked chromatin was sonicated using a Scientz18-A ultrasonic DNA interruptor (10 s on/15 s off for 50 cycles), resulting in small DNA fragments (100–200 bp in length). After centrifugation, the supernatant was diluted with ChIP dilution buffer containing 16.7 mM Tris-HCl (pH = 8), 1.1% Triton X-100, 1.2 mM EDTA, and 167 mM NaCl. The supernatant containing DNA fragments was divided into three aliquots, one of which was used as the input sample and was temporarily stored at −80°C. The remaining two aliquots were used for immunoprecipitation (IP) experiments, one of which was incubated with the corresponding antibody, whereas the other was incubated with anti-IgG1 antibody (Invitrogen, Thermo Fisher, MA1-10406; Waltham, MA, USA) as a mock sample. Before antibody incubation, protein A agarose beads (REF10001D; Invitrogen, Thermo Fisher, Waltham, MA, USA) were added to the IP and mock samples to prevent background binding to the beads. Monoclonal anti-GFP (AT0028, 1:500 dilution; Engibody, USA) was added to the IP samples and used as the IP sample antibody. Protein A agarose beads were added again for a second round of immunoprecipitation in the IP and mock samples. The beads were then washed sequentially using low-salt, high-salt, and LiCl wash buffers, and finally TE buffer, after which, the immunoprecipitated complexes were eluted from the beads using a freshly prepared elution buffer containing 0.5% sodium dodecyl sulfate and 0.1 M NaHCO_3_. Thereafter, 5 M NaCl was added to the chromatin-containing elution buffer followed by incubation overnight at 65°C to facilitate the re-crosslinking of chromatin. The remaining steps for DNA purification were carried out following the manufacturer’s instructions. The immunoprecipitated DNA was subsequently resuspended in 35 µL of water, and ChIP-qPCR was performed to analyze both the immunoprecipitated DNA and the total input DNA. The qPCR procedure was performed as mentioned previously. The PCR conditions consisted of 45 cycles. Relative enrichment levels were determined using the fold enrichment method, in which the ChIP signals were divided by the mock signals, representing the fold increase in signals relative to the background signal. The experiment was repeated three times.

S1: Δ*bcwcl2*::BcWCL2*-*GFP, % input fold enrichment = 2^(−[ΔCt (S1:normalized ChIP) − ΔCt (S1:normalized mock)]^; ΔCt (normalized ChIP) = Ct (ChIP) − [Ct (input) − log2 (input dilution factor)]; input dilution factor = 50.

### Electrophoretic mobility shift assay

The cDNA of the target protein was cloned into the pET-28a vector and fused with a His tag. The vector containing the fusion protein was transformed into *Escherichia coli* strain BL21, and after induction of the expression of the His fusion protein, the *E. coli* were disrupted using ultrasound for subsequent extraction of total protein. The His fusion protein was purified using His agarose, and the purified protein and the synthesized target gene promoter sequence were eluted and subsequently incubated together at room temperature for 20 min. The incubation system consisted of an appropriate concentration of fusion protein, DNA, and binding buffer (10× binding buffer: 100 mM Tris-HCl [pH 7.5], 0.5 M NaCl, 10 mM DTT, 10 mM EDTA, and 50% glycerol). The same procedure was repeated with the His tag serving as the control. After 20 min, the final sample was loaded on a 1.2% agarose gel for electrophoresis, conducted for 45 min in a low-temperature environment (ice bath) a constant voltage of 80 V. The electrophoresed DNA was stained with ethidium bromide for 15 min to visualize its migration in the gel. If the target protein can bind to the detected DNA, the migration distance of the DNA bound to the target protein is shorter than that of the unbound DNA. Thus, the retarded migration of DNA was used as an indicator to determine the binding of the DNA sequence to the target protein.

### Subcellular localization of GFP-labeled proteins

Conidia were obtained from 7-day-old cultures grown on CM plates. The cultures were flooded with sterile water and the resulting conidial suspensions were placed onto glass slides followed by incubation at 25°C. Three replicates of 20 µL droplets were used per slide. Cyan fluorescence was detected at 12 h using a Zeiss laser scanning microscope (ZEISS, Germany), with images being obtained under a 40× objective and processed using ZEN 3.0 software.

### Statistical analysis

The quantitative data presented in this study were derived from at least three independent experiments with triplicate treatments. The data obtained for the different biological treatments were subjected to a one-way or two-way analysis of variance. Statistical analysis was performed using GraphPad Prism 8.0 (GraphPad Software, USA). Histograms represent mean values with standard deviations, and differences were considered significant (*) at *P*-values < 0.05.

## SUPPLEMENTAL MATERIAL

Supplemental figures mbio.00133-24-s0001.pdf Fig. S1 to S7.

Table S1 mbio.00133-24-s0002.docx *B. cinerea* strains used in this study.

Table S2 mbio.00133-24-s0003.docx Primers used in this study.

Table S3 mbio.00133-24-s0004.docx *B. cinerea* genes differentially expressed in Δbcwcl2 compared to WT.

Table S4 mbio.00133-24-s0005.docx *B. cinerea* genes differentially expressed in Δbcwcl2+CA compared to Δbcwcl2.

Table S5 mbio.00133-24-s0006.docx Genes downregulated in Δbcwcl2 and upregulated in Δbcwcl2+CA.

Table S6 mbio.00133-24-s0007.docx Genes of Cluster1 upregulated in Δbcwcl2 and downregulated in Δbcwcl2+CA.

Table S7 mbio.00133-24-s0008.docx FPKM values of differentially expressed genes in Fig. 6A, 8G, and S5A.

Table S8 mbio.00133-24-s0009.docx FPKM values of differentially expressed genes in ROS scavenging-associated genes.

ASM does not own the copyrights to Supplemental Material that may be linked to, or accessed through, an article. The authors have granted ASM a non-exclusive, world-wide license to publish the Supplemental Material files. Please contact the corresponding author directly for reuse.
