# Supplementary material for: The GATA transcription factor BcWCL2 regulates citric acid secretion to maintain redox homeostasis and full virulence in Botrytis cinerea
Source: mBio. 2024 May 30;15(7):e00133-24. doi: 10.1128/mbio.00133-24 (PMC11253612; doi:10.1128/mbio.00133-24)
Supplement: Supplemental figures — Fig. S1 to S7. [file mbio.00133-24-s0001.pdf]

Supplemental Material

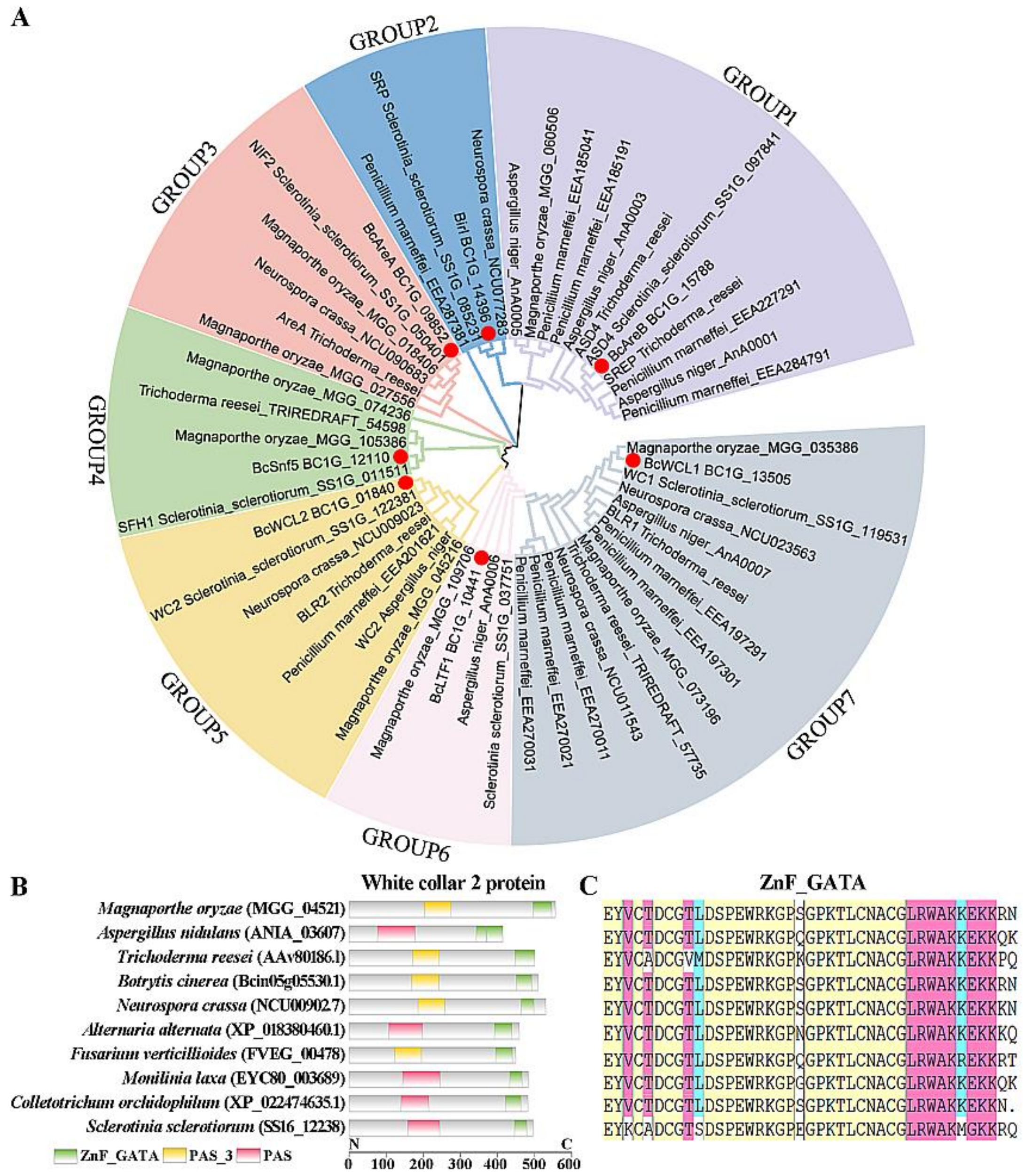

FIG S1 Characterization of White collar 2 (WCL2) proteins. (A) Comparative sequence analysis of GATA transcription factors (TFs). The GATA TFs are classified into seven subgroups in the Neighbor-Joining tree (70). (B) Comparative analysis of the structural domains in White collar 2 proteins from different fungal species revealed that BcWCL2 contains a Zn_GATA domain. (C) Comparative analysis of the Zn_GATA domains in different fungal species using DNAMAN.

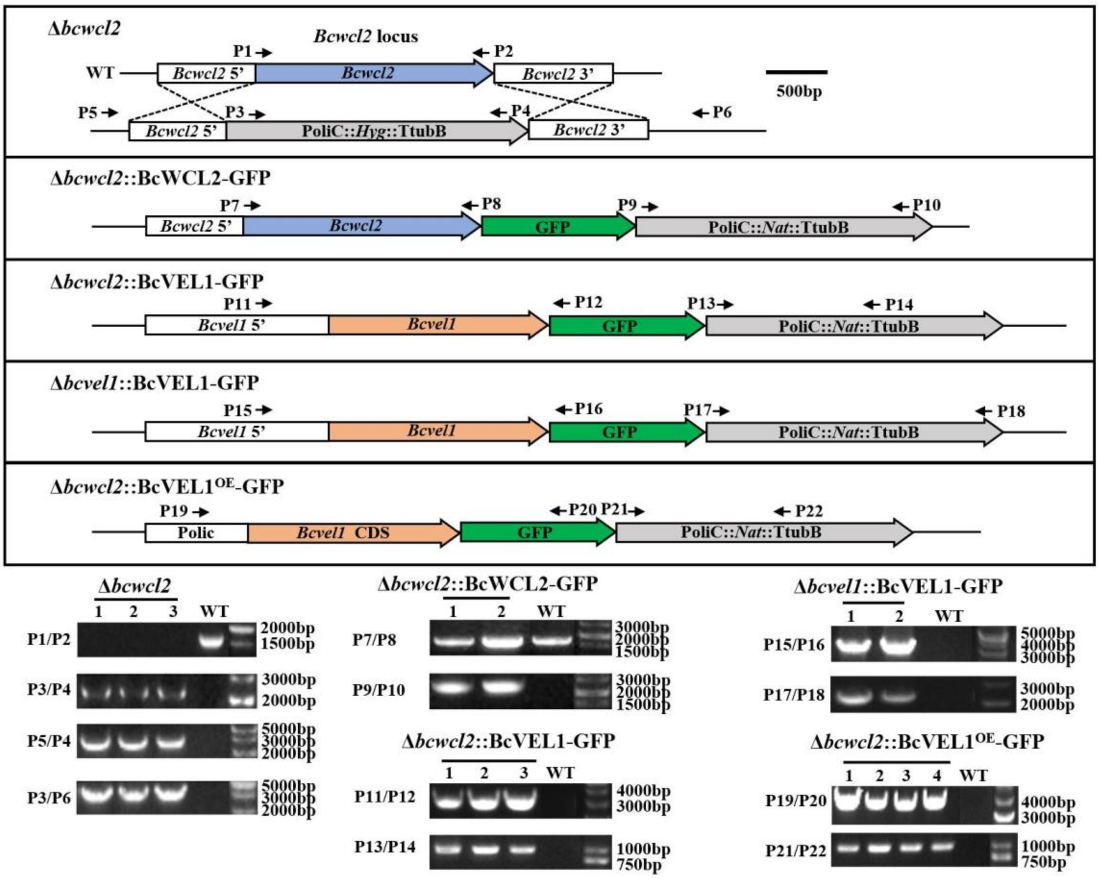

FIG S2 Construction and identification of strains Δ*bcwcl2*, Δ*bcwcl2*::BcWCL2-GFP, Δ*bcwcl2*::BcVEL1-GFP, Δ*bcvel1*::BcVEL1-GFP, Δ*bcwcl2*::BcVEL1^{OE}-GFP. The gene sequence of *Bcwcl2* (*Bcin05g05530.1*) was obtained from EnsemblFungi (http://fungi.ensembl.org/). The strains construction and identification.

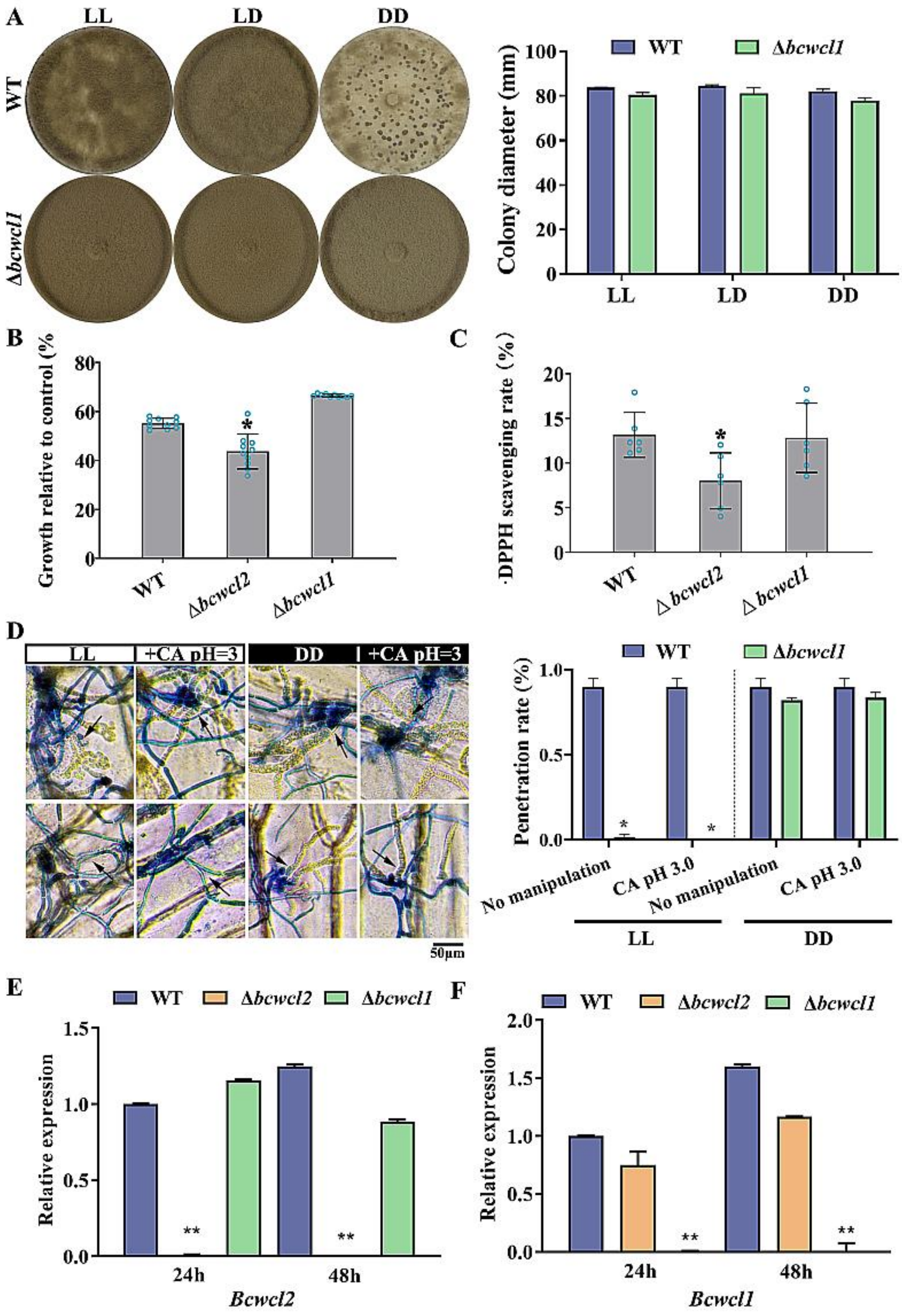

FIG S3 BcWCL1 did not affect virulence, acidification and host penetration under dark conditions. (A) Conidiation and sclerotium formation strains under different light conditions. *B. cinerea* strains were cultured on CM under LL, LD, and DD conditions. (B) Relative growth of WT, Δ*bcwcl2* and Δ*bcwcl1* strains was measured using colonies grown on CM plates supplemented with 8 mM H_2O_2 compared with cultures grown on control media

without treatment after 48 h. (C) •DPPH (2,2-diphenyl-1-picrylhydrazyl) radicals eliminating property of WT, Δ*bcwcl2* and Δ*bcwcl1* strains, data were presented as mean± SD (n = 3). (D) Hyphal penetration of *B. cinerea* mediated by BcWCL1, the regulation of which is associated with CA secretion. The test strains were used to inoculate onion inner epidermis and maintained under LL and DD conditions for 16 h, followed by lactophenol blue staining to visualize the infection hyphae. (E, F) Expression levels of *Bcwc11* and *Bcwc12* as measured by quantitative RT-PCR analyses in WT, Δ*bcwcl2* and Δ*bcwcl1* strains at 24 h and 48 h after inoculation on green bean leaves. * and ** denote values that are significantly different from the WT control values at the $P < 0.05$ and $P < 0.01$ levels, respectively.

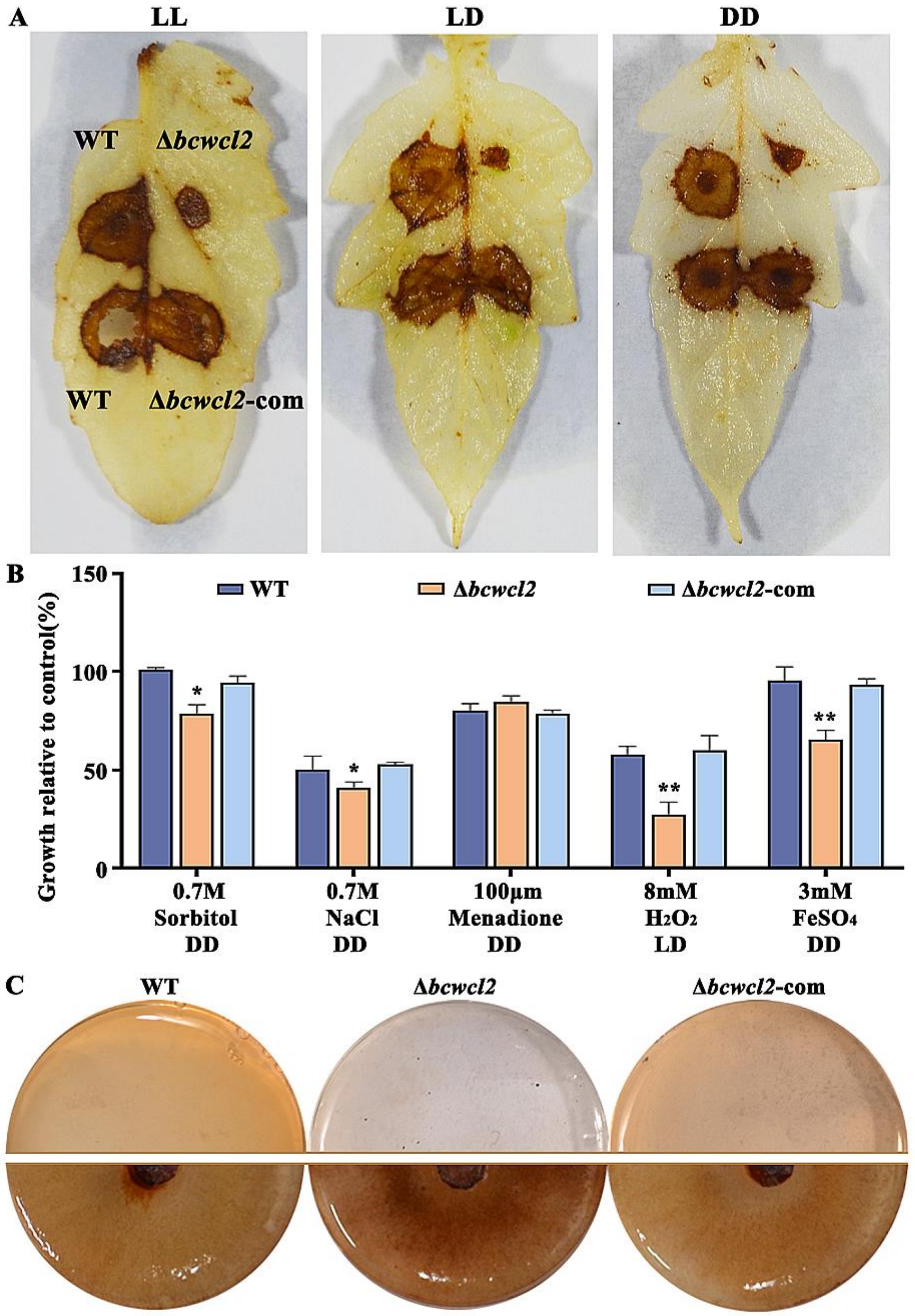

FIG S4 BcWCL2 regulates ROS secretion, osmotic and oxidative stress tolerances. (A) Tomato leaves inoculated with Δ*bcwcl2* demonstrated less ROS accumulation around the inoculation site. (B) Effects of sorbitol, NaCl, menadione, H_2O_2, and $FeSO_4$ addition in the media on mycelium growth of WT, Δ*bcwcl2*, and complementation strains. (C) Deletion of *Bcwcl2* leads to less ROS in culture exudates but more ROS in mycelium. The upper half cultures represent DAB staining of the culture exudates, while the lower halves are DAB staining of the mycelial colonies. The means and standard deviations of at least three

biological replicates are shown. Significant differences from WT are indicated (*, $P < 0.05$, **, $P < 0.01$).

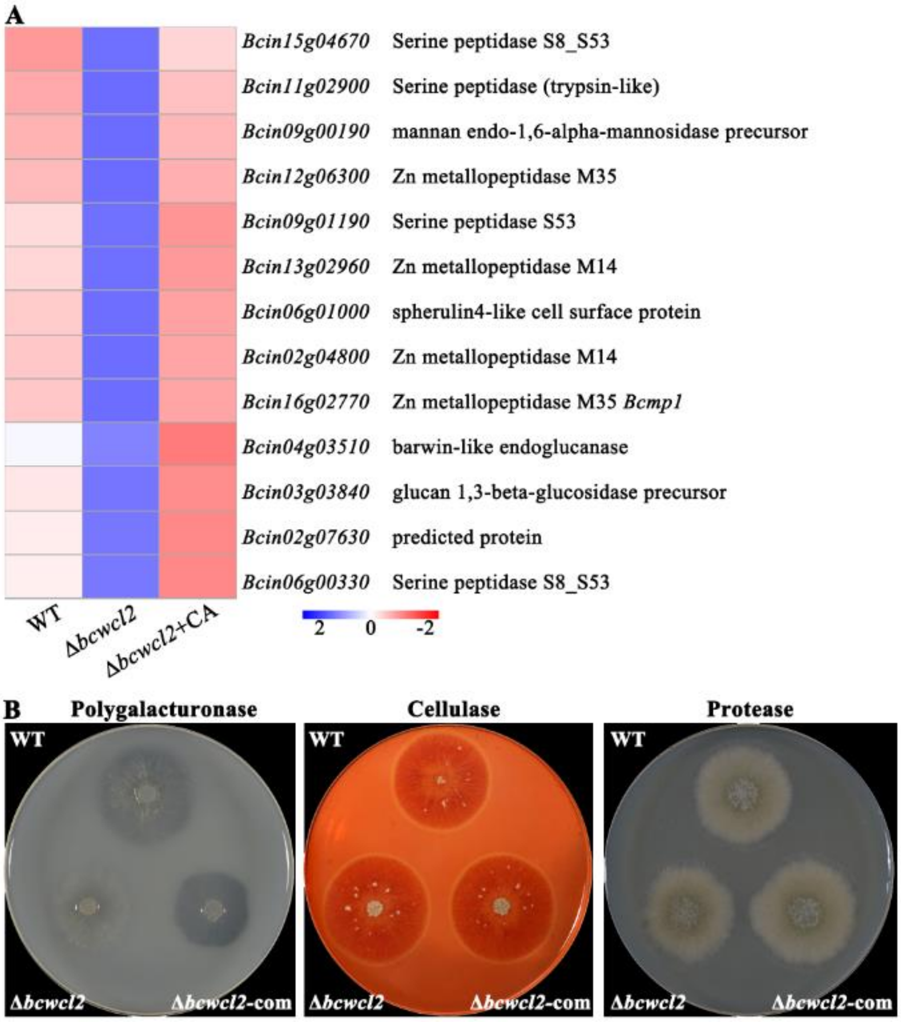

FIG S5 Transcript levels of secreted hydrolytic enzymes are regulated by BcWCL2. (A) Expression profiles derived from the transcriptome data of hydrolytic enzymes in *B. cinerea*. (B) Detection of cell wall degrading enzymes secreted by WT, Δ*bcwcl1*, Δ*bcwcl2*, and Δ*bcwcl2*-com strains. *B. cinerea* strains were cultured on CM under DD (constant dark) conditions at 23°C for 48 h.

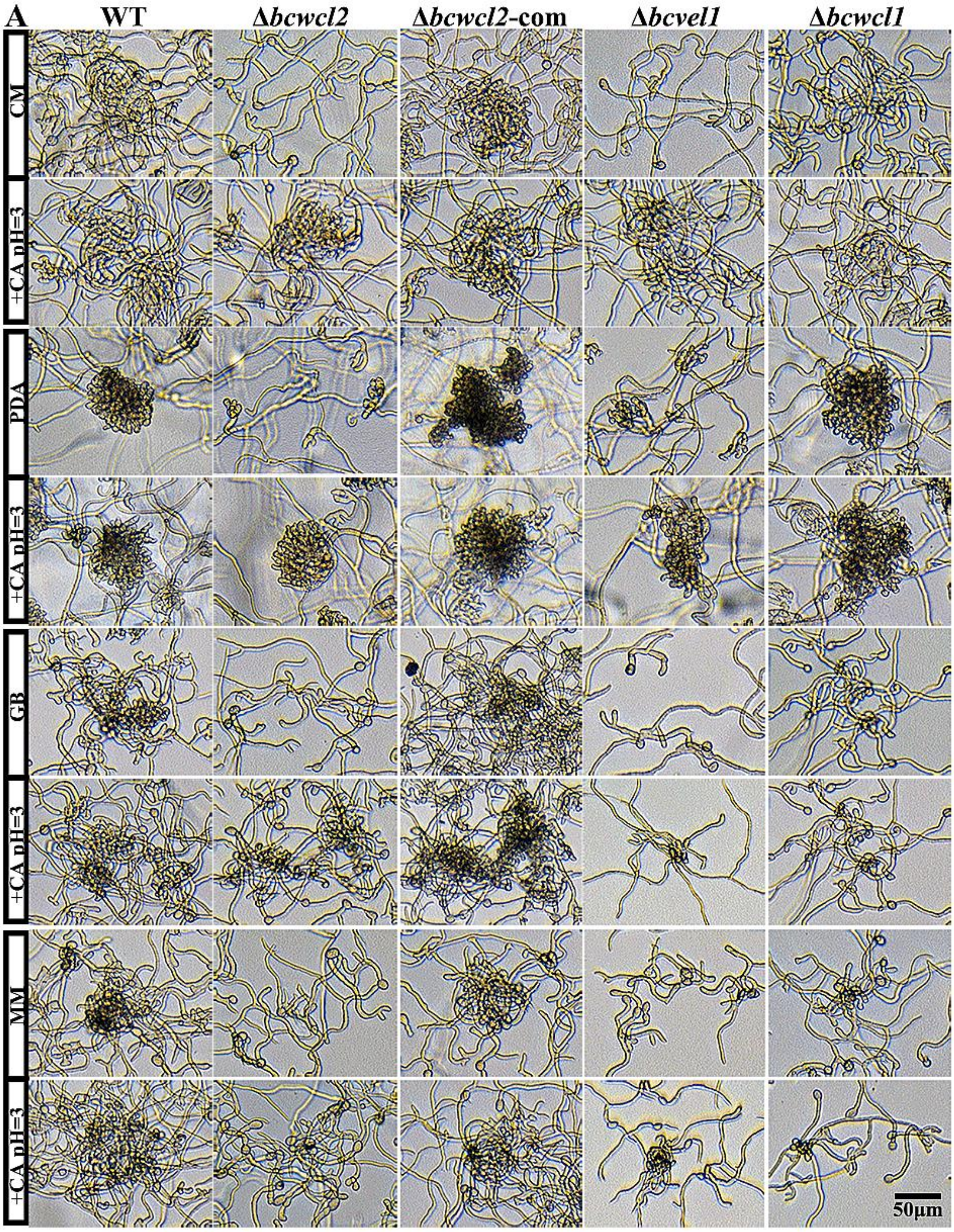

FIG S6 Exogenous addition of CA restores the formation of infection cushions. Infection cushions formed after 16 h by WT, Δ*bcwcl2*, Δ*bcwcl2*-com, Δ*bcvel1*, and Δ*bcwcl1* on CM, PDB, GB, and MM under DD conditions were observed by microscopy. The scale bar represents 50 μm.

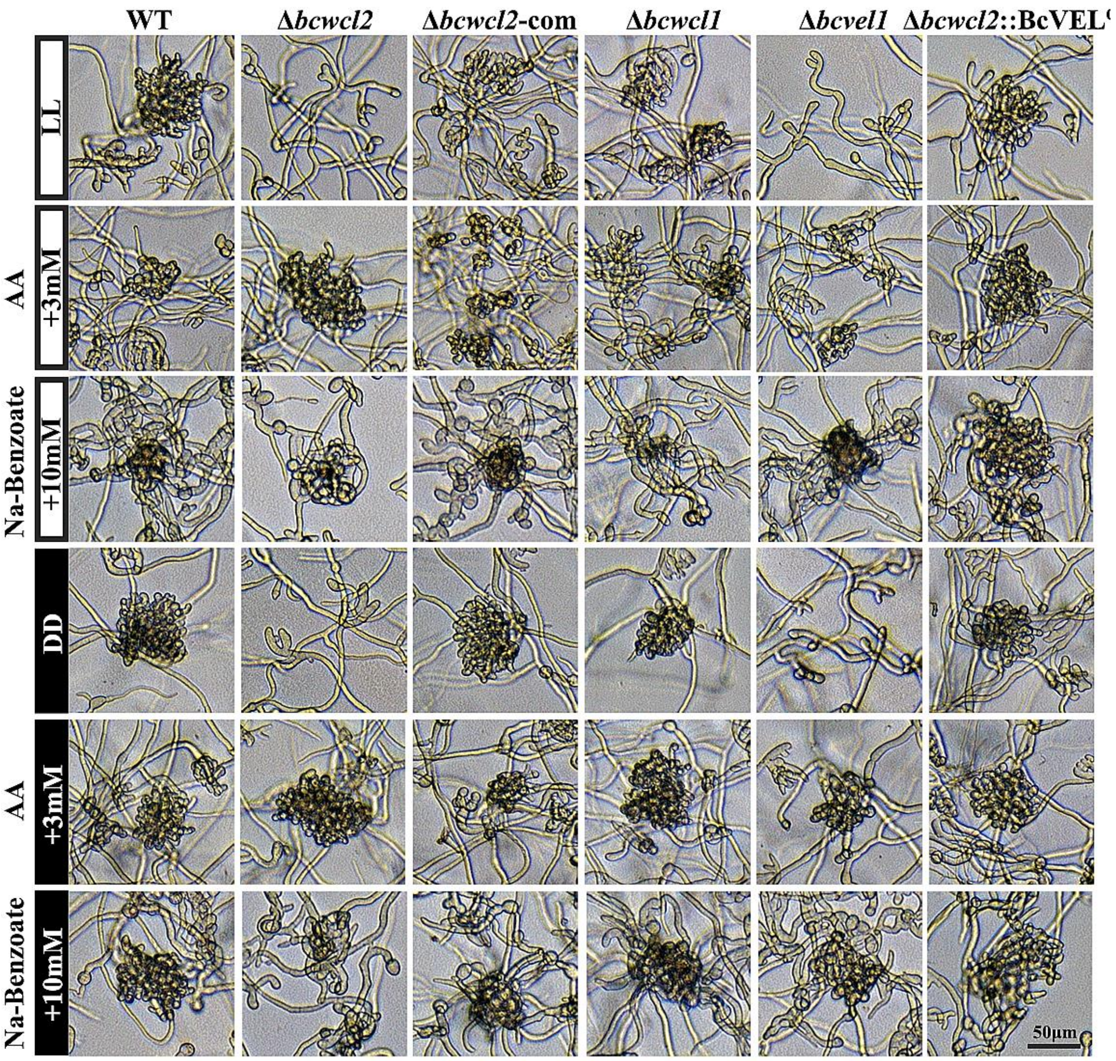

FIG S7 Exogenous addition of 3 mM ascorbic acid (AA), 10 mM sodium benzoate, or overexpression of BcVEL1 restore defects in infection cushion formation in Δ*bcwcl2* mutants. Infection cushions were observed by microscopy in CM liquid medium after 16 h under light and dark conditions for the WT, Δ*bcwcl2*, Δ*bcwcl2*-com, Δ*bcwcl1*, Δ*bcvel1*, and Δ*bcwcl2*::BcVEL1^{OE}. The scale bar represents 50 µm.
