# Supplementary material for: The GATA transcription factor BcWCL2 regulates citric acid secretion to maintain redox homeostasis and full virulence in Botrytis cinerea
Source: mBio. 2024 May 30;15(7):e00133-24. doi: 10.1128/mbio.00133-24 (PMC11253612; doi:10.1128/mbio.00133-24)
Supplement: Table S1 — B. cinerea strains used in this study. [file mbio.00133-24-s0002.docx]

| **Table S1. *B. cinerea* strains were used in this study.** | |  |
| --- | --- | --- |
| **Strain name** | **Genotype description** | **Strain source** |
| WT | B05.10, WT | Matthias Hahn et al., 2018 |
| Δ*bcwcl1* | B05.10, Δ*bcwcl1*::*hyg*; homokaryon | Cai et al., 2023 |
| Δ*bcwcl2* | B05.10, Δ*bcwcl2*::*hyg*; homokaryon | In this study |
| Δ*bcwcl2*::BcWCL2*-*GFP | B05.10, Δ*bcwcl2*::*hyg*::*wcl2-gfp*::*nat1*; homokaryon | In this study |
| Δ*bcvel1* | B05.10, Δ*bcvel1*::*hyg*; homokaryon | Matthias Hahn et al., 2018 |
| Δ*bcvel1*::BcVEL1*-*GFP | B05.10, Δ*bcvel1*:: *hpg*::*vel1-gfp*::*nat1*; homokaryon | In this study |
| Δ*bcwcl2*::BcVEL1-GFP | B05.10, Δ*bcwcl2*::*hpg*::*vel1-gfp*::*nat1*; homokaryon | In this study |
| Δ*bcwcl2*::BcVEL1^OE^ | B05.10, Δ*bcwcl2*::*hyg*::*PoliC*::*vel1-gfp*::*nat1*; homokaryon | In this study |
|  |  |  |
