# Supplementary material for: The GATA transcription factor BcWCL2 regulates citric acid secretion to maintain redox homeostasis and full virulence in Botrytis cinerea
Source: mBio. 2024 May 30;15(7):e00133-24. doi: 10.1128/mbio.00133-24 (PMC11253612; doi:10.1128/mbio.00133-24)
Supplement: Table S2 — Primers used in this study. [file mbio.00133-24-s0003.docx]

**Table S2 Primers used in this study.**

**Primers used for qRT-PCR analysis.**

| **Gene ID** | **Name or predicted function** | **Forward (5'-3')** | **Reverse (5'-3')** |
| --- | --- | --- | --- |
| Bcin05g05530 | *Bcwcl2* | TCCGAACCATCCTGATTTCC | GATTCGCTTTCGTCAACGTG |
| Bcin15g03390 | *Bcvel1* | TGGACTCCAATATGGCTGCG | TCGATCGGCAGATGATTTTGCT |
| Bcin01g09950 | *Bcpyc* | TGTCGATACATTCCACCCCC | GGTAAGAAGTTCACGGGCCA |
| Bcin02g02750 | *Bccit1* | AGGCTCTCCGCAAGGGTTA | CCTGGCACTCTGGGATAGTC |
| Bcin09g00650 | *Bccit3* | TTGGTGGTACCTTGGTTTCA | GGAGCTCGGATGGGGTAAAT |
| Bcin03g05230 | the homologue of *Aspergillus niger* citrate exporter CexA | GCTGGAGTCATTGGGGATATC | AATTCCTCCGAAGACTGGGC |
| Bcin10g04810 | *Bccic1* | GCTATTCTTCGGCGTTGCAG | ACAGGATTCGAAGAAGAGAACA |
| Bcin14g00870 | MFS_1 | ATGGGCTCCTTTGTCGGAAT | AGCTGCACCAAACATACCTCC |
| Bcin02g07440 | MFS_1 | ATGGTGGTTGCATCAACGTG | AACATCATGGGGCCAATACCA |
| Bcin05g00390 | MFS_1 | CTCAGTATCGCGCTCCCAAT | TCTTCCCACCAGCATCAAAGT |
| Bcin16g03420 | an orthologue of *Saccharomyces cerevisiae* SFC1 | GCCACAAATCTTGTTGCTGGT | GGGGAGTTTCGCGCTTCATA |
| Bcin06g03980 | *Bchem15* | CAGCTCACCCAGGTCTTGTC | CAGCAGGATATGGATCACCTCTA |
| Bcin03g06840 | *BcnoxR* | GGCTATCGAATTCCGCGATT | CGATCACGCAGCCCAAAT |
| Bcin12g00520 | *Bcprx2* | ACGGCCCTCAAAAACACCAT | AAGGTTATGAGGAGGGGGCT |
| Bcin02g03060 | *BcppoA80* | ATGGAGGGTGTCAACGCGG | CGATTTCAATATTGGTACTAGTCTC |
| Bcin07g05810 | *Bcccp2* | ATGAGTAAGAAAGGGGATTTTGCCG | ACCAGCATTTGCTGGATCTCCTCCT |
| Bcin09g04400 | *Bccat7* | CTCCACGGGCATTCCCTCCATC | CCACTACTCGCTGCGGCTTTCA |
| Bcactin |  | TCTGTCTTGGGTCTTGAGAG | GGTGCAAGAGCAGTGATTTC |
| Bcvel1-P5 | Chip-qPCR | CCTGTCGAGCCTGTCAAATG | CGGACTACCTACCAATGGCG |
| Bcvel1-P4 | Chip-qPCR | CGCCATTGGTAGGTAGTCCG | ACCACGACCAGAGCATTGAG |
| Bcvel1-P3 | Chip-qPCR | TGCTCTGGTCGTGGTAGACT | GTCTCCATCATTCCCGGTCC |
| Bcvel1-P2 | Chip-qPCR | TGGACTCCAATATGGCTGCG | TCGATCGGCAGATGATTTTGCT |
| Bcvel1-P1 | Chip-qPCR | TTGCAGACACTGATACCGCT | GAGCTACCTCCAAAGTTGCG |

**Primers used for deletion cassettes constructs and genes replacement analysis.**

| **Name** | **Sequence (5' - 3')** | **Used for** |
| --- | --- | --- |
| Bcwcl2-P1 | CCAAAAGTACACTATAGAGC | *Bcwcl2* knockout |
| Bcwcl2-P2 | GGACGCAAATCTTGACCAATAATAGAATCAACTATTACTCAAC | *Bcwcl2* knockout |
| Hyg-P3 | CGCCCACAGCACAATTAGAGTTCGGCTCTAGACTGCAGCTGTGGAGCCG | *Bcwcl2* knockout |
| Hyg-P4 | CGTAAAAGAGTCGGCACGGTGTCCGGGATCCGCTTAGACAACTT | *Bcwcl2* knockout |
| Bcwcl2-P5 | AGTTGTCTAAGCGGATCCCGAGACACCGTGCCGACTCT | *Bcwcl2* knockout |
| Bcwcl2-P6 | TACTCTTATTACTCTGTCTGGTATT | *Bcwcl2* knockout |
| Bcwcl2-P7 | ATGTCAGAGGGGGATACATCTAT | *Bcwcl2* knockout |
| Bcwcl2-P8 | TCAAGAGCCTGGGGAAGGTC | *Bcwcl2* knockout |
| Bcwcl2-P9 | GGTTGTAGGTGGCAATCAAAT | *Bcwcl2* knockout |
| Bcwcl2-P10 | TGCATATGCTTCCGGGAAAT | *Bcwcl2* knockout |
| Bcwcl2-P11 | CTGGCGGCATTATTGGT | *Bcwcl2* complementation |
| Bcwcl2-P12 | AGACGGTGTCGGTGGTG | *Bcwcl2* complementation |
| Bcvel1^OE^-jsF | CTTCTTCCATCCTCAGTCCAT | Δ*bcwcl2*::BcVEL1^OE^ |
| Bcvel1^OE^-jsR | CCATGCGCCAATCAAAAGACG | Δ*bcwcl2*::BcVEL1^OE^ |
| Bcvel1^OE^-GFP-F | CTTAAAGAATTCTTTCTCGAGGCTGCGTCCATAGGTCCCA | Δ*bcwcl2*::BcVEL1^OE^ |
| Bcvel1^OE^-GFP-R | AACCATCCCGGGTTTCTCGAGGTTAAGACCGGGGGCGGG | Δ*bcwcl2*::BcVEL1^OE^ |
| Bcwcl2^OE^-GFP-F | CTTAAAGAATTCTTTCTCGAGATGTCAGAGGGGGATACATCTATGA | Δ*bcwcl2*::BcWCL2^OE^*-*GFP |
| Bcwcl2^OE^-GFP-R | AACCATCCCGGGTTTCTCGAGTCAAGAGCCTGGGGAAGGTC | Δ*bcwcl2*::BcWCL2^OE^*-*GFP |
| Bcwcl2-GFP-F | GATTACGCCGAATTCGAGCTCCATGATTACGCCGAATTCGAG | Δ*bcwcl2*::BcWCL2*-*GFP |
| Bcwcl2-GFP-R | AACCATCCCGGGTTTCTCGAGAGAGCCTGGGGAAGGTCCC | Δ*bcwcl2*::BcWCL2*-*GFP |
| Bcvel1-GFP-F | GATTACGCCGAATTCGAGCTCTGCAAGAAAGGCTGGATGGA | Δ*bcvel1*::BcVEL1*-*GFP |
| Bcvel1-GFP-F | AACCATCCCGGGTTTCTCGAGGTTAAGACCGGGGGCGGG | Δ*bcvel1*::BcVEL1*-*GFP |
