# Supplementary material for: The GATA transcription factor BcWCL2 regulates citric acid secretion to maintain redox homeostasis and full virulence in Botrytis cinerea
Source: mBio. 2024 May 30;15(7):e00133-24. doi: 10.1128/mbio.00133-24 (PMC11253612; doi:10.1128/mbio.00133-24)
Supplement: Table S3 — B. cinerea genes differentially expressed in Δbcwcl2 compared to WT. [file mbio.00133-24-s0004.docx]

**Table S3: *B. cinerea* genes differentially expressed in Δ*bcwcl2* compared to WT.**

| **Feature ID (fungi.ensembl.org)** | **Putative Function** | **WT mean FPKM 48h in planta** | **Δ*bcwcl2* mean FPKM 48h in planta** | **log2FoldChange:Δ*bcwcl2* vs WT** | **padj adjusted for multiple testing with the Benjamini-Hochberg procedure** | |
| --- | --- | --- | --- | --- | --- | --- |
| novel.957 | - | 0 | 16.00172184 | 6.509466954 | | 7.51007E-05 |
| Bcin09g00730 | Q870L2.1 RecName: Full=Siderophore iron transporter mirB; AltName: Full=Major facilitator iron-regulated transporter B; AltName: Full=Triacetylfusarinine C permease | 0 | 8.979213887 | 5.673772885 | | 0.002217932 |
| Bcin10g04780 | - | 0 | 7.835838606 | 5.479499411 | | 0.003834668 |
| Bcin15g01540 | - | 0.311603149 | 14.05865284 | 5.359873422 | | 0.000789344 |
| Bcin16g04060 | Q4X084.1 RecName: Full=Probable endo-1,3(4)-beta-glucanase AFUA_2G14360; AltName: Full=Mixed-linked glucanase AFUA_2G14360; Flags: Precursor >B0XTU6.1 RecName: Full=Probable endo-1,3(4)-beta-glucanase AFUB_029980; AltName: Full=Mixed-linked glucanase | 1.296427364 | 46.07382854 | 5.173124528 | | 1.61351E-08 |
| Bcin04g06380 | - | 3.504582438 | 114.1745802 | 5.026362706 | | 3.65394E-20 |
| novel.1190 | - | 0.311603149 | 7.771932706 | 4.506899342 | | 0.02975616 |
| novel.464 | - | 0.311603149 | 7.513533494 | 4.456747895 | | 0.018908296 |
| Bcin02g00290 | E9RAH5.1 RecName: Full=Thioredoxin reductase gliT; AltName: Full=Gliotoxin biosynthesis protein T | 0.940966312 | 20.82201218 | 4.451926646 | | 0.000160742 |
| Bcin03g05400 | - | 0.317760013 | 7.477710785 | 4.450262411 | | 0.043020589 |
| Bcin06g07050 | Q7Z9M7.3 RecName: Full=Endoglucanase-7; AltName: Full=Cellulase-61B; Short=Cel61B; AltName: Full=Endo-1,4-beta-glucanase VII; Short=EGVII; AltName: Full=Endoglucanase VII; AltName: Full=Endoglucanase-61B; Flags: Precursor | 6.74141963 | 120.1710088 | 4.164364472 | | 4.54199E-21 |
| Bcin08g04620 | - | 0.623206299 | 11.31744958 | 4.160082081 | | 0.008497878 |
| Bcin05g03170 | O59942.2 RecName: Full=Amino-acid permease 2 | 3.179905707 | 54.44899681 | 4.096535449 | | 3.14758E-09 |
| Bcin02g02040 | O14405.1 RecName: Full=Endoglucanase-4; AltName: Full=Cellulase IV; AltName: Full=Cellulase-61A; Short=Cel61A; AltName: Full=Endo-1,4-beta-glucanase IV; Short=EGIV; AltName: Full=Endoglucanase IV; AltName: Full=Endoglucanase-61A; Flags: Precursor | 51.8501379 | 861.0044213 | 4.05252932 | | 1.9733E-149 |
| Bcin15g04000 | - | 0.934809448 | 15.64711976 | 4.045308998 | | 0.002436008 |
| Bcin12g05930 | - | 1.281808063 | 20.50791334 | 4.006989175 | | 0.000190245 |
| Bcin15g04790 | P25358.1 RecName: Full=Elongation of fatty acids protein 2; AltName: Full=3-keto acyl-CoA synthase ELO2; AltName: Full=Fenpropimorph resistance protein 1; AltName: Full=Glucan synthesis protein 1; AltName: Full=Very-long-chain 3-oxoacyl-CoA synthase | 25.76311051 | 399.3035155 | 3.953026788 | | 3.53679E-70 |
| Bcin09g01770 | - | 0.940966312 | 14.45341414 | 3.92503568 | | 0.002563186 |
| Bcin11g02690 | A0A0D2YG06.1 RecName: Full=Hydrolase FUB4; AltName: Full=Fusaric acid biosynthesis protein 4 | 16.52425454 | 239.7179731 | 3.856534166 | | 1.73334E-38 |
| novel.463 | - | 1.246412597 | 16.49430756 | 3.705876968 | | 0.002287582 |
| novel.966 | - | 0.623206299 | 8.200207136 | 3.69798118 | | 0.03466925 |
| Bcin11g06510 | - | 5.447297838 | 69.81784044 | 3.686112459 | | 5.68911E-12 |
| Bcin11g02630 | Q0C8A0.1 RecName: Full=Dioxygenase trt7; AltName: Full=Terretonin synthesis protein 7 | 84.69320422 | 1029.612175 | 3.603666547 | | 8.4015E-148 |
| Bcin11g06520 | Q12612.1 RecName: Full=Trichodiene oxygenase; AltName: Full=Cytochrome P450 58 | 5.487304449 | 61.65657637 | 3.493881145 | | 0.003673363 |
| novel.863 | - | 1.296427364 | 14.19860132 | 3.466864222 | | 0.021020865 |
| Bcin14g00080 | - | 4.108558291 | 45.51069155 | 3.463064336 | | 1.26104E-07 |
| Bcin06g02830 | P27121.1 RecName: Full=Ornithine decarboxylase; Short=ODC | 6.97531513 | 76.61580705 | 3.452896677 | | 2.15935E-12 |
| Bcin06g00510 | - | 26.39556511 | 265.4363182 | 3.328952268 | | 3.09019E-38 |
| novel.659 | - | 0.940966312 | 9.528714667 | 3.328178059 | | 0.026688195 |
| novel.1223 | - | 1.258726326 | 12.65843853 | 3.319767106 | | 0.00828176 |
| Bcin14g03990 | W7MMJ0.1 RecName: Full=Glutathione S-transferase-like protein FUS3; AltName: Full=Fusarin biosynthesis protein 3 | 0.934809448 | 9.311629467 | 3.297019862 | | 0.035852401 |
| novel.348 | - | 0.940966312 | 9.204099972 | 3.278294547 | | 0.034948244 |
| Bcin11g02680 | Q8BGA8.1 RecName: Full=Acyl-coenzyme A synthetase ACSM5, mitochondrial; Flags: Precursor | 31.32965501 | 303.0743543 | 3.269869774 | | 2.35335E-32 |
| novel.213 | - | 2.551302398 | 23.02382513 | 3.174527818 | | 0.000518262 |
| Bcin15g04760 | - | 20.40813962 | 181.4807226 | 3.153932861 | | 1.39799E-27 |
| Bcin03g00080 | - | 18.16920023 | 160.5395103 | 3.144430502 | | 1.17165E-23 |
| Bcin11g02650 | Q4WLW5.1 RecName: Full=Nonribosomal peptide synthetase 12 | 10.46373255 | 90.52492763 | 3.110154772 | | 1.2892E-12 |
| Bcin03g03480 | Q96VB6.1 RecName: Full=Endo-1,4-beta-xylanase F3; Short=Xylanase F3; AltName: Full=1,4-beta-D-xylan xylanohydrolase F3; Flags: Precursor | 122.3664536 | 1056.851776 | 3.109320048 | | 1.0786E-118 |
| Bcin13g00370 | - | 1.304889801 | 10.94724572 | 3.087194309 | | 0.030372044 |
| Bcin08g07050 | Q1E3R8.1 RecName: Full=Endochitinase 1; AltName: Full=Complement-fixation antigen; Short=CF-AG; Short=CF-antigen; Flags: Precursor | 42.22891386 | 350.4251045 | 3.052252447 | | 1.33707E-10 |
| Bcin15g04780 | Q93VK5.1 RecName: Full=Protein LUTEIN DEFICIENT 5, chloroplastic; AltName: Full=Cytochrome P450 97A3; Flags: Precursor | 33.78788392 | 276.0846615 | 3.031404449 | | 4.01425E-39 |
| Bcin03g05200 | D4AZ24.1 RecName: Full=Probable endo-1,3(4)-beta-glucanase ARB_01444; Short=Endo-1,3-beta-glucanase; Short=Endo-1,4-beta-glucanase; AltName: Full=Laminarinase; Flags: Precursor | 42.82977226 | 348.2507268 | 3.020382294 | | 6.96251E-44 |
| Bcin01g03520 | - | 11.78787881 | 93.67331224 | 2.990972927 | | 4.11281E-11 |
| novel.631 | - | 1.605724941 | 12.65572356 | 2.985314326 | | 0.017246 |
| Bcin07g00990 | Q9UQY0.2 RecName: Full=Demethylsterigmatocystin 6-O-methyltransferase; AltName: Full=Aflatoxin biosynthesis protein O; AltName: Full=Methyltransferase B; AltName: Full=O-methyltransferase I; Short=mt-I | 1.246412597 | 9.931276855 | 2.978018234 | | 0.043111757 |
| Bcin15g03990 | - | 6.076661001 | 47.63883215 | 2.974167417 | | 4.70878E-07 |
| Bcin07g06780 | Q0CCX6.1 RecName: Full=Dihydrogeodin oxidase; Short=DHGO; AltName: Full=Geodin synthesis protein J; Flags: Precursor | 29.63546773 | 231.6569044 | 2.966804685 | | 5.55866E-31 |
| Bcin15g01700 | E9R876.1 RecName: Full=MFS gliotoxin efflux transporter gliA; AltName: Full=Gliotoxin biosynthesis protein A | 10.72686665 | 83.98910798 | 2.963259419 | | 8.0997E-10 |
| Bcin15g04770 | - | 51.28619467 | 397.0765578 | 2.952724933 | | 1.01204E-45 |
| Bcin12g06760 | - | 82.92512125 | 640.6684315 | 2.947950074 | | 4.49604E-84 |
| novel.1462 | - | 1.564172611 | 12.03723096 | 2.929783085 | | 0.022588959 |
| Bcin01g07210 | D4ATR3.1 RecName: Full=Uncharacterized secreted glycosidase ARB_07629; Flags: Precursor | 20.18886342 | 151.5633554 | 2.912918936 | | 1.51308E-17 |
| Bcin11g02640 | A7HU16.1 RecName: Full=Baeyer-Villiger monooxygenase; Short=BVMO | 6.051273691 | 45.17671579 | 2.900638847 | | 7.28031E-07 |
| Bcin01g10150 | Q5AK66.1 RecName: Full=Phosphatidylserine decarboxylase proenzyme 2; Contains: RecName: Full=Phosphatidylserine decarboxylase 2 beta chain; Contains: RecName: Full=Phosphatidylserine decarboxylase 2 alpha chain | 24.83445793 | 182.8062318 | 2.880330088 | | 3.46148E-23 |
| Bcin01g07200 | - | 6.149757505 | 44.61744856 | 2.869355562 | | 1.98991E-05 |
| Bcin11g02620 | P53389.1 RecName: Full=Protein HOL1 | 8.282510503 | 59.78989396 | 2.852618425 | | 4.15733E-08 |
| Bcin11g02660 | - | 47.988562 | 342.1308835 | 2.836806038 | | 7.62651E-37 |
| Bcin15g04750 | P24458.1 RecName: Full=Cytochrome P450 52A3-B; Short=CYP52A3-B; AltName: Full=Alkane-inducible P450-ALK1-B; AltName: Full=CYPLIIA3 | 19.11401783 | 134.4035287 | 2.814531296 | | 1.98665E-16 |
| Bcin08g03850 | D7PI20.2 RecName: Full=Cytochrome P450 monooxygenase gsfF; AltName: Full=Griseofulvin synthesis protein F; Flags: Precursor | 1686.44093 | 11559.55337 | 2.777068213 | | 0 |
| Bcin03g00060 | - | 7.975518498 | 54.51948744 | 2.77435621 | | 1.9996E-07 |
| Bcin08g05370 | - | 12.0456159 | 82.36244812 | 2.769952627 | | 5.21128E-10 |
| novel.220 | PF11807:Mycotoxin biosynthesis protein UstYa | 3.169897551 | 21.63412667 | 2.766902423 | | 0.001754702 |
| Bcin03g00920 | - | 4.424772585 | 30.22013943 | 2.765671687 | | 0.000143034 |
| Bcin07g00890 | Q12713.1 RecName: Full=Endochitinase 33; AltName: Full=33 kDa endochitinase; AltName: Full=Chitinase 33; Flags: Precursor | 229.6806036 | 1535.834445 | 2.743001431 | | 7.7556E-111 |
| Bcin11g02670 | E9QUT3.1 RecName: Full=Hydroxynaphthalene reductase arp2; AltName: Full=Conidial pigment biosynthesis oxidase arp2 | 291.5374371 | 1938.813134 | 2.733143305 | | 6.0546E-217 |
| Bcin14g05510 | Q5B9Z8.2 RecName: Full=Probable alpha-L-arabinofuranosidase axhA-1; AltName: Full=Arabinoxylan arabinofuranohydrolase axhA-1; Flags: Precursor | 239.1487173 | 1589.137492 | 2.732277527 | | 1.7162E-159 |
| Bcin09g00960 | B8NM75.1 RecName: Full=Efflux pump ustT; AltName: Full=Ustiloxin B biosynthesis protein T | 9.771281036 | 64.70956889 | 2.720670534 | | 1.36402E-07 |
| Bcin15g04810 | B6HJU0.1 RecName: Full=Efflux pump roqT; AltName: Full=Roquefortine/meleagrin synthesis protein T | 6.699867299 | 43.8536382 | 2.711973834 | | 2.83394E-06 |
| Bcin04g03810 | - | 4.725607725 | 30.58138761 | 2.686055598 | | 0.000332203 |
| Bcin13g02000 | A0A097ZPE4.1 RecName: Full=Cytochrome P450 monooxygenase andK; AltName: Full=Anditomin synthesis protein K; Flags: Precursor | 40.18691608 | 258.3354574 | 2.685665404 | | 7.84379E-33 |
| Bcin07g01850 | - | 2.205849502 | 14.17086287 | 2.67453957 | | 0.020513342 |
| Bcin01g05680 | Q0D076.1 RecName: Full=Probable mannosyl-oligosaccharide alpha-1,2-mannosidase 1B; AltName: Full=Class I alpha-mannosidase 1B; AltName: Full=Man(9)-alpha-mannosidase 1B; Flags: Precursor | 272.605833 | 1709.630177 | 2.649692884 | | 2.7311E-166 |
| Bcin15g04800 | P38256.1 RecName: Full=Uncharacterized protein YBR096W | 11.24775116 | 68.87432776 | 2.620453466 | | 5.02848E-08 |
| novel.396 | - | 1.900403217 | 11.56377276 | 2.603008838 | | 0.048249798 |
| Bcin03g09020 | Q7RVX9.2 RecName: Full=Repressible high-affinity phosphate permease | 2.557459262 | 15.45222106 | 2.59729854 | | 0.015278765 |
| novel.592 | - | 1.894246353 | 11.28861698 | 2.569419844 | | 0.041677684 |
| Bcin01g07330 | A2QBB6.1 RecName: Full=Probable endopolygalacturonase E; Short=PGE; AltName: Full=Pectinase 4; AltName: Full=Pectinase E; AltName: Full=Polygalacturonase E; AltName: Full=Polygalacturonase IV; Short=PG-IV; Flags: Precursor | 5726.352134 | 33324.68257 | 2.540804701 | | 0 |
| Bcin03g00900 | O13752.1 RecName: Full=Uncharacterized TLC domain-containing protein C17A2.02c | 63.32484182 | 367.3260061 | 2.535573548 | | 1.99114E-37 |
| Bcin07g04020 | - | 15.21781902 | 85.47310511 | 2.487203585 | | 1.86324E-09 |
| Bcin10g01150 | - | 10.15443497 | 56.91619909 | 2.484199831 | | 7.71974E-07 |
| Bcin05g03820 | - | 3.840813044 | 21.24173632 | 2.470493407 | | 0.008268154 |
| Bcin06g04260 | - | 2.858294402 | 15.68334786 | 2.45522718 | | 0.012905375 |
| novel.1221 | - | 12.13409156 | 66.31126664 | 2.452698999 | | 4.2452E-08 |
| Bcin16g02560 | Q2U4L7.2 RecName: Full=Glutaminase A; Flags: Precursor | 147.9972959 | 796.7889983 | 2.428217192 | | 3.62964E-83 |
| Bcin02g07230 | A1C7B5.1 RecName: Full=Probable endo-1,3(4)-beta-glucanase ACLA_073210; AltName: Full=Mixed-linked glucanase ACLA_073210; Flags: Precursor | 3.195284861 | 17.03173231 | 2.417782652 | | 0.013472506 |
| novel.1156 | - | 8.310203385 | 43.92216398 | 2.402566385 | | 0.000423277 |
| Bcin02g00430 | B6GX22.1 RecName: Full=Mutanase Pc12g07500; AltName: Full=Endo-1,3-alpha-glucanase Pc12g07500; AltName: Full=Glucan endo-1,3-alpha-glucosidas Pc12g07500; Flags: Precursor | 24.46974857 | 128.592921 | 2.392286613 | | 5.42054E-13 |
| Bcin08g03830 | Q9LBG2.1 RecName: Full=Levodione reductase; AltName: Full=(6R)-2,2,6-trimethyl-1,4-cyclohexanedione reductase | 2261.710162 | 11797.97811 | 2.382721976 | | 0 |
| novel.1251 | - | 4.194728377 | 21.63921259 | 2.374798852 | | 0.006944523 |
| Bcin15g00290 | - | 4.143953757 | 21.17314989 | 2.354462877 | | 0.00668809 |
| Bcin06g03730 | - | 94.04672233 | 479.2708743 | 2.351431196 | | 4.53075E-32 |
| Bcin08g00410 | - | 7.316156881 | 37.08247797 | 2.34029423 | | 0.000102296 |
| Bcin10g04860 | - | 5.722745668 | 28.14105236 | 2.296453747 | | 0.00147932 |
| Bcin07g02730 | P49426.1 RecName: Full=Glucan 1,3-beta-glucosidase; AltName: Full=1,3-beta-D-glucanohydrolase; AltName: Full=Exo-beta 1,3 glucanase; Flags: Precursor | 662.7547485 | 3248.942952 | 2.293679451 | | 3.2762E-266 |
| Bcin03g00640 | P04842.2 RecName: Full=Alcohol oxidase 1; Short=AO 1; Short=AOX 1; AltName: Full=Methanol oxidase 1; Short=MOX 1 >F2QY27.1 RecName: Full=Alcohol oxidase 1; Short=AO 1; Short=AOX 1; AltName: Full=Methanol oxidase 1; Short=MOX 1 | 1309.517693 | 6392.919782 | 2.287271768 | | 0 |
| Bcin14g03270 | - | 72.34905875 | 352.2962447 | 2.284809307 | | 7.29565E-27 |
| Bcin03g00570 | - | 274.8508778 | 1337.344745 | 2.284039079 | | 2.92509E-68 |
| Bcin06g03740 | - | 137.8989932 | 671.8674403 | 2.283104387 | | 7.62683E-61 |
| Bcin12g05940 | Q9PFB0.1 RecName: Full=Beta-lactamase hydrolase-like protein; Short=BLH | 16.4626859 | 80.23621171 | 2.281151236 | | 2.55037E-08 |
| Bcin04g00460 | - | 6.946076528 | 33.87515136 | 2.279707277 | | 0.001721565 |
| Bcin01g04860 | - | 47.10296814 | 228.8260223 | 2.279522504 | | 7.6028E-20 |
| Bcin16g02580 | - | 3.496120001 | 16.85399556 | 2.267000809 | | 0.01447928 |
| Bcin06g04060 | B8N8R1.1 RecName: Full=O-methyltransferase afvC; AltName: Full=Aflavarin synthesis protein C | 4.449400042 | 20.64855058 | 2.21223844 | | 0.012831275 |
| Bcin03g09316 | - | 165.4363569 | 754.0019639 | 2.189052442 | | 2.50521E-53 |
| Bcin10g05210 | - | 5.432678537 | 24.38001047 | 2.167754122 | | 0.004076207 |
| Bcin03g09318 | - | 638.8675057 | 2858.970294 | 2.162777154 | | 3.2091E-183 |
| Bcin07g05690 | - | 4.123177592 | 18.47753578 | 2.16161247 | | 0.014091796 |
| Bcin02g07110 | - | 3.230680327 | 14.33652359 | 2.159338572 | | 0.042910572 |
| Bcin16g03240 | O34767.1 RecName: Full=Oxalate decarboxylase OxdD | 37.49404513 | 160.7857115 | 2.103545685 | | 7.7121E-12 |
| Bcin02g00360 | P20960.2 RecName: Full=Nitrilase, arylacetone-specific; AltName: Full=Arylacetonitrilase | 10.26447266 | 43.95960752 | 2.102234775 | | 0.000234953 |
| Bcin16g00680 | - | 8.956491421 | 38.13670115 | 2.093460967 | | 0.002374113 |
| Bcin01g09980 | A4FV08.1 RecName: Full=Glucosamine-6-phosphate isomerase 1; AltName: Full=Glucosamine-6-phosphate deaminase 1; Short=GNPDA 1; Short=GlcN6P deaminase 1; AltName: Full=Oscillin | 267.0946736 | 1131.588825 | 2.082897593 | | 2.07026E-91 |
| Bcin09g04620 | - | 70.99725894 | 299.6633567 | 2.079635722 | | 5.89773E-22 |
| Bcin10g04190 | D4AUF1.1 RecName: Full=WSC domain-containing protein ARB_07867; Flags: Precursor | 119.5820543 | 503.1959039 | 2.071071618 | | 4.48306E-32 |
| Bcin12g02120 | P78581.1 RecName: Full=Tannase; Contains: RecName: Full=Tannase 33 kDa subunit; Contains: RecName: Full=Tannase 30 kDa subunit; Flags: Precursor | 23.79268953 | 99.55319505 | 2.069187147 | | 3.48275E-06 |
| Bcin08g03760 | P11838.2 RecName: Full=Endothiapepsin; AltName: Full=Aspartate protease; Flags: Precursor | 63.42332564 | 263.2741378 | 2.053586365 | | 1.51839E-20 |
| Bcin03g00910 | Q12442.2 RecName: Full=ADIPOR-like receptor IZH2; AltName: Full=Phosphate metabolism protein 36 | 35.54747844 | 146.326928 | 2.044763263 | | 5.76636E-11 |
| Bcin08g03840 | O13317.1 RecName: Full=Isotrichodermin C-15 hydroxylase; AltName: Full=Cytochrome P450 65A1 | 1443.665108 | 5950.97799 | 2.043029816 | | 0 |
| Bcin15g05660 | P47734.2 RecName: Full=S-(hydroxymethyl)glutathione dehydrogenase; AltName: Full=Glutathione-dependent formaldehyde dehydrogenase; Short=FALDH; Short=FDH | 87.93541241 | 361.4020958 | 2.038872214 | | 6.14867E-25 |
| Bcin05g07680 | - | 224.4611091 | 919.1420431 | 2.034244546 | | 3.65205E-58 |
| novel.507 | - | 5.449603411 | 22.25377406 | 2.033621909 | | 0.017876659 |
| Bcin01g03360 | - | 22.42701695 | 91.3759859 | 2.028675588 | | 1.91275E-05 |
| Bcin03g00070 | - | 13.01658066 | 52.60484565 | 2.012172279 | | 3.97523E-05 |
| Bcin02g00050 | Q8E372.1 RecName: Full=Unsaturated chondroitin disaccharide hydrolase; AltName: Full=Unsaturated glucuronyl hydrolase; Short=SagUGL | 6.707569883 | 26.78371223 | 1.998779558 | | 0.004761189 |
| Bcin15g04010 | - | 51.85252151 | 206.6841749 | 1.994821827 | | 0.01941259 |
| Bcin07g05420 | - | 26.4101584 | 104.1313767 | 1.978159977 | | 1.89171E-08 |
| Bcin08g03820 | B3GQR3.1 RecName: Full=Probable pectin lyase E; Short=PLE; Flags: Precursor [Aspergillus niger] | 1549.69052 | 6085.938784 | 1.973350034 | | 0 |
| Bcin12g05920 | - | 6.375190569 | 25.0253701 | 1.973309115 | | 0.005779713 |
| novel.642 | - | 8.286361794 | 32.3716827 | 1.965221019 | | 0.002603013 |
| Bcin09g02260 | D4AK17.1 RecName: Full=PI-PLC X domain-containing protein 1; Flags: Precursor | 171.3260971 | 664.9869078 | 1.955852653 | | 4.46179E-30 |
| Bcin09g05340 | S0E028.1 RecName: Full=Bikaverin cluster transcription factor bik5; AltName: Full=Bikaverin biosynthesis protein 5 | 7.906273286 | 30.54787448 | 1.945701744 | | 0.004287955 |
| novel.327 | - | 78.52497612 | 301.9072944 | 1.941647061 | | 9.7371E-23 |
| Bcin06g03780 | - | 234.967835 | 902.9075935 | 1.941433023 | | 3.29805E-62 |
| novel.742 | - | 4.129334456 | 15.76984564 | 1.931504517 | | 0.044725786 |
| Bcin06g00500 | - | 77.9917912 | 296.3699377 | 1.925510958 | | 9.48377E-24 |
| Bcin12g06510 | A2QBQ3.1 RecName: Full=Probable endo-1,3(4)-beta-glucanase An02g00850; AltName: Full=Mixed-linked glucanase An02g00850; Flags: Precursor | 4.46171377 | 16.85399556 | 1.916937123 | | 0.045191306 |
| novel.1577 | - | 32.0575153 | 119.6923434 | 1.902949209 | | 2.07552E-07 |
| Bcin07g00690 | - | 7.044560342 | 26.21520596 | 1.899696413 | | 0.01866317 |
| Bcin01g04930 | - | 15.19780271 | 56.71431028 | 1.8960749 | | 0.000108812 |
| Bcin15g04330 | Q9US44.1 RecName: Full=Uncharacterized transporter C1002.16c | 6.743725202 | 24.98018632 | 1.894061123 | | 0.013464022 |
| Bcin09g00480 | Q2UG11.1 RecName: Full=Beta-cyclopiazonate dehydrogenase; AltName: Full=Beta-Cyclopiazonate oxidocyclase; AltName: Full=FAD-dependent oxidoreductase cpaO; Flags: Precursor >F5HN72.1 RecName: Full=Beta-cyclopiazonate dehydrogenase; AltName: Full=Beta- | 27.07646275 | 100.5031712 | 1.891358323 | | 2.10765E-07 |
| Bcin05g02270 | - | 8.944963559 | 33.02212824 | 1.885167172 | | 0.003105861 |
| Bcin12g05950 | O94284.1 RecName: Full=Sulfide:quinone oxidoreductase, mitochondrial; AltName: Full=Cadmium resistance protein 1; AltName: Full=Heavy metal tolerance protein 2; Flags: Precursor | 18.60622022 | 68.32482698 | 1.879974138 | | 3.63187E-05 |
| Bcin10g05120 | Q53552.1 RecName: Full=Salicylate hydroxylase; AltName: Full=Salicylate 1-monooxygenase | 18.09458402 | 66.52164509 | 1.876443749 | | 3.93795E-05 |
| Bcin01g01590 | - | 493.2583937 | 1793.862712 | 1.862998512 | | 5.7749E-120 |
| Bcin03g09320 | - | 43.9330966 | 159.5835162 | 1.860875995 | | 1.28301E-12 |
| Bcin10g02050 | Q6PW23.1 RecName: Full=Interferon-induced GTP-binding protein Mx; AltName: Full=Interferon-inducible Mx protein | 5.741216261 | 20.80993615 | 1.859528659 | | 0.03275076 |
| Bcin01g10140 | - | 7.985526654 | 28.72718662 | 1.849167037 | | 0.006164686 |
| Bcin10g04180 | - | 163.5482281 | 589.3896625 | 1.848889949 | | 1.38791E-41 |
| Bcin15g03140 | - | 15.8394796 | 57.065792 | 1.845643932 | | 0.000159006 |
| Bcin05g03390 | Q96V64.1 RecName: Full=Glucan 1,3-beta-glucosidase; AltName: Full=Exo-1,3-beta-glucanase; Flags: Precursor | 55.54707682 | 199.096219 | 1.84306994 | | 5.0286E-15 |
| novel.17 | - | 8.224033299 | 29.59933725 | 1.842930067 | | 0.007237333 |
| novel.1081 | - | 5.998953352 | 21.53127771 | 1.839411047 | | 0.022966668 |
| Bcin03g08100 | E9QUT3.1 RecName: Full=Hydroxynaphthalene reductase arp2; AltName: Full=Conidial pigment biosynthesis oxidase arp2 | 180.1155419 | 644.1808407 | 1.839083812 | | 2.14115E-40 |
| Bcin01g09970 | Q6P0U0.1 RecName: Full=N-acetylglucosamine-6-phosphate deacetylase; Short=GlcNAc 6-P deacetylase; AltName: Full=Amidohydrolase domain-containing protein 2 | 105.9545683 | 378.3327205 | 1.836127968 | | 4.57414E-26 |
| novel.1525 | - | 37.93720868 | 135.3359981 | 1.835534574 | | 5.38504E-11 |
| Bcin12g01950 | A7MBI7.1 RecName: Full=Catechol O-methyltransferase | 30.52487355 | 108.6846562 | 1.830607501 | | 2.95942E-07 |
| Bcin05g05020 | Q9UQY0.2 RecName: Full=Demethylsterigmatocystin 6-O-methyltransferase; AltName: Full=Aflatoxin biosynthesis protein O; AltName: Full=Methyltransferase B; AltName: Full=O-methyltransferase I; Short=mt-I | 28.31365305 | 100.6423096 | 1.828105253 | | 1.88543E-06 |
| Bcin11g05510 | P49374.1 RecName: Full=High-affinity glucose transporter | 266.3629499 | 931.1619515 | 1.806361497 | | 5.87582E-55 |
| Bcin01g11290 | Q05031.1 RecName: Full=Mannan endo-1,6-alpha-mannosidase DFG5; AltName: Full=Endo-alpha-1->6-D-mannanase DFG5; Flags: Precursor | 42.61665293 | 149.0902572 | 1.805853931 | | 1.77605E-11 |
| Bcin01g04900 | Q4WQZ2.1 RecName: Full=Glutathione S-transferase-like protein tpcF; AltName: Full=Trypacidin synthesis protein E | 326.8287242 | 1141.974777 | 1.805175256 | | 1.21095E-79 |
| Bcin15g02240 | Q12609.3 RecName: Full=Probable sterigmatocystin biosynthesis P450 monooxygenase stcF; AltName: Full=Cytochrome P450 60A2 | 19.78647904 | 68.96712691 | 1.803364094 | | 2.18759E-05 |
| Bcin06g00520 | - | 140.9781229 | 489.8241074 | 1.796471623 | | 6.63394E-35 |
| Bcin01g11040 | - | 128.6155201 | 442.6238821 | 1.783963738 | | 1.44664E-26 |
| novel.1599 | - | 8.196340417 | 28.26777001 | 1.782700119 | | 0.026122205 |
| Bcin06g03790 | - | 179.7585872 | 615.7158624 | 1.776541913 | | 6.46849E-42 |
| Bcin01g03370 | - | 27.32497755 | 93.41456901 | 1.772480914 | | 4.34257E-07 |
| Bcin03g00370 | - | 6.653703824 | 22.42093431 | 1.748288582 | | 0.026351009 |
| Bcin08g05280 | - | 13.0873716 | 43.81856489 | 1.744966969 | | 0.00066724 |
| Bcin04g05000 | Q4WY82.2 RecName: Full=Linoleate 10R-lipoxygenase; AltName: Full=Cyclooxygenase-like fatty acid oxygenase; AltName: Full=Fatty acid oxygenase ppoC; AltName: Full=Linoleate 10R-dioxygenase; Short=10R-DOX; AltName: Full=Psi-producing oxygenase C; Short | 54.97000798 | 182.0995593 | 1.729756738 | | 9.49556E-10 |
| Bcin08g01180 | - | 6.366728132 | 20.97981136 | 1.719219247 | | 0.032215084 |
| Bcin03g04380 | - | 12.68574707 | 41.81701993 | 1.718500417 | | 0.001544265 |
| Bcin12g03380 | - | 8.237106881 | 27.17107804 | 1.718499423 | | 0.019879126 |
| Bcin12g01100 | Q00001.1 RecName: Full=Rhamnogalacturonase A; Short=RGase A; Short=RHG A; AltName: Full=Rhamnogalacturonan hydrolase A; Flags: Precursor | 1332.767098 | 4372.351367 | 1.713849018 | | 1.9851E-225 |
| Bcin04g06930 | Q9USJ6.1 RecName: Full=NAD(P)H-dependent FMN reductase C4B3.06c; Short=FMN reductase C4B3.06c; AltName: Full=Azoreductase C4B3.06c; AltName: Full=FMN reductase [NAD(P)H] | 44.4339648 | 145.1379636 | 1.710212179 | | 9.49541E-09 |
| Bcin07g06050 | - | 48.32401944 | 157.7921277 | 1.706623351 | | 2.87621E-09 |
| Bcin07g04110 | - | 16.11107614 | 52.68556875 | 1.705713357 | | 0.000839376 |
| novel.757 | - | 16.51961738 | 53.43146776 | 1.692355016 | | 0.000493764 |
| Bcin04g05650 | O34767.1 RecName: Full=Oxalate decarboxylase OxdD | 235.0817761 | 757.4888623 | 1.687309477 | | 8.76079E-40 |
| Bcin05g04010 | - | 84.83629247 | 271.7689715 | 1.678384774 | | 4.56759E-17 |
| Bcin08g06830 | B8NJF4.2 RecName: Full=Probable beta-glucosidase D; AltName: Full=Beta-D-glucoside glucohydrolase D; AltName: Full=Cellobiase D; AltName: Full=Gentiobiase D; Flags: Precursor | 431.6854178 | 1370.59893 | 1.667536623 | | 7.52587E-71 |
| Bcin12g06330 | P53693.2 RecName: Full=Protein rds1 | 9.275037301 | 29.39866388 | 1.667030804 | | 0.014614067 |
| Bcin06g03900 | - | 542.2510358 | 1712.79599 | 1.659496959 | | 3.07813E-72 |
| Bcin02g00390 | P49412.1 RecName: Full=Protein priB | 7.270753259 | 23.03746134 | 1.65912121 | | 0.031897096 |
| Bcin06g04270 | - | 437.2458835 | 1360.243573 | 1.637636891 | | 2.14984E-67 |
| novel.1042 | - | 14.20607509 | 44.07418849 | 1.637262552 | | 0.021562276 |
| Bcin13g04750 | - | 12.37566363 | 38.50262986 | 1.637021406 | | 0.015469913 |
| Bcin01g11470 | - | 12.84655938 | 39.83148213 | 1.635751918 | | 0.010257358 |
| Bcin09g01740 | - | 201.6201019 | 624.0166681 | 1.630264978 | | 3.34777E-35 |
| novel.1302 | - | 12.02790516 | 37.28898667 | 1.628046669 | | 0.010377007 |
| Bcin10g02720 | Q8J0G0.1 RecName: Full=Acyltransferase mlcH; AltName: Full=Compactin biosynthesis protein H | 9.976711102 | 30.69758941 | 1.626799755 | | 0.029231724 |
| Bcin06g04290 | - | 34.67422432 | 107.0313112 | 1.626005321 | | 6.96704E-07 |
| novel.1261 | - | 13.67979357 | 41.74531315 | 1.609387457 | | 0.004112809 |
| Bcin01g05090 | P07921.1 RecName: Full=Lactose permease | 651.967911 | 1987.689356 | 1.608486189 | | 4.9432E-110 |
| Bcin06g00150 | Q6MYX6.1 RecName: Full=Probable quinate permease; AltName: Full=Quinate transporter | 317.3867824 | 963.7829557 | 1.601689997 | | 2.99866E-44 |
| Bcin09g05960 | - | 793.3675833 | 2400.89411 | 1.59736699 | | 9.3075E-132 |
| Bcin05g04760 | P42670.1 RecName: Full=Puromycin resistance protein pur8 | 185.1381855 | 558.732697 | 1.59407125 | | 8.9003E-32 |
| Bcin13g01380 | P21836.1 RecName: Full=Acetylcholinesterase; Short=AChE; Flags: Precursor | 79.33899318 | 238.0714349 | 1.585593702 | | 4.86483E-12 |
| Bcin01g10580 | - | 13.65825756 | 40.95498049 | 1.581889918 | | 0.004403081 |
| novel.745 | - | 7.06764208 | 20.92838688 | 1.570416453 | | 0.048938531 |
| Bcin12g04840 | - | 238.2739961 | 706.6815458 | 1.569146343 | | 4.51671E-35 |
| Bcin09g01890 | - | 116.5698909 | 345.8984495 | 1.568863828 | | 2.84127E-19 |
| Bcin14g04260 | - | 936.5545424 | 2778.649814 | 1.568489367 | | 2.5463E-118 |
| Bcin03g03200 | - | 60.0765161 | 177.3838614 | 1.563785901 | | 5.98685E-08 |
| Bcin01g01380 | B5RCB4.1 RecName: Full=2-keto-3-deoxy-L-rhamnonate aldolase; Short=KDR aldolase; AltName: Full=2-dehydro-3-deoxyrhamnonate aldolase | 34.59882225 | 101.9315293 | 1.5568274 | | 5.32992E-06 |
| novel.865 | - | 17.81373917 | 52.34113779 | 1.556015832 | | 0.004592072 |
| Bcin07g00260 | Q5RLY7.2 RecName: Full=Cysteine dioxygenase; Short=CDO | 59.03166896 | 173.4111036 | 1.55552267 | | 2.55097E-09 |
| Bcin06g06410 | G2QJ27.1 RecName: Full=Acetylesterase; AltName: Full=Carbohydrate esterase family 16 protein; Flags: Precursor | 867.0562461 | 2544.976415 | 1.55366742 | | 1.9759E-104 |
| Bcin04g04800 | P87025.4 RecName: Full=Trihydroxynaphthalene reductase; AltName: Full=T3HN reductase | 770.2173705 | 2254.033893 | 1.549494034 | | 1.27209E-80 |
| Bcin01g04910 | - | 15.27320479 | 44.6080875 | 1.545509223 | | 0.002594551 |
| Bcin03g00005 | Q00050.2 RecName: Full=Asp-hemolysin; Short=Asp-HS; Flags: Precursor | 700.8904572 | 2045.795593 | 1.545279498 | | 8.3682E-102 |
| Bcin14g03720 | - | 14.72309499 | 42.6829905 | 1.538947501 | | 0.004424308 |
| Bcin08g05540 | - | 13099.63711 | 37891.73768 | 1.532426179 | | 0 |
| Bcin03g02970 | D4B5F9.2 RecName: Full=Probable peptidoglycan-N-acetylglucosamine deacetylase ARB_03699; Short=Peptidoglycan GlcNAc deacetylase; AltName: Full=Peptidoglycan N-deacetylase; Short=PG N-deacetylase; Flags: Precursor | 62.66857164 | 181.1619433 | 1.531014546 | | 6.34498E-11 |
| Bcin01g05800 | Q00808.1 RecName: Full=Vegetative incompatibility protein HET-E-1 | 15.1870347 | 43.87831766 | 1.529710737 | | 0.041433447 |
| Bcin16g00060 | - | 1403.463717 | 4037.83918 | 1.524565298 | | 7.4377E-145 |
| Bcin14g01090 | P46333.3 RecName: Full=Probable metabolite transport protein CsbC | 12.39413422 | 35.68070348 | 1.524199627 | | 0.012037886 |
| novel.1348 | - | 43.69075137 | 125.8244454 | 1.524132352 | | 0.000142324 |
| Bcin05g02030 | O14091.1 RecName: Full=General alpha-glucoside permease | 12.36337591 | 35.56103731 | 1.520807784 | | 0.022868186 |
| Bcin08g06100 | Q9F131.1 RecName: Full=3-hydroxybenzoate 6-hydroxylase 1; AltName: Full=Constitutive 3-hydroxybenzoate 6-hydroxylase | 98.27607301 | 280.9084768 | 1.516496279 | | 5.19995E-15 |
| Bcin03g00280 | A1CYC2.2 RecName: Full=Probable pectin lyase A; Short=PLA; Flags: Precursor | 1661.544786 | 4753.48257 | 1.516376365 | | 2.8369E-201 |
| Bcin01g00580 | Q7FAX1.1 RecName: Full=Peroxygenase >A2XVG1.1 RecName: Full=Peroxygenase | 8.859553326 | 25.39638472 | 1.515971454 | | 0.046513799 |
| Bcin01g09990 | Q4U3Y2.1 RecName: Full=Hexokinase-1; AltName: Full=Hexokinase I | 296.6600709 | 846.680561 | 1.512548443 | | 2.05789E-40 |
| Bcin06g04520 | Q9C168.2 RecName: Full=Catalase-1 | 61.48215596 | 174.7925345 | 1.507504964 | | 6.48447E-10 |
| Bcin07g06820 | - | 889.634066 | 2523.603178 | 1.504052818 | | 1.9871E-113 |
| Bcin02g01620 | Q4WLD2.1 RecName: Full=Terpene cyclase pyr4; AltName: Full=Pyripyropene synthesis protein 4 | 94.26137407 | 266.7840753 | 1.501280168 | | 5.11324E-12 |
| Bcin03g07750 | - | 32.05980756 | 90.84942031 | 1.501186775 | | 9.13274E-05 |
| Bcin10g06060 | - | 1039.532605 | 2929.131553 | 1.494916541 | | 1.83181E-96 |
| Bcin04g06530 | - | 33.35468921 | 94.04819663 | 1.494773654 | | 2.79005E-05 |
| Bcin11g00940 | - | 44.64860384 | 125.5403946 | 1.492515869 | | 1.48966E-06 |
| Bcin05g03180 | - | 26.3278396 | 73.67474118 | 1.482515702 | | 0.000123118 |
| Bcin14g03170 | P80402.2 RecName: Full=2,3-dihydroxybenzoate decarboxylase; Short=2,3-DHBA decarboxylase; Short=DHBD; AltName: Full=o-pyrocatechuate decarboxylase | 227.8433008 | 636.0069937 | 1.481106338 | | 2.75605E-29 |
| Bcin06g04160 | G0RNA2.1 RecName: Full=L-xylo-3-hexulose reductase | 63.26023377 | 176.742595 | 1.480880211 | | 9.36242E-08 |
| Bcin03g01520 | P42328.1 RecName: Full=Alcohol dehydrogenase; AltName: Full=ADH-HT | 259.921553 | 723.8072314 | 1.477217557 | | 1.84723E-36 |
| Bcin03g08110 | O14434.1 RecName: Full=Scytalone dehydratase arp1; AltName: Full=Conidial pigment biosynthesis oxidase arp1 | 178.7398872 | 497.1631866 | 1.475640153 | | 6.48915E-22 |
| Bcin10g00310 | D4B0V1.1 RecName: Full=Probable glucan endo-1,3-beta-glucosidase ARB_02077; AltName: Full=(1->3)-beta-glucan endohydrolase ARB_02077; Short=(1->3)-beta-glucanase ARB_02077; Flags: Precursor | 1180.989942 | 3279.724222 | 1.473921617 | | 1.7453E-121 |
| novel.1425 | - | 9.609722188 | 26.51364238 | 1.465722207 | | 0.046827363 |
| Bcin06g01730 | - | 101.2660058 | 279.3755475 | 1.464191649 | | 0.007167549 |
| Bcin14g01360 | Q6ZQW0.4 RecName: Full=Indoleamine 2,3-dioxygenase 2; Short=IDO-2; AltName: Full=Indoleamine 2,3-dioxygenase-like protein 1; AltName: Full=Indoleamine-pyrrole 2,3-dioxygenase-like protein 1 | 919.4810426 | 2533.892904 | 1.462375421 | | 4.2802E-114 |
| novel.1460 | - | 14.95083363 | 41.23441141 | 1.462316962 | | 0.005991331 |
| Bcin12g05160 | Q0R4L2.1 RecName: Full=Pyranose dehydrogenase 3; Short=PDH 3; AltName: Full=Pyranose:quinone oxidoreductase 3; Flags: Precursor | 15.59327037 | 42.98454728 | 1.461764523 | | 0.008480806 |
| Bcin07g04230 | F1SWA0.1 RecName: Full=Zerumbone synthase | 33.1269899 | 90.74628797 | 1.457337496 | | 0.002266791 |
| Bcin06g03270 | - | 2713.072779 | 7440.617807 | 1.455527991 | | 3.33401E-35 |
| Bcin01g07640 | - | 12.41260481 | 34.05444829 | 1.454755314 | | 0.014730276 |
| Bcin03g08050 | Q4WZA8.1 RecName: Full=Conidial pigment polyketide synthase alb1; AltName: Full=Conidial pigment biosynthesis protein alb1; AltName: Full=Naphthopyrone synthase | 211.1212801 | 574.9032013 | 1.44523826 | | 1.38586E-25 |
| Bcin11g01040 | - | 912.3310031 | 2482.039146 | 1.443374707 | | 8.69293E-87 |
| Bcin01g00790 | - | 278.7585905 | 756.0950706 | 1.439772422 | | 8.43745E-35 |
| Bcin16g00220 | - | 20.99980175 | 56.90886424 | 1.439139194 | | 0.002091497 |
| Bcin05g05010 | - | 13.14430308 | 35.51937856 | 1.437784911 | | 0.024085134 |
| novel.1055 | - | 22.35316059 | 60.4800327 | 1.436617532 | | 0.000952887 |
| Bcin08g00400 | - | 191.064043 | 516.28667 | 1.435227129 | | 2.78551E-18 |
| novel.607 | - | 12.65574861 | 34.22750451 | 1.432939209 | | 0.038150513 |
| Bcin15g01600 | P36218.1 RecName: Full=Endo-1,4-beta-xylanase 1; Short=EX 1; Short=Xylanase 1; AltName: Full=1,4-beta-D-xylan xylanohydrolase 1; AltName: Full=Acidic endo-beta-1,4-xylanase; Flags: Precursor >G0R947.1 RecName: Full=Endo-1,4-beta-xylanase 1; Short=EX | 5853.361667 | 15761.36304 | 1.428899551 | | 0 |
| Bcin13g01360 | O74923.1 RecName: Full=Uncharacterized transporter C757.13 | 127.0297855 | 340.9177057 | 1.425294353 | | 1.4814E-15 |
| novel.1006 | - | 27.91280169 | 74.08469887 | 1.41118903 | | 0.000908481 |
| Bcin05g03580 | - | 1141.482337 | 3034.149183 | 1.410089119 | | 6.53948E-82 |
| Bcin08g00940 | - | 51107.35124 | 135685.331 | 1.408645883 | | 0 |
| Bcin16g01270 | - | 3327.098113 | 8830.912496 | 1.408301365 | | 0 |
| Bcin07g04000 | D4ATR3.1 RecName: Full=Uncharacterized secreted glycosidase ARB_07629; Flags: Precursor | 229.4851024 | 608.7032407 | 1.406471144 | | 6.19096E-19 |
| Bcin13g00200 | B0XQS8.1 RecName: Full=Probable quinate permease; AltName: Full=Quinate transporter | 406.908624 | 1079.04205 | 1.406402833 | | 8.87446E-44 |
| Bcin15g05080 | P0C7S9.1 RecName: Full=1,3-beta-glucanosyltransferase gel1; AltName: Full=Glucan elongating glucanosyltransferase 1; Flags: Precursor | 623.0610367 | 1646.442051 | 1.401815942 | | 1.9731E-79 |
| Bcin02g00090 | - | 14.66770923 | 38.65730869 | 1.399129861 | | 0.010644189 |
| Bcin02g01730 | P54007.1 RecName: Full=Uncharacterized protein YLR460C | 57.43515299 | 151.4987615 | 1.398153989 | | 6.76895E-07 |
| Bcin01g09830 | - | 50.31441802 | 132.5604716 | 1.397041607 | | 1.18138E-06 |
| novel.345 | - | 10.15598069 | 26.75528503 | 1.395480895 | | 0.039348539 |
| novel.1388 | - | 46.0273627 | 121.1393623 | 1.394155797 | | 3.02929E-06 |
| Bcin03g07730 | P00440.5 RecName: Full=Tyrosinase; AltName: Full=Monophenol monooxygenase; Flags: Precursor | 15.84178517 | 41.73514203 | 1.39381347 | | 0.014766388 |
| Bcin02g03050 | Q08268.1 RecName: Full=Probable transporter MCH4 | 323.8189178 | 848.2136518 | 1.389026586 | | 1.91539E-34 |
| Bcin15g01680 | - | 24.63441217 | 64.41962212 | 1.388960003 | | 0.001300162 |
| Bcin13g04070 | - | 41.89343041 | 109.0713332 | 1.382191689 | | 1.66675E-05 |
| novel.238 | - | 15.32091398 | 39.81160521 | 1.378403342 | | 0.010466059 |
| Bcin01g11160 | Q9UUZ2.1 RecName: Full=Endo-xylogalacturonan hydrolase A; Flags: Precursor | 63.17635656 | 163.9762823 | 1.376631761 | | 1.64931E-07 |
| Bcin09g05470 | P78581.1 RecName: Full=Tannase; Contains: RecName: Full=Tannase 33 kDa subunit; Contains: RecName: Full=Tannase 30 kDa subunit; Flags: Precursor | 64.4297379 | 166.9341653 | 1.37405126 | | 2.67127E-07 |
| Bcin12g03840 | - | 22.73248925 | 58.76184907 | 1.372805838 | | 0.006087547 |
| Bcin06g04150 | Q9SCU0.1 RecName: Full=Short-chain dehydrogenase reductase 2a; Short=AtSDR2a | 607.4231879 | 1568.918614 | 1.36923719 | | 3.78333E-64 |
| Bcin15g05470 | - | 36.8985184 | 95.20555286 | 1.366182758 | | 6.65144E-05 |
| Bcin07g05560 | - | 84.82553778 | 218.489873 | 1.365386784 | | 2.24649E-10 |
| Bcin07g03780 | A1D2R3.1 RecName: Full=Probable quinate permease; AltName: Full=Quinate transporter | 2251.438379 | 5794.343953 | 1.363884976 | | 4.549E-155 |
| Bcin03g00200 | Q44470.1 RecName: Full=Putative tartrate transporter | 505.9209671 | 1298.156311 | 1.359041108 | | 7.57075E-50 |
| Bcin10g05370 | O94562.1 RecName: Full=Uncharacterized aminotransferase C1771.03c | 1231.357452 | 3156.641893 | 1.358200526 | | 3.7578E-113 |
| Bcin02g00410 | P18696.2 RecName: Full=Proline-specific permease; AltName: Full=Proline transport protein | 37.24247761 | 95.43661829 | 1.357115952 | | 0.001554039 |
| Bcin06g04590 | P47734.2 RecName: Full=S-(hydroxymethyl)glutathione dehydrogenase; AltName: Full=Glutathione-dependent formaldehyde dehydrogenase; Short=FALDH; Short=FDH | 13.07736344 | 33.44572214 | 1.353973083 | | 0.029953128 |
| Bcin05g06610 | P39932.2 RecName: Full=Sugar transporter STL1 | 29.60698898 | 75.66848526 | 1.35379063 | | 0.00022036 |
| Bcin05g00740 | D2D3B6.1 RecName: Full=Fumonisin B1 esterase | 16.99127299 | 43.36376817 | 1.353370748 | | 0.026759724 |
| Bcin07g04120 | - | 18.16765451 | 46.41669933 | 1.352896087 | | 0.012027851 |
| Bcin03g05810 | - | 171.3090942 | 437.1630961 | 1.35066875 | | 7.4725E-19 |
| Bcin03g06320 | - | 36.77540713 | 93.87323622 | 1.350060193 | | 0.000197139 |
| Bcin09g05970 | - | 176.0147122 | 447.2469241 | 1.346361342 | | 2.87085E-17 |
| Bcin03g02020 | - | 14.93700019 | 37.98196167 | 1.345812923 | | 0.026824827 |
| Bcin10g01780 | - | 80.32612297 | 203.9776389 | 1.344793672 | | 5.36863E-10 |
| Bcin08g07120 | - | 297.8695684 | 750.0388293 | 1.331320285 | | 6.69753E-26 |
| novel.260 | - | 27.32882884 | 68.71849414 | 1.329795831 | | 0.000886023 |
| Bcin10g02160 | - | 15.53788461 | 38.82452958 | 1.320689465 | | 0.024758305 |
| Bcin13g01120 | - | 102.9654734 | 256.4082735 | 1.31632369 | | 1.02583E-09 |
| Bcin07g01840 | Q47944.1 RecName: Full=L-sorbose 1-dehydrogenase; Short=SDH | 28.45366319 | 70.64515132 | 1.313399732 | | 0.002598082 |
| Bcin06g02050 | - | 11.17465465 | 27.71589829 | 1.3120783 | | 0.04989658 |
| Bcin03g06470 | W7MT31.1 RecName: Full=Reducing polyketide synthase FUB1; AltName: Full=Fusaric acid biosynthesis protein 1 | 15.53788461 | 38.64904177 | 1.311500441 | | 0.036789508 |
| Bcin09g01790 | - | 903.6822254 | 2240.548649 | 1.30998014 | | 2.0945E-61 |
| Bcin03g00380 | D7UQ40.1 RecName: Full=Bifunctional solanapyrone synthase; AltName: Full=Prosolanapyrone-II oxidase; AltName: Full=Prosolanapyrone-III cycloisomerase; Flags: Precursor | 494.1846627 | 1224.83046 | 1.309802315 | | 4.02972E-42 |
| Bcin01g00870 | P28246.4 RecName: Full=Bicyclomycin resistance protein; AltName: Full=Sulfonamide resistance protein | 495.6673677 | 1228.330306 | 1.309462234 | | 8.24931E-44 |
| Bcin14g02080 | - | 134.4690663 | 332.943134 | 1.308368017 | | 4.70804E-15 |
| Bcin06g04140 | P49374.1 RecName: Full=High-affinity glucose transporter | 177.8790128 | 439.3142347 | 1.305485164 | | 1.27202E-10 |
| Bcin06g03800 | - | 162.6896199 | 400.7793678 | 1.301518077 | | 2.84802E-16 |
| Bcin02g09240 | - | 161.1708376 | 396.5316361 | 1.299308824 | | 7.28854E-17 |
| novel.1033 | - | 12.12177783 | 29.73263964 | 1.295992422 | | 0.038442475 |
| novel.1082 | - | 22.94791416 | 56.26927928 | 1.294002621 | | 0.003467937 |
| Bcin07g04770 | - | 32.63533068 | 79.36521587 | 1.280421602 | | 0.002255043 |
| novel.1489 | - | 977.7417672 | 2374.688143 | 1.280328455 | | 7.32588E-84 |
| Bcin12g03000 | - | 332.7707848 | 806.1904885 | 1.276455413 | | 8.19565E-29 |
| novel.1184 | - | 44.0631506 | 106.4637771 | 1.273204602 | | 0.000588089 |
| Bcin14g03730 | - | 68.62598598 | 164.8788863 | 1.265426898 | | 2.27844E-06 |
| Bcin05g01780 | - | 37.75716592 | 90.76188974 | 1.264743768 | | 0.000737094 |
| Bcin03g01250 | - | 740.9919758 | 1774.926106 | 1.259898654 | | 1.44813E-64 |
| novel.517 | - | 54.4745368 | 130.2851076 | 1.259171134 | | 3.44283E-05 |
| Bcin07g00920 | Q12177.1 RecName: Full=Uncharacterized protein YLL056C | 88.12081284 | 210.7429031 | 1.257071463 | | 2.87534E-08 |
| Bcin08g02660 | Q08902.1 RecName: Full=Drug resistance protein YOR378W | 646.9137099 | 1540.322826 | 1.251588339 | | 1.49397E-59 |
| novel.232 | - | 14.74926817 | 35.04898006 | 1.250883336 | | 0.048316868 |
| Bcin15g03080 | Q00298.1 RecName: Full=Cutinase; AltName: Full=Cutin hydrolase; Flags: Precursor | 4066.736255 | 9644.539061 | 1.245642133 | | 7.7337E-170 |
| Bcin11g01370 | P49426.1 RecName: Full=Glucan 1,3-beta-glucosidase; AltName: Full=1,3-beta-D-glucanohydrolase; AltName: Full=Exo-beta 1,3 glucanase; Flags: Precursor | 37.32322467 | 88.33478532 | 1.24463543 | | 0.000955528 |
| Bcin04g00010 | - | 266.3914021 | 630.3136821 | 1.242579655 | | 1.37238E-24 |
| Bcin01g08340 | - | 1289.203557 | 3049.469439 | 1.242006475 | | 1.33038E-92 |
| Bcin06g04950 | Q99385.1 RecName: Full=Vacuolar calcium ion transporter; AltName: Full=High copy number undoes manganese protein 1; AltName: Full=Manganese resistance 1 protein; AltName: Full=Vacuolar Ca(2+)/H(+) exchanger | 57.18281291 | 135.1103626 | 1.239281977 | | 2.99078E-05 |
| Bcin03g00480 | Q2LMP0.1 RecName: Full=Endo-1,4-beta-xylanase 11A; Short=Xylanase 11A; AltName: Full=1,4-beta-D-xylan xylanohydrolase 11A; Flags: Precursor | 15875.60305 | 37252.20289 | 1.230587488 | | 0 |
| Bcin06g04940 | O74628.1 RecName: Full=Uncharacterized oxidoreductase C162.03 | 9506.292415 | 22304.85566 | 1.23036542 | | 0 |
| Bcin01g07390 | - | 64.63742089 | 151.8517427 | 1.230101798 | | 1.30213E-05 |
| novel.1498 | - | 40.52157495 | 94.56920955 | 1.223401751 | | 0.001424271 |
| Bcin04g05960 | - | 267.9932884 | 625.9701937 | 1.223027344 | | 9.98574E-22 |
| Bcin06g06670 | - | 216.770968 | 506.2326666 | 1.222851129 | | 1.28133E-18 |
| novel.91 | - | 25.8600486 | 60.20528231 | 1.219285806 | | 0.00663598 |
| Bcin03g04690 | - | 415.7090589 | 966.3760255 | 1.217659204 | | 8.17163E-26 |
| novel.1285 | - | 20.66202542 | 48.01424394 | 1.2169723 | | 0.046154635 |
| novel.504 | - | 21.32987549 | 49.53550195 | 1.214430642 | | 0.013123804 |
| Bcin06g07380 | - | 231.3602363 | 535.976004 | 1.212790292 | | 9.08468E-17 |
| Bcin15g05510 | P37967.2 RecName: Full=Para-nitrobenzyl esterase; AltName: Full=Intracellular esterase B; AltName: Full=PNB carboxy-esterase; Short=PNBCE | 185.9198592 | 430.7773354 | 1.212265735 | | 4.83737E-17 |
| Bcin13g00280 | Q9Y8H5.2 RecName: Full=Delta(12) fatty acid desaturase; AltName: Full=Delta-12 fatty acid desaturase | 573.6815455 | 1324.994103 | 1.207795608 | | 4.12639E-39 |
| Bcin14g01190 | - | 152.0550669 | 349.4109799 | 1.200865766 | | 5.83067E-14 |
| Bcin07g05410 | P53048.1 RecName: Full=General alpha-glucoside permease; AltName: Full=Maltose permease MAL11; AltName: Full=Maltose transport protein MAL11 | 25.65539171 | 58.67332507 | 1.195377502 | | 0.031475955 |
| Bcin16g02830 | - | 40.04612008 | 91.67136189 | 1.194343513 | | 0.000685122 |
| novel.119 | - | 45.5411011 | 104.1933777 | 1.193964223 | | 0.000200269 |
| Bcin15g02750 | - | 22.03925187 | 50.29041728 | 1.192038597 | | 0.010388083 |
| Bcin09g01760 | - | 214.1095892 | 488.3325927 | 1.189072644 | | 6.03079E-16 |
| Bcin05g00810 | P78972.1 RecName: Full=WD repeat-containing protein slp1 | 698.0999276 | 1589.710416 | 1.187723454 | | 1.78243E-49 |
| Bcin13g02680 | - | 65.49147058 | 149.2559186 | 1.187375962 | | 1.69314E-05 |
| Bcin05g01660 | P29717.4 RecName: Full=Glucan 1,3-beta-glucosidase; AltName: Full=Exo-1,3-beta-glucanase; Flags: Precursor | 2266.094273 | 5147.135365 | 1.183503611 | | 1.1511E-136 |
| Bcin10g02560 | B9W4V8.2 RecName: Full=Aromatic peroxygenase | 68.17510654 | 154.7761127 | 1.183219674 | | 1.01472E-06 |
| Bcin01g10790 | - | 102.9339159 | 233.7197009 | 1.182092643 | | 1.19773E-07 |
| Bcin01g03540 | - | 26.38631681 | 59.57246475 | 1.174244995 | | 0.005515417 |
| Bcin01g10910 | Q9US37.1 RecName: Full=Uncharacterized transporter C1039.04 | 144.511158 | 325.3578937 | 1.170735019 | | 1.86224E-12 |
| Bcin02g00260 | - | 454.0624327 | 1020.364268 | 1.168272666 | | 3.53177E-31 |
| Bcin02g07390 | - | 76.9846451 | 172.9609254 | 1.167426942 | | 1.63486E-06 |
| Bcin02g00180 | - | 16.48731336 | 37.06260106 | 1.166115642 | | 0.036495654 |
| Bcin09g00210 | O74923.1 RecName: Full=Uncharacterized transporter C757.13 | 505.600299 | 1130.631555 | 1.161374496 | | 2.95247E-30 |
| Bcin05g05030 | Q0UPV4.1 RecName: Full=DNA replication complex GINS protein PSF3 | 262.7213835 | 587.1424438 | 1.160213715 | | 1.62343E-15 |
| Bcin01g04890 | Q27517.1 RecName: Full=Putative cytochrome P450 CYP13A3 | 97.97678359 | 218.5277833 | 1.158324908 | | 6.58763E-08 |
| novel.268 | - | 86.1973539 | 192.2868651 | 1.156843332 | | 9.52399E-08 |
| Bcin12g02360 | - | 33.33236732 | 74.2694871 | 1.155022748 | | 0.012487828 |
| Bcin11g01560 | - | 126.2234183 | 280.9731329 | 1.153016797 | | 8.4894E-08 |
| Bcin16g00810 | - | 62.97173837 | 140.005074 | 1.152379296 | | 0.000104232 |
| Bcin05g05050 | - | 64.70746529 | 143.8167303 | 1.152171725 | | 9.79349E-06 |
| novel.414 | - | 89.30646868 | 198.1640329 | 1.148758682 | | 1.76168E-07 |
| Bcin08g04590 | - | 517.4803613 | 1146.748384 | 1.148391826 | | 1.30486E-37 |
| Bcin01g06990 | B4JII0.1 RecName: Full=Spastin | 16.87277286 | 37.39270705 | 1.147606116 | | 0.043111757 |
| Bcin12g02830 | Q2UNR0.1 RecName: Full=Probable beta-glucosidase D; AltName: Full=Beta-D-glucoside glucohydrolase D; AltName: Full=Cellobiase D; AltName: Full=Gentiobiase D; Flags: Precursor | 300.2308858 | 664.2263997 | 1.146167332 | | 9.27047E-19 |
| Bcin09g02230 | S0DPY2.1 RecName: Full=Efflux pump apf11; AltName: Full=Apicidin F synthesis protein 11 | 25.24147946 | 55.79853605 | 1.14606924 | | 0.012509688 |
| Bcin01g08110 | P28351.1 RecName: Full=Alpha-galactosidase A; AltName: Full=Melibiase A; Flags: Precursor | 117.0507428 | 257.689287 | 1.139066764 | | 5.62193E-08 |
| novel.559 | - | 44.8940399 | 99.01617568 | 1.138614252 | | 0.002634951 |
| Bcin07g03720 | - | 588.3814541 | 1294.749914 | 1.137338999 | | 1.54973E-35 |
| Bcin02g00070 | - | 1264.260517 | 2779.226324 | 1.136805265 | | 2.3824E-73 |
| Bcin10g01070 | Q9X248.1 RecName: Full=3-oxoacyl-[acyl-carrier-protein] reductase FabG; AltName: Full=3-ketoacyl-acyl carrier protein reductase; AltName: Full=Beta-Ketoacyl-acyl carrier protein reductase; AltName: Full=Beta-ketoacyl-ACP reductase | 196.2451147 | 430.7527786 | 1.134338071 | | 1.49586E-15 |
| Bcin02g01420 | Q08806.1 RecName: Full=Alpha-amylase 2; AltName: Full=1,4-alpha-D-glucan glucanohydrolase 2; Flags: Precursor | 345.248011 | 757.3166374 | 1.133498092 | | 9.6601E-24 |
| Bcin12g03650 | Q00258.1 RecName: Full=Norsolorinic acid reductase A; AltName: Full=Aflatoxin biosynthesis protein E | 5616.41347 | 12314.16892 | 1.132639264 | | 2.6463E-281 |
| Bcin16g03320 | - | 19.69645767 | 43.1342622 | 1.130027947 | | 0.031397269 |
| novel.746 | - | 30.33483596 | 66.3174467 | 1.128854508 | | 0.006850404 |
| novel.102 | - | 30.0270841 | 65.51740824 | 1.126681318 | | 0.006104703 |
| Bcin01g01240 | - | 43.64224361 | 95.20913925 | 1.125644939 | | 0.000551206 |
| Bcin08g00130 | Q9F131.1 RecName: Full=3-hydroxybenzoate 6-hydroxylase 1; AltName: Full=Constitutive 3-hydroxybenzoate 6-hydroxylase | 57.1974062 | 124.6950503 | 1.123424529 | | 6.64375E-05 |
| Bcin06g07340 | D4B1P2.1 RecName: Full=Uncharacterized FAD-linked oxidoreductase ARB_02372; Flags: Precursor | 161.9563759 | 352.5649172 | 1.12275504 | | 2.2628E-11 |
| Bcin08g06110 | O74556.1 RecName: Full=Putative mannan endo-1,6-alpha-mannosidase C970.02; AltName: Full=Endo-alpha-1->6-D-mannanase C970.02; Flags: Precursor | 124.0191539 | 270.1376304 | 1.122360229 | | 4.32672E-09 |
| Bcin09g01910 | Q8IU85.1 RecName: Full=Calcium/calmodulin-dependent protein kinase type 1D; AltName: Full=CaM kinase I delta; Short=CaM kinase ID; Short=CaM-KI delta; Short=CaMKI delta; Short=CaMKID; AltName: Full=CaMKI-like protein kinase; Short=CKLiK | 32.02056081 | 69.81893459 | 1.121996873 | | 0.008510023 |
| Bcin01g10750 | Q9Y8H5.2 RecName: Full=Delta(12) fatty acid desaturase; AltName: Full=Delta-12 fatty acid desaturase | 164.8077009 | 358.3157713 | 1.119625484 | | 3.77821E-10 |
| Bcin07g03600 | Q4IBU5.1 RecName: Full=Probable kinetochore protein SPC24 | 179.8393996 | 389.722161 | 1.116771488 | | 9.8134E-11 |
| Bcin02g01610 | A5PJM4.1 RecName: Full=Apoptosis-inducing factor 2 | 500.073539 | 1081.959308 | 1.113349432 | | 1.95954E-29 |
| Bcin05g01250 | - | 777.558744 | 1680.354261 | 1.111534729 | | 6.35807E-34 |
| Bcin11g06310 | P52578.1 RecName: Full=Isoflavone reductase homolog; AltName: Full=CP100 | 25.49460541 | 55.05419722 | 1.111490179 | | 0.012917023 |
| Bcin01g02720 | - | 135.7255131 | 293.0182254 | 1.110687371 | | 6.17771E-09 |
| Bcin12g03260 | - | 1504.047058 | 3246.052134 | 1.109890964 | | 1.54488E-91 |
| Bcin08g03460 | Q6GV12.1 RecName: Full=3-ketodihydrosphingosine reductase; Short=KDS reductase; AltName: Full=3-dehydrosphinganine reductase; AltName: Full=Follicular variant translocation protein 1 homolog; Short=FVT-1; Flags: Precursor | 789.1728422 | 1703.116974 | 1.109870428 | | 9.97506E-41 |
| Bcin10g05010 | B0Y0Q3.1 RecName: Full=Probable rhamnogalacturonase B; Short=RGase B; Short=RHG B; Flags: Precursor | 467.2675186 | 1007.947532 | 1.109665501 | | 4.14679E-31 |
| Bcin03g00890 | - | 23.92349007 | 51.61302884 | 1.108924385 | | 0.019099965 |
| Bcin09g03310 | - | 212.0761445 | 457.5286893 | 1.108732297 | | 5.14498E-14 |
| Bcin09g07140 | - | 2512.340606 | 5417.392581 | 1.108635994 | | 4.1795E-143 |
| Bcin01g06790 | Q00017.1 RecName: Full=Rhamnogalacturonan acetylesterase; Short=RGAE; Flags: Precursor | 85.53951199 | 184.4209178 | 1.10693758 | | 2.0893E-05 |
| Bcin15g01880 | - | 44.8832852 | 96.67285408 | 1.106561678 | | 0.000383633 |
| Bcin12g05820 | - | 143.832566 | 309.845982 | 1.106447763 | | 4.30468E-10 |
| Bcin01g05510 | P38865.1 RecName: Full=Copper transport protein CTR2; Short=Copper transporter 2 | 656.121009 | 1412.525547 | 1.106328224 | | 2.79812E-41 |
| Bcin01g02870 | Q9HDX2.1 RecName: Full=Uncharacterized lactate 2-monooxygenase PB1A11.03 | 537.7262077 | 1157.588384 | 1.106081615 | | 3.06871E-37 |
| novel.585 | - | 35.60981964 | 76.6583365 | 1.105774693 | | 0.005971978 |
| Bcin05g00030 | - | 32.86766776 | 70.51583999 | 1.103165105 | | 0.014870103 |
| Bcin02g04320 | - | 276.1942926 | 592.8751025 | 1.102609312 | | 9.68076E-16 |
| Bcin02g00400 | - | 24.17352458 | 51.92597289 | 1.101840159 | | 0.036492705 |
| Bcin02g04620 | - | 570.4653938 | 1224.138397 | 1.101737766 | | 2.72519E-35 |
| Bcin01g07850 | - | 21.30063689 | 45.6638102 | 1.098761674 | | 0.023517337 |
| Bcin04g00080 | - | 90.45593048 | 193.4723644 | 1.097069255 | | 7.80275E-07 |
| novel.795 | - | 25.34838639 | 54.25369272 | 1.09540178 | | 0.027286391 |
| Bcin01g01430 | Q10072.1 RecName: Full=Uncharacterized transporter C3H1.06c | 2487.928062 | 5314.849937 | 1.095251938 | | 6.3483E-104 |
| Bcin01g10770 | O13317.1 RecName: Full=Isotrichodermin C-15 hydroxylase; AltName: Full=Cytochrome P450 65A1 | 80.98165931 | 172.7423406 | 1.09383461 | | 9.57458E-06 |
| Bcin01g08610 | Q6F6Y2.1 RecName: Full=FAD-dependent urate hydroxylase; AltName: Full=Flavoprotein urate hydroxylase | 1819.910592 | 3881.526609 | 1.092954521 | | 1.2316E-100 |
| Bcin14g03870 | - | 26.45097689 | 56.37571463 | 1.092519643 | | 0.035776021 |
| Bcin02g06320 | A0A0B5EMG9.1 RecName: Full=Efflux pump FUBT; AltName: Full=Fusaric acid biosynthesis protein T; AltName: Full=Fusaric acid transporter | 130.1266125 | 277.2507499 | 1.092452612 | | 1.13211E-08 |
| Bcin03g01280 | - | 362.4224048 | 772.8068088 | 1.092275527 | | 1.50985E-23 |
| Bcin12g00390 | Q9P6I8.1 RecName: Full=Zinc-type alcohol dehydrogenase-like protein C1198.01 | 128.2239171 | 272.9323457 | 1.091405995 | | 3.23431E-07 |
| Bcin07g04100 | Q9P900.1 RecName: Full=Demethylsterigmatocystin 6-O-methyltransferase; AltName: Full=Methyltransferase B | 27.80200415 | 59.18734788 | 1.090468984 | | 0.015521322 |
| Bcin06g01530 | Q3M7P3.1 RecName: Full=Anhydro-N-acetylmuramic acid kinase; AltName: Full=AnhMurNAc kinase | 1121.221204 | 2382.8705 | 1.087695928 | | 7.24601E-70 |
| novel.910 | - | 22.95559073 | 48.60765169 | 1.083092651 | | 0.026659239 |
| Bcin04g03750 | P36029.1 RecName: Full=Polyamine transporter TPO5 | 129.1779303 | 273.2725016 | 1.080379073 | | 4.6767E-09 |
| Bcin10g01470 | Q5AUY5.1 RecName: Full=Zinc-binding alcohol dehydrogenase domain-containing protein cipB; AltName: Full=Concanamycin-induced protein B | 120.5961044 | 254.7400762 | 1.078665015 | | 2.22717E-08 |
| Bcin03g01580 | O16171.1 RecName: Full=Esterase-5C; Short=Est-5C; AltName: Full=Carboxylic-ester hydrolase 5C; Short=Carboxylesterase-5C; Flags: Precursor | 127.8168822 | 269.6806454 | 1.077909127 | | 3.46823E-09 |
| Bcin05g03920 | - | 355.5462547 | 749.4044714 | 1.075575933 | | 7.6502E-19 |
| Bcin04g06330 | O62742.1 RecName: Full=Non-specific lipid-transfer protein; Short=NSL-TP; AltName: Full=Propanoyl-CoA C-acyltransferase; AltName: Full=SCP-chi; AltName: Full=SCPX; AltName: Full=Sterol carrier protein 2; Short=SCP-2; AltName: Full=Sterol carrier prot | 3953.701772 | 8328.718908 | 1.074893444 | | 2.9123E-174 |
| Bcin12g04870 | Q9P3V5.1 RecName: Full=Uncharacterized transporter C1348.05 | 616.1525965 | 1297.286977 | 1.074274664 | | 5.17098E-32 |
| Bcin16g03190 | - | 373.0685244 | 785.2979044 | 1.073358431 | | 2.62305E-19 |
| Bcin07g00090 | - | 91.40231982 | 192.1158957 | 1.07227586 | | 2.81148E-06 |
| Bcin14g01630 | B0XT32.1 RecName: Full=Probable pectate lyase A; Flags: Precursor | 209.7218231 | 440.2104153 | 1.069547236 | | 2.04355E-11 |
| Bcin12g01620 | - | 33.07848152 | 69.41568364 | 1.069359892 | | 0.008142298 |
| Bcin11g02030 | - | 1314.029487 | 2754.961301 | 1.068274668 | | 3.07275E-72 |
| Bcin02g04360 | Q4WZB3.1 RecName: Full=Heptaketide hydrolyase ayg1; AltName: Full=Conidial pigment biosynthesis protein ayg1 | 113.3515503 | 237.4889477 | 1.067555651 | | 1.96518E-06 |
| novel.325 | - | 35.44898131 | 74.39567736 | 1.06687884 | | 0.008201561 |
| Bcin07g02390 | - | 31.2588774 | 65.38803626 | 1.065747378 | | 0.006766283 |
| Bcin06g03840 | - | 46.93449258 | 98.1120721 | 1.065523582 | | 0.001010602 |
| Bcin07g05480 | - | 61.04672101 | 127.4907991 | 1.062976986 | | 0.002442893 |
| Bcin03g00660 | - | 152.0365696 | 317.8396797 | 1.062720323 | | 1.55312E-08 |
| Bcin13g02320 | O94218.1 RecName: Full=Xyloglucan-specific endo-beta-1,4-glucanase A; AltName: Full=Xyloglucanase A; AltName: Full=Xyloglucanendohydrolase A; Flags: Precursor | 7808.422372 | 16299.04211 | 1.061839738 | | 1.8578E-191 |
| Bcin03g00860 | Q4X1Q4.1 RecName: Full=Adenine deaminase; Short=ADE; AltName: Full=Adenine aminohydrolase; Short=AAH | 182.6460936 | 380.9425869 | 1.059769695 | | 2.01784E-10 |
| Bcin11g05540 | D4AUF1.1 RecName: Full=WSC domain-containing protein ARB_07867; Flags: Precursor | 39.12747566 | 81.4906415 | 1.058189484 | | 0.01100198 |
| Bcin02g02740 | Q04013.1 RecName: Full=Citrate/oxoglutarate carrier protein; Short=Coc1p; AltName: Full=Mitochondrial DNA replication protein YHM2 | 3818.879224 | 7938.25828 | 1.055722067 | | 1.2262E-141 |
| Bcin01g05880 | Q9Y7K4.1 RecName: Full=Uncharacterized protein C2A9.02 | 79.84216695 | 165.5631883 | 1.053624821 | | 6.38443E-05 |
| Bcin06g01490 | P0CU11.1 RecName: Full=Uncharacterized transporter SPBPB2B2.16c >P0CU10.1 RecName: Full=Uncharacterized transporter SPAC750.02c | 544.2883057 | 1129.146929 | 1.053024296 | | 1.98858E-33 |
| Bcin05g00100 | - | 45.21645039 | 93.78985878 | 1.052764838 | | 0.002449509 |
| Bcin02g01650 | P53048.1 RecName: Full=General alpha-glucoside permease; AltName: Full=Maltose permease MAL11; AltName: Full=Maltose transport protein MAL11 | 444.898049 | 922.6133815 | 1.051839702 | | 8.04615E-19 |
| Bcin13g03080 | Q50EK3.1 RecName: Full=Cytochrome P450 704C1; AltName: Full=Cytochrome P450 CYPD [Pinus taeda] | 51.3400087 | 106.3792448 | 1.05123062 | | 0.001406627 |
| novel.565 | - | 23.6834377 | 48.9502391 | 1.049677816 | | 0.038376353 |
| Bcin09g05570 | O04036.3 RecName: Full=Sugar transporter ERD6; AltName: Full=Early-responsive to dehydration protein 6; AltName: Full=Sugar transporter-like protein 1 | 2560.464565 | 5300.109308 | 1.049481178 | | 1.3869E-123 |
| novel.628 | - | 36.69849866 | 75.88007916 | 1.049368154 | | 0.008793396 |
| Bcin09g02930 | P11838.2 RecName: Full=Endothiapepsin; AltName: Full=Aspartate protease; Flags: Precursor | 14161.29377 | 29235.6861 | 1.04580636 | | 0 |
| Bcin01g04540 | O74631.1 RecName: Full=Protein FDD123; AltName: Full=CvHSP30/1 | 97.15659635 | 200.6359764 | 1.045169363 | | 6.57518E-06 |
| Bcin06g04350 | - | 52.36871683 | 107.7713135 | 1.042763788 | | 0.000688926 |
| Bcin16g01470 | P24657.1 RecName: Full=Ribonuclease Trv; Short=RNase Trv | 640.6155324 | 1318.895271 | 1.041707527 | | 1.14E-33 |
| Bcin04g00820 | - | 212.4731192 | 437.2565233 | 1.041085422 | | 2.29898E-12 |
| novel.1005 | - | 39.19669486 | 80.62195669 | 1.039477022 | | 0.009983605 |
| Bcin14g03030 | - | 120.9854545 | 248.3235794 | 1.038405611 | | 5.40492E-07 |
| Bcin09g06880 | D4B1N9.1 RecName: Full=Probable secreted lipase ARB_02369; Flags: Precursor | 1035.603957 | 2125.893375 | 1.037591491 | | 6.59813E-58 |
| novel.45 | - | 38.66736123 | 79.17817942 | 1.035341594 | | 0.005715299 |
| novel.1370 | - | 35.15125093 | 71.96978909 | 1.03518001 | | 0.008967579 |
| Bcin07g07000 | - | 96.05328532 | 196.7907463 | 1.034488655 | | 1.6745E-06 |
| Bcin16g05110 | - | 32.79459727 | 66.99481992 | 1.031159981 | | 0.008622623 |
| novel.312 | - | 37.36016585 | 76.29743307 | 1.030465144 | | 0.008129356 |
| Bcin01g00800 | Q96WM9.1 RecName: Full=Laccase-2; AltName: Full=Benzenediol:oxygen oxidoreductase 2; AltName: Full=Diphenol oxidase 2; AltName: Full=Urishiol oxidase 2; Flags: Precursor | 78.96741914 | 160.8587557 | 1.027042189 | | 0.000382806 |
| Bcin01g03670 | - | 87.43530406 | 177.9963353 | 1.02513497 | | 2.1564E-05 |
| Bcin15g05490 | - | 95.37697282 | 194.1913964 | 1.025104999 | | 1.92603E-06 |
| Bcin01g05300 | Q9L9F0.1 RecName: Full=Decarboxylase NovR; AltName: Full=Novobiocin biosynthesis protein R | 243.3019321 | 495.2293165 | 1.024740519 | | 1.97415E-13 |
| Bcin03g08070 | P27747.3 RecName: Full=Dihydrolipoyllysine-residue acetyltransferase component of acetoin cleaving system; AltName: Full=Acetoin dehydrogenase E2 component; AltName: Full=Dihydrolipoamide acetyltransferase component of acetoin cleaving system; AltNam | 280.162776 | 569.8634548 | 1.024689915 | | 8.63949E-17 |
| Bcin01g02430 | - | 201.3861931 | 409.7304378 | 1.024078503 | | 4.41481E-12 |
| Bcin06g01470 | - | 26.81180894 | 54.41819865 | 1.022473648 | | 0.024758305 |
| Bcin01g02880 | P54202.2 RecName: Full=Alcohol dehydrogenase 2; AltName: Full=Alcohol dehydrogenase II; Short=ADH II | 5182.72618 | 10518.65612 | 1.0212655 | | 6.9826E-185 |
| Bcin09g03230 | - | 226.778412 | 459.7811166 | 1.020034578 | | 1.15374E-09 |
| Bcin05g00010 | Q09923.1 RecName: Full=Aldo-keto reductase yakc [NADP(+)] | 12160.10365 | 24655.42198 | 1.019723419 | | 0 |
| novel.1423 | - | 26.77101646 | 54.21122392 | 1.017855618 | | 0.019478829 |
| Bcin10g03150 | Q15125.3 RecName: Full=3-beta-hydroxysteroid-Delta(8),Delta(7)-isomerase; AltName: Full=Cholestenol Delta-isomerase; AltName: Full=Delta(8)-Delta(7) sterol isomerase; Short=D8-D7 sterol isomerase; AltName: Full=Emopamil-binding protein | 434.5753211 | 879.7779619 | 1.017540047 | | 1.50482E-21 |
| Bcin12g02390 | P18172.4 RecName: Full=Glucose dehydrogenase [FAD, quinone]; Contains: RecName: Full=Glucose dehydrogenase [FAD, quinone] short protein; Flags: Precursor | 108.8382634 | 220.1240511 | 1.017208437 | | 4.21724E-06 |
| Bcin16g02990 | - | 220.9642293 | 446.2987309 | 1.01501515 | | 2.43441E-10 |
| Bcin14g03470 | - | 157.4169938 | 317.8883899 | 1.014697254 | | 6.90813E-08 |
| Bcin08g02100 | Q10DK7.1 RecName: Full=1-aminocyclopropane-1-carboxylate synthase 1; Short=ACC synthase 1; AltName: Full=S-adenosyl-L-methionine methylthioadenosine-lyase 1 >A2XLL2.2 RecName: Full=1-aminocyclopropane-1-carboxylate synthase 1; Short=ACC synthase 1; A | 36.59000669 | 73.90471319 | 1.014197761 | | 0.008969187 |
| Bcin14g05470 | - | 618.1524388 | 1248.245966 | 1.014160491 | | 1.09689E-26 |
| Bcin15g04560 | - | 23.23258428 | 46.85127511 | 1.012179234 | | 0.032258963 |
| novel.1576 | - | 37.35248928 | 75.25893296 | 1.012062884 | | 0.009247038 |
| Bcin06g03010 | - | 302.6944984 | 609.5689687 | 1.009971766 | | 4.31241E-15 |
| Bcin08g00280 | P34946.1 RecName: Full=Carboxypeptidase S1 | 5103.689085 | 10275.60049 | 1.009590557 | | 3.0844E-128 |
| novel.1571 | - | 30.92649808 | 62.16863362 | 1.007177253 | | 0.014782987 |
| Bcin02g03560 | Q9P6J9.1 RecName: Full=Putative inorganic phosphate transporter C1683.01 | 645.3803736 | 1295.125506 | 1.004919293 | | 5.27014E-36 |
| Bcin07g02910 | W7MWX7.1 RecName: Full=Esterase FUS5; AltName: Full=Fusarin biosynthesis protein 5 | 97.85904331 | 196.1882608 | 1.004097311 | | 2.23017E-06 |
| novel.1298 | - | 67.85658667 | 136.0285063 | 1.004028679 | | 0.000742016 |
| Bcin07g05940 | - | 61.22286045 | 122.5173288 | 1.002336659 | | 0.008294295 |
| Bcin07g05140 | O60008.1 RecName: Full=Heat shock protein 60, mitochondrial; AltName: Full=60 kDa chaperonin; AltName: Full=Protein Cpn60; Flags: Precursor | 19973.86261 | 9985.778336 | -1.000093034 | | 3.26E-233 |
| Bcin13g05630 | - | 678.7382583 | 339.2790297 | -1.000206732 | | 3.25E-16 |
| Bcin09g01400 | Q9P3X9.1 RecName: Full=41 kDa peptidyl-prolyl cis-trans isomerase; Short=PPIase; AltName: Full=Cyclophilin-41; Short=CyP41; AltName: Full=Rotamase | 7274.866513 | 3635.65599 | -1.000835017 | | 1.43E-94 |
| Bcin17g00070 | - | 130.0943345 | 64.88487405 | -1.004213699 | | 0.000689132 |
| Bcin09g04430 | P20261.3 RecName: Full=Lipase 1; Flags: Precursor | 410.280153 | 204.2072661 | -1.005808113 | | 1.01E-11 |
| Bcin02g04060 | - | 145.4267243 | 72.44203113 | -1.005912055 | | 0.000853938 |
| Bcin08g01320 | Q55505.1 RecName: Full=Chaperone protein DnaJ 1 | 249.1331564 | 124.0287203 | -1.006712812 | | 5.65E-06 |
| Bcin03g03720 | - | 251.2290075 | 125.0645048 | -1.006788665 | | 1.71E-07 |
| Bcin01g03030 | - | 201.301556 | 100.04497 | -1.008727488 | | 1.92E-06 |
| Bcin16g01770 | B8M9K6.1 RecName: Full=Tropolone cluster transcription factor tropK; AltName: Full=Tropolone synthesis protein K | 588.5330054 | 292.3232234 | -1.009712502 | | 2.84E-15 |
| Bcin09g03190 | O94701.1 RecName: Full=Ingression protein fic1; AltName: Full=Cdc15-interacting C2 domain-containing protein 1 | 251.2328594 | 124.8520394 | -1.010095248 | | 2.93E-06 |
| Bcin12g04620 | - | 1842.014439 | 914.259367 | -1.010364687 | | 2.16E-47 |
| Bcin10g04660 | Q54YH4.1 RecName: Full=Hybrid signal transduction histidine kinase B | 142.3822696 | 70.6104834 | -1.011471436 | | 0.000179837 |
| Bcin04g03420 | G0S9A7.1 RecName: Full=Nucleoporin NUP133; AltName: Full=Nuclear pore protein NUP133 | 373.1585331 | 184.9581191 | -1.01158842 | | 7.32E-10 |
| Bcin13g05640 | Q09752.2 RecName: Full=Multidrug resistance protein fnx1 | 280.1951315 | 139.0705177 | -1.011771821 | | 8.05E-07 |
| Bcin02g06610 | Q5ZI13.1 RecName: Full=DnaJ homolog subfamily C member 3; Flags: Precursor | 1633.709023 | 809.8582038 | -1.012229089 | | 9.28E-43 |
| Bcin01g10600 | P46463.1 RecName: Full=Peroxisome biosynthesis protein PAS1; AltName: Full=Peroxin-1 | 333.6463312 | 165.4092596 | -1.012452777 | | 7.89E-10 |
| Bcin06g05230 | - | 2426.787624 | 1201.418275 | -1.014304414 | | 2.58E-46 |
| Bcin06g00430 | P32386.2 RecName: Full=ATP-dependent bile acid permease | 710.5710217 | 351.2710162 | -1.016763777 | | 1.24E-19 |
| Bcin13g05500 | - | 313.2543953 | 154.7406347 | -1.018389721 | | 1.40E-06 |
| Bcin05g03310 | - | 68.45519153 | 33.75173672 | -1.018628097 | | 0.036460217 |
| Bcin12g06650 | Q4IEV5.1 RecName: Full=RNA exonuclease 4 | 161.7825293 | 79.6817463 | -1.019458774 | | 0.00079897 |
| Bcin07g04700 | P07799.2 RecName: Full=DNA topoisomerase 1; AltName: Full=DNA topoisomerase I | 601.3065203 | 296.4218289 | -1.020964189 | | 3.65E-13 |
| Bcin09g06110 | P28345.2 RecName: Full=Malate synthase, glyoxysomal; AltName: Full=Acetate utilization protein 9 | 21529.11952 | 10607.58623 | -1.021128844 | | 0 |
| Bcin10g05620 | Q5ATC7.1 RecName: Full=Pectate lyase H; Flags: Precursor | 139.1739499 | 68.47685149 | -1.022974529 | | 0.000386077 |
| Bcin15g02590 | Q01513.1 RecName: Full=Adenylate cyclase; AltName: Full=ATP pyrophosphate-lyase; AltName: Full=Adenylyl cyclase | 407.5433588 | 200.6849086 | -1.023192779 | | 2.38E-09 |
| Bcin15g02700 | O13768.1 RecName: Full=Probable DNA repair helicase ercc3 | 122.4003687 | 60.16986427 | -1.023332699 | | 0.000380747 |
| Bcin12g06750 | P46030.1 RecName: Full=Peptide transporter PTR2 | 115.5520421 | 56.80242889 | -1.023507667 | | 0.001040617 |
| Bcin08g03030 | Q75AF5.2 RecName: Full=Golgin IMH1 | 192.0727475 | 94.46548917 | -1.024470712 | | 3.94E-05 |
| Bcin10g03960 | Q9DG67.1 RecName: Full=DNA repair and recombination protein RAD54B; AltName: Full=RAD54 homolog B | 66.7355776 | 32.74974808 | -1.02571718 | | 0.017455113 |
| Bcin09g00980 | P79089.1 RecName: Full=Isocitrate dehydrogenase [NADP], mitochondrial; Short=IDH; AltName: Full=IDP; AltName: Full=NADP(+)-specific ICDH; AltName: Full=Oxalosuccinate decarboxylase; Flags: Precursor | 7201.245075 | 3536.317749 | -1.026133303 | | 3.30E-143 |
| Bcin13g00630 | - | 131.3053256 | 64.3654213 | -1.028113497 | | 0.000222441 |
| Bcin11g04740 | Q10313.2 RecName: Full=tRNA ligase 1 | 129.270244 | 63.41572928 | -1.028389952 | | 0.00031433 |
| Bcin14g05230 | Q9HE11.2 RecName: Full=U3 small nucleolar RNA-associated protein 5; Short=U3 snoRNA-associated protein 5; AltName: Full=U3 protein 5 required for transcription | 482.4630157 | 236.5239993 | -1.028552671 | | 1.28E-13 |
| Bcin13g03260 | Q01389.1 RecName: Full=Serine/threonine-protein kinase BCK1/SLK1/SSP31 | 121.9379614 | 59.66601329 | -1.0292294 | | 0.000734701 |
| Bcin03g02340 | O94300.1 RecName: Full=Putative xanthine/uracil permease C887.17 | 104.1780623 | 51.01799955 | -1.029610025 | | 0.000559971 |
| Bcin07g02750 | Q5ZLG0.1 RecName: Full=Acetoacetyl-CoA synthetase | 668.2497422 | 327.0508918 | -1.030320412 | | 1.00E-18 |
| Bcin15g02360 | - | 92.98260542 | 45.43118388 | -1.032026717 | | 0.002789976 |
| Bcin01g08440 | O94242.1 RecName: Full=Aspartate--tRNA ligase, mitochondrial; AltName: Full=Aspartyl-tRNA synthetase; Short=AspRS; Flags: Precursor | 258.5059049 | 126.3349417 | -1.032754911 | | 6.26E-08 |
| Bcin02g04520 | Q09744.1 RecName: Full=Uncharacterized FAD-binding protein C12C2.03c | 351.5778239 | 171.7596022 | -1.032986868 | | 1.44E-08 |
| Bcin11g00590 | P54789.1 RecName: Full=Origin recognition complex subunit 1 | 106.5001323 | 51.96931311 | -1.034073382 | | 0.0022777 |
| Bcin14g00370 | Q9H5Z1.2 RecName: Full=Probable ATP-dependent RNA helicase DHX35; AltName: Full=DEAH box protein 35 | 75.65667356 | 36.93403985 | -1.034165382 | | 0.005557887 |
| Bcin11g02910 | - | 458.2625708 | 223.2362076 | -1.037339949 | | 3.23E-14 |
| Bcin15g03780 | Q03319.2 RecName: Full=Probable ATP-dependent RNA helicase prh1 | 125.4410501 | 61.03265384 | -1.039808167 | | 0.000482303 |
| Bcin02g01010 | - | 167.6106362 | 81.38195722 | -1.041385679 | | 3.12E-05 |
| Bcin15g04180 | P36616.2 RecName: Full=Protein kinase dsk1; AltName: Full=Dis1-suppressing protein kinase | 39.47675383 | 19.17588007 | -1.041580499 | | 0.047779991 |
| Bcin11g04450 | - | 43.82461795 | 21.28535991 | -1.04176742 | | 0.047989918 |
| Bcin03g03730 | Q9USI6.1 RecName: Full=Myosin type-2 heavy chain 1; AltName: Full=Myosin type II heavy chain 1 | 173.540305 | 84.33204013 | -1.041885245 | | 1.98E-05 |
| Bcin01g01530 | O43071.1 RecName: Full=Pre-mRNA-processing factor 17 | 278.4539822 | 135.1528307 | -1.042242485 | | 7.20E-08 |
| Bcin10g04150 | Q10155.1 RecName: Full=Ribonuclease Z 1; Short=RNase Z 1; AltName: Full=tRNA 3 endonuclease 1; AltName: Full=tRNase Z 1 | 113.7000686 | 55.15121086 | -1.043720047 | | 0.000296006 |
| Bcin11g00280 | - | 125.4679051 | 60.79968349 | -1.043988242 | | 0.000662362 |
| Bcin04g01790 | - | 277.622241 | 134.6883289 | -1.044013078 | | 4.57E-08 |
| Bcin10g02140 | P36619.2 RecName: Full=Leptomycin B resistance protein pmd1 | 2742.100409 | 1324.901671 | -1.049376498 | | 2.56E-44 |
| novel.1050 | - | 59.47711206 | 28.71667077 | -1.049794258 | | 0.014077848 |
| Bcin03g08640 | P08158.1 RecName: Full=Acetamidase | 96.45713738 | 46.56207774 | -1.050605623 | | 0.014739296 |
| Bcin09g00370 | F4HX15.1 RecName: Full=Phospholipase A I; Short=AtPLA1 | 1185.563318 | 572.2663465 | -1.050955061 | | 4.08E-28 |
| Bcin11g00560 | Q9Y7Y1.1 RecName: Full=Protein sgm1 | 158.0247816 | 76.06989266 | -1.054755545 | | 2.95E-05 |
| Bcin04g03370 | - | 342.0166101 | 164.3852064 | -1.055910477 | | 1.45E-08 |
| Bcin14g04610 | A0QZE3.1 RecName: Full=Putative hydrolase MSMEG_3995/MSMEI_3903 | 102.4315413 | 49.24093528 | -1.056175195 | | 0.001017305 |
| Bcin13g00050 | - | 3063.685941 | 1472.654565 | -1.056969326 | | 2.05E-80 |
| Bcin03g04270 | - | 457.1423343 | 219.6275343 | -1.057238644 | | 1.29E-12 |
| Bcin13g05560 | Q9H3U1.1 RecName: Full=Protein unc-45 homolog A; Short=Unc-45A; AltName: Full=GCUNC-45; AltName: Full=Smooth muscle cell-associated protein 1; Short=SMAP-1 | 861.5757543 | 413.7476293 | -1.057682609 | | 1.48E-22 |
| Bcin13g05350 | - | 98.91157972 | 47.46508718 | -1.058140568 | | 0.001364876 |
| Bcin02g00005 | - | 801.8625706 | 384.4125872 | -1.059803331 | | 7.82E-18 |
| Bcin01g09520 | - | 409.4770346 | 196.3854085 | -1.060005806 | | 6.15E-10 |
| Bcin02g07400 | Q01371.2 RecName: Full=White collar 1 protein; Short=WC1 | 378.8988981 | 181.5758314 | -1.060515281 | | 1.47E-10 |
| Bcin04g01050 | P32599.1 RecName: Full=Fimbrin; AltName: Full=ABP67 | 3716.19555 | 1779.816135 | -1.062226616 | | 1.62E-84 |
| novel.397 | - | 3516.42651 | 1682.4389 | -1.063440256 | | 2.32E-11 |
| Bcin15g03220 | O13776.2 RecName: Full=Ubp5-interacting protein ftp105; AltName: Full=Down-regulated in multiple cancers 1 homolog 3; AltName: Full=Hid-1 family protein ftp105 | 716.5692419 | 342.8891412 | -1.063758962 | | 5.67E-20 |
| Bcin05g08130 | - | 2109.222661 | 1008.705447 | -1.064496279 | | 7.05E-43 |
| Bcin08g03490 | Q10347.1 RecName: Full=Uncharacterized protein C1F12.05 | 587.731342 | 280.8799276 | -1.064865313 | | 1.41E-15 |
| Bcin08g04180 | Q4WVH4.1 RecName: Full=Histone-lysine N-methyltransferase, H3 lysine-79 specific; AltName: Full=Histone H3-K79 methyltransferase; Short=H3-K79-HMTase | 1036.077996 | 495.1061846 | -1.065286852 | | 1.50E-27 |
| Bcin09g06960 | - | 144.0672606 | 68.80262098 | -1.065657237 | | 7.88E-05 |
| Bcin13g05460 | P87308.1 RecName: Full=Protein lsb5 | 1086.674689 | 518.8735991 | -1.066202827 | | 3.23E-30 |
| Bcin16g02040 | Q9HE88.1 RecName: Full=Probable exocyst complex component sec8 | 347.9732119 | 166.1446427 | -1.066602534 | | 6.10E-11 |
| Bcin02g07660 | P24686.2 RecName: Full=Negative regulator of mitosis; AltName: Full=Anaphase-promoting complex subunit 1 | 261.1634064 | 124.6474956 | -1.067184104 | | 2.28E-07 |
| Bcin03g01050 | - | 100.928872 | 48.1042061 | -1.068160992 | | 0.002623162 |
| Bcin02g05560 | - | 302.3028433 | 143.9801421 | -1.06907934 | | 5.57E-09 |
| Bcin13g05220 | Q12000.2 RecName: Full=Translation machinery-associated protein 46; AltName: Full=DRG family-regulatory protein 1 | 942.7543014 | 448.6818464 | -1.071920397 | | 2.74E-22 |
| Bcin10g01500 | Q03672.1 RecName: Full=ATP synthase subunit 9, mitochondrial; AltName: Full=Lipid-binding protein; Flags: Precursor | 21492.74957 | 10219.07816 | -1.072634065 | | 7.30E-277 |
| Bcin03g00510 | - | 224.4695716 | 106.6349291 | -1.073946985 | | 1.13E-06 |
| Bcin12g06020 | P49380.1 RecName: Full=Plasma membrane ATPase; AltName: Full=Proton pump | 27841.04974 | 13221.6773 | -1.07424106 | | 2.10E-233 |
| Bcin13g05150 | - | 8419.499513 | 3998.551399 | -1.074323485 | | 3.08E-175 |
| Bcin05g00840 | Q07878.1 RecName: Full=Vacuolar protein sorting-associated protein 13; AltName: Full=Suppression of the onset of impotence protein 1; AltName: Full=Vacuolar protein-targeting protein 2 | 435.1461024 | 206.2004835 | -1.078393418 | | 9.11E-13 |
| novel.526 | - | 72.92688744 | 34.49797975 | -1.07850036 | | 0.003978656 |
| Bcin05g03270 | O13920.3 RecName: Full=LETM1 domain-containing protein mdm28, mitochondrial; Flags: Precursor | 2022.50644 | 957.3897381 | -1.07908673 | | 5.20E-33 |
| Bcin12g01230 | Q9HDX1.1 RecName: Full=Uncharacterized transcriptional regulatory protein PB1A11.04c | 1501.57586 | 709.9520896 | -1.080562971 | | 6.72E-39 |
| Bcin14g01770 | O59700.1 RecName: Full=Uncharacterized transporter C36.03c | 3569.419152 | 1683.195537 | -1.084118561 | | 7.69E-87 |
| Bcin01g00440 | P78615.1 RecName: Full=Fatty acid synthase subunit alpha; Includes: RecName: Full=Acyl carrier; Includes: RecName: Full=3-oxoacyl-[acyl-carrier-protein] reductase; AltName: Full=Beta-ketoacyl reductase; Includes: RecName: Full=3-oxoacyl-[acyl-carrier | 1870.236603 | 882.1496486 | -1.084188856 | | 1.57E-37 |
| Bcin13g05290 | - | 370.3325554 | 174.6016889 | -1.084503913 | | 6.98E-11 |
| Bcin05g00390 | W7MLD3.1 RecName: Full=Efflux pump FUS6; AltName: Full=Fusarin biosynthesis protein 6 | 195.7135275 | 92.24576486 | -1.08543383 | | 2.69E-06 |
| Bcin05g07340 | O13901.1 RecName: Full=Uncharacterized CDP-alcohol phosphatidyltransferase class-I family protein C22A12.10 | 1460.634217 | 687.6684888 | -1.086813301 | | 8.86E-43 |
| Bcin04g06390 | Q9URX1.1 RecName: Full=UNC93-like protein C922.05c | 207.846008 | 97.76486552 | -1.087231619 | | 3.98E-07 |
| Bcin11g01630 | G0S0E7.1 RecName: Full=Nucleoporin NUP120; AltName: Full=Nuclear pore protein NUP120 | 210.2327121 | 98.80099468 | -1.089976985 | | 8.08E-07 |
| Bcin11g01000 | Q9URU2.2 RecName: Full=DNA replication ATP-dependent helicase/nuclease dna2; Includes: RecName: Full=DNA replication nuclease dna2; Includes: RecName: Full=DNA replication ATP-dependent helicase dna2 | 54.99078415 | 25.77825922 | -1.093557354 | | 0.027986652 |
| Bcin14g04130 | - | 715.4182096 | 334.9680217 | -1.095111713 | | 3.08E-15 |
| Bcin08g06930 | O94536.1 RecName: Full=Putative ATP-dependent RNA helicase ucp12 | 195.5295554 | 91.25868924 | -1.098343543 | | 2.04E-06 |
| Bcin01g05890 | Q55DQ2.1 RecName: Full=ABC transporter G family member 11; AltName: Full=ABC transporter ABCG.11 | 11710.23429 | 5464.477757 | -1.099498709 | | 9.73E-238 |
| Bcin13g05250 | Q9H270.1 RecName: Full=Vacuolar protein sorting-associated protein 11 homolog; Short=hVPS11; AltName: Full=RING finger protein 108 | 1266.197087 | 590.2244902 | -1.101142501 | | 4.08E-41 |
| Bcin14g01820 | Q5B367.2 RecName: Full=RNA exonuclease 3 | 63.22558546 | 29.3324484 | -1.10582601 | | 0.02133924 |
| Bcin13g02780 | O94342.1 RecName: Full=Probable metabolite transport protein C1271.09 | 5593.663728 | 2593.141222 | -1.109158774 | | 8.01E-125 |
| Bcin11g01950 | P53326.1 RecName: Full=Uncharacterized protein YGR266W | 333.619386 | 154.5909804 | -1.109650928 | | 1.30E-10 |
| Bcin01g09340 | - | 507.7098789 | 234.9449534 | -1.110870949 | | 9.29E-16 |
| Bcin05g05830 | C5FHK0.1 RecName: Full=Tripeptidyl-peptidase SED1; AltName: Full=Sedolisin-A; Flags: Precursor | 576.2750561 | 266.6412284 | -1.111997414 | | 2.17E-19 |
| novel.1108 | - | 44.57396161 | 20.62439851 | -1.113264934 | | 0.048417589 |
| Bcin10g00540 | Q9Y7M3.1 RecName: Full=FYVE-type zinc finger-containing protein C9B6.03 | 1941.640123 | 897.309736 | -1.113740491 | | 8.46E-52 |
| Bcin11g02120 | Q9P6R0.2 RecName: Full=Transcription factor IIIB 60 kDa subunit; Short=TFIIIB; AltName: Full=B-related factor 1; Short=BRF-1; AltName: Full=TFIIB-related factor | 94.212853 | 43.53913397 | -1.114067947 | | 0.001213228 |
| Bcin02g05080 | O74740.1 RecName: Full=Cleavage factor two protein 2 | 297.7711505 | 137.3403187 | -1.115091777 | | 7.65E-09 |
| Bcin14g05250 | - | 99.98333388 | 46.1127716 | -1.115234557 | | 0.000779014 |
| Bcin09g02070 | P36091.1 RecName: Full=Mannan endo-1,6-alpha-mannosidase DCW1; AltName: Full=Defective cell wall 1; AltName: Full=Endo-alpha-1->6-D-mannanase DCW1; Flags: Precursor | 174.1189722 | 80.26700987 | -1.117457304 | | 8.83E-06 |
| Bcin10g00660 | Q04608.1 RecName: Full=Uncharacterized protein YDR124W | 257.9449485 | 118.9340236 | -1.11766809 | | 4.82E-08 |
| Bcin05g01580 | - | 75.82670816 | 34.90793744 | -1.118526476 | | 0.001939748 |
| Bcin14g04380 | Q9QXE7.2 RecName: Full=F-box-like/WD repeat-containing protein TBL1X; AltName: Full=Transducin beta-like protein 1X | 368.6513897 | 169.4829621 | -1.119999282 | | 2.62E-10 |
| Bcin10g00520 | - | 2053.074255 | 943.9575813 | -1.120319229 | | 8.52E-41 |
| Bcin03g05860 | Q9US06.2 RecName: Full=RNA polymerase II-associated protein 1 homolog | 214.5197281 | 98.62637828 | -1.12255355 | | 5.74E-07 |
| Bcin10g00460 | Q10250.1 RecName: Full=Uncharacterized protein C56F8.02 | 756.7815459 | 347.2694858 | -1.123512431 | | 1.90E-20 |
| Bcin07g04430 | P38093.1 RecName: Full=Developmental regulator flbA | 516.8340987 | 237.2449355 | -1.123867174 | | 2.41E-17 |
| Bcin05g02460 | - | 4643.344848 | 2127.956781 | -1.126019695 | | 7.04E-92 |
| Bcin02g07990 | - | 287.375864 | 131.5149807 | -1.126405666 | | 6.59E-09 |
| Bcin10g05610 | - | 1251.299256 | 572.6981453 | -1.127508101 | | 9.74E-36 |
| Bcin02g05860 | Q07181.1 RecName: Full=Polygalacturonase; Short=PG; AltName: Full=FmPG; AltName: Full=Pectinase; Flags: Precursor | 2710.557084 | 1238.304881 | -1.129769645 | | 1.61E-64 |
| Bcin02g00660 | Q0JL44.1 RecName: Full=Protein SGT1 homolog; Short=OsSGT1; AltName: Full=Suppressor of G2 allele of SKP1 homolog | 2092.454043 | 954.301977 | -1.132682938 | | 8.85E-62 |
| Bcin13g00010 | Q4WMJ0.1 RecName: Full=Cytochrome P450 monooxygenase gliF; AltName: Full=Gliotoxin biosynthesis protein F | 1184.953041 | 539.8818983 | -1.133742956 | | 1.19E-38 |
| Bcin13g01960 | - | 213.9049583 | 97.41869172 | -1.135319653 | | 1.80E-06 |
| Bcin03g09300 | Q9UYV8.1 RecName: Full=Nitrilase; AltName: Full=PaNit | 766.9590093 | 348.9624846 | -1.136084612 | | 1.35E-24 |
| Bcin10g04250 | - | 48.34403575 | 22.0051413 | -1.137443108 | | 0.024837379 |
| Bcin07g06220 | Q5B0C0.1 RecName: Full=Heat shock 70 kDa protein; Flags: Precursor | 24588.85694 | 11131.976 | -1.143218885 | | 0 |
| Bcin13g05260 | Q59QC7.1 RecName: Full=Sterol uptake control protein 2 | 4242.933357 | 1918.68821 | -1.14464906 | | 8.36E-89 |
| Bcin05g01530 | - | 136.2871247 | 61.62658898 | -1.14474335 | | 9.82E-06 |
| Bcin08g03730 | - | 180.5410208 | 81.48636636 | -1.147503484 | | 1.07E-05 |
| Bcin11g06260 | A7E727.1 RecName: Full=Mitochondrial outer membrane protein iml2 | 897.388082 | 405.0465096 | -1.14791415 | | 7.53E-26 |
| Bcin06g03040 | - | 142.4661208 | 64.08680115 | -1.152509229 | | 8.10E-06 |
| Bcin18g00180 | - | 33.56014529 | 15.06682082 | -1.154221959 | | 0.048641088 |
| Bcin13g00560 | - | 481.1566062 | 215.9747713 | -1.155144775 | | 5.74E-15 |
| Bcin11g05840 | - | 8762.734088 | 3932.305297 | -1.155796435 | | 1.93E-157 |
| Bcin09g02510 | - | 77.69017019 | 34.78324599 | -1.157310999 | | 0.002755171 |
| Bcin14g04990 | - | 784.3250525 | 351.4207304 | -1.15767251 | | 1.25E-24 |
| Bcin07g00420 | - | 323.9042627 | 145.1016749 | -1.15821913 | | 3.82E-12 |
| Bcin13g04830 | A1CEK6.1 RecName: Full=Class E vacuolar protein-sorting machinery protein hse1 | 747.2864326 | 334.2265184 | -1.160238226 | | 1.37E-24 |
| Bcin10g01130 | - | 656.103441 | 292.8504157 | -1.164144391 | | 6.47E-15 |
| Bcin18g00110 | - | 79.66060449 | 35.39278291 | -1.167588327 | | 0.004168326 |
| Bcin16g01920 | - | 306.6945387 | 136.4189318 | -1.168981522 | | 1.72E-09 |
| Bcin07g04370 | B8NM69.1 RecName: Full=Peptidase S41 family protein ustP; AltName: Full=Ustiloxin B biosynthesis protein P | 1351.038633 | 600.9564943 | -1.169231258 | | 1.11E-41 |
| Bcin07g04040 | O14031.1 RecName: Full=Glutathione transporter 1 | 64.7620912 | 28.77162099 | -1.169949945 | | 0.003113773 |
| Bcin04g03150 | P28812.2 RecName: Full=Uncharacterized protein PA3568 | 270.7977694 | 120.3603556 | -1.170237432 | | 4.38E-10 |
| Bcin03g07020 | G4N5Q2.2 RecName: Full=C2H2 finger domain transcription factor CON7 | 6167.813692 | 2737.605925 | -1.171767984 | | 3.96E-169 |
| Bcin02g08130 | Q10142.1 RecName: Full=Inositol phosphorylceramide synthase catalytic subunit aur1; Short=IPC synthase catalytic subunit aur1; AltName: Full=Aureobasidin A resistance protein homolog; AltName: Full=Phosphatidylinositol:ceramide phosphoinositol transf | 2049.124058 | 909.2130972 | -1.17228514 | | 3.99E-68 |
| Bcin02g06480 | O94676.1 RecName: Full=U3 small nucleolar RNA-associated protein 22; Short=U3 snoRNA-associated protein 22 | 194.7124989 | 86.29851179 | -1.174008888 | | 1.24E-07 |
| Bcin01g01850 | - | 600.2694018 | 265.9690018 | -1.174607 | | 4.75E-21 |
| Bcin13g05550 | O74366.1 RecName: Full=RNA polymerase II transcription factor B subunit 4; AltName: Full=RNA polymerase II transcription factor B 34 kDa subunit; AltName: Full=RNA polymerase II transcription factor B p34 subunit | 357.6129066 | 158.387845 | -1.175306403 | | 1.41E-12 |
| Bcin03g00450 | Q6FJJ1.1 RecName: Full=Oleate activated transcription factor 3 | 824.6850159 | 365.0771658 | -1.175538296 | | 1.90E-30 |
| Bcin14g05430 | - | 289.8979405 | 128.3555535 | -1.176386162 | | 6.93E-10 |
| Bcin07g06530 | - | 93.89745058 | 41.53955457 | -1.177798255 | | 0.000339983 |
| Bcin13g05360 | Q90597.1 RecName: Full=Interferon-induced GTP-binding protein Mx; AltName: Full=Interferon-inducible Mx protein | 44.42243694 | 19.596759 | -1.181519696 | | 0.024636869 |
| Bcin13g00130 | O74959.1 RecName: Full=Uncharacterized oxidoreductase C736.13 | 165.4617442 | 72.83691373 | -1.181648701 | | 0.000292469 |
| Bcin06g07420 | - | 132.6294326 | 58.24788762 | -1.185061838 | | 0.000125285 |
| Bcin09g03930 | Q5ASN8.1 RecName: Full=Putative peroxiredoxin pmp20; AltName: Full=Peroxisomal membrane protein pmp20; AltName: Full=Thioredoxin reductase | 10456.28902 | 4597.07816 | -1.185634082 | | 7.03E-189 |
| Bcin05g04850 | P22579.2 RecName: Full=Transcriptional regulatory protein SIN3 | 630.1771612 | 276.4186972 | -1.18783017 | | 1.29E-17 |
| Bcin05g05480 | - | 166.7411813 | 73.06804125 | -1.189137167 | | 1.93E-05 |
| Bcin05g06800 | Q1E306.1 RecName: Full=ATP-dependent RNA helicase ROK1 | 152.9390757 | 66.84129588 | -1.192068563 | | 1.47E-05 |
| Bcin06g07150 | D4B1P2.1 RecName: Full=Uncharacterized FAD-linked oxidoreductase ARB_02372; Flags: Precursor | 39.14209496 | 17.08159661 | -1.195265389 | | 0.044490769 |
| Bcin01g10720 | - | 123.8175751 | 54.03083285 | -1.196256673 | | 5.72E-05 |
| Bcin14g03090 | - | 192.7974637 | 84.18273059 | -1.196745149 | | 2.33E-07 |
| Bcin10g05840 | W7N2B4.2 RecName: Full=Efflux pump FUB11; AltName: Full=Fusaric acid biosynthesis protein 11 | 87.39297918 | 38.11208305 | -1.197983306 | | 0.000688682 |
| Bcin01g03810 | - | 98.13066587 | 42.74724042 | -1.198197195 | | 0.002640942 |
| Bcin12g06800 | - | 87.9061738 | 38.23839532 | -1.199250289 | | 0.00056962 |
| Bcin12g05780 | P45816.2 RecName: Full=SEC14 cytosolic factor; AltName: Full=Phosphatidylinositol/phosphatidylcholine transfer protein; Short=PI/PC TP | 603.9800574 | 262.7820788 | -1.199929128 | | 7.50E-20 |
| Bcin13g05800 | - | 60.95819333 | 26.54871568 | -1.201302075 | | 0.013846355 |
| Bcin08g06760 | G0SA56.1 RecName: Full=Protein MLP1 homolog | 308.4433259 | 134.085438 | -1.202453503 | | 2.44E-11 |
| Bcin13g00710 | Q8T675.1 RecName: Full=ABC transporter G family member 19; AltName: Full=ABC transporter ABCG.19 | 12135.8465 | 5267.671654 | -1.20392389 | | 3.88E-297 |
| Bcin13g05510 | Q10234.1 RecName: Full=Probable 37S ribosomal protein S5, mitochondrial | 1022.521146 | 443.4662887 | -1.204667061 | | 1.11E-33 |
| Bcin11g03290 | - | 124.5846295 | 53.83368521 | -1.209360207 | | 0.000479767 |
| Bcin01g09500 | P37297.1 RecName: Full=Phosphatidylinositol 4-kinase STT4; Short=PI4-kinase; Short=PtdIns-4-kinase | 1060.804632 | 457.8253435 | -1.211948006 | | 3.03E-37 |
| Bcin09g06240 | Q12446.1 RecName: Full=Proline-rich protein LAS17 | 860.9887808 | 371.1004615 | -1.21390185 | | 3.22E-31 |
| Bcin04g06710 | P0CD89.1 RecName: Full=Aquaporin-2 | 44.66864616 | 19.25457697 | -1.215238448 | | 0.031206707 |
| Bcin07g04410 | - | 139.2700501 | 59.96953563 | -1.215559169 | | 4.45E-06 |
| Bcin13g04910 | A7F7B2.1 RecName: Full=Autophagy-related protein 13 | 1761.653419 | 756.5777891 | -1.219036216 | | 6.15E-51 |
| Bcin12g03920 | Q2US83.1 RecName: Full=Probable endo-beta-1,4-glucanase D; Short=Endoglucanase D; AltName: Full=Carboxymethylcellulase D; AltName: Full=Cellulase D; Flags: Precursor >B8MXJ7.1 RecName: Full=Probable endo-beta-1,4-glucanase D; Short=Endoglucanase D; A | 56.11180591 | 24.03823382 | -1.223945325 | | 0.004644732 |
| Bcin02g02730 | P21657.3 RecName: Full=Transcriptional activator protein DAL81; AltName: Full=Regulatory protein UGA35 | 35.24357788 | 15.06058011 | -1.224884705 | | 0.047678467 |
| Bcin01g02190 | Q5B3U2.1 RecName: Full=Pre-mRNA-splicing factor slu7; AltName: Full=Splicing factor sluA | 253.2825078 | 108.2039238 | -1.225387073 | | 1.20E-08 |
| Bcin02g07860 | Q10146.2 RecName: Full=Exosome complex exonuclease rrp6; AltName: Full=Ribosomal RNA-processing protein 6 | 45.5611041 | 19.47674809 | -1.226315316 | | 0.022685421 |
| Bcin05g00530 | Q9Y7D1.1 RecName: Full=Acyltransferase LovD; AltName: Full=Lovastatin hydrolase; AltName: Full=Simvastatin synthase LovD; Short=SV synthase | 54.45376063 | 23.16533379 | -1.230369486 | | 0.007715744 |
| Bcin13g02500 | O14296.1 RecName: Full=Vacuolar protein sorting-associated protein 24 | 2708.77281 | 1153.877835 | -1.231434102 | | 2.35E-91 |
| Bcin07g05270 | O74482.1 RecName: Full=Uncharacterized protein C1840.09 | 2587.031107 | 1096.589407 | -1.238799689 | | 1.94E-71 |
| Bcin14g00280 | P41412.1 RecName: Full=Cell division cycle-related protein res2/pct1 | 85.35411155 | 36.0879462 | -1.243586307 | | 0.000371843 |
| Bcin12g05560 | - | 214.5413815 | 90.42554304 | -1.24491316 | | 2.20E-06 |
| Bcin02g05110 | - | 2026.890302 | 854.6746443 | -1.246094872 | | 6.84E-66 |
| Bcin06g05780 | - | 87.54149046 | 36.852972 | -1.246388667 | | 0.000457154 |
| Bcin06g04490 | Q8S4P4.1 RecName: Full=Histone-lysine N-methyltransferase EZ3; AltName: Full=Enhancer of zeste protein 3 | 43.92308845 | 18.47441543 | -1.248894479 | | 0.010890944 |
| Bcin04g02930 | Q8T673.1 RecName: Full=ABC transporter G family member 21; AltName: Full=ABC transporter ABCG.21 | 31.7758973 | 13.32211489 | -1.253199137 | | 0.041441355 |
| Bcin05g02400 | Q9P3B2.1 RecName: Full=Respiratory supercomplex factor 2 homolog C1565.01 | 866.6945622 | 363.2042434 | -1.254987045 | | 1.84E-34 |
| Bcin11g05810 | O14234.1 RecName: Full=Calcium-channel protein cch1 | 328.2513016 | 137.6114834 | -1.255214764 | | 1.14E-11 |
| Bcin09g01260 | P53064.2 RecName: Full=RNA polymerase-associated protein RTF1 | 411.0903575 | 172.0416261 | -1.25693578 | | 8.64E-17 |
| Bcin01g06950 | - | 116.2013823 | 48.37733566 | -1.265111996 | | 1.54E-05 |
| Bcin13g03990 | Q00341.2 RecName: Full=Vigilin; AltName: Full=High density lipoprotein-binding protein; Short=HDL-binding protein | 5182.383578 | 2147.96601 | -1.270588563 | | 3.49E-135 |
| Bcin12g04510 | Q5A599.2 RecName: Full=Histidine protein kinase NIK1 | 368.4536622 | 152.7304781 | -1.27143681 | | 4.05E-15 |
| Bcin14g04550 | O14111.2 RecName: Full=Phosphatidylserine decarboxylase proenzyme 3; Contains: RecName: Full=Phosphatidylserine decarboxylase 3 beta chain; Contains: RecName: Full=Phosphatidylserine decarboxylase 3 alpha chain | 387.7607704 | 160.6153702 | -1.27225087 | | 9.05E-14 |
| Bcin07g01290 | O74225.1 RecName: Full=Heat shock protein hsp88 | 11905.64296 | 4919.026832 | -1.275097129 | | 1.19E-281 |
| Bcin09g06800 | - | 4876.877383 | 2014.534462 | -1.275820935 | | 2.43E-145 |
| Bcin07g01640 | - | 66.63785364 | 27.55220313 | -1.277413397 | | 0.002952624 |
| Bcin09g01440 | Q54Y08.1 RecName: Full=Probable Ras GTPase-activating-like protein ngap; Short=DdNGAP | 404.7291056 | 166.9126669 | -1.277587556 | | 1.03E-10 |
| Bcin11g03840 | P53746.1 RecName: Full=Ferric reductase transmembrane component 4; AltName: Full=Ferric-chelate reductase 4; Flags: Precursor | 159.4289538 | 65.74535331 | -1.277932006 | | 1.23E-05 |
| novel.1314 | - | 64.47664791 | 26.5078677 | -1.279772256 | | 0.009724264 |
| Bcin08g00160 | P53338.1 RecName: Full=Maltose fermentation regulatory protein MAL13 | 1495.556942 | 614.3845785 | -1.283928402 | | 1.23E-54 |
| Bcin03g02880 | Q8U484.1 RecName: Full=Argininosuccinate synthase; AltName: Full=Citrulline--aspartate ligase | 153.1283667 | 62.8065371 | -1.288050422 | | 5.93E-05 |
| Bcin01g01760 | A2QUQ2.1 RecName: Full=Endochitinase A; AltName: Full=Chitinase A; Flags: Precursor | 162.1956557 | 66.30808563 | -1.291248704 | | 1.20E-06 |
| Bcin13g04620 | - | 101.6852371 | 41.53331386 | -1.292573846 | | 3.69E-05 |
| Bcin13g00030 | Q4WVZ0.1 RecName: Full=Uncharacterized protein AFUA_5G13800 | 89.36725145 | 36.4083464 | -1.29282783 | | 0.000717369 |
| Bcin13g05070 | - | 158.755694 | 64.78982597 | -1.293060819 | | 1.97E-07 |
| Bcin12g06470 | - | 227.4077358 | 92.55015862 | -1.297185625 | | 7.70E-08 |
| Bcin12g03480 | A6S950.1 RecName: Full=Phosphatidylethanolamine N-methyltransferase; Short=PE methyltransferase; Short=PEAMT; Short=PEMT | 3162.143014 | 1282.806067 | -1.301279288 | | 2.47E-87 |
| Bcin02g03300 | P38811.1 RecName: Full=Transcription-associated protein 1; AltName: Full=p400 kDa component of SAGA | 400.1809864 | 162.2427809 | -1.303807682 | | 2.22E-12 |
| Bcin02g01350 | O94580.1 RecName: Full=Ubiquitin and WLM domain-containing metalloprotease SPCC1442.07c | 728.2685809 | 294.71023 | -1.305058262 | | 3.10E-28 |
| Bcin14g03260 | - | 252.0422907 | 101.7425266 | -1.308903209 | | 2.61E-10 |
| Bcin13g05010 | - | 107.825785 | 43.5375738 | -1.309012577 | | 4.37E-05 |
| Bcin03g06430 | - | 1805.864505 | 728.7430748 | -1.309308365 | | 3.19E-62 |
| Bcin10g02370 | - | 288.1337487 | 116.2466158 | -1.309591907 | | 2.40E-11 |
| Bcin02g04940 | - | 61.15362793 | 24.65944139 | -1.310129317 | | 0.001304999 |
| Bcin04g01080 | P84285.1 RecName: Full=Sterol O-acyltransferase 2; AltName: Full=ASAT; AltName: Full=Sterol-ester synthase | 1307.063539 | 526.5934186 | -1.310956026 | | 7.09E-39 |
| Bcin04g02790 | Q9P793.1 RecName: Full=Chromatin remodeling factor mit1; AltName: Full=Mi2-like interacting with clr3 protein 1; AltName: Full=Snf2/Hdac-containing repressor complex protein mit1; Short=SHREC protein mit1 | 120.6515162 | 48.42454565 | -1.314943068 | | 1.71E-05 |
| Bcin10g02200 | - | 684.7309647 | 274.9378211 | -1.315419162 | | 1.10E-25 |
| Bcin09g06820 | Q12229.1 RecName: Full=UBX domain-containing protein 3 | 287.8574624 | 115.6927785 | -1.316029861 | | 4.88E-11 |
| Bcin16g03650 | O13817.2 RecName: Full=Protein transport protein sec73 | 307.0292369 | 123.1717047 | -1.316915836 | | 7.62E-12 |
| Bcin12g00450 | O13889.1 RecName: Full=Eukaryotic translation initiation factor 2-alpha kinase 1; AltName: Full=Heme-regulated eukaryotic initiation factor eIF-2-alpha kinase; AltName: Full=Heme-regulated inhibitor 1 | 343.0398172 | 137.6209051 | -1.31715676 | | 7.68E-13 |
| Bcin06g00310 | - | 35.91757149 | 14.38950824 | -1.320194017 | | 0.024395723 |
| Bcin16g04050 | O93934.1 RecName: Full=NADP-specific glutamate dehydrogenase; Short=NADP-GDH; AltName: Full=NADP-dependent glutamate dehydrogenase | 1992.802069 | 797.2372709 | -1.321607574 | | 1.36E-76 |
| Bcin02g03240 | Q1MTM9.2 RecName: Full=Uncharacterized transcriptional regulatory protein C1327.01c | 465.2431523 | 186.0905731 | -1.321635045 | | 3.16E-17 |
| Bcin12g03210 | Q7SA85.1 RecName: Full=Lon protease homolog 2, peroxisomal | 3520.355127 | 1405.658305 | -1.324269266 | | 1.67E-137 |
| Bcin13g04920 | A6QP15.1 RecName: Full=Hydroxyacid-oxoacid transhydrogenase, mitochondrial; Short=HOT; AltName: Full=Alcohol dehydrogenase iron-containing protein 1; Short=ADHFe1; Flags: Precursor | 5133.787232 | 2047.924971 | -1.325849981 | | 4.32E-174 |
| Bcin16g03420 | P33303.2 RecName: Full=Succinate/fumarate mitochondrial transporter; AltName: Full=Regulator of acetyl-CoA synthase activity | 6366.964394 | 2537.963143 | -1.326971394 | | 2.53E-228 |
| Bcin01g01170 | - | 86.25121996 | 34.29412537 | -1.331288094 | | 0.000172301 |
| Bcin13g05340 | P40381.1 RecName: Full=Chromatin-associated protein swi6 | 398.0667554 | 157.9684656 | -1.334645453 | | 1.30E-13 |
| Bcin13g00620 | O60059.1 RecName: Full=Pumilio domain-containing protein C56F2.08c | 2261.376316 | 896.5739475 | -1.334992671 | | 1.57E-90 |
| Bcin08g00880 | - | 65.99388449 | 26.00083572 | -1.342944365 | | 0.002636629 |
| Bcin09g02790 | P16928.2 RecName: Full=Acetyl-coenzyme A synthetase; AltName: Full=Acetate--CoA ligase; AltName: Full=Acyl-activating enzyme | 18153.28787 | 7152.401987 | -1.343632799 | | 1.07E-296 |
| Bcin09g06830 | Q0D0A1.2 RecName: Full=PAB-dependent poly(A)-specific ribonuclease subunit pan3; AltName: Full=PAB1P-dependent poly(A)-nuclease; AltName: Full=PAN deadenylation complex subunit 3 | 368.9875023 | 145.1164659 | -1.346355441 | | 3.54E-14 |
| Bcin07g02800 | Q9MBH2.1 RecName: Full=Protein AIG2 B; AltName: Full=Avirulence-induced gene 2 protein B; AltName: Full=Putative gamma-glutamylcyclotransferase | 135.7778074 | 53.28805419 | -1.34772942 | | 1.55E-06 |
| Bcin07g01750 | Q9LQV2.1 RecName: Full=RNA-dependent RNA polymerase 1; Short=AtRDRP1; AltName: Full=RNA-directed RNA polymerase 1 | 67.27719895 | 26.29465226 | -1.354605199 | | 0.001555344 |
| Bcin13g05540 | O65268.3 RecName: Full=Putative threonine aspartase; Short=Taspase-1; Contains: RecName: Full=Putative threonine aspartase subunit alpha; Contains: RecName: Full=Putative threonine aspartase subunit beta; Flags: Precursor | 213.7072435 | 83.48086056 | -1.357923427 | | 1.48E-08 |
| Bcin05g08140 | Q99758.2 RecName: Full=ATP-binding cassette sub-family A member 3; AltName: Full=ABC-C transporter; AltName: Full=ATP-binding cassette transporter 3; Short=ATP-binding cassette 3 | 481.2688702 | 187.3029402 | -1.36166202 | | 1.97E-20 |
| Bcin10g03530 | Q59QC7.1 RecName: Full=Sterol uptake control protein 2 | 6019.034594 | 2340.728136 | -1.36235285 | | 1.03E-197 |
| Bcin01g10730 | - | 197.6554831 | 76.76511659 | -1.364424275 | | 1.22E-08 |
| Bcin05g02190 | - | 557.8157754 | 216.3827634 | -1.366014194 | | 2.66E-20 |
| Bcin07g06160 | A2QCJ2.1 RecName: Full=Lon protease homolog, mitochondrial; Flags: Precursor | 2959.96726 | 1145.502788 | -1.369638097 | | 2.16E-124 |
| Bcin08g06700 | Q9P782.1 RecName: Full=Uncharacterized protein C1711.08 | 8987.449929 | 3477.245045 | -1.370104582 | | 2.46E-263 |
| Bcin16g02970 | Q5F364.1 RecName: Full=Multidrug resistance-associated protein 1; AltName: Full=ATP-binding cassette sub-family C member 1; AltName: Full=Leukotriene C(4) transporter; Short=LTC4 transporter | 76.23911339 | 29.50815894 | -1.370865554 | | 0.000288426 |
| Bcin01g09910 | B6HJU0.1 RecName: Full=Efflux pump roqT; AltName: Full=Roquefortine/meleagrin synthesis protein T | 983.9947364 | 379.5824098 | -1.374194576 | | 2.48E-44 |
| Bcin05g01390 | Q2U5H8.1 RecName: Full=Probable efflux pump kojT; AltName: Full=Kojic acid transporter | 304.2470524 | 117.1374272 | -1.377987691 | | 2.59E-12 |
| Bcin11g03300 | - | 637.2094205 | 244.9334294 | -1.379383822 | | 8.00E-29 |
| Bcin10g01980 | O00093.2 RecName: Full=3-phytase B; AltName: Full=3 phytase B; AltName: Full=Myo-inositol hexakisphosphate phosphohydrolase B; AltName: Full=Myo-inositol-hexaphosphate 3-phosphohydrolase B; Flags: Precursor | 424.6592881 | 162.5686717 | -1.385051546 | | 4.73E-19 |
| Bcin13g05160 | - | 95.02453786 | 36.35258612 | -1.387536595 | | 0.00065837 |
| Bcin06g03990 | Q7SA95.2 RecName: Full=ATP-dependent DNA helicase II subunit 1; AltName: Full=ATP-dependent DNA helicase II subunit Ku70; AltName: Full=Protein mus-51 | 45.56726097 | 17.39650622 | -1.388534883 | | 0.012801973 |
| Bcin06g01830 | Q4WU59.1 RecName: Full=NADPH-dependent diflavin oxidoreductase 1; AltName: Full=NADPH-dependent FMN and FAD-containing oxidoreductase | 221.7358688 | 84.64417341 | -1.389450718 | | 1.84E-11 |
| Bcin09g01120 | - | 37.69333103 | 14.3711915 | -1.389862374 | | 0.016407126 |
| Bcin14g04570 | P22189.1 RecName: Full=Calcium-transporting ATPase 3 | 109.8015122 | 41.6549456 | -1.396886068 | | 2.51E-05 |
| Bcin03g03880 | O94289.2 RecName: Full=Ubiquitin homeostasis protein lub1 | 1898.11617 | 717.7782625 | -1.40353166 | | 3.55E-78 |
| Bcin05g05430 | - | 290.4034458 | 109.5269407 | -1.406361784 | | 9.70E-14 |
| Bcin09g03330 | P87049.3 RecName: Full=G1/S-specific cyclin pas1 | 1679.21414 | 632.9196567 | -1.407846006 | | 5.11E-45 |
| Bcin10g01550 | P46030.1 RecName: Full=Peptide transporter PTR2 | 4418.406462 | 1664.006343 | -1.408785463 | | 8.22E-129 |
| Bcin01g10570 | - | 35.41128028 | 13.33731128 | -1.409454317 | | 0.023307167 |
| Bcin13g05140 | Q9Y758.1 RecName: Full=Cytochrome P450 52A13; AltName: Full=Alkane hydroxylase 2; AltName: Full=Alkane-inducible p450alk 2; AltName: Full=DH-ALK2 [Debaryomyces hansenii] | 84.14382826 | 31.55067317 | -1.411700135 | | 0.002583316 |
| Bcin08g01800 | P27121.1 RecName: Full=Ornithine decarboxylase; Short=ODC | 331.2334538 | 124.3548945 | -1.412542373 | | 3.66E-13 |
| Bcin09g05030 | O43032.1 RecName: Full=Signal recognition particle receptor subunit alpha homolog; Short=SR-alpha; AltName: Full=Docking protein alpha; Short=DP-alpha | 711.7442084 | 267.2160974 | -1.41337563 | | 2.49E-31 |
| Bcin15g03820 | A5IMH8.1 RecName: Full=Alanine--tRNA ligase; AltName: Full=Alanyl-tRNA synthetase; Short=AlaRS | 1242.981362 | 465.3054245 | -1.417103147 | | 1.94E-53 |
| Bcin02g08300 | O14116.2 RecName: Full=Uncharacterized protein C328.01c | 212.974786 | 79.57362948 | -1.422744353 | | 1.08E-08 |
| Bcin14g00210 | P32382.1 RecName: Full=NADH oxidase | 6633.557623 | 2460.27654 | -1.431002884 | | 5.58E-239 |
| Bcin10g05950 | Q9P413.1 RecName: Full=pH-response transcription factor pacC/RIM101 | 2023.818114 | 750.0742058 | -1.431774176 | | 3.06E-80 |
| Bcin14g00200 | Q9P7M9.1 RecName: Full=Probable GTP cyclohydrolase-2; AltName: Full=GTP cyclohydrolase II | 884.2368095 | 326.9215198 | -1.434601645 | | 1.57E-38 |
| Bcin07g03060 | - | 5450.426543 | 2013.288642 | -1.43704012 | | 7.14E-207 |
| Bcin05g01940 | - | 47.22450768 | 17.42337325 | -1.437205272 | | 0.022408823 |
| Bcin16g04070 | Q67ZE1.2 RecName: Full=3beta-hydroxysteroid-dehydrogenase/decarboxylase isoform 2; Short=At3BETAHSD/D2; AltName: Full=4alpha-carboxysterol-C3-dehydrogenase/C4-decarboxylase isoform 1-2; AltName: Full=Reticulon-like protein B19; Short=AtRTNLB19; AltNa | 39.9691856 | 14.71724329 | -1.440196621 | | 0.01735716 |
| Bcin03g04800 | Q1K9C4.1 RecName: Full=Probable E3 ubiquitin protein ligase C167.07c; AltName: Full=HECT-type E3 ubiquitin transferase C167.07c | 867.0232742 | 319.0731399 | -1.442084434 | | 2.05E-31 |
| Bcin13g05570 | - | 54.92152562 | 20.14625978 | -1.445903857 | | 0.002056422 |
| Bcin13g05450 | - | 88.42012828 | 32.39982653 | -1.446501978 | | 0.000111798 |
| Bcin15g05350 | - | 122.8843247 | 44.9347284 | -1.448701322 | | 1.29E-05 |
| Bcin01g06290 | P22152.2 RecName: Full=Nitrate transporter; AltName: Full=Nitrate permease | 208.380752 | 76.18799865 | -1.450057702 | | 1.01E-10 |
| Bcin12g06000 | Q10297.3 RecName: Full=Importin subunit beta-5; AltName: Full=114 kDa karyopherin; AltName: Full=Karyopherin subunit beta-5; AltName: Full=Karyopherin-114 | 2228.070843 | 813.8721558 | -1.453317136 | | 4.36E-87 |
| Bcin07g03210 | - | 28.99764213 | 10.55051886 | -1.45528975 | | 0.036443597 |
| Bcin08g01000 | - | 66.04853642 | 24.10682026 | -1.456180576 | | 0.00117877 |
| Bcin03g05330 | O59905.1 RecName: Full=Pyridoxal 5'-phosphate synthase subunit PDX1; Short=PLP synthase subunit PDX1; AltName: Full=Singlet oxygen resistance protein 1 | 3082.68809 | 1115.731671 | -1.465967553 | | 9.91E-120 |
| Bcin08g02300 | - | 25.82698472 | 9.296433081 | -1.476584817 | | 0.035852401 |
| novel.911 | - | 606.0459615 | 217.9742301 | -1.476732408 | | 6.83E-23 |
| Bcin11g05960 | - | 190.4346925 | 67.38727092 | -1.496187588 | | 6.07E-07 |
| Bcin01g01840 | O74469.1 RecName: Full=Exosome complex protein C1739.07 | 204.5060759 | 72.40667446 | -1.496671586 | | 7.99E-11 |
| Bcin10g00300 | O43109.1 RecName: Full=Heat shock protein 90 homolog; AltName: Full=Suppressor of vegetative incompatibility MOD-E | 78678.07963 | 27877.13325 | -1.496865196 | | 0 |
| Bcin15g01310 | - | 24.15508 | 8.49910959 | -1.503364193 | | 0.044208146 |
| Bcin01g06040 | P53390.1 RecName: Full=Ammonium transporter MEP3 | 455.1679967 | 160.0706106 | -1.508253567 | | 4.79E-23 |
| Bcin13g05210 | Q06686.1 RecName: Full=Copper transport protein CTR3; Short=Copper transporter 3 | 214.3143374 | 75.25234823 | -1.509402201 | | 1.46E-07 |
| Bcin09g02640 | Q2U325.1 RecName: Full=Probable beta-glucosidase G; AltName: Full=Beta-D-glucoside glucohydrolase G; AltName: Full=Cellobiase G; AltName: Full=Gentiobiase G; Flags: Precursor >B8NMR5.1 RecName: Full=Probable beta-glucosidase G; AltName: Full=Beta-D-g | 3027.627521 | 1060.152775 | -1.513696382 | | 5.43E-107 |
| Bcin07g06550 | P55251.1 RecName: Full=3-isopropylmalate dehydratase; AltName: Full=Alpha-IPM isomerase; Short=IPMI; AltName: Full=Isopropylmalate isomerase | 4758.943248 | 1664.756479 | -1.515139508 | | 3.15E-221 |
| Bcin05g00620 | - | 717.6224341 | 251.0135809 | -1.516587846 | | 2.69E-31 |
| Bcin06g04860 | P53322.1 RecName: Full=High-affinity nicotinic acid transporter; AltName: Full=Nicotinic acid permease | 1103.773654 | 385.651012 | -1.517090304 | | 5.57E-49 |
| Bcin03g00760 | - | 465.5540349 | 162.4783042 | -1.518072352 | | 5.51E-25 |
| Bcin15g02000 | - | 75.03578615 | 26.12356161 | -1.523351519 | | 0.000223928 |
| Bcin16g00970 | - | 83.6337771 | 28.99806725 | -1.525592807 | | 8.62E-05 |
| Bcin07g02540 | - | 23.69114029 | 8.21072299 | -1.526748218 | | 0.037586226 |
| Bcin15g04520 | - | 27.82278032 | 9.648725579 | -1.530085424 | | 0.024514104 |
| Bcin03g06610 | - | 326.2047841 | 112.3441259 | -1.537682971 | | 4.29E-19 |
| Bcin11g05020 | - | 51.17923572 | 17.55858055 | -1.54498169 | | 0.002373635 |
| Bcin04g03920 | - | 132.8556261 | 45.43898477 | -1.545926895 | | 3.24E-07 |
| Bcin04g06160 | - | 83.00977163 | 28.40725246 | -1.54682434 | | 1.52E-05 |
| Bcin03g08650 | B8NM76.2 RecName: Full=ustiloxin B cluster transcription factor ustR; AltName: Full=Ustiloxin B biosynthesis protein R | 44.32164755 | 15.18642635 | -1.5481207 | | 0.013607023 |
| Bcin03g06600 | Q01233.2 RecName: Full=Heat shock 70 kDa protein; AltName: Full=HSP70 | 126901.8697 | 43348.89079 | -1.549651299 | | 0 |
| Bcin04g06660 | - | 603.0238021 | 205.7898985 | -1.550976895 | | 1.42E-25 |
| Bcin02g05670 | - | 67.23186067 | 22.92525132 | -1.551447406 | | 0.000176958 |
| Bcin08g06990 | - | 40.4700665 | 13.70127443 | -1.562416962 | | 0.01558613 |
| Bcin01g00610 | - | 180.1009487 | 60.96129179 | -1.5651854 | | 5.04E-09 |
| Bcin16g01080 | P55824.2 RecName: Full=Probable ubiquitin carboxyl-terminal hydrolase FAF; AltName: Full=Protein fat facets; AltName: Full=Ubiquitin thioesterase FAF; AltName: Full=Ubiquitin-specific-processing protease FAF; Short=Deubiquitinating enzyme FAF | 288.2207041 | 97.28984714 | -1.565304936 | | 1.29E-12 |
| Bcin14g00010 | Q6WAU1.1 RecName: Full=(-)-isopiperitenone reductase | 81.721794 | 27.61570364 | -1.568246926 | | 0.000204789 |
| Bcin11g06180 | P53099.1 RecName: Full=Vitamin B6 transporter TPN1; AltName: Full=Transport of pyridoxine protein 1 | 4209.951553 | 1397.182498 | -1.590975505 | | 1.23E-144 |
| Bcin03g08660 | B5DF89.2 RecName: Full=Cullin-3 | 307.7093614 | 101.8972054 | -1.595198191 | | 7.76E-17 |
| Bcin10g04810 | Q2YIJ8.1 RecName: Full=Glucose/galactose transporter >P0C105.1 RecName: Full=Glucose/galactose transporter | 88.63399477 | 29.18163932 | -1.599104512 | | 0.00051823 |
| Bcin10g04970 | - | 731.4491805 | 240.8399719 | -1.602735423 | | 6.30E-43 |
| Bcin15g03000 | - | 168.3308066 | 55.29109869 | -1.607324412 | | 2.08E-10 |
| Bcin09g03840 | - | 114.3855381 | 37.57893345 | -1.609082298 | | 8.54E-06 |
| Bcin12g02770 | P47173.1 RecName: Full=Uncharacterized protein YJR142W | 744.7904766 | 243.4085244 | -1.61401558 | | 4.37E-44 |
| Bcin03g01750 | O14123.1 RecName: Full=Probable Na(+)/H(+) antiporter C3A11.09 | 928.4775406 | 302.1447226 | -1.619660932 | | 8.43E-51 |
| Bcin10g03900 | - | 1169.697677 | 379.8560668 | -1.622499869 | | 2.67E-56 |
| Bcin13g05400 | Q9UTM5.2 RecName: Full=Uncharacterized protein C1420.01c | 903.1769282 | 293.3022148 | -1.622824255 | | 1.55E-53 |
| Bcin10g02440 | Q9HGM9.1 RecName: Full=DnaJ homolog subfamily C member 7 homolog | 7252.270979 | 2351.598063 | -1.624661712 | | 0 |
| Bcin11g01730 | - | 23.28566447 | 7.515093671 | -1.629547123 | | 0.028507876 |
| Bcin03g00460 | Q87GU5.1 RecName: Full=Autoinducer 2 sensor kinase/phosphatase LuxQ | 970.7557621 | 312.5911763 | -1.634421951 | | 7.18E-58 |
| Bcin02g05300 | - | 1057.221477 | 338.8623653 | -1.64174968 | | 2.37E-59 |
| Bcin05g01870 | Q8WZM0.1 RecName: Full=Histone acetyltransferase GCN5 | 152.2289394 | 48.51884432 | -1.648135284 | | 6.31E-10 |
| Bcin01g08840 | O04235.1 RecName: Full=FACT complex subunit SSRP1; AltName: Full=Facilitates chromatin transcription complex subunit SSRP1; AltName: Full=Recombination signal sequence recognition protein 1 | 1235.17445 | 391.4528852 | -1.657226457 | | 7.06E-63 |
| Bcin11g02100 | Q4INS6.1 RecName: Full=Crossover junction endonuclease MUS81 | 106.7532062 | 33.7656563 | -1.66335941 | | 2.99E-06 |
| Bcin14g01410 | Q9URZ3.1 RecName: Full=Probable proline-specific permease put4 | 153.3207099 | 48.39952216 | -1.664348645 | | 5.03E-09 |
| Bcin03g08670 | Q09759.1 RecName: Full=Uncharacterized protein C24H6.02c | 386.4289742 | 120.9137261 | -1.675813258 | | 3.78E-20 |
| Bcin15g05670 | Q01896.1 RecName: Full=Sodium transport ATPase 2 | 90.09361805 | 28.21431932 | -1.676515812 | | 2.99E-05 |
| Bcin12g05980 | - | 260.4948104 | 81.27396169 | -1.6804657 | | 2.63E-13 |
| Bcin12g05550 | P00504.3 RecName: Full=Aspartate aminotransferase, cytoplasmic; Short=cAspAT; AltName: Full=Cysteine aminotransferase, cytoplasmic; AltName: Full=Cysteine transaminase, cytoplasmic; Short=cCAT; AltName: Full=Glutamate oxaloacetate transaminase 1; Alt | 5016.72678 | 1562.830623 | -1.682216014 | | 2.06E-203 |
| Bcin06g06280 | O74752.1 RecName: Full=Mitochondrial protein import protein mas5; Flags: Precursor | 17335.31793 | 5342.375697 | -1.698138028 | | 0 |
| Bcin10g03970 | Q9HGN7.1 RecName: Full=Translocation protein sec63 | 2738.524434 | 842.7550739 | -1.699604195 | | 2.77E-139 |
| Bcin09g00120 | A6S7T2.1 RecName: Full=ATPase get3; AltName: Full=Arsenical pump-driving ATPase; AltName: Full=Arsenite-stimulated ATPase; AltName: Full=Golgi to ER traffic protein 3; AltName: Full=Guided entry of tail-anchored proteins 3 | 6398.598393 | 1966.913318 | -1.702027638 | | 7.98E-304 |
| Bcin16g04120 | O74023.1 RecName: Full=Methylated-DNA--protein-cysteine methyltransferase; AltName: Full=6-O-methylguanine-DNA methyltransferase; Short=MGMT; AltName: Full=O-6-methylguanine-DNA-alkyltransferase; AltName: Full=Pk-MGMT | 209.4086869 | 64.28822393 | -1.705728997 | | 1.03E-12 |
| Bcin11g04460 | Q8T675.1 RecName: Full=ABC transporter G family member 19; AltName: Full=ABC transporter ABCG.19 | 2520.42252 | 770.6731755 | -1.709319982 | | 8.95E-126 |
| Bcin09g01620 | Q4I1Q6.2 RecName: Full=Putative cryptochrome DASH | 133.715806 | 40.85912164 | -1.71187701 | | 1.50E-09 |
| novel.1086 | - | 36.86545452 | 11.19784405 | -1.714711613 | | 0.008254923 |
| Bcin13g01770 | - | 19.02554217 | 5.784023952 | -1.714876739 | | 0.047524616 |
| novel.540 | - | 198.3878105 | 60.11572482 | -1.721324855 | | 1.04E-11 |
| Bcin05g04840 | O94469.1 RecName: Full=Probable urea active transporter 1 | 86.07580167 | 26.05382038 | -1.724277324 | | 6.20E-06 |
| Bcin07g06140 | C8V213.1 RecName: Full=DnaJ homolog 1, mitochondrial; Flags: Precursor | 2258.464274 | 682.9448074 | -1.725918421 | | 6.63E-134 |
| Bcin12g00910 | - | 76.41837028 | 22.87850737 | -1.738141588 | | 1.42E-05 |
| Bcin14g03350 | - | 28.53523483 | 8.543138571 | -1.739492639 | | 0.01192169 |
| Bcin08g05420 | - | 265.0287689 | 79.09243104 | -1.74445126 | | 8.11E-18 |
| Bcin10g05190 | - | 54.99234257 | 16.39261339 | -1.744785815 | | 0.000177088 |
| Bcin03g02870 | D7PHZ1.1 RecName: Full=Oxidoreductase vrtI; AltName: Full=Viridicatumtoxin synthesis protein I | 167.1413382 | 49.57728199 | -1.752289432 | | 2.03E-11 |
| Bcin01g05820 | - | 183.5709602 | 54.07255225 | -1.763065723 | | 1.54E-10 |
| novel.486 | - | 108.8459786 | 32.07162545 | -1.76618757 | | 1.91E-06 |
| Bcin04g01430 | - | 10070.28534 | 2956.622806 | -1.768019073 | | 0 |
| Bcin11g02370 | - | 18.83395886 | 5.538106157 | -1.771270991 | | 0.044021566 |
| Bcin02g01890 | - | 66.44397807 | 19.38204403 | -1.773791164 | | 0.000653118 |
| Bcin02g02420 | P80235.2 RecName: Full=Putative mitochondrial carnitine O-acetyltransferase | 2029.093033 | 589.3190698 | -1.782843165 | | 2.40E-90 |
| Bcin15g01370 | - | 102.9708964 | 29.80902695 | -1.788287772 | | 8.57E-08 |
| novel.782 | - | 28.2836546 | 8.149937446 | -1.790769708 | | 0.01356499 |
| Bcin07g06730 | - | 22.5393469 | 6.506925689 | -1.792982464 | | 0.017327981 |
| Bcin12g00690 | Q9P7T1.1 RecName: Full=Hydroxamate-type ferrichrome siderophore peptide synthetase | 533.0737225 | 153.3096222 | -1.798578423 | | 4.69E-35 |
| novel.720 | - | 23.20565125 | 6.505365512 | -1.834891804 | | 0.012702184 |
| Bcin06g04070 | Q94CA0.1 RecName: Full=Protein LAZ1 homolog 1; AltName: Full=Lazarus1 homolog 1; Flags: Precursor | 87.31529755 | 24.48285943 | -1.837686279 | | 7.82E-06 |
| Bcin11g03050 | Q12691.1 RecName: Full=Sodium transport ATPase 5 | 119.9937523 | 33.5166788 | -1.839276919 | | 6.92E-08 |
| Bcin01g09630 | - | 484.7511834 | 135.3090704 | -1.84244823 | | 7.05E-31 |
| Bcin14g01960 | - | 48.02239843 | 13.35562802 | -1.846361188 | | 0.000240296 |
| Bcin01g01450 | P43071.1 RecName: Full=Multidrug resistance protein CDR1 | 24.79135988 | 6.888050783 | -1.851464488 | | 0.010639998 |
| Bcin04g02890 | - | 1571.14128 | 433.8162863 | -1.856300607 | | 7.05E-109 |
| Bcin01g09230 | Q9R6X3.1 RecName: Full=Cyanobacterial phytochrome B | 348.496428 | 96.13196423 | -1.857196669 | | 3.89E-25 |
| Bcin13g00140 | Q2HEW4.1 RecName: Full=Enoyl reductase CHGG_01240; AltName: Full=Chaetoglobosin biosynthesis protein CHGG_01240 | 201.5555459 | 55.47969603 | -1.863721671 | | 1.55E-10 |
| Bcin02g06140 | Q5M7R9.1 RecName: Full=Grainyhead-like protein 2 homolog; AltName: Full=Transcription factor CP2-like 3 | 2084.689235 | 572.3934082 | -1.865071197 | | 6.30E-147 |
| Bcin12g05130 | - | 122.4442133 | 33.57162902 | -1.867134605 | | 4.55E-10 |
| Bcin04g06880 | Q09766.1 RecName: Full=Uncharacterized membrane protein C24H6.13 | 193.1775389 | 52.97660968 | -1.868984765 | | 2.11E-13 |
| Bcin13g05470 | - | 897.6849479 | 243.7783228 | -1.881193329 | | 3.53E-66 |
| Bcin16g02870 | - | 19.04401276 | 5.187373836 | -1.882161769 | | 0.041342892 |
| Bcin03g00410 | O34703.1 RecName: Full=Uncharacterized ATPase YjoB | 26.52480723 | 7.153845495 | -1.886337362 | | 0.008347433 |
| Bcin15g04190 | Q54IP0.1 RecName: Full=DnaJ homolog subfamily C member 7 homolog | 2460.249695 | 658.628196 | -1.900615505 | | 1.35E-157 |
| Bcin07g05260 | Q4I7S1.1 RecName: Full=Protein AF-9 homolog | 757.5362333 | 202.961326 | -1.900770348 | | 6.00E-53 |
| Bcin12g04580 | Q10451.2 RecName: Full=Pentatricopeptide repeat-containing protein 5, mitochondrial; Flags: Precursor | 4920.614952 | 1317.242858 | -1.901433185 | | 0 |
| Bcin10g05670 | - | 125.7918353 | 33.68227887 | -1.902335892 | | 3.59E-09 |
| Bcin10g03880 | P40467.1 RecName: Full=Activator of stress genes 1 | 609.0543002 | 162.3223493 | -1.90865925 | | 3.53E-41 |
| Bcin05g02980 | - | 45.17180662 | 11.93865714 | -1.917312651 | | 0.00023474 |
| Bcin11g05820 | - | 22.26007379 | 5.808581401 | -1.936991193 | | 0.020589244 |
| Bcin05g01340 | - | 48.06319091 | 12.49277781 | -1.950594539 | | 0.000828713 |
| Bcin02g07460 | P0CH35.1 RecName: Full=Ubiquitin-60S ribosomal protein L40-2; Contains: RecName: Full=Ubiquitin; Contains: RecName: Full=60S ribosomal protein L40-2; AltName: Full=CEP52; Flags: Precursor >P0CH34.1 RecName: Full=Ubiquitin-60S ribosomal protein L40-1; | 17.49060816 | 4.484349016 | -1.967699113 | | 0.048204348 |
| Bcin13g05860 | A1CFL2.1 RecName: Full=Dehydrogenase patE; AltName: Full=Patulin synthesis protein E; Flags: Precursor | 86.00425088 | 21.98953952 | -1.969356505 | | 3.15E-07 |
| Bcin14g04240 | Q03016.1 RecName: Full=GLC7-interacting protein 3 | 1082.139066 | 274.2125484 | -1.979587351 | | 8.53E-72 |
| Bcin15g05040 | - | 35.57826276 | 8.918022956 | -1.995874286 | | 0.006873319 |
| novel.927 | - | 30.24096329 | 7.601591457 | -1.997118569 | | 0.005504763 |
| Bcin12g00760 | O94300.1 RecName: Full=Putative xanthine/uracil permease C887.17 | 459.0995389 | 115.1432777 | -1.997169664 | | 8.77E-28 |
| Bcin11g02420 | - | 413.0460811 | 103.0036032 | -2.005153004 | | 4.03E-32 |
| Bcin10g02530 | L8FSM5.1 RecName: Full=Subtilisin-like protease 2; AltName: Full=Destructin-1; AltName: Full=Serine protease 2; Short=PdSP2; Flags: Precursor | 561.2718761 | 138.2592739 | -2.019414524 | | 9.97E-38 |
| novel.1119 | - | 42.05191052 | 10.34238934 | -2.027354397 | | 0.004496869 |
| novel.901 | - | 29.24306549 | 7.137088932 | -2.030065928 | | 0.003263774 |
| Bcin06g01360 | Q54RJ4.1 RecName: Full=Probable serine/threonine-protein kinase iksA; AltName: Full=Ira1 kinase suppressor protein A | 440.0041097 | 107.4903224 | -2.032634552 | | 5.92E-35 |
| Bcin05g03010 | - | 1762.770668 | 427.8412948 | -2.042654764 | | 2.57E-141 |
| Bcin01g10630 | - | 200.3536996 | 48.18480791 | -2.057679817 | | 1.32E-12 |
| Bcin02g04420 | - | 17.10281707 | 4.066590441 | -2.066808432 | | 0.033124645 |
| Bcin01g03240 | - | 202.2817297 | 48.09980966 | -2.07338277 | | 0.002949635 |
| Bcin16g00830 | O94740.1 RecName: Full=Hsp90 co-chaperone Cdc37; AltName: Full=Cell division control protein 37; AltName: Full=Hsp90 chaperone protein kinase-targeting subunit | 2443.412642 | 580.2058434 | -2.074211327 | | 1.46E-150 |
| Bcin09g05150 | P42502.1 RecName: Full=Arabinose 5-phosphate isomerase KpsF; Short=API; AltName: Full=K-antigen-specific arabinose 5-phosphate isomerase; Short=K-API; AltName: Full=Polysialic acid capsule expression protein kpsF | 1083.565652 | 256.7777879 | -2.076743085 | | 1.11E-78 |
| novel.481 | - | 991.834044 | 233.9475861 | -2.085135485 | | 2.78E-53 |
| Bcin03g09120 | - | 87.1875358 | 20.43817212 | -2.09049927 | | 7.74E-07 |
| Bcin13g05270 | Q8LNW4.1 RecName: Full=Flotillin-like protein 2; AltName: Full=Nodulin-like protein 2 | 312.5242046 | 72.83881865 | -2.100042373 | | 1.29E-25 |
| Bcin05g01970 | - | 92.53557727 | 21.54959445 | -2.101709768 | | 2.49E-08 |
| Bcin10g02660 | - | 17642.09832 | 4098.378412 | -2.105945443 | | 0 |
| Bcin04g02090 | Q9HE05.1 RecName: Full=Ubiquitin conjugation factor E4; AltName: Full=Ubiquitin fusion degradation protein 2; Short=UB fusion protein 2 | 1098.034218 | 254.952765 | -2.107395585 | | 2.15E-70 |
| Bcin09g03120 | - | 31.60742173 | 7.258660022 | -2.127475653 | | 0.002264296 |
| Bcin03g01210 | - | 13.66902556 | 3.126603504 | -2.137760911 | | 0.047434239 |
| Bcin09g07120 | - | 69.70852084 | 15.80647912 | -2.142972304 | | 5.80E-07 |
| Bcin13g05200 | P15315.1 RecName: Full=Transcriptional activator protein CUP2; AltName: Full=Copper-fist transcription factor | 16.55731842 | 3.717418297 | -2.14702567 | | 0.038104292 |
| Bcin11g01680 | - | 1347.225788 | 303.7163723 | -2.149353711 | | 6.01E-89 |
| novel.1025 | - | 22.7040105 | 5.094635341 | -2.149465858 | | 0.008186486 |
| Bcin12g03350 | - | 248.0791136 | 55.88930898 | -2.150791036 | | 1.91E-23 |
| Bcin12g03340 | Q55GW8.2 RecName: Full=AN1-type zinc finger and UBX domain-containing protein DDB_G0268260 | 2259.4377 | 508.0420271 | -2.152598924 | | 4.78E-151 |
| Bcin08g03480 | - | 50.51061248 | 11.27850651 | -2.162112222 | | 0.000179837 |
| Bcin04g05700 | P0CH36.1 RecName: Full=NADP-dependent alcohol dehydrogenase C 1; Short=Ms-ADHC 1 >P0CH37.1 RecName: Full=NADP-dependent alcohol dehydrogenase C 2; Short=Ms-ADHC 2 | 3728.305069 | 831.9415966 | -2.163110026 | | 1.58E-213 |
| Bcin05g03100 | Q9USI5.1 RecName: Full=Heat shock protein sti1 homolog | 15500.7251 | 3454.45498 | -2.1655397 | | 0 |
| Bcin10g00530 | O74465.2 RecName: Full=Helicase required for RNAi-mediated heterochromatin assembly 1 | 46.96451704 | 10.35799111 | -2.184882573 | | 0.000184707 |
| Bcin01g03420 | - | 830.5430686 | 181.2909712 | -2.196170071 | | 1.38E-61 |
| Bcin07g05740 | - | 20.3866036 | 4.43719968 | -2.197321924 | | 0.008565291 |
| Bcin13g05050 | - | 33.16237205 | 7.213070863 | -2.202416681 | | 0.000597941 |
| Bcin14g00870 | O59700.1 RecName: Full=Uncharacterized transporter C36.03c | 3624.881735 | 782.2952621 | -2.211345186 | | 1.01E-247 |
| Bcin03g01870 | - | 20.6258701 | 4.422003294 | -2.217616132 | | 0.008565291 |
| Bcin16g02000 | - | 14.32608161 | 3.05218175 | -2.224184321 | | 0.0386476 |
| Bcin16g04250 | - | 444.2480536 | 94.54783311 | -2.230065591 | | 3.56E-34 |
| Bcin03g07360 | Q8NJK6.1 RecName: Full=Probable pectin lyase F; Short=PLF; Flags: Precursor >A2R6A1.1 RecName: Full=Probable pectin lyase F; Short=PLF; Flags: Precursor | 1458.537555 | 310.415137 | -2.231628538 | | 3.47E-107 |
| Bcin01g00360 | Q9C1W3.1 RecName: Full=Probable squalene monooxygenase; AltName: Full=Squalene epoxidase; Short=SE | 745.5099664 | 157.2140777 | -2.246432906 | | 4.83E-55 |
| Bcin03g02640 | - | 50.91532845 | 10.67989083 | -2.257224621 | | 1.34E-05 |
| Bcin12g00640 | - | 12418.11459 | 2589.552222 | -2.261268191 | | 0 |
| Bcin13g04610 | Q9M8Z7.1 RecName: Full=Sterol 3-beta-glucosyltransferase UGT80A2; AltName: Full=UDP-glucose:sterol glucosyltransferase 80A2 | 548.7915718 | 112.0854433 | -2.290249056 | | 8.24E-42 |
| Bcin01g09530 | P19752.1 RecName: Full=30 kDa heat shock protein | 85888.03274 | 17383.36329 | -2.304754284 | | 6.69E-271 |
| Bcin05g08060 | P27526.2 RecName: Full=Deoxyribodipyrimidine photo-lyase; AltName: Full=DNA photolyase; AltName: Full=Photoreactivating enzyme | 155.8235953 | 31.45896818 | -2.312775769 | | 9.57E-11 |
| Bcin12g06640 | Q7S9B6.1 RecName: Full=Histone acetyltransferase esa-1; AltName: Full=Histone acetyltransferase hat-4 | 346.8998473 | 69.20050264 | -2.325640018 | | 3.58E-33 |
| Bcin16g02770 | A1DA48.1 RecName: Full=Neutral protease 2 homolog NFIA_031120; AltName: Full=Deuterolysin NFIA_031120; Flags: Precursor | 9369.452485 | 1866.775769 | -2.3275568 | | 0 |
| Bcin01g05790 | P22944.2 RecName: Full=Nitrite reductase [NAD(P)H] | 371.9798847 | 74.09053419 | -2.328676741 | | 3.04E-32 |
| Bcin08g03080 | Q9UUD1.1 RecName: Full=Sterol regulatory element-binding protein 1; Contains: RecName: Full=Processed sterol regulatory element-binding protein 1 | 184.4911116 | 36.72313399 | -2.329047036 | | 0.000679539 |
| Bcin14g01400 | Q6ZFZ4.1 RecName: Full=Calpain-type cysteine protease ADL1; AltName: Full=Phytocalpain ADL1; AltName: Full=Protein ADAXIALIZED LEAF1; AltName: Full=Protein DEFECTIVE KERNEL 1; Short=OsDEK1; AltName: Full=Protein SHOOTLESS 3; Flags: Precursor | 33.44549645 | 6.426263226 | -2.372067361 | | 0.001343692 |
| Bcin12g06230 | Q2H0G2.2 RecName: Full=Dicer-like protein 1; Includes: RecName: Full=Endoribonuclease DCL1; Includes: RecName: Full=ATP-dependent helicase DCL1 | 120.7431226 | 23.18677088 | -2.377222124 | |  |
| Bcin08g00020 | - | 27.20266546 | 5.223601931 | -2.38884608 | | 0.007603494 |
| Bcin15g01960 | - | 141.9328966 | 26.91429965 | -2.39533476 | | 1.39E-13 |
| novel.1380 | - | 19.76415716 | 3.714297943 | -2.40197377 | | 0.010490685 |
| Bcin10g03870 | Q9ST27.1 RecName: Full=Phototropin-2; AltName: Full=Non-phototropic hypocotyl protein 1B; Short=OsNPH1B | 193.621475 | 36.52292737 | -2.404906292 | | 2.32E-14 |
| Bcin14g04530 | Q9P6N2.1 RecName: Full=Pdp3-interacting factor 1 | 9771.384758 | 1832.416483 | -2.414691245 | | 0 |
| Bcin08g02190 | P53199.1 RecName: Full=Sterol-4-alpha-carboxylate 3-dehydrogenase, decarboxylating | 41.95419987 | 7.79608477 | -2.421367721 | | 9.75E-05 |
| Bcin06g03510 | - | 266.3929744 | 49.57531643 | -2.424182917 | | 8.89E-27 |
| Bcin04g05120 | - | 459.6713414 | 83.19755988 | -2.468204312 | | 2.01E-41 |
| Bcin13g04780 | - | 183.2239096 | 32.80666387 | -2.480053841 | | 1.30E-19 |
| Bcin08g06640 | P51979.3 RecName: Full=ATP-dependent DNA helicase MER3; AltName: Full=Protein HFM1 | 30.99959459 | 5.422775776 | -2.507247857 | | 0.000727328 |
| Bcin07g02790 | A0A0D2YG01.1 RecName: Full=Non-canonical non-ribosomal peptide synthetase FUB8; AltName: Full=Fusaric acid biosynthesis protein 8 | 3735.211009 | 600.7796889 | -2.635785584 | | 0 |
| Bcin03g05820 | A2QV36.1 RecName: Full=Probable pectate lyase A; Flags: Precursor | 4109.909769 | 658.3027092 | -2.641545163 | | 0 |
| Bcin01g03760 | - | 72.88305555 | 11.70481538 | -2.642158463 | | 1.44E-08 |
| Bcin13g05880 | - | 58.07068633 | 9.183817668 | -2.655638016 | | 1.98E-06 |
| Bcin10g04730 | - | 10.89999269 | 1.706917656 | -2.671178361 | | 0.049677316 |
| Bcin02g07090 | P32382.1 RecName: Full=NADH oxidase | 1046.786523 | 162.275666 | -2.689023874 | | 2.55E-96 |
| Bcin11g00010 | Q5N863.1 RecName: Full=Beta-glucosidase 4; Short=Os1bglu4 | 79.97142177 | 12.22704374 | -2.703412552 | | 6.21E-09 |
| Bcin08g02540 | Q1DKE7.1 RecName: Full=DNA ligase 4; AltName: Full=DNA ligase IV; AltName: Full=Polydeoxyribonucleotide synthase [ATP] 4 | 33.93253121 | 5.184253481 | -2.71609604 | | 0.000119667 |
| Bcin13g04470 | Q59VM4.1 RecName: Full=Transcriptional regulator RPN4 | 492.007396 | 72.93935803 | -2.753518541 | | 8.32E-59 |
| Bcin03g02860 | Q09912.2 RecName: Full=Protein psi1; AltName: Full=Protein psi | 8888.118873 | 1317.138854 | -2.754273002 | | 0 |
| Bcin14g04470 | Q9P768.1 RecName: Full=Uncharacterized amino-acid permease P7G5.06 | 213.2248858 | 30.4890545 | -2.805879894 | | 2.48E-27 |
| novel.606 | - | 11.99560113 | 1.706917656 | -2.812010395 | | 0.039148533 |
| Bcin07g06440 | P25351.2 RecName: Full=Uncharacterized membrane protein YCR023C | 2197.379131 | 307.1512386 | -2.838415716 | | 1.63E-268 |
| Bcin12g06660 | O53732.3 RecName: Full=Tuberculostearic acid methyltransferase UfaA1; Short=TSA methyltransferase | 2823.495366 | 391.8441215 | -2.848969959 | | 0 |
| novel.876 | - | 20.33504312 | 2.759114619 | -2.887787822 | | 0.001643586 |
| Bcin05g02530 | P78581.1 RecName: Full=Tannase; Contains: RecName: Full=Tannase 33 kDa subunit; Contains: RecName: Full=Tannase 30 kDa subunit; Flags: Precursor | 12.97502833 | 1.703797301 | -2.925788828 | | 0.013712684 |
| Bcin13g05870 | A1CFL2.1 RecName: Full=Dehydrogenase patE; AltName: Full=Patulin synthesis protein E; Flags: Precursor | 16.17647006 | 2.037773059 | -2.982829793 | | 0.008116663 |
| Bcin06g00120 | Q9FG72.1 RecName: Full=Oligopeptide transporter 1; Short=AtOPT1 | 297.9888543 | 37.07467709 | -3.008699831 | | 7.45E-38 |
| Bcin03g09310 | - | 334.3279886 | 39.86852027 | -3.06441893 | | 1.61E-35 |
| Bcin02g09270 | O74957.1 RecName: Full=Protein argonaute; AltName: Full=Cell cycle control protein ago1; AltName: Full=Eukaryotic translation initiation factor 2C 2-like protein ago1; AltName: Full=PAZ Piwi domain protein ago1; AltName: Full=Protein slicer; AltName: | 11.70707972 | 1.395533785 | -3.084522416 | | 0.022862912 |
| Bcin11g03580 | Q9C0V1.1 RecName: Full=Ammonium transporter 1 | 361.5322391 | 40.03764608 | -3.173737715 | | 9.64E-52 |
| Bcin02g07510 | - | 1461.374168 | 159.2627106 | -3.199497941 | | 4.00E-180 |
| Bcin07g05710 | Q54DY9.1 RecName: Full=Probable mitochondrial chaperone BCS1-B; AltName: Full=BCS1-like protein 2 | 12.5718714 | 1.354625158 | -3.204082506 | | 0.028912316 |
| Bcin07g01270 | P36842.2 RecName: Full=Nitrate reductase [NADPH]; Short=NR | 236.7452183 | 25.68396055 | -3.204293587 | | 5.01E-29 |
| Bcin02g06220 | - | 178.9238593 | 19.24244029 | -3.218704084 | | 0.012480846 |
| Bcin09g01070 | Q10085.1 RecName: Full=Uncharacterized transporter C11D3.06 | 75.83286502 | 8.060258659 | -3.238307279 | | 0.010999375 |
| Bcin07g04510 | - | 76.46146833 | 7.836993397 | -3.281607218 | | 1.60E-11 |
| Bcin18g00020 | Q9FEW9.1 RecName: Full=12-oxophytodienoate reductase 3; AltName: Full=12-oxophytodienoate-10,11-reductase 3; Short=OPDA-reductase 3; AltName: Full=LeOPR3 | 10.27909196 | 1.056877495 | -3.303584519 | | 0.033324781 |
| Bcin16g02010 | Q4I624.1 RecName: Full=Hsp70 nucleotide exchange factor FES1 | 6472.987068 | 655.4331682 | -3.304010567 | | 0 |
| Bcin09g01190 | Q70J59.1 RecName: Full=Tripeptidyl-peptidase sed2; AltName: Full=Sedolisin-B; Flags: Precursor | 1643.155784 | 164.8213218 | -3.318166908 | | 1.23E-20 |
| Bcin02g07470 | - | 2827.464712 | 276.1746236 | -3.356083458 | | 0 |
| Bcin12g02780 | - | 2252.599122 | 217.2690163 | -3.373021177 | | 7.65E-297 |
| Bcin11g01720 | - | 1033.230379 | 96.65060621 | -3.418664586 | | 4.91E-141 |
| Bcin02g07440 | Q7Z9I0.2 RecName: Full=Uncharacterized MFS-type transporter SPBC409.08 | 3028.811554 | 282.8691132 | -3.419256623 | | 0 |
| Bcin02g07490 | B8NM67.1 RecName: Full=Oxidase ustYa; AltName: Full=Ustiloxin B biosynthesis protein Ya | 1337.869021 | 122.6433577 | -3.445182396 | | 3.24E-174 |
| Bcin06g06720 | - | 1945.856153 | 176.5902864 | -3.462737117 | | 5.22E-274 |
| Bcin02g07480 | Q8L970.1 RecName: Full=Probable prolyl 4-hydroxylase 7; Short=AtP4H7 | 639.9484682 | 56.96253765 | -3.491369597 | | 8.83E-91 |
| Bcin02g07500 | - | 832.9028657 | 71.23481132 | -3.547442774 | | 2.07E-119 |
| Bcin02g04800 | B6V865.1 RecName: Full=Metallocarboxypeptidase A; Short=MCPA; AltName: Full=Carboxypeptidase M14A; Flags: Precursor | 1106.941324 | 94.00139203 | -3.559178218 | | 1.67E-169 |
| Bcin10g03910 | P31540.2 RecName: Full=Heat shock protein hsp98; AltName: Full=Protein aggregation-remodeling factor hsp98 | 23559.22558 | 1839.404101 | -3.678686439 | | 0 |
| Bcin12g04330 | - | 71.78049106 | 5.454728726 | -3.71397435 | | 2.75E-11 |
| Bcin11g04410 | O74402.1 RecName: Full=Heat shock protein 78, mitochondrial; Flags: Precursor | 5516.18635 | 405.8010802 | -3.764542711 | | 0 |
| Bcin06g04920 | - | 9.2950276 | 0.675752402 | -3.76708352 | | 0.024550443 |
| Bcin05g01510 | - | 257.6672737 | 17.81541959 | -3.854428319 | | 1.05E-43 |
| Bcin12g06300 | A6SBW7.1 RecName: Full=Neutral protease 2 homolog SNOG_10522; AltName: Full=Deuterolysin SNOG_10522; Flags: Precursor | 5414.719813 | 322.0205677 | -4.070672819 | | 0 |
| Bcin07g01720 | D4D5P5.1 RecName: Full=Probable dipeptidyl-peptidase 5; AltName: Full=Dipeptidyl-peptidase V; Short=DPP V; Short=DppV; Flags: Precursor | 882.8634217 | 51.31141142 | -4.109792071 | | 4.11E-125 |
| novel.1397 | - | 278.2816553 | 15.91129365 | -4.13231353 | | 6.33E-14 |
| Bcin01g04140 | - | 6.649852533 | 0.338656289 | -4.175564068 | | 0.035497145 |
| Bcin06g00130 | Q9T095.1 RecName: Full=Oligopeptide transporter 6; Short=AtOPT6 | 280.5259131 | 14.75543695 | -4.25092852 | | 1.20E-47 |
| Bcin13g03270 | - | 7.043800489 | 0.338656289 | -4.256778353 | | 0.036316927 |
| Bcin13g01270 | Q01371.2 RecName: Full=White collar 1 protein; Short=WC1 | 973.6933485 | 49.18252069 | -4.304115969 | | 9.78E-155 |
| Bcin05g05530 | Q00858.1 RecName: Full=Cutinase gene palindrome-binding protein; Short=PBP | 798.1256651 | 39.96987043 | -4.316806297 | | 6.46E-116 |
| novel.560 | - | 8.594113652 | 0.352292498 | -4.544679372 | | 0.011453919 |
| Bcin13g02960 | P19222.1 RecName: Full=Carboxypeptidase A2; Flags: Precursor | 923.97358 | 37.97104043 | -4.60327289 | | 1.49E-156 |
| Bcin06g02060 | - | 9.220411389 | 0.337096112 | -4.646367309 | | 0.010524913 |
| Bcin02g08360 | Q09033.1 RecName: Full=Frequency clock protein [Sordaria fimicola] | 1859.536486 | 72.45070344 | -4.679211121 | | 1.77E-304 |
| Bcin11g02900 | P35049.1 RecName: Full=Trypsin; Flags: Precursor | 8316.582069 | 205.4288723 | -5.340616713 | | 0 |
| Bcin05g05270 | - | 10.15443497 | 0 | -5.747648052 | | 0.000944232 |
| Bcin08g06980 | - | 12.07024335 | 0 | -5.99683155 | | 0.000395848 |
| Bcin01g01260 | - | 376.7514738 | 0 | -10.96049571 | | 4.41E-19 |
