# Supplementary material for: The GATA transcription factor BcWCL2 regulates citric acid secretion to maintain redox homeostasis and full virulence in Botrytis cinerea
Source: mBio. 2024 May 30;15(7):e00133-24. doi: 10.1128/mbio.00133-24 (PMC11253612; doi:10.1128/mbio.00133-24)
Supplement: Table S4 — B. cinerea genes differentially expressed in Δbcwcl2+CA compared to Δbcwcl2. [file mbio.00133-24-s0005.docx]

**Table S4: *B. cinerea* genes differentially expressed in Δ*bcwcl2*+CA compared to Δ*bcwcl2*.**

| **Feature ID (fungi.ensembl.org)** | **Putative Function** | **Δ*bcwcl2* mean FPKM 48h in planta** | **Δ*bcwcl2* +CA mean FPKM 48h in planta** | **log2FoldChange:Δ*bcwcl2*+CA vs Δ*bcwcl2*** | **padj adjusted for multiple testing with the Benjamini-Hochberg procedure** | |
| --- | --- | --- | --- | --- | --- | --- |
| Bcin01g08120 | - | 0 | 13.30014891 | 6.154874862 | | 0.000176372 |
| Bcin13g02960 | P19222.1 RecName: Full=Carboxypeptidase A2; Flags: Precursor | 37.46990722 | 2369.826986 | 5.981239957 | | 0 |
| Bcin07g01720 | D4D5P5.1 RecName: Full=Probable dipeptidyl-peptidase 5; AltName: Full=Dipeptidyl-peptidase V; Short=DPP V; Short=DppV; Flags: Precursor | 50.63945204 | 1845.597494 | 5.190232284 | | 7.1485E-81 |
| Bcin11g02900 | P35049.1 RecName: Full=Trypsin; Flags: Precursor | 202.7181198 | 5640.485666 | 4.79960851 | | 0 |
| Bcin09g01190 | Q70J59.1 RecName: Full=Tripeptidyl-peptidase sed2; AltName: Full=Sedolisin-B; Flags: Precursor | 162.657157 | 3801.376641 | 4.547241816 | | 5.83077E-35 |
| Bcin12g06300 | A6SBW7.1 RecName: Full=Neutral protease 2 homolog SNOG_10522; AltName: Full=Deuterolysin SNOG_10522; Flags: Precursor | 317.7702621 | 6120.891065 | 4.266672236 | | 0 |
| Bcin06g04920 | - | 0.666717832 | 12.51001801 | 4.21604423 | | 0.006042534 |
| Bcin02g04800 | B6V865.1 RecName: Full=Metallocarboxypeptidase A; Short=MCPA; AltName: Full=Carboxypeptidase M14A; Flags: Precursor | 92.76239665 | 1590.518293 | 4.101496539 | | 3.9526E-261 |
| Bcin15g05670 | Q01896.1 RecName: Full=Sodium transport ATPase 2 | 27.84400065 | 403.6991813 | 3.862362915 | | 1.94159E-65 |
| Bcin09g04520 | Q9FKE7.2 RecName: Full=Putative flavin-containing monooxygenase 2 | 0.668438745 | 8.96642768 | 3.736136442 | | 0.030789695 |
| novel.1162 | - | 1.014484384 | 12.53292265 | 3.628290315 | | 0.007987533 |
| Bcin14g04570 | P22189.1 RecName: Full=Calcium-transporting ATPase 3 | 41.10373774 | 479.2316757 | 3.540232196 | | 6.81149E-72 |
| Bcin06g00130 | Q9T095.1 RecName: Full=Oligopeptide transporter 6; Short=AtOPT6 | 14.56065328 | 169.2098628 | 3.539962922 | | 4.00137E-26 |
| Bcin10g02530 | L8FSM5.1 RecName: Full=Subtilisin-like protease 2; AltName: Full=Destructin-1; AltName: Full=Serine protease 2; Short=PdSP2; Flags: Precursor | 136.4281124 | 1498.818653 | 3.454754659 | | 4.9427E-188 |
| Bcin09g01070 | Q10085.1 RecName: Full=Uncharacterized transporter C11D3.06 | 7.956268246 | 85.06884513 | 3.422885174 | | 0.008282055 |
| Bcin11g03580 | Q9C0V1.1 RecName: Full=Ammonium transporter 1 | 39.50898134 | 417.8821729 | 3.401891741 | | 7.32342E-66 |
| Bcin06g05050 | P14804.3 RecName: Full=Glucoamylase; AltName: Full=1,4-alpha-D-glucan glucohydrolase; AltName: Full=Glucan 1,4-alpha-glucosidase; Flags: Precursor | 23.45356466 | 236.7496676 | 3.340373521 | | 1.42671E-34 |
| Bcin04g03820 | - | 1.016205297 | 9.95154403 | 3.294771932 | | 0.027816495 |
| Bcin07g02950 | - | 1.000937204 | 9.704143677 | 3.265613389 | | 0.035742913 |
| Bcin11g01320 | - | 2.995927961 | 28.61199184 | 3.244213258 | | 0.000291074 |
| Bcin04g03800 | - | 1.329993838 | 12.22149065 | 3.187105 | | 0.020789995 |
| Bcin13g04470 | Q59VM4.1 RecName: Full=Transcriptional regulator RPN4 | 71.97570332 | 642.7616074 | 3.15825112 | | 5.23577E-80 |
| Bcin13g04780 | - | 32.37383277 | 283.896929 | 3.130685933 | | 3.70167E-38 |
| Bcin11g05360 | Q7Z9M7.3 RecName: Full=Endoglucanase-7; AltName: Full=Cellulase-61B; Short=Cel61B; AltName: Full=Endo-1,4-beta-glucanase VII; Short=EGVII; AltName: Full=Endoglucanase VII; AltName: Full=Endoglucanase-61B; Flags: Precursor | 8.918284824 | 77.18223459 | 3.122681595 | | 1.90064E-08 |
| Bcin07g05710 | Q54DY9.1 RecName: Full=Probable mitochondrial chaperone BCS1-B; AltName: Full=BCS1-like protein 2 | 1.33687749 | 11.56304227 | 3.104364239 | | 0.027481264 |
| novel.998 | - | 4.711108188 | 40.18568917 | 3.089580852 | | 2.41887E-06 |
| Bcin03g09120 | - | 20.16776306 | 166.1473304 | 3.037671141 | | 1.54694E-21 |
| Bcin14g04470 | Q9P768.1 RecName: Full=Uncharacterized amino-acid permease P7G5.06 | 30.08676496 | 245.207516 | 3.026306154 | | 2.64023E-32 |
| Bcin02g07440 | Q7Z9I0.2 RecName: Full=Uncharacterized MFS-type transporter SPBC409.08 | 279.1312485 | 2252.238175 | 3.011219994 | | 1.0117E-278 |
| Bcin10g04970 | - | 237.6605518 | 1910.682884 | 3.007267274 | | 5.1066E-248 |
| Bcin04g06540 | - | 9.069287084 | 68.16970459 | 2.905908524 | | 3.87506E-09 |
| Bcin15g04640 | - | 2.000153495 | 14.95074945 | 2.891335924 | | 0.021837182 |
| novel.12 | - | 1.998432582 | 14.94978776 | 2.890612569 | | 0.01309203 |
| Bcin05g01510 | - | 17.5804539 | 123.1271224 | 2.808098605 | | 5.77908E-17 |
| Bcin09g05240 | Q92253.2 RecName: Full=Probable glucose transporter rco-3 | 56.76850067 | 388.9083644 | 2.780171291 | | 6.79287E-40 |
| Bcin03g07090 | - | 1.725285581 | 11.25619121 | 2.721995643 | | 0.040343327 |
| Bcin09g05940 | W7MLD3.1 RecName: Full=Efflux pump FUS6; AltName: Full=Fusarin biosynthesis protein 6 | 14.42840318 | 95.01899183 | 2.714069017 | | 1.63346E-10 |
| Bcin16g02770 | A1DA48.1 RecName: Full=Neutral protease 2 homolog NFIA_031120; AltName: Full=Deuterolysin NFIA_031120; Flags: Precursor | 1842.13601 | 12055.45688 | 2.710509315 | | 0 |
| Bcin03g01210 | - | 3.085815604 | 19.71971935 | 2.685754951 | | 0.006711049 |
| Bcin09g02710 | Q9M8Z7.1 RecName: Full=Sterol 3-beta-glucosyltransferase UGT80A2; AltName: Full=UDP-glucose:sterol glucosyltransferase 80A2 | 2.351361873 | 14.8826684 | 2.656839821 | | 0.014661284 |
| Bcin12g06750 | P46030.1 RecName: Full=Peptide transporter PTR2 | 56.05301923 | 352.7068596 | 2.653150452 | | 7.07295E-41 |
| Bcin05g01970 | - | 21.26525144 | 132.4152428 | 2.638366208 | | 1.04373E-14 |
| novel.358 | - | 2.339535607 | 14.54737008 | 2.629173873 | | 0.038262342 |
| Bcin02g00440 | - | 476.2862463 | 2914.514332 | 2.612495743 | | 6.8662E-271 |
| Bcin10g05950 | Q9P413.1 RecName: Full=pH-response transcription factor pacC/RIM101 | 740.1663466 | 4435.252688 | 2.582729636 | | 0 |
| Bcin08g00330 | - | 3.659203966 | 21.63305755 | 2.554131186 | | 0.012185578 |
| Bcin10g05670 | - | 33.23859555 | 190.5769032 | 2.522179921 | | 5.63777E-21 |
| Bcin01g06040 | P53390.1 RecName: Full=Ammonium transporter MEP3 | 157.9571614 | 875.4574259 | 2.470807265 | | 8.40376E-86 |
| Bcin10g04810 | Q2YIJ8.1 RecName: Full=Glucose/galactose transporter >P0C105.1 RecName: Full=Glucose/galactose transporter | 28.7925123 | 159.1590371 | 2.461668065 | | 3.42007E-12 |
| Bcin14g01410 | Q9URZ3.1 RecName: Full=Probable proline-specific permease put4 | 47.76010808 | 261.8540837 | 2.456286512 | | 2.57325E-25 |
| Bcin04g03500 | - | 6.419404763 | 35.22578324 | 2.455594527 | | 8.64419E-05 |
| Bcin14g03680 | Q84P25.2 RecName: Full=4-coumarate--CoA ligase-like 2 | 29.73683725 | 162.3606876 | 2.448329521 | | 1.673E-11 |
| Bcin05g06250 | - | 63.62083714 | 344.222846 | 2.438976376 | | 1.16562E-23 |
| Bcin01g02940 | - | 732.389369 | 3967.960276 | 2.437814374 | | 8.9466E-270 |
| Bcin15g04510 | - | 10.45519055 | 56.50140234 | 2.433678938 | | 5.14869E-06 |
| Bcin06g05070 | - | 3.058721244 | 16.43418442 | 2.430246988 | | 0.012223198 |
| Bcin04g00160 | - | 129.854746 | 700.1854918 | 2.428492457 | | 2.10931E-46 |
| Bcin12g00760 | O94300.1 RecName: Full=Putative xanthine/uracil permease C887.17 | 113.6267111 | 596.7027388 | 2.394712133 | | 1.74421E-50 |
| Bcin09g03090 | Q3UMZ3.1 RecName: Full=Phospholipid phosphatase 5; AltName: Full=Phosphatidic acid phosphatase type 2 domain-containing protein 1B | 425.6846232 | 2235.250432 | 2.39366107 | | 9.4019E-188 |
| novel.525 | - | 3.057000331 | 16.04773142 | 2.392779111 | | 0.029267492 |
| Bcin11g03050 | Q12691.1 RecName: Full=Sodium transport ATPase 5 | 33.07236745 | 168.9364755 | 2.351621789 | | 3.10059E-17 |
| Bcin12g00640 | - | 2555.345991 | 13024.51964 | 2.349273417 | | 0 |
| Bcin01g03760 | - | 11.55117814 | 58.17723075 | 2.33581029 | | 1.36314E-06 |
| Bcin05g04840 | O94469.1 RecName: Full=Probable urea active transporter 1 | 25.71181719 | 128.461196 | 2.320997762 | | 2.75224E-12 |
| Bcin13g03260 | Q01389.1 RecName: Full=Serine/threonine-protein kinase BCK1/SLK1/SSP31 | 58.87411452 | 290.1097001 | 2.298389781 | | 4.71153E-24 |
| Bcin03g05230 | P40474.1 RecName: Full=Quinidine resistance protein 2 | 53.46076746 | 261.5045111 | 2.291023195 | | 6.82409E-26 |
| novel.927 | - | 7.501625045 | 35.96658696 | 2.266169417 | | 0.000801658 |
| Bcin05g07090 | Q29496.1 RecName: Full=Cytochrome P450 3A24; AltName: Full=CYPIIIA24 | 7.410016489 | 35.54883197 | 2.260227682 | | 0.002533385 |
| Bcin11g02370 | - | 5.465992749 | 26.01504293 | 2.256109188 | | 0.005009623 |
| Bcin01g03320 | - | 5.05887474 | 23.97310994 | 2.242229877 | | 0.004049565 |
| Bcin13g05270 | Q8LNW4.1 RecName: Full=Flotillin-like protein 2; AltName: Full=Nodulin-like protein 2 | 71.87376929 | 335.4979417 | 2.221495528 | | 1.19825E-29 |
| Bcin03g00760 | - | 160.3311575 | 744.1258282 | 2.213614976 | | 5.10523E-68 |
| Bcin13g05050 | - | 7.118379693 | 32.67391489 | 2.200051834 | | 0.000725239 |
| Bcin11g05020 | - | 17.3275176 | 79.37580413 | 2.199181611 | | 7.92497E-08 |
| novel.155 | - | 10.18204355 | 46.06581399 | 2.180852061 | | 7.90329E-05 |
| Bcin01g03240 | - | 47.4738119 | 211.8211846 | 2.158798878 | | 0.002084342 |
| novel.1025 | - | 5.026617641 | 22.22129884 | 2.139171746 | | 0.009091343 |
| Bcin03g06460 | P38256.1 RecName: Full=Uncharacterized protein YBR096W | 7.091285333 | 31.02832545 | 2.128576794 | | 0.001246976 |
| Bcin07g00210 | - | 13.20700691 | 55.58818348 | 2.074513143 | | 1.11786E-05 |
| Bcin08g01130 | - | 4.811101184 | 20.06268632 | 2.0695028 | | 0.023075012 |
| Bcin02g07640 | Q96UM2.1 RecName: Full=Laccase-3; AltName: Full=Benzenediol:oxygen oxidoreductase 3; AltName: Full=Diphenol oxidase 3; AltName: Full=Urishiol oxidase 3 | 12.16864987 | 49.57126371 | 2.027009018 | | 0.000113277 |
| Bcin05g07510 | O74529.1 RecName: Full=Uncharacterized methyltransferase C70.08c | 76.36781799 | 309.707693 | 2.02191196 | | 1.49161E-21 |
| Bcin05g03560 | - | 75.89300632 | 306.4806416 | 2.01203083 | | 6.41659E-24 |
| Bcin07g00250 | - | 26.42932416 | 106.5364275 | 2.006756248 | | 3.59279E-06 |
| Bcin08g06490 | - | 15.16651887 | 60.6935147 | 1.998843131 | | 1.07155E-05 |
| Bcin10g04570 | - | 5.430293825 | 21.5501707 | 1.992041734 | | 0.015753813 |
| Bcin05g04580 | P55306.1 RecName: Full=Catalase | 6.097011657 | 24.11525032 | 1.985981896 | | 0.011165798 |
| Bcin14g01400 | Q6ZFZ4.1 RecName: Full=Calpain-type cysteine protease ADL1; AltName: Full=Phytocalpain ADL1; AltName: Full=Protein ADAXIALIZED LEAF1; AltName: Full=Protein DEFECTIVE KERNEL 1; Short=OsDEK1; AltName: Full=Protein SHOOTLESS 3; Flags: Precursor | 6.339622473 | 25.22235582 | 1.985445459 | | 0.022971626 |
| Bcin07g01270 | P36842.2 RecName: Full=Nitrate reductase [NADPH]; Short=NR | 25.34317955 | 100.0521926 | 1.981018864 | | 8.64736E-08 |
| Bcin08g02540 | Q1DKE7.1 RecName: Full=DNA ligase 4; AltName: Full=DNA ligase IV; AltName: Full=Polydeoxyribonucleotide synthase [ATP] 4 | 5.116505284 | 20.04595714 | 1.975187249 | | 0.027415715 |
| Bcin15g02820 | Q9TLY2.1 RecName: Full=Protein CfxQ homolog | 18.69493128 | 72.74461492 | 1.962204092 | | 1.35383E-05 |
| Bcin07g04770 | - | 78.31338476 | 303.0169875 | 1.951235009 | | 1.82894E-21 |
| Bcin09g03120 | - | 7.164183971 | 27.57465779 | 1.948632485 | | 0.012052273 |
| Bcin16g04050 | O93934.1 RecName: Full=NADP-specific glutamate dehydrogenase; Short=NADP-GDH; AltName: Full=NADP-dependent glutamate dehydrogenase | 786.7184255 | 3010.224233 | 1.935800615 | | 6.8596E-207 |
| Bcin07g02850 | Q09686.1 RecName: Full=Putative glutamine amidotransferase-like protein C13C5.04 | 165.5562603 | 630.9916341 | 1.929704564 | | 2.74355E-47 |
| Bcin03g07360 | Q8NJK6.1 RecName: Full=Probable pectin lyase F; Short=PLF; Flags: Precursor >A2R6A1.1 RecName: Full=Probable pectin lyase F; Short=PLF; Flags: Precursor | 306.3212381 | 1164.120273 | 1.925717547 | | 1.29153E-75 |
| Bcin05g01530 | - | 60.8131534 | 228.0457749 | 1.906503846 | | 1.66236E-17 |
| Bcin06g06720 | - | 174.2641914 | 652.3130982 | 1.904943862 | | 9.62123E-50 |
| Bcin08g05090 | - | 62.33006855 | 233.3203978 | 1.902306056 | | 1.321E-16 |
| Bcin12g00650 | - | 35.26928523 | 131.2374587 | 1.897248423 | | 5.49983E-09 |
| Bcin06g05580 | - | 217.5247302 | 808.6220462 | 1.893089392 | | 3.22611E-50 |
| Bcin07g06730 | - | 6.421125676 | 23.75555417 | 1.888654003 | | 0.012155192 |
| Bcin13g01790 | - | 116.8499818 | 429.7352716 | 1.880051523 | | 4.89869E-25 |
| Bcin09g06670 | - | 116.2792371 | 425.7526161 | 1.87278105 | | 7.75451E-27 |
| Bcin09g02070 | P36091.1 RecName: Full=Mannan endo-1,6-alpha-mannosidase DCW1; AltName: Full=Defective cell wall 1; AltName: Full=Endo-alpha-1->6-D-mannanase DCW1; Flags: Precursor | 79.21150527 | 288.9240144 | 1.867317676 | | 9.63085E-19 |
| Bcin03g05820 | A2QV36.1 RecName: Full=Probable pectate lyase A; Flags: Precursor | 649.5987488 | 2359.465391 | 1.860419897 | | 1.6542E-127 |
| Bcin14g03430 | Q5BA61.1 RecName: Full=Pectin lyase B; Short=PLB; Flags: Precursor | 224.0536663 | 809.7032607 | 1.85486455 | | 2.53834E-45 |
| Bcin02g02420 | P80235.2 RecName: Full=Putative mitochondrial carnitine O-acetyltransferase | 581.5303254 | 2103.3697 | 1.853919302 | | 1.5081E-101 |
| novel.782 | - | 8.041255521 | 29.14247281 | 1.851880217 | | 0.008114994 |
| Bcin02g04840 | Q7RVX9.2 RecName: Full=Repressible high-affinity phosphate permease | 28.03758549 | 100.3591395 | 1.838879386 | | 4.57912E-07 |
| Bcin02g05670 | - | 22.62233963 | 80.48363834 | 1.829284567 | | 4.53754E-06 |
| novel.481 | - | 230.8754666 | 816.6161386 | 1.823775305 | | 6.89176E-38 |
| Bcin06g05410 | - | 168.6405329 | 594.0898307 | 1.817701737 | | 1.80174E-35 |
| Bcin06g03440 | Q8NJ59.1 RecName: Full=Alternative oxidase, mitochondrial; Flags: Precursor | 224.5015526 | 786.6130949 | 1.808193703 | | 2.46685E-55 |
| Bcin16g04120 | O74023.1 RecName: Full=Methylated-DNA--protein-cysteine methyltransferase; AltName: Full=6-O-methylguanine-DNA methyltransferase; Short=MGMT; AltName: Full=O-6-methylguanine-DNA-alkyltransferase; AltName: Full=Pk-MGMT | 63.44234252 | 217.6871006 | 1.780400543 | | 5.64806E-14 |
| Bcin13g03720 | - | 13.08486217 | 44.96252491 | 1.776544405 | | 0.001792222 |
| Bcin02g00720 | - | 13.17969243 | 44.90224417 | 1.76965373 | | 0.002282967 |
| Bcin05g05830 | C5FHK0.1 RecName: Full=Tripeptidyl-peptidase SED1; AltName: Full=Sedolisin-A; Flags: Precursor | 263.1226544 | 895.0818832 | 1.766842387 | | 5.932E-57 |
| Bcin05g03570 | - | 209.5978552 | 713.2900576 | 1.765830082 | | 3.04411E-39 |
| Bcin04g02370 | - | 61.81731231 | 209.0591906 | 1.757320749 | | 3.45979E-14 |
| Bcin03g08650 | B8NM76.2 RecName: Full=ustiloxin B cluster transcription factor ustR; AltName: Full=Ustiloxin B biosynthesis protein R | 14.98626108 | 50.50151027 | 1.756663954 | | 0.003225258 |
| Bcin10g06050 | - | 13.48875848 | 45.26074563 | 1.745091771 | | 0.002530231 |
| Bcin04g06370 | P50276.1 RecName: Full=High-affinity methionine permease | 18.65256883 | 62.23331265 | 1.740650466 | | 5.5765E-05 |
| Bcin10g03770 | - | 337.3704731 | 1126.504335 | 1.740183159 | | 1.84812E-45 |
| Bcin07g04370 | B8NM69.1 RecName: Full=Peptidase S41 family protein ustP; AltName: Full=Ustiloxin B biosynthesis protein P | 593.0320062 | 1967.681167 | 1.730733082 | | 2.9318E-123 |
| Bcin06g00120 | Q9FG72.1 RecName: Full=Oligopeptide transporter 1; Short=AtOPT1 | 36.58573189 | 121.1141682 | 1.728253551 | | 1.42489E-08 |
| Bcin15g01890 | - | 12.81321595 | 42.32265727 | 1.722403964 | | 0.00274626 |
| Bcin14g03350 | - | 8.429663612 | 27.85412463 | 1.722345481 | | 0.015673615 |
| Bcin14g02610 | P38041.1 RecName: Full=Protein BOB1; AltName: Full=BEM1-binding protein; AltName: Full=Growth inhibitory protein 7 | 102.6518274 | 339.023937 | 1.721393262 | | 5.82566E-17 |
| Bcin09g06680 | P12394.1 RecName: Full=Steroid 17-alpha-hydroxylase/17,20 lyase; AltName: Full=17-alpha-hydroxyprogesterone aldolase; AltName: Full=CYPXVII; AltName: Full=Cytochrome P450 17A1; AltName: Full=Cytochrome P450-C17; Short=Cytochrome P450c17 | 76.65477454 | 252.063105 | 1.716897918 | | 3.40348E-17 |
| Bcin09g05450 | - | 1070.323995 | 3507.584024 | 1.712257511 | | 5.3354E-170 |
| Bcin14g05350 | Q9USG8.1 RecName: Full=Meiotically up-regulated gene 190 protein | 120.808629 | 395.7103026 | 1.710722557 | | 6.39658E-25 |
| Bcin08g01160 | - | 98.77172408 | 319.0135042 | 1.690222413 | | 2.31105E-19 |
| novel.613 | - | 111.549419 | 359.1379151 | 1.687061145 | | 2.03928E-23 |
| Bcin12g00910 | - | 22.57675548 | 72.58269344 | 1.683278893 | | 4.20953E-05 |
| Bcin12g03980 | - | 35.54583182 | 113.5873551 | 1.679273727 | | 9.08263E-05 |
| Bcin04g06590 | - | 21.93514872 | 69.70840373 | 1.67254626 | | 0.002374576 |
| Bcin01g10420 | - | 48.94496502 | 155.1277832 | 1.663297857 | | 1.3228E-09 |
| Bcin07g00230 | Q01262.1 RecName: Full=Hydantoin utilization protein A; AltName: Full=ORF2 [Pseudomonas sp. NS671] | 49.22579384 | 155.0435044 | 1.657234033 | | 1.77552E-08 |
| Bcin09g04510 | Q4PRC2.1 RecName: Full=Glutamate decarboxylase 2; AltName: Full=65 kDa glutamic acid decarboxylase; Short=GAD-65; AltName: Full=Glutamate decarboxylase 65 kDa isoform | 8.40923278 | 26.53317845 | 1.65493343 | | 0.032523593 |
| Bcin12g05550 | P00504.3 RecName: Full=Aspartate aminotransferase, cytoplasmic; Short=cAspAT; AltName: Full=Cysteine aminotransferase, cytoplasmic; AltName: Full=Cysteine transaminase, cytoplasmic; Short=cCAT; AltName: Full=Glutamate oxaloacetate transaminase 1; Alt | 1542.195754 | 4850.643986 | 1.652770137 | | 4.7581E-194 |
| Bcin01g11010 | P13584.2 RecName: Full=Cytochrome P450 4B1; AltName: Full=CYPIVB1; AltName: Full=Cytochrome P450-HP | 36.16162488 | 113.0668005 | 1.644457558 | | 9.5501E-08 |
| Bcin03g08830 | Q8T9S7.1 RecName: Full=Phosphatidylinositol 3,4,5-trisphosphate 3-phosphatase and dual-specificity protein phosphatase PTEN; AltName: Full=Pten 3-phosphoinositide phosphatase alpha | 319.021712 | 992.4301407 | 1.637330263 | | 8.53281E-61 |
| Bcin15g02260 | - | 64.64056876 | 200.4805775 | 1.630627286 | | 2.45945E-11 |
| Bcin11g01080 | - | 22.14206061 | 67.92548783 | 1.620054622 | | 0.001444284 |
| Bcin07g02790 | A0A0D2YG01.1 RecName: Full=Non-canonical non-ribosomal peptide synthetase FUB8; AltName: Full=Fusaric acid biosynthesis protein 8 | 592.8419899 | 1820.735051 | 1.618547596 | | 3.41398E-79 |
| Bcin02g01010 | - | 80.3034329 | 246.1252199 | 1.61491269 | | 1.98537E-13 |
| Bcin09g05440 | - | 10.87757666 | 33.23317742 | 1.614527515 | | 0.010909212 |
| Bcin05g06980 | - | 17.56346489 | 53.46144025 | 1.606149167 | | 0.000600675 |
| Bcin16g03370 | O74869.3 RecName: Full=High-affinity nickel transport protein nic1 | 59.06739474 | 179.0134666 | 1.602264034 | | 2.33106E-08 |
| Bcin15g01960 | - | 26.55641151 | 80.11467901 | 1.589616256 | | 0.000177038 |
| Bcin06g04070 | Q94CA0.1 RecName: Full=Protein LAZ1 homolog 1; AltName: Full=Lazarus1 homolog 1; Flags: Precursor | 24.1607039 | 72.45940828 | 1.587328605 | | 0.000379126 |
| Bcin13g00340 | - | 11.86840851 | 35.45230367 | 1.581137648 | | 0.00599699 |
| Bcin02g02050 | - | 28.75182851 | 85.31411945 | 1.57007398 | | 6.85522E-05 |
| Bcin15g00630 | - | 21.03768859 | 62.33292869 | 1.569023871 | | 0.000421018 |
| Bcin10g02710 | - | 76.50553544 | 227.1289423 | 1.568203305 | | 7.35485E-13 |
| Bcin11g06200 | - | 60.19073906 | 178.0286433 | 1.56495574 | | 1.07001E-09 |
| Bcin09g00370 | F4HX15.1 RecName: Full=Phospholipase A I; Short=AtPLA1 | 564.7253759 | 1667.965285 | 1.5629896 | | 2.07984E-86 |
| Bcin04g04350 | P53388.1 RecName: Full=Dicarboxylic amino acid permease | 300.602269 | 887.6401431 | 1.561615925 | | 1.69545E-43 |
| Bcin10g01550 | P46030.1 RecName: Full=Peptide transporter PTR2 | 1642.026775 | 4838.377675 | 1.55905459 | | 1.4869E-164 |
| Bcin10g03910 | P31540.2 RecName: Full=Heat shock protein hsp98; AltName: Full=Protein aggregation-remodeling factor hsp98 | 1815.122465 | 5347.507283 | 1.558743322 | | 9.0275E-217 |
| Bcin02g07090 | P32382.1 RecName: Full=NADH oxidase | 160.1365121 | 471.7114082 | 1.558363506 | | 1.88849E-25 |
| Bcin15g01370 | - | 29.41682543 | 86.60585388 | 1.557827122 | | 1.15079E-05 |
| Bcin05g01640 | Q9XIG1.1 RecName: Full=Sterol 3-beta-glucosyltransferase UGT80B1; AltName: Full=Protein TRANSPARENT TESTA 15; AltName: Full=UDP-glucose:sterol glucosyltransferase 80B1 | 60.44945838 | 177.914287 | 1.556727617 | | 7.43341E-08 |
| Bcin06g02240 | - | 1701.197069 | 4958.750516 | 1.543272441 | | 7.2914E-199 |
| Bcin06g01170 | - | 150.0562247 | 437.2295522 | 1.542058238 | | 3.49855E-20 |
| Bcin04g03550 | - | 89.26275727 | 259.2581978 | 1.539982121 | | 1.49315E-12 |
| Bcin03g00540 | P19571.1 RecName: Full=Glucan 1,4-alpha-maltohexaosidase; AltName: Full=Exo-maltohexaohydrolase; AltName: Full=G6-amylase; AltName: Full=Maltohexaose-producing amylase; Flags: Precursor | 195.5410912 | 568.4685642 | 1.539040694 | | 1.35375E-26 |
| Bcin14g03670 | Q08645.1 RecName: Full=Folylpolyglutamate synthase; AltName: Full=Folylpoly-gamma-glutamate synthetase; Short=FPGS; AltName: Full=Tetrahydrofolylpolyglutamate synthase; Short=Tetrahydrofolate synthase >B3LJR0.1 RecName: Full=Folylpolyglutamate syntha | 10.41132731 | 30.28954644 | 1.53771562 | | 0.021019452 |
| Bcin13g04610 | Q9M8Z7.1 RecName: Full=Sterol 3-beta-glucosyltransferase UGT80A2; AltName: Full=UDP-glucose:sterol glucosyltransferase 80A2 | 110.6068149 | 321.0788679 | 1.536389862 | | 3.89984E-14 |
| Bcin03g06610 | - | 110.8622703 | 320.3060031 | 1.530923778 | | 3.15648E-18 |
| Bcin05g01520 | Q8N0N3.1 RecName: Full=Beta-1,3-glucan-binding protein; Short=GBP; Flags: Precursor | 98.70222502 | 285.1696711 | 1.530169984 | | 1.81288E-14 |
| Bcin12g00380 | D4AK18.2 RecName: Full=Uncharacterized secreted protein ARB_06907; Flags: Precursor | 399.3758918 | 1152.935423 | 1.529572538 | | 1.27853E-55 |
| Bcin14g05530 | Q00746.1 RecName: Full=Antigen 1; AltName: Full=ASPND1; Flags: Precursor | 14.17740793 | 40.89037699 | 1.528139188 | | 0.004087656 |
| Bcin16g03040 | - | 135.6714646 | 388.3560363 | 1.516487718 | | 2.72931E-21 |
| novel.968 | - | 38.32650569 | 109.4326547 | 1.515458672 | | 3.45379E-06 |
| Bcin01g05790 | P22944.2 RecName: Full=Nitrite reductase [NAD(P)H] | 73.11405336 | 208.5148352 | 1.512422838 | | 3.79258E-11 |
| Bcin09g07120 | - | 15.59901032 | 44.408805 | 1.510220927 | | 0.005258726 |
| Bcin10g01980 | O00093.2 RecName: Full=3-phytase B; AltName: Full=3 phytase B; AltName: Full=Myo-inositol hexakisphosphate phosphohydrolase B; AltName: Full=Myo-inositol-hexaphosphate 3-phosphohydrolase B; Flags: Precursor | 160.418884 | 452.8514298 | 1.497017523 | | 6.59695E-22 |
| Bcin16g03420 | P33303.2 RecName: Full=Succinate/fumarate mitochondrial transporter; AltName: Full=Regulator of acetyl-CoA synthase activity | 2504.475334 | 7063.156228 | 1.495979421 | | 5.6656E-251 |
| novel.482 | - | 14.87960456 | 41.84140215 | 1.492665384 | | 0.00512085 |
| Bcin13g00250 | Q75A82.1 RecName: Full=Peroxisomal adenine nucleotide transporter 1 | 67.80224233 | 190.5333159 | 1.492146072 | | 4.77759E-10 |
| Bcin12g02780 | - | 214.3951891 | 602.9397761 | 1.491102527 | | 1.00288E-26 |
| Bcin01g11030 | N4WEA4.1 RecName: Full=3-hydroxyacyl-CoA dehydrogenase-like protein LAM1; AltName: Full=T-toxin biosynthesis protein LAM1 | 57.16753886 | 159.7539495 | 1.47993304 | | 1.24511E-06 |
| Bcin04g02380 | Q67JH7.1 RecName: Full=Imidazolonepropionase; AltName: Full=Imidazolone-5-propionate hydrolase | 17.49034613 | 48.85409332 | 1.479212572 | | 0.005998193 |
| Bcin09g03260 | - | 100.5228234 | 277.8399453 | 1.466149898 | | 1.33643E-12 |
| Bcin12g02300 | Q92253.2 RecName: Full=Probable glucose transporter rco-3 | 1448.815279 | 4002.490614 | 1.465742425 | | 1.8566E-172 |
| Bcin01g02950 | G3XMC0.1 RecName: Full=Acyl-CoA ligase azaF; AltName: Full=Azaphilone biosynthesis cluster protein azaF | 52.72049061 | 145.4333328 | 1.463685322 | | 7.65373E-07 |
| novel.842 | - | 27.0093338 | 74.14944544 | 1.456741306 | | 0.000199682 |
| Bcin10g00520 | - | 931.472233 | 2550.581485 | 1.452523118 | | 1.14307E-77 |
| Bcin07g00140 | - | 11.17459632 | 30.45223242 | 1.44650437 | | 0.01994634 |
| Bcin05g05240 | F4I111.1 RecName: Full=Calcium uniporter protein 6, mitochondrial; Flags: Precursor | 169.7139707 | 459.5318746 | 1.436975925 | | 3.52056E-24 |
| Bcin04g00870 | - | 25.83572509 | 70.0852647 | 1.436502987 | | 0.001647157 |
| Bcin09g02820 | P53806.2 RecName: Full=Calcipressin-like protein; AltName: Full=Down syndrome candidate region 1-like protein | 1999.497345 | 5400.542239 | 1.43330849 | | 4.142E-196 |
| Bcin10g05190 | - | 16.17584051 | 43.26856999 | 1.417884065 | | 0.007151286 |
| novel.908 | - | 27.76766019 | 74.10576221 | 1.417758429 | | 0.000177905 |
| Bcin05g06000 | - | 222.9626213 | 592.715801 | 1.411508497 | | 1.21364E-24 |
| Bcin03g00460 | Q87GU5.1 RecName: Full=Autoinducer 2 sensor kinase/phosphatase LuxQ | 308.4645874 | 820.7682471 | 1.411324871 | | 2.24091E-36 |
| Bcin10g05610 | - | 565.1329785 | 1503.180279 | 1.41114793 | | 2.49354E-54 |
| Bcin04g05700 | P0CH36.1 RecName: Full=NADP-dependent alcohol dehydrogenase C 1; Short=Ms-ADHC 1 >P0CH37.1 RecName: Full=NADP-dependent alcohol dehydrogenase C 2; Short=Ms-ADHC 2 | 820.9450203 | 2173.421208 | 1.404010266 | | 1.4179E-70 |
| Bcin13g04620 | - | 40.98649336 | 108.0955108 | 1.400461418 | | 4.17882E-06 |
| Bcin08g06370 | - | 20.23033622 | 53.38462752 | 1.399594982 | | 0.002727036 |
| Bcin04g00640 | A0A0R8YWJ7.2 RecName: Full=Methylphloroacetophenone synthase; Short=MPAS; AltName: Full=Non-reducing polyketide synthase MPAS; AltName: Full=Usnic acid biosynthesis protein MPAS | 23.39725702 | 61.87125748 | 1.399531578 | | 0.0315017 |
| Bcin06g07160 | A0MTQ2.1 RecName: Full=Beta-peptidyl aminopeptidase BapA; Contains: RecName: Full=Beta-peptidyl aminopeptidase BapA alpha subunit; Contains: RecName: Full=Beta-peptidyl aminopeptidase BapA beta subunit; Flags: Precursor | 16.2063767 | 42.59445545 | 1.393706002 | | 0.00888131 |
| Bcin11g01680 | - | 299.7163705 | 785.7002704 | 1.390479597 | | 4.81831E-32 |
| Bcin06g03910 | P32793.2 RecName: Full=Protein YSC84; AltName: Full=LAS seventeen-binding protein 4; Short=LAS17-binding protein 4 | 209.0382342 | 545.4355968 | 1.382997857 | | 3.812E-26 |
| Bcin01g08230 | Q4WJ81.1 RecName: Full=C2H2 finger domain transcription factor crzA | 504.2696964 | 1314.496108 | 1.382434763 | | 1.53142E-56 |
| Bcin01g06570 | P21657.3 RecName: Full=Transcriptional activator protein DAL81; AltName: Full=Regulatory protein UGA35 | 287.7232581 | 749.0317903 | 1.379840464 | | 6.85957E-33 |
| Bcin11g01950 | P53326.1 RecName: Full=Uncharacterized protein YGR266W | 152.5526812 | 396.8537157 | 1.379114288 | | 5.15424E-17 |
| Bcin06g01880 | - | 11.82110344 | 30.77027005 | 1.379035785 | | 0.033683008 |
| Bcin03g06400 | - | 13.87522553 | 35.85472135 | 1.37022222 | | 0.018471184 |
| Bcin10g06070 | Q9URW6.1 RecName: Full=SH3 domain-containing protein PJ696.02 | 733.8900954 | 1891.483917 | 1.36587593 | | 4.92295E-73 |
| Bcin14g04610 | A0QZE3.1 RecName: Full=Putative hydrolase MSMEG_3995/MSMEI_3903 | 48.58815365 | 125.2506821 | 1.365338289 | | 3.41415E-06 |
| Bcin01g10380 | Q2UNA2.1 RecName: Full=Bifunctional cytochrome P450/NADPH--P450 reductase; AltName: Full=AoCYP505A3; AltName: Full=Cytochrome P450 monooxygenase; AltName: Full=Fatty acid monooxygenase; AltName: Full=Flavocytochrome P450; Includes: RecName: Full=Cyto | 34.17007595 | 87.96059272 | 1.365151453 | | 9.05272E-05 |
| Bcin08g06460 | - | 1030.94313 | 2645.401877 | 1.359998983 | | 1.41286E-92 |
| Bcin13g02860 | Q9C291.3 RecName: Full=Double-strand break repair protein mus-23; AltName: Full=Recombinational repair protein mus-23 | 11.82088332 | 30.30328922 | 1.358148493 | | 0.029329669 |
| Bcin04g02890 | - | 428.0868587 | 1096.90418 | 1.357343868 | | 1.79735E-43 |
| Bcin16g02010 | Q4I624.1 RecName: Full=Hsp70 nucleotide exchange factor FES1 | 646.7825116 | 1646.287916 | 1.348038097 | | 9.90529E-72 |
| Bcin09g06910 | O14197.1 RecName: Full=Uncharacterized transporter C5D6.04 | 644.248593 | 1639.659845 | 1.347723711 | | 6.35434E-72 |
| Bcin13g01150 | - | 144.9636454 | 368.4555045 | 1.347506501 | | 1.42908E-12 |
| Bcin05g06500 | Q9ZVF6.2 RecName: Full=Phytanoyl-CoA dioxygenase; AltName: Full=Phytanoyl-CoA 2-hydroxylase | 79.5945305 | 202.2851378 | 1.346491101 | | 5.43977E-09 |
| Bcin14g01520 | D4B1N9.1 RecName: Full=Probable secreted lipase ARB_02369; Flags: Precursor | 32.63643416 | 83.09060946 | 1.346127416 | | 0.001547436 |
| novel.1099 | - | 13.59885683 | 34.36757129 | 1.341549655 | | 0.032798031 |
| Bcin04g03900 | - | 24.37471957 | 61.74063799 | 1.341423594 | | 0.001072151 |
| novel.103 | - | 16.49479179 | 41.71044834 | 1.33590664 | | 0.011710048 |
| Bcin08g03410 | - | 13.82103681 | 34.5759652 | 1.321285146 | | 0.023666523 |
| Bcin03g02760 | Q8NKB6.1 RecName: Full=Probable aspartic-type endopeptidase opsB; AltName: Full=Oryzapsin B; Flags: Precursor | 678.6981237 | 1694.547815 | 1.31965909 | | 6.99095E-50 |
| Bcin08g04970 | Q09887.1 RecName: Full=Uncharacterized amino-acid permease C584.13 | 14.68451893 | 36.51775066 | 1.318820014 | | 0.034524528 |
| Bcin03g08030 | - | 943.2361572 | 2352.018169 | 1.317894958 | | 2.57744E-88 |
| Bcin13g05470 | - | 240.563108 | 599.2476072 | 1.317414082 | | 4.87684E-26 |
| Bcin04g05600 | - | 319.8344473 | 796.6390271 | 1.316817261 | | 7.65421E-34 |
| Bcin04g04240 | O94611.2 RecName: Full=Meiotically up-regulated gene 65 protein | 51.86773197 | 128.6674639 | 1.311222834 | | 8.75575E-05 |
| Bcin02g03140 | - | 23.91879571 | 59.35072692 | 1.30986522 | | 0.00632159 |
| Bcin13g00810 | Q99042.1 RecName: Full=D-amino-acid oxidase; Short=DAAO; Short=DAMOX; Short=DAO | 18.6353597 | 46.08008827 | 1.307570676 | | 0.020798548 |
| Bcin07g02170 | Q12645.1 RecName: Full=Pisatin demethylase; AltName: Full=Cytochrome P450 57A1 | 57.87189665 | 143.3359985 | 1.306512984 | | 9.0013E-06 |
| Bcin04g00660 | Q00808.1 RecName: Full=Vegetative incompatibility protein HET-E-1 | 102.8991184 | 254.5920688 | 1.306174405 | | 1.95062E-11 |
| Bcin11g04070 | Q9HDW7.1 RecName: Full=Calcium-transporting ATPase 2 | 516.6889435 | 1277.552216 | 1.305996288 | | 3.93646E-48 |
| Bcin11g06210 | - | 20.22711452 | 50.05102563 | 1.305787103 | | 0.007314507 |
| Bcin08g01660 | - | 87.41214749 | 216.1777388 | 1.304973341 | | 1.01291E-06 |
| Bcin03g05480 | - | 925.224217 | 2286.690736 | 1.304776101 | | 4.10676E-60 |
| Bcin14g00870 | O59700.1 RecName: Full=Uncharacterized transporter C36.03c | 771.9554895 | 1902.690376 | 1.300841642 | | 1.96221E-62 |
| Bcin02g03510 | - | 1551.804967 | 3815.705882 | 1.298498565 | | 1.0116E-108 |
| Bcin01g04670 | - | 194.7523643 | 478.4474872 | 1.297413103 | | 2.63173E-18 |
| Bcin09g05130 | - | 149.7421315 | 364.2606029 | 1.283432961 | | 1.29507E-12 |
| Bcin03g05090 | Q99385.1 RecName: Full=Vacuolar calcium ion transporter; AltName: Full=High copy number undoes manganese protein 1; AltName: Full=Manganese resistance 1 protein; AltName: Full=Vacuolar Ca(2+)/H(+) exchanger | 1138.651809 | 2765.626501 | 1.280666951 | | 4.01845E-87 |
| Bcin09g06700 | - | 65.03193618 | 157.7514889 | 1.280096763 | | 1.21667E-06 |
| Bcin14g03150 | - | 33.92058148 | 82.2273864 | 1.279593028 | | 0.000429074 |
| Bcin15g05350 | - | 44.33711378 | 107.6586677 | 1.277103632 | | 0.000379538 |
| Bcin15g03340 | P40433.1 RecName: Full=6-phosphofructo-2-kinase 1; Short=6PF-2-K 1; AltName: Full=Phosphofructokinase 2 I | 343.3243734 | 829.872037 | 1.273022535 | | 2.9876E-31 |
| Bcin03g04090 | A6WXV2.1 RecName: Full=Glycerol kinase; AltName: Full=ATP:glycerol 3-phosphotransferase; AltName: Full=Glycerokinase; Short=GK | 3880.634653 | 9372.16201 | 1.272202141 | | 1.2831E-270 |
| Bcin05g01940 | - | 17.19182568 | 41.52293434 | 1.271691466 | | 0.030647867 |
| Bcin06g02480 | - | 93.67366634 | 225.4699444 | 1.267754277 | | 6.55927E-10 |
| Bcin08g06690 | - | 162.9299883 | 392.1673342 | 1.26769181 | | 5.39109E-13 |
| Bcin03g02140 | - | 18.94936836 | 45.45722429 | 1.263371556 | | 0.01085281 |
| Bcin07g00160 | Q873X9.1 RecName: Full=Endochitinase B1; AltName: Full=Chitinase B1; Flags: Precursor >E9QRF2.1 RecName: Full=Endochitinase B1; AltName: Full=Chitinase B1; Flags: Precursor | 1830.723014 | 4388.951904 | 1.26138514 | | 5.6338E-104 |
| Bcin14g03260 | - | 100.398035 | 240.5519462 | 1.260691 | | 1.32888E-09 |
| Bcin12g02330 | Q5EXK1.1 RecName: Full=3-hydroxybenzoate 6-hydroxylase | 112.4161651 | 269.5165929 | 1.260668227 | | 8.08649E-12 |
| novel.444 | - | 31.19802064 | 74.3782891 | 1.254670388 | | 0.001270357 |
| Bcin15g04180 | P36616.2 RecName: Full=Protein kinase dsk1; AltName: Full=Dis1-suppressing protein kinase | 18.922274 | 45.01387679 | 1.249497124 | | 0.019485493 |
| Bcin02g01440 | Q4WYA5.1 RecName: Full=Plasma membrane proteolipid 3 | 493.8779172 | 1173.352126 | 1.249153601 | | 5.37314E-38 |
| Bcin09g02230 | S0DPY2.1 RecName: Full=Efflux pump apf11; AltName: Full=Apicidin F synthesis protein 11 | 55.06218738 | 130.8295998 | 1.248142224 | | 4.1173E-06 |
| Bcin11g06180 | P53099.1 RecName: Full=Vitamin B6 transporter TPN1; AltName: Full=Transport of pyridoxine protein 1 | 1378.749524 | 3271.513213 | 1.246284168 | | 4.56635E-79 |
| Bcin07g01750 | Q9LQV2.1 RecName: Full=RNA-dependent RNA polymerase 1; Short=AtRDRP1; AltName: Full=RNA-directed RNA polymerase 1 | 25.94560331 | 61.51647658 | 1.244628419 | | 0.00499735 |
| Bcin12g00250 | - | 107.8698264 | 255.6259808 | 1.243554703 | | 4.08912E-10 |
| Bcin10g00420 | - | 51.12255476 | 120.9502875 | 1.240373478 | | 0.000555626 |
| Bcin02g07860 | Q10146.2 RecName: Full=Exosome complex exonuclease rrp6; AltName: Full=Ribosomal RNA-processing protein 6 | 19.21929366 | 45.26758426 | 1.236132893 | | 0.017144839 |
| Bcin09g06850 | Q9UV10.1 RecName: Full=Heterokaryon incompatibility protein 6, OR allele; Short=Het-6(OR) | 36.45198101 | 85.90372227 | 1.235947167 | | 0.000324758 |
| Bcin14g04310 | - | 21.01081435 | 49.45617871 | 1.235930909 | | 0.020388126 |
| Bcin04g05710 | Q09704.1 RecName: Full=Exosome complex component rrp4; AltName: Full=Ribosomal RNA-processing protein 4 | 130.1039359 | 306.1282798 | 1.233735169 | | 3.03965E-10 |
| Bcin16g04040 | O14088.1 RecName: Full=Uncharacterized oxidoreductase C2F3.05c | 890.1287171 | 2089.371368 | 1.230944535 | | 5.33129E-78 |
| Bcin09g01610 | P39932.2 RecName: Full=Sugar transporter STL1 | 406.9298068 | 954.7401899 | 1.230105264 | | 1.90621E-31 |
| Bcin03g04880 | - | 186.4883529 | 436.7227646 | 1.2271326 | | 8.98108E-17 |
| Bcin08g06880 | - | 48.75566242 | 113.6909605 | 1.22245814 | | 0.001468869 |
| Bcin08g00400 | - | 509.4857945 | 1186.777914 | 1.220419805 | | 5.98126E-35 |
| Bcin01g02920 | - | 397.3221276 | 925.2324668 | 1.219396569 | | 2.16857E-26 |
| Bcin02g07030 | - | 275.1807657 | 639.779584 | 1.21756581 | | 1.31033E-21 |
| Bcin14g00410 | P75791.1 RecName: Full=Uncharacterized protein YbiU | 18.77819764 | 43.71659974 | 1.216726442 | | 0.036279411 |
| Bcin13g05010 | - | 42.9627742 | 99.58063082 | 1.213731937 | | 0.000140425 |
| Bcin05g05710 | - | 26.79725917 | 61.83066198 | 1.207321634 | | 0.006037111 |
| Bcin15g02000 | - | 25.781274 | 59.42690681 | 1.206284522 | | 0.011063689 |
| Bcin01g09950 | O93918.1 RecName: Full=Pyruvate carboxylase; AltName: Full=Pyruvic carboxylase; Short=PCB | 11966.5001 | 27587.87887 | 1.204973571 | | 0 |
| Bcin04g00750 | P07038.1 RecName: Full=Plasma membrane ATPase; AltName: Full=Proton pump | 22.88021854 | 52.78818607 | 1.204779332 | | 0.015181746 |
| Bcin04g00880 | - | 88.83397001 | 204.8668095 | 1.204412886 | | 5.31788E-07 |
| Bcin16g03650 | O13817.2 RecName: Full=Protein transport protein sec73 | 121.5409215 | 279.9029912 | 1.203520051 | | 3.35574E-09 |
| Bcin08g04880 | D4GWH7.1 RecName: Full=Uncharacterized isochorismatase family protein HVO_2328 | 92.35007366 | 211.8450151 | 1.199386633 | | 3.02354E-07 |
| Bcin16g01050 | D4AUX6.1 RecName: Full=Uncharacterized secreted protein ARB_08043; Flags: Precursor | 448.4619739 | 1029.543462 | 1.19850299 | | 6.31409E-35 |
| Bcin12g05870 | Q1LZH1.1 RecName: Full=Mitochondrial amidoxime reducing component 2; Short=mARC2; AltName: Full=Molybdenum cofactor sulfurase C-terminal domain-containing protein 2; Short=MOSC domain-containing protein 2; Short=Moco sulfurase C-terminal domain-conta | 52.0152924 | 118.7995767 | 1.191229749 | | 3.26223E-05 |
| Bcin14g04530 | Q9P6N2.1 RecName: Full=Pdp3-interacting factor 1 | 1808.226361 | 4120.369936 | 1.188056188 | | 7.2329E-126 |
| Bcin05g04520 | - | 1171.245084 | 2666.916787 | 1.187162962 | | 1.16644E-60 |
| Bcin08g01350 | - | 863.3404605 | 1963.759653 | 1.185558579 | | 9.90527E-69 |
| Bcin08g00640 | - | 105.0534536 | 238.812415 | 1.184906297 | | 1.74286E-08 |
| Bcin06g05780 | - | 36.36359415 | 82.67913126 | 1.183136056 | | 0.001213065 |
| Bcin11g01450 | Q06153.1 RecName: Full=Cerato-ulmin; Short=CU; AltName: Full=Dutch elm disease toxin; Flags: Precursor | 24.89069751 | 56.56105025 | 1.181903431 | | 0.015657617 |
| Bcin14g02490 | P32867.2 RecName: Full=Protein SSO1 | 729.8750429 | 1654.884149 | 1.180730788 | | 1.16308E-56 |
| Bcin02g06300 | - | 26.34433688 | 59.55563872 | 1.178138823 | | 0.015748922 |
| Bcin06g02270 | - | 75.48412516 | 170.5611851 | 1.176688906 | | 4.21337E-07 |
| Bcin07g02430 | - | 273.6763371 | 617.7768324 | 1.174741762 | | 1.19228E-20 |
| novel.673 | - | 21.09381834 | 47.50778762 | 1.174078672 | | 0.024944808 |
| Bcin10g04180 | - | 581.624064 | 1306.053466 | 1.16748097 | | 2.52158E-42 |
| Bcin06g00330 | Q70J59.1 RecName: Full=Tripeptidyl-peptidase sed2; AltName: Full=Sedolisin-B; Flags: Precursor | 1054.404492 | 2366.665686 | 1.16611604 | | 2.13382E-74 |
| novel.223 | - | 16.85116291 | 37.79139437 | 1.163716169 | | 0.040070334 |
| Bcin01g00920 | - | 102.3528667 | 229.4992336 | 1.162917546 | | 5.15363E-07 |
| Bcin05g06270 | - | 166.5569352 | 372.6693929 | 1.162655229 | | 4.15541E-11 |
| Bcin10g01690 | - | 50.69002106 | 113.1383036 | 1.15865833 | | 0.00016986 |
| Bcin03g07060 | - | 67.96507086 | 151.2947038 | 1.154400403 | | 7.15856E-06 |
| Bcin16g00080 | A1CFM2.1 RecName: Full=FAD-linked oxidoreductase patO; AltName: Full=Patulin synthesis protein O; Flags: Precursor | 68.5270732 | 152.3646716 | 1.153160561 | | 0.000101392 |
| novel.1450 | PF11779:Small subunit of serine palmitoyltransferase-like | 17.51077696 | 38.9567917 | 1.152649137 | | 0.047585097 |
| Bcin01g08380 | - | 26.873862 | 59.84924272 | 1.152040609 | | 0.013218756 |
| Bcin05g00390 | W7MLD3.1 RecName: Full=Efflux pump FUS6; AltName: Full=Fusarin biosynthesis protein 6 | 91.02850651 | 201.623936 | 1.148055548 | | 3.23514E-07 |
| Bcin01g11060 | - | 129.4971898 | 286.6860384 | 1.146486103 | | 1.3487E-09 |
| Bcin08g03080 | Q9UUD1.1 RecName: Full=Sterol regulatory element-binding protein 1; Contains: RecName: Full=Processed sterol regulatory element-binding protein 1 | 36.23580418 | 79.83790552 | 1.140548117 | | 0.032385118 |
| Bcin14g04080 | P87000.2 RecName: Full=Transcriptional activator protein acu-15; AltName: Full=Acetate utilization protein 15 | 96.88338984 | 213.1603586 | 1.137695701 | | 8.7291E-07 |
| Bcin04g04190 | P14804.3 RecName: Full=Glucoamylase; AltName: Full=1,4-alpha-D-glucan glucohydrolase; AltName: Full=Glucan 1,4-alpha-glucosidase; Flags: Precursor | 3459.17274 | 7606.546682 | 1.136772296 | | 2.3743E-189 |
| Bcin15g01340 | Q9US37.1 RecName: Full=Uncharacterized transporter C1039.04 | 72.82612076 | 160.2546271 | 1.13672629 | | 4.47473E-05 |
| Bcin09g02790 | P16928.2 RecName: Full=Acetyl-coenzyme A synthetase; AltName: Full=Acetate--CoA ligase; AltName: Full=Acyl-activating enzyme | 7058.02872 | 15508.23823 | 1.135620223 | | 5.2546E-179 |
| Bcin16g01670 | - | 93.87073525 | 206.2671962 | 1.134386946 | | 5.9566E-06 |
| Bcin13g03340 | - | 35.02521588 | 76.92431631 | 1.134157509 | | 0.003437988 |
| Bcin02g01850 | Q9Y7N9.1 RecName: Full=PX domain-containing protein C1450.12 | 398.6588273 | 872.3933706 | 1.129813886 | | 9.00347E-22 |
| Bcin15g02200 | P36044.2 RecName: Full=Protein MNN4 | 475.6070418 | 1040.354818 | 1.128650472 | | 1.04489E-31 |
| Bcin02g00780 | Q28262.1 RecName: Full=Platelet-activating factor acetylhydrolase; Short=PAF acetylhydrolase; AltName: Full=1-alkyl-2-acetylglycerophosphocholine esterase; AltName: Full=2-acetyl-1-alkylglycerophosphocholine esterase; AltName: Full=LDL-associated pho | 96.58442915 | 210.0668172 | 1.121114538 | | 5.3703E-07 |
| Bcin15g03710 | - | 96.78877971 | 210.6617297 | 1.12089468 | | 6.71119E-07 |
| Bcin05g02400 | Q9P3B2.1 RecName: Full=Respiratory supercomplex factor 2 homolog C1565.01 | 358.41539 | 779.0528538 | 1.120194795 | | 2.48364E-25 |
| Bcin09g03330 | P87049.3 RecName: Full=G1/S-specific cyclin pas1 | 624.5620449 | 1353.599985 | 1.116050591 | | 1.20268E-28 |
| novel.107 | - | 478.7015976 | 1037.444938 | 1.115780319 | | 7.03673E-33 |
| Bcin13g04830 | A1CEK6.1 RecName: Full=Class E vacuolar protein-sorting machinery protein hse1 | 329.8075022 | 714.6011436 | 1.114745838 | | 2.54518E-21 |
| Bcin13g00050 | - | 1453.235064 | 3143.47169 | 1.113101242 | | 3.3512E-86 |
| Bcin08g05570 | O74541.4 RecName: Full=Uncharacterized transcriptional regulatory protein C777.02 | 238.2705196 | 515.1690509 | 1.112048341 | | 6.83283E-16 |
| Bcin09g07010 | D4B387.1 RecName: Full=Gamma-glutamyltransferase ARB_02921; AltName: Full=Gamma-glutamyltranspeptidase; Short=Gamma-GT; AltName: Full=Glutathione hydrolase; AltName: Full=Leukotriene-C4 hydrolase; Contains: RecName: Full=Gamma-glutamyltransferase hea | 100.6460286 | 217.5027702 | 1.110812248 | | 2.11004E-07 |
| Bcin14g03610 | - | 2328.002677 | 5024.176599 | 1.110028433 | | 5.85189E-65 |
| Bcin15g03000 | - | 54.56319845 | 117.6104447 | 1.109421952 | | 0.001080037 |
| Bcin01g09180 | P47032.1 RecName: Full=Protein PRY1; AltName: Full=Pathogenesis-related protein 1; Flags: Precursor | 633.32981 | 1365.991245 | 1.109218721 | | 1.24585E-29 |
| Bcin02g05740 | P32375.2 RecName: Full=Allantoinase | 491.2552159 | 1058.782408 | 1.107889699 | | 1.89276E-26 |
| Bcin01g09720 | - | 37.7243443 | 81.35360416 | 1.107072671 | | 0.002075486 |
| Bcin02g00870 | Q9VSL3.1 RecName: Full=Pyrimidodiazepine synthase; AltName: Full=Protein sepia | 22.36790255 | 48.1138211 | 1.106362602 | | 0.019485493 |
| Bcin09g06580 | - | 45.77200098 | 98.09005871 | 1.101270791 | | 0.000858568 |
| Bcin01g04480 | - | 300.1495245 | 643.2214924 | 1.099640613 | | 8.09834E-20 |
| Bcin08g01010 | - | 168.6487395 | 361.0529795 | 1.098575531 | | 5.81796E-12 |
| Bcin12g02310 | Q12553.2 RecName: Full=Xanthine dehydrogenase; AltName: Full=Purine hydroxylase I | 65.66489612 | 140.2630083 | 1.095413207 | | 2.38826E-05 |
| Bcin01g00430 | - | 143.735092 | 307.1341147 | 1.094605351 | | 7.78052E-10 |
| Bcin11g01940 | - | 40.74758675 | 87.01009908 | 1.093263517 | | 0.002202003 |
| Bcin01g08080 | - | 232.7462359 | 496.2357473 | 1.091703081 | | 8.56999E-16 |
| Bcin02g05300 | - | 334.3892774 | 711.8311934 | 1.09010764 | | 1.51832E-22 |
| Bcin13g01160 | - | 85.81695084 | 182.4782795 | 1.088148408 | | 3.75054E-06 |
| Bcin06g03090 | - | 30.20030514 | 63.95797252 | 1.085319836 | | 0.013882 |
| Bcin09g03900 | Q09897.1 RecName: Full=Chitin synthase regulatory factor 3; AltName: Full=Chs four homolog 1 | 172.1105188 | 364.9170266 | 1.083598577 | | 8.5346E-10 |
| Bcin01g02890 | - | 534.2056814 | 1132.057068 | 1.083595677 | | 7.13055E-26 |
| Bcin04g05720 | - | 1065.099605 | 2254.582515 | 1.082118744 | | 2.36514E-61 |
| Bcin14g01430 | - | 18.30308136 | 38.68041259 | 1.081422865 | | 0.042785248 |
| Bcin11g04950 | - | 20.27269867 | 42.8626344 | 1.080551369 | | 0.03079297 |
| Bcin16g00810 | - | 138.1546364 | 292.1171117 | 1.080344111 | | 2.59848E-09 |
| Bcin05g01750 | - | 423.8670175 | 895.7430492 | 1.078913589 | | 1.02647E-20 |
| Bcin01g10180 | - | 1863.537947 | 3934.482423 | 1.077943475 | | 5.5461E-102 |
| Bcin13g02470 | Q8RXN0.1 RecName: Full=ABC transporter G family member 11; Short=ABC transporter ABCG.11; Short=AtABCG11; AltName: Full=Protein CUTICULAR DEFECT AND ORGAN FUSION 1; AltName: Full=Protein DESPERADO; AltName: Full=Protein PERMEABLE LEAVES 1; AltName: F | 22.6051305 | 47.67601622 | 1.074884296 | | 0.043778576 |
| novel.331 | - | 18.55257583 | 38.995894 | 1.071218446 | | 0.044584979 |
| Bcin07g04040 | O14031.1 RecName: Full=Glutathione transporter 1 | 28.39201557 | 59.66746854 | 1.070456049 | | 0.017341469 |
| novel.672 | - | 38.6368524 | 81.01661545 | 1.070433792 | | 0.005258726 |
| Bcin04g00180 | - | 1243.257091 | 2609.825344 | 1.069845885 | | 2.23373E-69 |
| Bcin03g01750 | O14123.1 RecName: Full=Probable Na(+)/H(+) antiporter C3A11.09 | 298.1625181 | 624.0434405 | 1.065536688 | | 2.57617E-17 |
| Bcin05g07350 | Q058N0.1 RecName: Full=IQ domain-containing protein IQM5; AltName: Full=IQ motif-containing protein 5 | 50.247382 | 105.0780177 | 1.065195179 | | 0.002075486 |
| Bcin03g07320 | Q4WXZ5.2 RecName: Full=Ribonuclease T2-like; Short=RNase T2-like; Flags: Precursor | 1425.656268 | 2981.700068 | 1.064351887 | | 1.16848E-78 |
| Bcin11g06260 | A7E727.1 RecName: Full=Mitochondrial outer membrane protein iml2 | 399.7083058 | 835.1878172 | 1.063586481 | | 5.91294E-23 |
| Bcin15g02750 | - | 49.62478978 | 103.6445786 | 1.061925954 | | 0.000532742 |
| Bcin04g01090 | - | 74.32556441 | 154.9038189 | 1.058806561 | | 1.76826E-05 |
| Bcin01g00650 | O14057.1 RecName: Full=Probable guanine deaminase; Short=Guanase; Short=Guanine aminase; AltName: Full=Guanine aminohydrolase; Short=GAH | 126.5699627 | 263.9007948 | 1.05820961 | | 8.1408E-07 |
| Bcin10g06080 | P40977.1 RecName: Full=1-phosphatidylinositol 4,5-bisphosphate phosphodiesterase 1; AltName: Full=Phospholipase C-1; Short=PLC-1 | 337.5271607 | 701.975682 | 1.056797835 | | 6.04077E-20 |
| Bcin10g05690 | - | 450.4431128 | 936.8296664 | 1.056561768 | | 1.44374E-27 |
| Bcin03g04690 | - | 953.6360541 | 1981.957289 | 1.055231123 | | 9.88429E-42 |
| Bcin09g01210 | P30583.3 RecName: Full=Chitin synthase C; AltName: Full=Chitin-UDP acetyl-glucosaminyl transferase C; AltName: Full=Class-I chitin synthase C | 264.7904451 | 549.6754776 | 1.054856263 | | 2.23164E-13 |
| Bcin11g05960 | - | 66.48932199 | 138.0196814 | 1.051129681 | | 0.002394663 |
| Bcin10g03880 | P40467.1 RecName: Full=Activator of stress genes 1 | 160.1869966 | 331.4236077 | 1.050193671 | | 1.65047E-08 |
| Bcin06g02530 | P39992.1 RecName: Full=Uncharacterized protein YEL023C | 553.4308805 | 1144.250988 | 1.047973554 | | 3.84715E-26 |
| Bcin02g07500 | - | 70.29666227 | 145.2742661 | 1.047260223 | | 7.18799E-05 |
| Bcin11g02920 | Q9VXG4.2 RecName: Full=Annexin B11 | 185.5622554 | 382.3758295 | 1.042852381 | | 4.45565E-11 |
| Bcin14g04550 | O14111.2 RecName: Full=Phosphatidylserine decarboxylase proenzyme 3; Contains: RecName: Full=Phosphatidylserine decarboxylase 3 beta chain; Contains: RecName: Full=Phosphatidylserine decarboxylase 3 alpha chain | 158.4963093 | 326.1371952 | 1.041626483 | | 6.85215E-09 |
| Bcin05g00860 | O52535.1 RecName: Full=Carbonic anhydrase; AltName: Full=Carbonate dehydratase; Flags: Precursor | 63.09677938 | 129.872202 | 1.041154539 | | 0.000173962 |
| Bcin03g04100 | - | 354.6071671 | 729.6840712 | 1.040653212 | | 6.16231E-18 |
| Bcin15g01880 | - | 95.39895192 | 195.9775646 | 1.03911439 | | 9.75264E-07 |
| Bcin12g00090 | B7SIW1.1 RecName: Full=Endo-1,4-beta-xylanase B; Short=Xylanase B; AltName: Full=1,4-beta-D-xylan xylanohydrolase B; Flags: Precursor | 49.62651069 | 101.9732953 | 1.037778708 | | 0.001010279 |
| Bcin06g04670 | - | 192.3532149 | 394.3676107 | 1.03692719 | | 2.40557E-09 |
| Bcin09g01320 | P0CT06.1 RecName: Full=Isocitrate lyase; Short=ICL; Short=Isocitrase; Short=Isocitratase; AltName: Full=Methylisocitrate lyase; Short=MICA; AltName: Full=Threo-D(S)-isocitrate glyoxylate-lyase | 8974.540483 | 18391.92206 | 1.035157034 | | 4.3711E-212 |
| Bcin15g02100 | C4ZGF5.1 RecName: Full=ATP-dependent Clp protease ATP-binding subunit ClpX | 113.9685168 | 233.5797134 | 1.035037895 | | 2.01265E-07 |
| Bcin13g05280 | - | 48.99054917 | 100.2398079 | 1.032322607 | | 0.001070107 |
| Bcin10g03530 | Q59QC7.1 RecName: Full=Sterol uptake control protein 2 | 2309.837998 | 4719.87987 | 1.030850456 | | 6.75932E-74 |
| Bcin16g01660 | O74446.2 RecName: Full=Sad1-interacting factor 2; AltName: Full=Sporulation protein sif2 | 193.7943565 | 396.1417949 | 1.030072711 | | 9.14141E-10 |
| Bcin05g04510 | Q7SEZ0.2 RecName: Full=U6 snRNA phosphodiesterase | 61.25473192 | 125.0553623 | 1.027420405 | | 0.001575522 |
| Bcin01g10400 | - | 48.68320403 | 99.23892406 | 1.027309788 | | 0.001174095 |
| Bcin07g06180 | P48777.2 RecName: Full=Purine permease | 94.97784648 | 193.4765167 | 1.026420362 | | 2.61062E-06 |
| Bcin05g06400 | - | 86.71118928 | 176.3800674 | 1.02573275 | | 1.2627E-05 |
| Bcin16g01000 | - | 325.911386 | 662.9955196 | 1.024916304 | | 8.21576E-17 |
| Bcin09g06830 | Q0D0A1.2 RecName: Full=PAB-dependent poly(A)-specific ribonuclease subunit pan3; AltName: Full=PAB1P-dependent poly(A)-nuclease; AltName: Full=PAN deadenylation complex subunit 3 | 143.198243 | 291.3748093 | 1.024683877 | | 4.44068E-08 |
| Bcin07g01220 | O60094.2 RecName: Full=DNA polymerase V; Short=POL V | 56.70208769 | 115.214758 | 1.022406715 | | 0.001916536 |
| Bcin11g05380 | - | 439.0637658 | 891.5067877 | 1.022066091 | | 1.20781E-22 |
| Bcin08g00870 | - | 37.57978544 | 75.96572383 | 1.016817902 | | 0.011310021 |
| Bcin03g01140 | - | 519.3457496 | 1050.818853 | 1.016662731 | | 8.72172E-29 |
| Bcin15g03720 | P54472.2 RecName: Full=GTP cyclohydrolase 1 type 2 homolog | 36.38746681 | 73.58271145 | 1.013686207 | | 0.013122774 |
| Bcin02g03400 | Q9UTC4.1 RecName: Full=Uncharacterized protein C227.15 | 359.0413729 | 725.3420843 | 1.013564696 | | 4.03952E-13 |
| Bcin13g03410 | - | 260.0318962 | 524.9098778 | 1.013486478 | | 8.60466E-16 |
| Bcin09g03190 | O94701.1 RecName: Full=Ingression protein fic1; AltName: Full=Cdc15-interacting C2 domain-containing protein 1 | 123.212154 | 248.0679671 | 1.011151527 | | 2.90988E-06 |
| Bcin01g06250 | B0Y665.1 RecName: Full=Lysophospholipase 1; AltName: Full=Phospholipase B 1; Flags: Precursor >P0C957.1 RecName: Full=Lysophospholipase 1; AltName: Full=Phospholipase B 1; Flags: Precursor | 697.2088575 | 1405.334347 | 1.011055374 | | 2.82437E-20 |
| Bcin16g04250 | - | 93.29346481 | 188.0788059 | 1.009582432 | | 5.88837E-05 |
| Bcin16g00090 | - | 204.0128549 | 410.7119683 | 1.009523421 | | 1.34927E-11 |
| novel.570 | - | 40.72195093 | 81.87960552 | 1.008881513 | | 0.007253973 |
| Bcin15g01130 | O66761.1 RecName: Full=Putative L-lysine 2,3-aminomutase aq_454; Short=LAM | 96.83538216 | 194.577548 | 1.007286372 | | 6.26045E-06 |
| Bcin06g06480 | Q9SJJ3.1 RecName: Full=BEL1-like homeodomain protein 8; Short=BEL1-like protein 8; AltName: Full=Protein POUND-FOOLISH | 167.4980384 | 336.0492067 | 1.005589297 | | 4.9711E-08 |
| Bcin12g03340 | Q55GW8.2 RecName: Full=AN1-type zinc finger and UBX domain-containing protein DDB_G0268260 | 501.3256603 | 1005.963183 | 1.00459446 | | 1.73849E-23 |
| Bcin10g06060 | - | 2890.500069 | 1444.36579 | -1.000641136 | | 6.87E-55 |
| Bcin01g09450 | Q0QWS4.1 RecName: Full=L-galactonate dehydratase | 12335.943 | 6162.539731 | -1.001079446 | | 2.83E-117 |
| Bcin01g05280 | P37967.2 RecName: Full=Para-nitrobenzyl esterase; AltName: Full=Intracellular esterase B; AltName: Full=PNB carboxy-esterase; Short=PNBCE | 2083.199248 | 1040.322751 | -1.001558066 | | 2.76E-54 |
| Bcin03g05720 | - | 2188.645212 | 1089.859589 | -1.005748347 | | 2.86E-57 |
| Bcin04g05310 | Q10423.1 RecName: Full=Cruciform cutting endonuclease 1, mitochondrial; AltName: Full=Protein ydc2; Flags: Precursor | 85.29682845 | 42.39585076 | -1.008307577 | | 0.036583377 |
| Bcin07g05480 | - | 125.8186133 | 62.53226208 | -1.009124596 | | 0.001927286 |
| Bcin09g04790 | - | 4956.224185 | 2461.977291 | -1.009348056 | | 3.37E-113 |
| Bcin10g06130 | A2QG68.2 RecName: Full=Alpha-L-rhamnosidase rgxB; AltName: Full=Exopolygalacturonase B; AltName: Full=Exorhamnogalacturonase B; AltName: Full=Pnp-rhamnohydrolase; Flags: Precursor | 8022.143443 | 3984.174116 | -1.009688542 | | 2.65E-131 |
| Bcin13g00190 | - | 95.08251972 | 47.18154983 | -1.010246083 | | 0.008244672 |
| Bcin15g03370 | Q872T7.1 RecName: Full=4-hydroxyphenylpyruvate dioxygenase; Short=4HPPD; Short=HPD; Short=HPPDase | 6306.425641 | 3130.421569 | -1.010407862 | | 4.71E-121 |
| Bcin06g04140 | P49374.1 RecName: Full=High-affinity glucose transporter | 433.5319359 | 214.9496774 | -1.011099548 | | 2.4E-10 |
| Bcin09g05520 | P42938.1 RecName: Full=Probable ATP-dependent kinase TDA10; AltName: Full=Topoisomerase I damage affected protein 10 | 474.5183047 | 235.2640107 | -1.01134152 | | 3.91E-11 |
| Bcin05g03580 | - | 2994.088149 | 1482.78307 | -1.014089661 | | 2.45E-59 |
| Bcin01g05180 | - | 157.7942906 | 77.65244027 | -1.021139298 | | 0.000150043 |
| Bcin11g02630 | Q0C8A0.1 RecName: Full=Dioxygenase trt7; AltName: Full=Terretonin synthesis protein 7 | 1016.041403 | 500.6414018 | -1.02140365 | | 5.65E-23 |
| novel.123 | - | 176.598124 | 86.84930068 | -1.025092453 | | 0.0000306 |
| Bcin09g05970 | - | 441.3395743 | 216.6849924 | -1.025641452 | | 1.04E-09 |
| Bcin04g06230 | Q7TTP0.1 RecName: Full=Flavohemoprotein; AltName: Full=Flavohemoglobin; AltName: Full=Hemoglobin-like protein; AltName: Full=Nitric oxide dioxygenase; Short=NO oxygenase; Short=NOD | 323.8319728 | 159.0748542 | -1.026028211 | | 3.57E-09 |
| Bcin08g03830 | Q9LBG2.1 RecName: Full=Levodione reductase; AltName: Full=(6R)-2,2,6-trimethyl-1,4-cyclohexanedione reductase | 11642.30033 | 5711.382262 | -1.027367661 | | 2.69E-140 |
| Bcin16g02990 | - | 440.402711 | 215.9458432 | -1.028963911 | | 8.03E-12 |
| Bcin13g00200 | B0XQS8.1 RecName: Full=Probable quinate permease; AltName: Full=Quinate transporter | 1064.793668 | 521.5149734 | -1.029197941 | | 9.92E-26 |
| novel.268 | - | 189.7509422 | 92.706425 | -1.033281266 | | 0.00000142 |
| Bcin01g05770 | - | 67.24072248 | 32.7687528 | -1.034861574 | | 0.012171072 |
| Bcin03g02290 | - | 140.8453381 | 68.48644621 | -1.039903122 | | 0.0000684 |
| Bcin05g03240 | Q92253.2 RecName: Full=Probable glucose transporter rco-3 | 40.61679519 | 19.7649971 | -1.040303358 | | 0.046433863 |
| Bcin01g02490 | P37769.2 RecName: Full=2-dehydro-3-deoxy-D-gluconate 5-dehydrogenase; AltName: Full=2-deoxy-D-gluconate 3-dehydrogenase; AltName: Full=2-keto-3-deoxygluconate 5-dehydrogenase; AltName: Full=2-keto-3-deoxygluconate oxidoreductase; Short=KDG oxidoreduc | 4295.830757 | 2087.169198 | -1.041505614 | | 3.26E-99 |
| Bcin07g05940 | - | 120.9012558 | 58.4644979 | -1.045768894 | | 0.001069792 |
| Bcin16g01490 | Q4WMJ0.1 RecName: Full=Cytochrome P450 monooxygenase gliF; AltName: Full=Gliotoxin biosynthesis protein F | 5547.342931 | 2686.260379 | -1.046284997 | | 2.93E-100 |
| Bcin03g08110 | O14434.1 RecName: Full=Scytalone dehydratase arp1; AltName: Full=Conidial pigment biosynthesis oxidase arp1 | 490.6178048 | 236.7311467 | -1.050335287 | | 1.84E-12 |
| novel.69 | - | 77.74714242 | 37.59534589 | -1.050678962 | | 0.008499625 |
| Bcin03g00480 | Q2LMP0.1 RecName: Full=Endo-1,4-beta-xylanase 11A; Short=Xylanase 11A; AltName: Full=1,4-beta-D-xylan xylanohydrolase 11A; Flags: Precursor | 36760.43271 | 17727.7977 | -1.052201764 | | 0 |
| Bcin05g06510 | Q01738.1 RecName: Full=Cellobiose dehydrogenase; Short=CDH; AltName: Full=Cellobiose-quinone oxidoreductase; Flags: Precursor | 58.36846206 | 28.11622945 | -1.054575989 | | 0.016784457 |
| Bcin06g04290 | - | 105.6188978 | 50.81164626 | -1.056954122 | | 0.001149248 |
| Bcin05g07630 | - | 187.1974332 | 89.80358671 | -1.061228987 | | 0.0000329 |
| Bcin07g04030 | - | 54.66835418 | 26.13939111 | -1.062946173 | | 0.021156705 |
| Bcin14g03170 | P80402.2 RecName: Full=2,3-dihydroxybenzoate decarboxylase; Short=2,3-DHBA decarboxylase; Short=DHBD; AltName: Full=o-pyrocatechuate decarboxylase | 627.6006268 | 299.8836152 | -1.065662486 | | 3.89E-15 |
| Bcin03g01580 | O16171.1 RecName: Full=Esterase-5C; Short=Est-5C; AltName: Full=Carboxylic-ester hydrolase 5C; Short=Carboxylesterase-5C; Flags: Precursor | 266.119285 | 127.2434851 | -1.06582202 | | 2.52E-08 |
| novel.274 | - | 44.01790012 | 21.01626769 | -1.066669574 | | 0.036524824 |
| Bcin01g06910 | - | 211.4939959 | 100.7853277 | -1.070226444 | | 0.00000722 |
| Bcin16g05198 | Q6Q887.1 RecName: Full=Probable aminotransferase sirI; AltName: Full=Sirodesmin biosynthesis protein I | 139.6237639 | 66.50266793 | -1.071945131 | | 0.000101482 |
| Bcin08g03760 | P11838.2 RecName: Full=Endothiapepsin; AltName: Full=Aspartate protease; Flags: Precursor | 259.8098941 | 123.4890405 | -1.073960944 | | 0.000000532 |
| Bcin07g03780 | A1D2R3.1 RecName: Full=Probable quinate permease; AltName: Full=Quinate transporter | 5717.88097 | 2713.382849 | -1.075338735 | | 5.41E-123 |
| Bcin16g02560 | Q2U4L7.2 RecName: Full=Glutaminase A; Flags: Precursor | 786.2836884 | 372.516603 | -1.077050285 | | 1.66E-23 |
| Bcin03g02670 | O14361.2 RecName: Full=Putative prephenate dehydratase; Short=PDT | 2128.943483 | 1007.879507 | -1.078687595 | | 4.24E-63 |
| novel.363 | - | 114.1736655 | 54.00822021 | -1.079994909 | | 0.00028752 |
| Bcin03g08130 | P86029.1 RecName: Full=Catechol 1,2-dioxygenase | 550.1026341 | 259.838143 | -1.08195293 | | 2.02E-17 |
| Bcin06g05560 | - | 1722.610286 | 813.0967489 | -1.082676944 | | 5.22E-45 |
| Bcin03g01920 | P55306.1 RecName: Full=Catalase | 7204.080944 | 3399.031889 | -1.083524712 | | 1.35E-142 |
| Bcin09g04440 | B8NKA3.2 RecName: Full=Probable alpha-L-arabinofuranosidase A; Short=ABF A; Short=Arabinosidase A; Flags: Precursor | 3288.619969 | 1550.769615 | -1.084044027 | | 5.98E-74 |
| Bcin01g03900 | - | 1930.755024 | 908.40726 | -1.087375977 | | 7.8E-47 |
| Bcin05g00100 | - | 92.55446647 | 43.47836124 | -1.088982961 | | 0.001531152 |
| Bcin16g03950 | Q0CMT2.1 RecName: Full=Probable 1,4-beta-D-glucan cellobiohydrolase B; AltName: Full=Beta-glucancellobiohydrolase B; AltName: Full=Exocellobiohydrolase B; AltName: Full=Exoglucanase B; Flags: Precursor | 71156.28899 | 33359.3738 | -1.092881201 | | 0 |
| Bcin01g11040 | - | 436.7818206 | 204.6149653 | -1.094169662 | | 1.94E-13 |
| Bcin03g01500 | - | 4342.664108 | 2033.365221 | -1.094855669 | | 2.46E-113 |
| Bcin03g05810 | - | 431.3991343 | 201.7748026 | -1.096074683 | | 1.55E-14 |
| Bcin03g03560 | O00058.1 RecName: Full=Probable NADP-dependent mannitol dehydrogenase; Short=MtDH; AltName: Full=Mannitol 2-dehydrogenase [NADP(+)]; AltName: Full=Planta-induced rust protein 8 | 1648.956004 | 770.0912813 | -1.0981518 | | 1.33E-38 |
| Bcin01g04540 | O74631.1 RecName: Full=Protein FDD123; AltName: Full=CvHSP30/1 | 197.9875035 | 92.46061917 | -1.099092402 | | 0.000000643 |
| Bcin04g04680 | P40397.2 RecName: Full=Uncharacterized oxidoreductase YhxC; AltName: Full=ORFX | 152.9563574 | 71.36146463 | -1.099526766 | | 0.0000151 |
| Bcin16g01820 | - | 51.92708343 | 24.13410553 | -1.102963371 | | 0.02795627 |
| Bcin01g08160 | - | 329.1067642 | 153.242462 | -1.103330238 | | 8.63E-11 |
| Bcin02g07770 | - | 4337.633032 | 2015.665276 | -1.105412667 | | 5.45E-113 |
| Bcin12g02830 | Q2UNR0.1 RecName: Full=Probable beta-glucosidase D; AltName: Full=Beta-D-glucoside glucohydrolase D; AltName: Full=Cellobiase D; AltName: Full=Gentiobiase D; Flags: Precursor | 655.4626748 | 304.4033985 | -1.105828192 | | 3.07E-16 |
| Bcin12g03000 | - | 795.5436855 | 369.2781321 | -1.107675081 | | 3.1E-24 |
| Bcin12g04870 | Q9P3V5.1 RecName: Full=Uncharacterized transporter C1348.05 | 1280.164211 | 592.4320876 | -1.111633993 | | 3.52E-40 |
| Bcin04g01410 | - | 12472.85902 | 5764.586713 | -1.11344901 | | 2.31E-132 |
| Bcin05g05020 | Q9UQY0.2 RecName: Full=Demethylsterigmatocystin 6-O-methyltransferase; AltName: Full=Aflatoxin biosynthesis protein O; AltName: Full=Methyltransferase B; AltName: Full=O-methyltransferase I; Short=mt-I | 99.32433474 | 45.74063921 | -1.116669908 | | 0.00449852 |
| Bcin11g06070 | - | 42.76773082 | 19.73399364 | -1.11763831 | | 0.032992995 |
| novel.305 | - | 55.34473712 | 25.46006281 | -1.118719272 | | 0.042943737 |
| Bcin14g04580 | Q4R0J7.1 RecName: Full=D-arabinitol dehydrogenase 1; AltName: Full=NADP-dependent D-arabitol dehydrogenase | 23473.93521 | 10801.91188 | -1.119750995 | | 0 |
| Bcin05g01660 | P29717.4 RecName: Full=Glucan 1,3-beta-glucosidase; AltName: Full=Exo-1,3-beta-glucanase; Flags: Precursor | 5079.187201 | 2336.852126 | -1.120076006 | | 1.73E-109 |
| Bcin07g04120 | - | 45.80597899 | 21.08136235 | -1.120490222 | | 0.027371713 |
| Bcin01g08600 | - | 368.498679 | 169.3043719 | -1.121968437 | | 7.39E-12 |
| Bcin01g03400 | W7N2B4.2 RecName: Full=Efflux pump FUB11; AltName: Full=Fusaric acid biosynthesis protein 11 | 1104.429685 | 506.4583966 | -1.124730993 | | 4.73E-27 |
| Bcin12g01950 | A7MBI7.1 RecName: Full=Catechol O-methyltransferase | 107.2528905 | 49.035336 | -1.128003954 | | 0.002426398 |
| Bcin12g01620 | - | 68.50019897 | 31.23449747 | -1.132107939 | | 0.004989257 |
| Bcin16g01630 | - | 105.6489937 | 48.15707415 | -1.13417537 | | 0.000331558 |
| Bcin06g07600 | - | 69.67897042 | 31.75080547 | -1.135277739 | | 0.003379844 |
| Bcin09g06990 | B8N5S6.1 RecName: Full=Probable beta-glucosidase M; AltName: Full=Beta-D-glucoside glucohydrolase M; AltName: Full=Cellobiase M; AltName: Full=Gentiobiase M; Flags: Precursor | 988.7015135 | 449.8413123 | -1.135580957 | | 3.04E-30 |
| Bcin03g01490 | A6Y9S5.1 RecName: Full=L-threo-3-deoxy-hexylosonate aldolase; AltName: Full=L-threo-3-deoxy-hexulosonate aldolase | 16315.86016 | 7425.38756 | -1.135693742 | | 0 |
| Bcin07g04920 | Q9USW3.1 RecName: Full=Probable glycosidase C21B10.07 | 334.416812 | 152.2116377 | -1.13580911 | | 1.77E-12 |
| Bcin14g00080 | - | 44.91068002 | 20.44986802 | -1.137611828 | | 0.025085324 |
| Bcin07g05460 | - | 165.1594255 | 74.86233342 | -1.140826814 | | 0.00000246 |
| Bcin07g02730 | P49426.1 RecName: Full=Glucan 1,3-beta-glucosidase; AltName: Full=1,3-beta-D-glucanohydrolase; AltName: Full=Exo-beta 1,3 glucanase; Flags: Precursor | 3206.038302 | 1453.193738 | -1.141392148 | | 4.41E-78 |
| Bcin12g01070 | A6RSP5.1 RecName: Full=Kynureninase; AltName: Full=Biosynthesis of nicotinic acid protein 5; AltName: Full=L-kynurenine hydrolase | 2824.627928 | 1279.550091 | -1.142765825 | | 8.23E-76 |
| Bcin06g04950 | Q99385.1 RecName: Full=Vacuolar calcium ion transporter; AltName: Full=High copy number undoes manganese protein 1; AltName: Full=Manganese resistance 1 protein; AltName: Full=Vacuolar Ca(2+)/H(+) exchanger | 133.3321069 | 60.28395988 | -1.14405016 | | 0.0000291 |
| Bcin06g04270 | - | 1342.293549 | 606.3380127 | -1.145720211 | | 8.65E-36 |
| Bcin16g04150 | - | 251.4086477 | 113.2703805 | -1.151874288 | | 0.000000224 |
| Bcin09g02260 | D4AK17.1 RecName: Full=PI-PLC X domain-containing protein 1; Flags: Precursor | 656.2288186 | 295.2862602 | -1.152509266 | | 4.04E-19 |
| Bcin03g04010 | Q2UPQ4.1 RecName: Full=Probable endo-beta-1,4-glucanase B; Short=Endoglucanase B; AltName: Full=Carboxymethylcellulase B; AltName: Full=Cellulase B; Flags: Precursor >B8MW97.1 RecName: Full=probable endo-beta-1,4-glucanase B; Short=Endoglucanase B; A | 25938.67284 | 11667.50464 | -1.152608571 | | 0 |
| Bcin12g00040 | Q12559.2 RecName: Full=Acetamidase | 106.9656183 | 48.01825449 | -1.154135534 | | 0.000193635 |
| Bcin03g01980 | Q5BEK1.1 RecName: Full=Asperfuranone cluster transcription factor afoA; AltName: Full=Asperfuranone biosynthesis protein A | 47.87090905 | 21.45407257 | -1.154980962 | | 0.027816495 |
| Bcin09g04800 | - | 59.90232396 | 26.92291637 | -1.155096876 | | 0.021669101 |
| Bcin03g05670 | - | 202.0518844 | 90.6431819 | -1.156007226 | | 5.23E-08 |
| Bcin07g06820 | - | 2490.31508 | 1115.125705 | -1.158640234 | | 2.56E-62 |
| Bcin05g03080 | Q4IPH4.1 RecName: Full=Peptidyl-prolyl cis-trans isomerase H; Short=PPIase H; AltName: Full=Rotamase H | 1044.068405 | 466.4151051 | -1.163271641 | | 1.47E-32 |
| Bcin09g00460 | - | 4425.496223 | 1968.510937 | -1.168887323 | | 1.95E-114 |
| novel.238 | - | 39.28852227 | 17.32117793 | -1.178052715 | | 0.038227548 |
| Bcin15g05080 | P0C7S9.1 RecName: Full=1,3-beta-glucanosyltransferase gel1; AltName: Full=Glucan elongating glucanosyltransferase 1; Flags: Precursor | 1624.717345 | 716.6084896 | -1.180906828 | | 1.57E-50 |
| Bcin13g05760 | Q00298.1 RecName: Full=Cutinase; AltName: Full=Cutin hydrolase; Flags: Precursor | 6292.495658 | 2772.260342 | -1.182706927 | | 9.64E-177 |
| Bcin03g00080 | - | 158.4211117 | 69.77074498 | -1.183377403 | | 0.000000903 |
| Bcin04g00780 | - | 407.5199307 | 178.744386 | -1.1885884 | | 1.85E-10 |
| Bcin03g00230 | C5CZC3.1 RecName: Full=D-galactonate dehydratase; Short=GalD | 5556.161385 | 2427.912061 | -1.194159848 | | 5.33E-166 |
| Bcin10g01150 | - | 56.16655941 | 24.49327017 | -1.196114477 | | 0.007580679 |
| Bcin03g05200 | D4AZ24.1 RecName: Full=Probable endo-1,3(4)-beta-glucanase ARB_01444; Short=Endo-1,3-beta-glucanase; Short=Endo-1,4-beta-glucanase; AltName: Full=Laminarinase; Flags: Precursor | 343.6641112 | 149.9897223 | -1.197027006 | | 2.4E-11 |
| Bcin01g00250 | - | 152.5047158 | 66.45058735 | -1.197893015 | | 0.00000433 |
| novel.312 | - | 75.29592318 | 32.73080939 | -1.202625464 | | 0.002620344 |
| Bcin12g00030 | - | 1531.061935 | 663.4123433 | -1.206735333 | | 3.05E-49 |
| Bcin01g05680 | Q0D076.1 RecName: Full=Probable mannosyl-oligosaccharide alpha-1,2-mannosidase 1B; AltName: Full=Class I alpha-mannosidase 1B; AltName: Full=Man(9)-alpha-mannosidase 1B; Flags: Precursor | 1687.043356 | 729.9190606 | -1.208981675 | | 1.34E-50 |
| Bcin15g03080 | Q00298.1 RecName: Full=Cutinase; AltName: Full=Cutin hydrolase; Flags: Precursor | 9517.371106 | 4106.235112 | -1.212742806 | | 1.06E-189 |
| Bcin06g03600 | - | 52.37382468 | 22.58152649 | -1.213357533 | | 0.018066358 |
| Bcin12g02360 | - | 73.29303048 | 31.56627788 | -1.214402579 | | 0.004210698 |
| Bcin14g05330 | - | 230.3488074 | 99.10392083 | -1.217876937 | | 0.000000014 |
| Bcin03g00900 | O13752.1 RecName: Full=Uncharacterized TLC domain-containing protein C17A2.02c | 362.478408 | 155.6040988 | -1.218128409 | | 6.76E-08 |
| novel.951 | - | 144.1546143 | 61.90939809 | -1.220742444 | | 0.00000944 |
| Bcin10g00310 | D4B0V1.1 RecName: Full=Probable glucan endo-1,3-beta-glucosidase ARB_02077; AltName: Full=(1->3)-beta-glucan endohydrolase ARB_02077; Short=(1->3)-beta-glucanase ARB_02077; Flags: Precursor | 3236.449256 | 1387.817228 | -1.221185661 | | 3.75E-98 |
| Bcin08g02100 | Q10DK7.1 RecName: Full=1-aminocyclopropane-1-carboxylate synthase 1; Short=ACC synthase 1; AltName: Full=S-adenosyl-L-methionine methylthioadenosine-lyase 1 >A2XLL2.2 RecName: Full=1-aminocyclopropane-1-carboxylate synthase 1; Short=ACC synthase 1; A | 72.93149662 | 31.14626519 | -1.229714849 | | 0.002881191 |
| Bcin07g00890 | Q12713.1 RecName: Full=Endochitinase 33; AltName: Full=33 kDa endochitinase; AltName: Full=Chitinase 33; Flags: Precursor | 1515.552016 | 646.0363314 | -1.23006152 | | 2.28E-47 |
| Bcin15g02440 | B0YB65.1 RecName: Full=Probable beta-glucosidase L; AltName: Full=Beta-D-glucoside glucohydrolase L; AltName: Full=Cellobiase L; AltName: Full=Gentiobiase L; Flags: Precursor | 955.0406274 | 406.5783092 | -1.232950393 | | 5.51E-31 |
| Bcin01g06380 | - | 441.5746411 | 187.9212323 | -1.233624367 | | 3.04E-15 |
| Bcin13g02180 | - | 86.72495658 | 36.78848582 | -1.236226445 | | 0.000335309 |
| Bcin02g05630 | - | 54.83656557 | 23.2267636 | -1.238400761 | | 0.005056691 |
| Bcin15g01690 | P37694.2 RecName: Full=Ketoacyl reductase HetN | 179.4067305 | 75.79151697 | -1.24222567 | | 0.000000681 |
| Bcin09g05570 | O04036.3 RecName: Full=Sugar transporter ERD6; AltName: Full=Early-responsive to dehydration protein 6; AltName: Full=Sugar transporter-like protein 1 | 5230.149337 | 2187.114733 | -1.258055894 | | 3.69E-172 |
| Bcin14g00860 | P17872.1 RecName: Full=Pectinesterase; Short=PE; AltName: Full=Pectin methylesterase; Flags: Precursor | 6243.431219 | 2603.68075 | -1.261587013 | | 4.85E-193 |
| Bcin05g08230 | Q2US83.1 RecName: Full=Probable endo-beta-1,4-glucanase D; Short=Endoglucanase D; AltName: Full=Carboxymethylcellulase D; AltName: Full=Cellulase D; Flags: Precursor >B8MXJ7.1 RecName: Full=Probable endo-beta-1,4-glucanase D; Short=Endoglucanase D; A | 214.1034145 | 89.23240892 | -1.263623678 | | 0.000000197 |
| Bcin03g04480 | A0A097ZPE8.1 RecName: Full=Short chain dehydrogenase andI; AltName: Full=Anditomin synthesis protein I | 5801.605124 | 2409.681976 | -1.26770549 | | 7.42E-203 |
| Bcin03g01680 | Q4WR80.1 RecName: Full=Probable endopolygalacturonase AFUA_1G17220; AltName: Full=Pectinase AFUA_1G17220; AltName: Full=Polygalacturonase AFUA_1G17220; Flags: Precursor >B0XPA1.1 RecName: Full=Probable endopolygalacturonase AFUB_016610; AltName: Full | 7570.633196 | 3143.735902 | -1.267902566 | | 1.32E-241 |
| Bcin16g01810 | - | 45.08189298 | 18.73769072 | -1.268016211 | | 0.013212483 |
| Bcin05g06610 | P39932.2 RecName: Full=Sugar transporter STL1 | 74.66988914 | 30.84987198 | -1.272974244 | | 0.000818066 |
| Bcin15g01990 | - | 254.7523 | 105.3165906 | -1.273960952 | | 9.6E-11 |
| novel.1528 | - | 79.69178429 | 32.85004513 | -1.277675724 | | 0.001994147 |
| novel.1302 | - | 36.8012061 | 15.12751253 | -1.279408937 | | 0.043785326 |
| Bcin12g00350 | - | 59.05022786 | 24.31202749 | -1.282407346 | | 0.004497282 |
| novel.1042 | - | 43.49741982 | 17.85985906 | -1.28533742 | | 0.024352686 |
| Bcin12g01060 | P47125.1 RecName: Full=Indoleamine 2,3-dioxygenase; Short=IDO; AltName: Full=Biosynthesis of nicotinic acid protein 2 | 1097.671172 | 448.8138688 | -1.290365937 | | 2.66E-40 |
| Bcin05g05420 | - | 155.9506067 | 63.75306071 | -1.291877962 | | 0.000000407 |
| Bcin15g03380 | - | 1355.574987 | 551.3206727 | -1.297620251 | | 5.69E-51 |
| Bcin01g03910 | - | 3676.715873 | 1493.798891 | -1.299609418 | | 4.11E-120 |
| Bcin01g07330 | A2QBB6.1 RecName: Full=Probable endopolygalacturonase E; Short=PGE; AltName: Full=Pectinase 4; AltName: Full=Pectinase E; AltName: Full=Polygalacturonase E; AltName: Full=Polygalacturonase IV; Short=PG-IV; Flags: Precursor | 32884.91504 | 13276.18726 | -1.30848218 | | 0 |
| Bcin13g02320 | O94218.1 RecName: Full=Xyloglucan-specific endo-beta-1,4-glucanase A; AltName: Full=Xyloglucanase A; AltName: Full=Xyloglucanendohydrolase A; Flags: Precursor | 16083.86481 | 6487.399102 | -1.309915892 | | 2.31E-279 |
| Bcin08g04590 | - | 1131.60852 | 453.6048775 | -1.319170953 | | 1.35E-48 |
| Bcin03g03200 | - | 175.0419726 | 70.19297956 | -1.319551114 | | 0.000000188 |
| novel.865 | - | 51.6458566 | 20.72774033 | -1.319742845 | | 0.016202847 |
| Bcin13g03660 | - | 154.1775336 | 61.62067354 | -1.32251905 | | 0.000000155 |
| novel.663 | - | 56.11947447 | 22.18827066 | -1.336743612 | | 0.00512085 |
| Bcin01g07210 | D4ATR3.1 RecName: Full=Uncharacterized secreted glycosidase ARB_07629; Flags: Precursor | 149.5561351 | 58.89845055 | -1.343175661 | | 0.00000167 |
| Bcin03g01970 | - | 365.3608358 | 143.2371112 | -1.351297583 | | 1.02E-16 |
| Bcin05g02950 | - | 3011.983654 | 1179.735706 | -1.352519035 | | 1.33E-124 |
| Bcin13g03390 | - | 886.8219488 | 346.4365255 | -1.356658903 | | 1.27E-35 |
| Bcin06g03730 | - | 472.9635163 | 184.3902259 | -1.35878574 | | 5.5E-15 |
| Bcin05g02270 | - | 32.58908685 | 12.72608058 | -1.360202249 | | 0.042165107 |
| Bcin03g01960 | - | 187.1540102 | 72.65482392 | -1.36519424 | | 0.000000188 |
| Bcin06g03160 | D4B5D4.1 RecName: Full=Cell surface Cu-only superoxide dismutase ARB_03674; Flags: Precursor | 333.0401334 | 128.8007767 | -1.3703576 | | 4.48E-12 |
| novel.1466 | - | 76.79386604 | 29.6397284 | -1.371662776 | | 0.002639599 |
| Bcin07g06480 | Q99034.1 RecName: Full=Acetylxylan esterase; Flags: Precursor | 135.6426071 | 52.33365201 | -1.373971669 | | 0.000000288 |
| Bcin06g01600 | - | 8785.431041 | 3383.861416 | -1.376541547 | | 6.18E-241 |
| Bcin06g03740 | - | 663.0077761 | 254.7590972 | -1.380721967 | | 3.8E-25 |
| novel.112 | - | 76.15410693 | 29.08046587 | -1.38993638 | | 0.00027037 |
| Bcin13g02270 | - | 32.24286333 | 12.2677301 | -1.395423768 | | 0.036599588 |
| Bcin03g05660 | - | 73.94726167 | 28.06145552 | -1.401803105 | | 0.003438978 |
| Bcin02g07700 | A2QT85.1 RecName: Full=Probable arabinan endo-1,5-alpha-L-arabinosidase A; AltName: Full=Endo-1,5-alpha-L-arabinanase A; Short=ABN A; Flags: Precursor | 2297.933412 | 861.0839344 | -1.415701672 | | 8.15E-79 |
| novel.410 | - | 46.45570782 | 17.30136103 | -1.420446321 | | 0.006984326 |
| Bcin12g01100 | Q00001.1 RecName: Full=Rhamnogalacturonase A; Short=RGase A; Short=RHG A; AltName: Full=Rhamnogalacturonan hydrolase A; Flags: Precursor | 4314.655066 | 1611.180843 | -1.420883533 | | 2.02E-166 |
| Bcin03g01220 | - | 133.693116 | 49.79884716 | -1.422031489 | | 0.0000784 |
| Bcin06g02290 | - | 99.95141827 | 37.24992404 | -1.425843611 | | 0.000334955 |
| Bcin05g03410 | P42106.2 RecName: Full=Quercetin 2,3-dioxygenase; Short=Quercetinase; AltName: Full=Flavonol 2,4-dioxygenase | 856.2734036 | 318.7635118 | -1.425872891 | | 2.64E-37 |
| Bcin01g11290 | Q05031.1 RecName: Full=Mannan endo-1,6-alpha-mannosidase DFG5; AltName: Full=Endo-alpha-1->6-D-mannanase DFG5; Flags: Precursor | 147.1219472 | 54.38956077 | -1.433774892 | | 0.00000012 |
| Bcin14g04260 | - | 2741.998126 | 1002.014015 | -1.452581286 | | 1.91E-104 |
| Bcin08g06160 | P83692.1 RecName: Full=Arabinogalactan endo-beta-1,4-galactanase; AltName: Full=Endo-1,4-beta-galactanase; Short=Galactanase | 3620.627328 | 1307.160936 | -1.469734051 | | 2.03E-139 |
| Bcin03g01520 | P42328.1 RecName: Full=Alcohol dehydrogenase; AltName: Full=ADH-HT | 714.2497787 | 257.7823301 | -1.471066481 | | 1.34E-31 |
| Bcin02g07310 | Q4WDN4.1 RecName: Full=Probable aspartic-type endopeptidase opsB; Flags: Precursor | 76.88031186 | 27.67055872 | -1.471522247 | | 0.000780907 |
| Bcin06g04940 | O74628.1 RecName: Full=Uncharacterized oxidoreductase C162.03 | 22010.60082 | 7931.343018 | -1.472626291 | | 0 |
| novel.768 | - | 32.4394075 | 11.70537984 | -1.473784686 | | 0.028061131 |
| Bcin01g08620 | P80402.2 RecName: Full=2,3-dihydroxybenzoate decarboxylase; Short=2,3-DHBA decarboxylase; Short=DHBD; AltName: Full=o-pyrocatechuate decarboxylase | 2561.007843 | 909.3042945 | -1.494246262 | | 2.29E-87 |
| novel.607 | - | 33.7747842 | 11.88821705 | -1.504060197 | | 0.017756815 |
| Bcin05g01800 | P14306.2 RecName: Full=Carboxypeptidase Y inhibitor; Short=CPY inhibitor; AltName: Full=CDC25 suppressor 1; AltName: Full=I(C); AltName: Full=Ic; AltName: Full=Protein DKA1; AltName: Full=Protein NSP1 | 454.4554261 | 158.92827 | -1.514848212 | | 4.41E-22 |
| novel.557 | - | 26.6684509 | 9.288514725 | -1.517727856 | | 0.032203525 |
| Bcin15g04750 | P24458.1 RecName: Full=Cytochrome P450 52A3-B; Short=CYP52A3-B; AltName: Full=Alkane-inducible P450-ALK1-B; AltName: Full=CYPLIIA3 | 132.6321137 | 46.15019404 | -1.523067737 | | 0.000000547 |
| Bcin08g06230 | - | 28.26960846 | 9.704675186 | -1.541528825 | | 0.026395491 |
| Bcin15g04770 | - | 391.8446644 | 134.122702 | -1.546664123 | | 3.48E-20 |
| Bcin06g06410 | G2QJ27.1 RecName: Full=Acetylesterase; AltName: Full=Carbohydrate esterase family 16 protein; Flags: Precursor | 2511.434543 | 845.3976248 | -1.570691954 | | 1.92E-92 |
| Bcin10g04860 | - | 27.76916098 | 9.326123826 | -1.571936489 | | 0.041327659 |
| Bcin08g06830 | B8NJF4.2 RecName: Full=Probable beta-glucosidase D; AltName: Full=Beta-D-glucoside glucohydrolase D; AltName: Full=Cellobiase D; AltName: Full=Gentiobiase D; Flags: Precursor | 1352.495697 | 452.7620629 | -1.579867184 | | 1.66E-59 |
| Bcin15g01700 | E9R876.1 RecName: Full=MFS gliotoxin efflux transporter gliA; AltName: Full=Gliotoxin biosynthesis protein A | 82.87353293 | 27.45064393 | -1.594832913 | | 0.0000412 |
| Bcin03g03480 | Q96VB6.1 RecName: Full=Endo-1,4-beta-xylanase F3; Short=Xylanase F3; AltName: Full=1,4-beta-D-xylan xylanohydrolase F3; Flags: Precursor | 1042.897397 | 342.832857 | -1.604939038 | | 2.01E-59 |
| novel.1553 | - | 48.26275897 | 15.83422506 | -1.608641484 | | 0.0007632 |
| Bcin14g03960 | - | 28.7114071 | 9.388662267 | -1.613947747 | | 0.031313542 |
| novel.1361 | - | 59.69179237 | 19.45356511 | -1.620265926 | | 0.00022722 |
| Bcin08g02050 | - | 58.97905013 | 19.1365905 | -1.626357304 | | 0.00119837 |
| Bcin14g05510 | Q5B9Z8.2 RecName: Full=Probable alpha-L-arabinofuranosidase axhA-1; AltName: Full=Arabinoxylan arabinofuranohydrolase axhA-1; Flags: Precursor | 1568.156252 | 507.4609053 | -1.626997083 | | 6.21E-66 |
| Bcin15g04760 | - | 179.0834992 | 57.98416867 | -1.628440181 | | 8.13E-10 |
| Bcin03g06930 | - | 196.5819674 | 63.24259385 | -1.637789696 | | 8.19E-10 |
| Bcin16g04060 | Q4X084.1 RecName: Full=Probable endo-1,3(4)-beta-glucanase AFUA_2G14360; AltName: Full=Mixed-linked glucanase AFUA_2G14360; Flags: Precursor >B0XTU6.1 RecName: Full=Probable endo-1,3(4)-beta-glucanase AFUB_029980; AltName: Full=Mixed-linked glucanase | 45.46363755 | 14.39992008 | -1.654865351 | | 0.002549069 |
| Bcin01g10150 | Q5AK66.1 RecName: Full=Phosphatidylserine decarboxylase proenzyme 2; Contains: RecName: Full=Phosphatidylserine decarboxylase 2 beta chain; Contains: RecName: Full=Phosphatidylserine decarboxylase 2 alpha chain | 180.3907632 | 57.07214995 | -1.660020154 | | 1.8E-10 |
| Bcin12g06760 | - | 632.2175761 | 198.0490079 | -1.67473258 | | 1.76E-38 |
| Bcin06g00520 | - | 483.3634597 | 151.2519822 | -1.676583604 | | 5.5E-32 |
| Bcin07g02390 | - | 64.5250453 | 20.19234412 | -1.680183181 | | 0.000318858 |
| Bcin06g04350 | - | 106.3460276 | 32.75905944 | -1.696390286 | | 0.00000148 |
| Bcin02g05470 | - | 56.10372388 | 17.14345317 | -1.710002122 | | 0.000580661 |
| Bcin15g04330 | Q9US44.1 RecName: Full=Uncharacterized transporter C1002.16c | 24.65152852 | 7.445383615 | -1.728711417 | | 0.030532598 |
| Bcin05g01780 | - | 89.56202258 | 26.94571968 | -1.736053213 | | 0.0000155 |
| Bcin11g06510 | - | 68.8969915 | 20.41076572 | -1.756668058 | | 0.000222199 |
| Bcin01g03520 | - | 92.43338227 | 27.32224633 | -1.762310879 | | 0.00000375 |
| Bcin01g05880 | Q9Y7K4.1 RecName: Full=Uncharacterized protein C2A9.02 | 163.3724495 | 47.86183983 | -1.772253 | | 1.07E-10 |
| Bcin10g05370 | O94562.1 RecName: Full=Uncharacterized aminotransferase C1771.03c | 3114.968711 | 908.2239817 | -1.778252724 | | 9.35E-137 |
| Bcin15g04780 | Q93VK5.1 RecName: Full=Protein LUTEIN DEFICIENT 5, chloroplastic; AltName: Full=Cytochrome P450 97A3; Flags: Precursor | 272.4438172 | 79.19051204 | -1.782955426 | | 3.51E-16 |
| Bcin04g05960 | - | 617.7175127 | 178.4596095 | -1.790735245 | | 8.41E-44 |
| Bcin12g06180 | B6HCY4.1 RecName: Full=Cyanide hydratase | 297.1864607 | 85.44430877 | -1.798163692 | | 4.09E-16 |
| Bcin03g01810 | - | 29.36241659 | 8.441686527 | -1.801974037 | | 0.006019206 |
| Bcin06g02830 | P27121.1 RecName: Full=Ornithine decarboxylase; Short=ODC | 75.60777069 | 21.3478509 | -1.818852918 | | 0.00040635 |
| Bcin04g05020 | D4B388.1 RecName: Full=Fasciclin-like arabinogalactan protein ARB_02922; Flags: Precursor | 65.01322626 | 18.16873483 | -1.8415189 | | 0.0000209 |
| Bcin13g01380 | P21836.1 RecName: Full=Acetylcholinesterase; Short=AChE; Flags: Precursor | 234.9214021 | 65.02467972 | -1.853967346 | | 8.22E-15 |
| Bcin01g10140 | - | 28.34599117 | 7.694808677 | -1.877432131 | | 0.007933861 |
| Bcin04g00460 | - | 33.43191802 | 9.021828977 | -1.88668098 | | 0.005513894 |
| Bcin13g02750 | - | 363.655363 | 98.20043113 | -1.888957479 | | 3.38E-29 |
| Bcin02g09240 | - | 391.2949175 | 102.6482757 | -1.930555357 | | 7.77E-31 |
| novel.220 | PF11807:Mycotoxin biosynthesis protein UstYa | 21.3480353 | 5.5448265 | -1.949964934 | | 0.022156389 |
| Bcin15g03140 | - | 56.31363735 | 14.47311357 | -1.957134667 | | 0.0000976 |
| Bcin15g04790 | P25358.1 RecName: Full=Elongation of fatty acids protein 2; AltName: Full=3-keto acyl-CoA synthase ELO2; AltName: Full=Fenpropimorph resistance protein 1; AltName: Full=Glucan synthesis protein 1; AltName: Full=Very-long-chain 3-oxoacyl-CoA synthase | 394.0322639 | 101.1079462 | -1.962644631 | | 7.95E-34 |
| Bcin01g05190 | - | 2469.706359 | 629.3960404 | -1.972554255 | | 3.35E-184 |
| Bcin04g06910 | - | 19.14789581 | 4.893515266 | -1.975355312 | | 0.029836992 |
| novel.1391 | - | 23.28755668 | 5.805438123 | -2.003608147 | | 0.023293634 |
| Bcin10g01710 | - | 19.59243366 | 4.902677118 | -2.004958141 | | 0.037073621 |
| Bcin08g05370 | - | 81.27939466 | 20.05000655 | -2.019642219 | | 0.000000392 |
| Bcin13g01360 | O74923.1 RecName: Full=Uncharacterized transporter C757.13 | 336.4204074 | 80.4040722 | -2.064794573 | | 5.01E-30 |
| Bcin11g00940 | - | 123.8828384 | 28.99757902 | -2.093885662 | | 5.06E-11 |
| Bcin09g02250 | - | 24.14737685 | 5.447235176 | -2.142480116 | | 0.010136426 |
| Bcin07g06780 | Q0CCX6.1 RecName: Full=Dihydrogeodin oxidase; Short=DHGO; AltName: Full=Geodin synthesis protein J; Flags: Precursor | 228.6086517 | 51.60509787 | -2.146108081 | | 1.64E-19 |
| Bcin15g04800 | P38256.1 RecName: Full=Uncharacterized protein YBR096W | 67.96529098 | 15.27901195 | -2.156887743 | | 0.00000435 |
| Bcin09g03770 | - | 17.29548063 | 3.859603254 | -2.160317925 | | 0.017904758 |
| Bcin03g00370 | - | 22.12679252 | 4.871142143 | -2.185423346 | | 0.008164949 |
| Bcin03g00280 | A1CYC2.2 RecName: Full=Probable pectin lyase A; Short=PLA; Flags: Precursor | 4690.772859 | 1007.786396 | -2.21829216 | | 0 |
| Bcin06g07050 | Q7Z9M7.3 RecName: Full=Endoglucanase-7; AltName: Full=Cellulase-61B; Short=Cel61B; AltName: Full=Endo-1,4-beta-glucanase VII; Short=EGVII; AltName: Full=Endoglucanase VII; AltName: Full=Endoglucanase-61B; Flags: Precursor | 118.5865156 | 25.1608804 | -2.236023471 | | 2.18E-12 |
| novel.297 | - | 13.90576171 | 2.910071296 | -2.256693358 | | 0.047638878 |
| Bcin01g08610 | Q6F6Y2.1 RecName: Full=FAD-dependent urate hydroxylase; AltName: Full=Flavoprotein urate hydroxylase | 3830.305359 | 775.2747455 | -2.304873274 | | 0 |
| Bcin01g07200 | - | 44.02994652 | 8.310535519 | -2.398125993 | | 0.0000343 |
| Bcin11g04530 | P25340.2 RecName: Full=Delta(24(24(1)))-sterol reductase; AltName: Full=C-24(28) sterol reductase; AltName: Full=Sterol Delta(24(28))-reductase | 23.96610077 | 4.520076341 | -2.406675212 | | 0.002320745 |
| Bcin06g00510 | - | 261.9339977 | 46.97793404 | -2.481461719 | | 1.76E-29 |
| novel.507 | - | 21.9588435 | 3.87739545 | -2.502155753 | | 0.002585584 |
| Bcin01g07360 | - | 10.78424719 | 1.614054462 | -2.740508182 | | 0.039426539 |
| Bcin02g08490 | - | 12.08242417 | 1.625241023 | -2.900742392 | | 0.022072992 |
| Bcin02g06830 | - | 10.21946339 | 1.311784321 | -2.972355565 | | 0.03433078 |
| Bcin02g04050 | - | 21.63516973 | 2.57679769 | -3.067472052 | | 0.000684426 |
| novel.863 | - | 14.00967903 | 1.62982195 | -3.110714397 | | 0.039111203 |
| Bcin04g06380 | - | 112.6727211 | 12.54965182 | -3.163942742 | | 1.41E-12 |
| Bcin09g06060 | - | 15.11038911 | 1.612029753 | -3.228339975 | | 0.007196601 |
| Bcin02g02040 | O14405.1 RecName: Full=Endoglucanase-4; AltName: Full=Cellulase IV; AltName: Full=Cellulase-61A; Short=Cel61A; AltName: Full=Endo-1,4-beta-glucanase IV; Short=EGIV; AltName: Full=Endoglucanase IV; AltName: Full=Endoglucanase-61A; Flags: Precursor | 849.6482319 | 88.28051794 | -3.269928572 | | 2.13E-86 |
| novel.957 | - | 15.78699217 | 1.600843192 | -3.296161968 | | 0.00939707 |
| novel.952 | - | 10.50615757 | 0.993746693 | -3.424007335 | | 0.022111141 |
| novel.1562 | - | 10.92682276 | 0.973929789 | -3.49045688 | | 0.021747318 |
| Bcin01g04420 | W7N2C1.1 RecName: Full=Non-canonical non-ribosomal peptide synthetase FUB8; AltName: Full=Fusaric acid biosynthesis protein 8 | 41.03079685 | 1.951908995 | -4.40101183 | | 0.000000132 |
| Bcin01g01690 | - | 9.164117342 | 0.331248898 | -4.721447507 | | 0.011347402 |
| novel.1102 | - | 10.12119131 | 0.324643263 | -4.865865748 | | 0.005355071 |
| Bcin01g07080 | - | 26.69532514 | 0.662497796 | -5.363628535 | | 0.0000409 |
| novel.350 | - | 643.6570016 | 9.646717672 | -6.059706534 | | 0.00027841 |
