# Supplementary material for: The GATA transcription factor BcWCL2 regulates citric acid secretion to maintain redox homeostasis and full virulence in Botrytis cinerea
Source: mBio. 2024 May 30;15(7):e00133-24. doi: 10.1128/mbio.00133-24 (PMC11253612; doi:10.1128/mbio.00133-24)
Supplement: Table S5 — Genes downregulated in Δbcwcl2 and upregulated in Δbcwcl2+CA. [file mbio.00133-24-s0006.docx]

**Table S5: 129 genes were down-regulated in Δ*bcwcl2* and up-regulated in Δ*bcwcl2*+CA.**

| **Feature ID (fungi.ensembl.org)** | **Putative Function** | **log2FoldChange:Δ*bcwcl2* vs WT** | **log2FoldChange:Δ*bcwcl2*+CA vs Δ*bcwcl2*** |
| --- | --- | --- | --- |
| Bcin11g02900 | P35049.1 RecName: Full=Trypsin; Flags: Precursor | -5.340616713 | 4.79960851 |
| Bcin13g02960 | P19222.1 RecName: Full=Carboxypeptidase A2; Flags: Precursor | -4.60327289 | 5.981239957 |
| Bcin06g00130 | Q9T095.1 RecName: Full=Oligopeptide transporter 6; Short=AtOPT6 | -4.25092852 | 3.539962922 |
| Bcin07g01720 | D4D5P5.1 RecName: Full=Probable dipeptidyl-peptidase 5; AltName: Full=Dipeptidyl-peptidase V; Short=DPP V; Short=DppV; Flags: Precursor | -4.109792071 | 5.190232284 |
| Bcin12g06300 | A6SBW7.1 RecName: Full=Neutral protease 2 homolog SNOG_10522; AltName: Full=Deuterolysin SNOG_10522; Flags: Precursor | -4.070672819 | 4.266672236 |
| Bcin05g01510 | - | -3.854428319 | 2.808098605 |
| Bcin06g04920 | - | -3.76708352 | 4.21604423 |
| Bcin10g03910 | P31540.2 RecName: Full=Heat shock protein hsp98; AltName: Full=Protein aggregation-remodeling factor hsp98 | -3.678686439 | 1.558743322 |
| Bcin02g04800 | B6V865.1 RecName: Full=Metallocarboxypeptidase A; Short=MCPA; AltName: Full=Carboxypeptidase M14A; Flags: Precursor | -3.559178218 | 4.101496539 |
| Bcin02g07500 | - | -3.547442774 | 1.047260223 |
| Bcin06g06720 | - | -3.462737117 | 1.904943862 |
| Bcin02g07440 | Q7Z9I0.2 RecName: Full=Uncharacterized MFS-type transporter SPBC409.08 | -3.419256623 | 3.011219994 |
| Bcin12g02780 | - | -3.373021177 | 1.491102527 |
| Bcin09g01190 | Q70J59.1 RecName: Full=Tripeptidyl-peptidase sed2; AltName: Full=Sedolisin-B; Flags: Precursor | -3.318166908 | 4.547241816 |
| Bcin16g02010 | Q4I624.1 RecName: Full=Hsp70 nucleotide exchange factor FES1 | -3.304010567 | 1.348038097 |
| Bcin09g01070 | Q10085.1 RecName: Full=Uncharacterized transporter C11D3.06 | -3.238307279 | 3.422885174 |
| Bcin07g01270 | P36842.2 RecName: Full=Nitrate reductase [NADPH]; Short=NR | -3.204293587 | 1.981018864 |
| Bcin07g05710 | Q54DY9.1 RecName: Full=Probable mitochondrial chaperone BCS1-B; AltName: Full=BCS1-like protein 2 | -3.204082506 | 3.104364239 |
| Bcin11g03580 | Q9C0V1.1 RecName: Full=Ammonium transporter 1 | -3.173737715 | 3.401891741 |
| Bcin06g00120 | Q9FG72.1 RecName: Full=Oligopeptide transporter 1; Short=AtOPT1 | -3.008699831 | 1.728253551 |
| Bcin14g04470 | Q9P768.1 RecName: Full=Uncharacterized amino-acid permease P7G5.06 | -2.805879894 | 3.026306154 |
| Bcin13g04470 | Q59VM4.1 RecName: Full=Transcriptional regulator RPN4 | -2.753518541 | 3.15825112 |
| Bcin08g02540 | Q1DKE7.1 RecName: Full=DNA ligase 4; AltName: Full=DNA ligase IV; AltName: Full=Polydeoxyribonucleotide synthase [ATP] 4 | -2.71609604 | 1.975187249 |
| Bcin02g07090 | P32382.1 RecName: Full=NADH oxidase | -2.689023874 | 1.558363506 |
| Bcin01g03760 | - | -2.642158463 | 2.33581029 |
| Bcin03g05820 | A2QV36.1 RecName: Full=Probable pectate lyase A; Flags: Precursor | -2.641545163 | 1.860419897 |
| Bcin07g02790 | A0A0D2YG01.1 RecName: Full=Non-canonical non-ribosomal peptide synthetase FUB8; AltName: Full=Fusaric acid biosynthesis protein 8 | -2.635785584 | 1.618547596 |
| Bcin13g04780 | - | -2.480053841 | 3.130685933 |
| Bcin14g04530 | Q9P6N2.1 RecName: Full=Pdp3-interacting factor 1 | -2.414691245 | 1.188056188 |
| Bcin15g01960 | - | -2.39533476 | 1.589616256 |
| Bcin14g01400 | Q6ZFZ4.1 RecName: Full=Calpain-type cysteine protease ADL1; AltName: Full=Phytocalpain ADL1; AltName: Full=Protein ADAXIALIZED LEAF1; AltName: Full=Protein DEFECTIVE KERNEL 1; Short=OsDEK1; AltName: Full=Protein SHOOTLESS 3; Flags: Precursor | -2.372067361 | 1.985445459 |
| Bcin08g03080 | Q9UUD1.1 RecName: Full=Sterol regulatory element-binding protein 1; Contains: RecName: Full=Processed sterol regulatory element-binding protein 1 | -2.329047036 | 1.140548117 |
| Bcin01g05790 | P22944.2 RecName: Full=Nitrite reductase [NAD(P)H] | -2.328676741 | 1.512422838 |
| Bcin16g02770 | A1DA48.1 RecName: Full=Neutral protease 2 homolog NFIA_031120; AltName: Full=Deuterolysin NFIA_031120; Flags: Precursor | -2.3275568 | 2.710509315 |
| Bcin13g04610 | Q9M8Z7.1 RecName: Full=Sterol 3-beta-glucosyltransferase UGT80A2; AltName: Full=UDP-glucose:sterol glucosyltransferase 80A2 | -2.290249056 | 1.536389862 |
| Bcin12g00640 | - | -2.261268191 | 2.349273417 |
| Bcin03g07360 | Q8NJK6.1 RecName: Full=Probable pectin lyase F; Short=PLF; Flags: Precursor >A2R6A1.1 RecName: Full=Probable pectin lyase F; Short=PLF; Flags: Precursor | -2.231628538 | 1.925717547 |
| Bcin16g04250 | - | -2.230065591 | 1.009582432 |
| Bcin14g00870 | O59700.1 RecName: Full=Uncharacterized transporter C36.03c | -2.211345186 | 1.300841642 |
| Bcin13g05050 | - | -2.202416681 | 2.200051834 |
| Bcin04g05700 | P0CH36.1 RecName: Full=NADP-dependent alcohol dehydrogenase C 1; Short=Ms-ADHC 1 >P0CH37.1 RecName: Full=NADP-dependent alcohol dehydrogenase C 2; Short=Ms-ADHC 2 | -2.163110026 | 1.404010266 |
| Bcin12g03340 | Q55GW8.2 RecName: Full=AN1-type zinc finger and UBX domain-containing protein DDB_G0268260 | -2.152598924 | 1.00459446 |
| novel.1025 | - | -2.149465858 | 2.139171746 |
| Bcin11g01680 | - | -2.149353711 | 1.390479597 |
| Bcin09g07120 | - | -2.142972304 | 1.510220927 |
| Bcin03g01210 | - | -2.137760911 | 2.685754951 |
| Bcin09g03120 | - | -2.127475653 | 1.948632485 |
| Bcin05g01970 | - | -2.101709768 | 2.638366208 |
| Bcin13g05270 | Q8LNW4.1 RecName: Full=Flotillin-like protein 2; AltName: Full=Nodulin-like protein 2 | -2.100042373 | 2.221495528 |
| Bcin03g09120 | - | -2.09049927 | 3.037671141 |
| novel.481 | - | -2.085135485 | 1.823775305 |
| Bcin01g03240 | - | -2.07338277 | 2.158798878 |
| Bcin10g02530 | L8FSM5.1 RecName: Full=Subtilisin-like protease 2; AltName: Full=Destructin-1; AltName: Full=Serine protease 2; Short=PdSP2; Flags: Precursor | -2.019414524 | 3.454754659 |
| Bcin12g00760 | O94300.1 RecName: Full=Putative xanthine/uracil permease C887.17 | -1.997169664 | 2.394712133 |
| novel.927 | - | -1.997118569 | 2.266169417 |
| Bcin10g03880 | P40467.1 RecName: Full=Activator of stress genes 1 | -1.90865925 | 1.050193671 |
| Bcin10g05670 | - | -1.902335892 | 2.522179921 |
| Bcin13g05470 | - | -1.881193329 | 1.317414082 |
| Bcin04g02890 | - | -1.856300607 | 1.357343868 |
| Bcin11g03050 | Q12691.1 RecName: Full=Sodium transport ATPase 5 | -1.839276919 | 2.351621789 |
| Bcin06g04070 | Q94CA0.1 RecName: Full=Protein LAZ1 homolog 1; AltName: Full=Lazarus1 homolog 1; Flags: Precursor | -1.837686279 | 1.587328605 |
| Bcin07g06730 | - | -1.792982464 | 1.888654003 |
| novel.782 | - | -1.790769708 | 1.851880217 |
| Bcin15g01370 | - | -1.788287772 | 1.557827122 |
| Bcin02g02420 | P80235.2 RecName: Full=Putative mitochondrial carnitine O-acetyltransferase | -1.782843165 | 1.853919302 |
| Bcin11g02370 | - | -1.771270991 | 2.256109188 |
| Bcin10g05190 | - | -1.744785815 | 1.417884065 |
| Bcin14g03350 | - | -1.739492639 | 1.722345481 |
| Bcin12g00910 | - | -1.738141588 | 1.683278893 |
| Bcin05g04840 | O94469.1 RecName: Full=Probable urea active transporter 1 | -1.724277324 | 2.320997762 |
| Bcin16g04120 | O74023.1 RecName: Full=Methylated-DNA--protein-cysteine methyltransferase; AltName: Full=6-O-methylguanine-DNA methyltransferase; Short=MGMT; AltName: Full=O-6-methylguanine-DNA-alkyltransferase; AltName: Full=Pk-MGMT | -1.705728997 | 1.780400543 |
| Bcin12g05550 | P00504.3 RecName: Full=Aspartate aminotransferase, cytoplasmic; Short=cAspAT; AltName: Full=Cysteine aminotransferase, cytoplasmic; AltName: Full=Cysteine transaminase, cytoplasmic; Short=cCAT; AltName: Full=Glutamate oxaloacetate transaminase 1; Alt | -1.682216014 | 1.652770137 |
| Bcin15g05670 | Q01896.1 RecName: Full=Sodium transport ATPase 2 | -1.676515812 | 3.862362915 |
| Bcin14g01410 | Q9URZ3.1 RecName: Full=Probable proline-specific permease put4 | -1.664348645 | 2.456286512 |
| Bcin02g05300 | - | -1.64174968 | 1.09010764 |
| Bcin03g00460 | Q87GU5.1 RecName: Full=Autoinducer 2 sensor kinase/phosphatase LuxQ | -1.634421951 | 1.411324871 |
| Bcin03g01750 | O14123.1 RecName: Full=Probable Na(+)/H(+) antiporter C3A11.09 | -1.619660932 | 1.065536688 |
| Bcin15g03000 | - | -1.607324412 | 1.109421952 |
| Bcin10g04970 | - | -1.602735423 | 3.007267274 |
| Bcin10g04810 | Q2YIJ8.1 RecName: Full=Glucose/galactose transporter >P0C105.1 RecName: Full=Glucose/galactose transporter | -1.599104512 | 2.461668065 |
| Bcin11g06180 | P53099.1 RecName: Full=Vitamin B6 transporter TPN1; AltName: Full=Transport of pyridoxine protein 1 | -1.590975505 | 1.246284168 |
| Bcin02g05670 | - | -1.551447406 | 1.829284567 |
| Bcin03g08650 | B8NM76.2 RecName: Full=ustiloxin B cluster transcription factor ustR; AltName: Full=Ustiloxin B biosynthesis protein R | -1.5481207 | 1.756663954 |
| Bcin11g05020 | - | -1.54498169 | 2.199181611 |
| Bcin03g06610 | - | -1.537682971 | 1.530923778 |
| Bcin15g02000 | - | -1.523351519 | 1.206284522 |
| Bcin03g00760 | - | -1.518072352 | 2.213614976 |
| Bcin01g06040 | P53390.1 RecName: Full=Ammonium transporter MEP3 | -1.508253567 | 2.470807265 |
| Bcin11g05960 | - | -1.496187588 | 1.051129681 |
| Bcin15g05350 | - | -1.448701322 | 1.277103632 |
| Bcin05g01940 | - | -1.437205272 | 1.271691466 |
| Bcin10g05950 | Q9P413.1 RecName: Full=pH-response transcription factor pacC/RIM101 | -1.431774176 | 2.582729636 |
| Bcin10g01550 | P46030.1 RecName: Full=Peptide transporter PTR2 | -1.408785463 | 1.55905459 |
| Bcin09g03330 | P87049.3 RecName: Full=G1/S-specific cyclin pas1 | -1.407846006 | 1.116050591 |
| Bcin14g04570 | P22189.1 RecName: Full=Calcium-transporting ATPase 3 | -1.396886068 | 3.540232196 |
| Bcin10g01980 | O00093.2 RecName: Full=3-phytase B; AltName: Full=3 phytase B; AltName: Full=Myo-inositol hexakisphosphate phosphohydrolase B; AltName: Full=Myo-inositol-hexaphosphate 3-phosphohydrolase B; Flags: Precursor | -1.385051546 | 1.497017523 |
| Bcin10g03530 | Q59QC7.1 RecName: Full=Sterol uptake control protein 2 | -1.36235285 | 1.030850456 |
| Bcin07g01750 | Q9LQV2.1 RecName: Full=RNA-dependent RNA polymerase 1; Short=AtRDRP1; AltName: Full=RNA-directed RNA polymerase 1 | -1.354605199 | 1.244628419 |
| Bcin09g06830 | Q0D0A1.2 RecName: Full=PAB-dependent poly(A)-specific ribonuclease subunit pan3; AltName: Full=PAB1P-dependent poly(A)-nuclease; AltName: Full=PAN deadenylation complex subunit 3 | -1.346355441 | 1.024683877 |
| Bcin09g02790 | P16928.2 RecName: Full=Acetyl-coenzyme A synthetase; AltName: Full=Acetate--CoA ligase; AltName: Full=Acyl-activating enzyme | -1.343632799 | 1.135620223 |
| Bcin16g03420 | P33303.2 RecName: Full=Succinate/fumarate mitochondrial transporter; AltName: Full=Regulator of acetyl-CoA synthase activity | -1.326971394 | 1.495979421 |
| Bcin16g04050 | O93934.1 RecName: Full=NADP-specific glutamate dehydrogenase; Short=NADP-GDH; AltName: Full=NADP-dependent glutamate dehydrogenase | -1.321607574 | 1.935800615 |
| Bcin16g03650 | O13817.2 RecName: Full=Protein transport protein sec73 | -1.316915836 | 1.203520051 |
| Bcin13g05010 | - | -1.309012577 | 1.213731937 |
| Bcin14g03260 | - | -1.308903209 | 1.260691 |
| Bcin13g04620 | - | -1.292573846 | 1.400461418 |
| Bcin14g04550 | O14111.2 RecName: Full=Phosphatidylserine decarboxylase proenzyme 3; Contains: RecName: Full=Phosphatidylserine decarboxylase 3 beta chain; Contains: RecName: Full=Phosphatidylserine decarboxylase 3 alpha chain | -1.27225087 | 1.041626483 |
| Bcin05g02400 | Q9P3B2.1 RecName: Full=Respiratory supercomplex factor 2 homolog C1565.01 | -1.254987045 | 1.120194795 |
| Bcin06g05780 | - | -1.246388667 | 1.183136056 |
| Bcin02g07860 | Q10146.2 RecName: Full=Exosome complex exonuclease rrp6; AltName: Full=Ribosomal RNA-processing protein 6 | -1.226315316 | 1.236132893 |
| Bcin07g04040 | O14031.1 RecName: Full=Glutathione transporter 1 | -1.169949945 | 1.070456049 |
| Bcin07g04370 | B8NM69.1 RecName: Full=Peptidase S41 family protein ustP; AltName: Full=Ustiloxin B biosynthesis protein P | -1.169231258 | 1.730733082 |
| Bcin13g04830 | A1CEK6.1 RecName: Full=Class E vacuolar protein-sorting machinery protein hse1 | -1.160238226 | 1.114745838 |
| Bcin11g06260 | A7E727.1 RecName: Full=Mitochondrial outer membrane protein iml2 | -1.14791415 | 1.063586481 |
| Bcin05g01530 | - | -1.14474335 | 1.906503846 |
| Bcin10g05610 | - | -1.127508101 | 1.41114793 |
| Bcin10g00520 | - | -1.120319229 | 1.452523118 |
| Bcin09g02070 | P36091.1 RecName: Full=Mannan endo-1,6-alpha-mannosidase DCW1; AltName: Full=Defective cell wall 1; AltName: Full=Endo-alpha-1->6-D-mannanase DCW1; Flags: Precursor | -1.117457304 | 1.867317676 |
| Bcin05g05830 | C5FHK0.1 RecName: Full=Tripeptidyl-peptidase SED1; AltName: Full=Sedolisin-A; Flags: Precursor | -1.111997414 | 1.766842387 |
| Bcin11g01950 | P53326.1 RecName: Full=Uncharacterized protein YGR266W | -1.109650928 | 1.379114288 |
| Bcin05g00390 | W7MLD3.1 RecName: Full=Efflux pump FUS6; AltName: Full=Fusarin biosynthesis protein 6 | -1.08543383 | 1.148055548 |
| Bcin13g00050 | - | -1.056969326 | 1.113101242 |
| Bcin14g04610 | A0QZE3.1 RecName: Full=Putative hydrolase MSMEG_3995/MSMEI_3903 | -1.056175195 | 1.365338289 |
| Bcin09g00370 | F4HX15.1 RecName: Full=Phospholipase A I; Short=AtPLA1 | -1.050955061 | 1.5629896 |
| Bcin15g04180 | P36616.2 RecName: Full=Protein kinase dsk1; AltName: Full=Dis1-suppressing protein kinase | -1.041580499 | 1.249497124 |
| Bcin02g01010 | - | -1.041385679 | 1.61491269 |
| Bcin13g03260 | Q01389.1 RecName: Full=Serine/threonine-protein kinase BCK1/SLK1/SSP31 | -1.0292294 | 2.298389781 |
| Bcin12g06750 | P46030.1 RecName: Full=Peptide transporter PTR2 | -1.023507667 | 2.653150452 |
| Bcin09g03190 | O94701.1 RecName: Full=Ingression protein fic1; AltName: Full=Cdc15-interacting C2 domain-containing protein 1 | -1.010095248 | 1.011151527 |
