# Supplementary material for: The GATA transcription factor BcWCL2 regulates citric acid secretion to maintain redox homeostasis and full virulence in Botrytis cinerea
Source: mBio. 2024 May 30;15(7):e00133-24. doi: 10.1128/mbio.00133-24 (PMC11253612; doi:10.1128/mbio.00133-24)
Supplement: Table S6 — Genes of Cluster1 upregulated in Δbcwcl2 and downregulated in Δbcwcl2+CA. [file mbio.00133-24-s0007.docx]

**Table S6: 116 genes of Cluster1 were up-regulated in Δ*bcwcl2* and down-regulated in Δ*bcwcl2*+CA.**

| **Feature ID (fungi.ensembl.org)** | **Putative Function** | **log2FoldChange:Δ*bcwcl2* vs WT** | **log2FoldChange:Δ*bcwcl2*+CA vs Δ*bcwcl2*** |
| --- | --- | --- | --- |
| Bcin07g05940 | - | 1.002336659 | -1.045768894 |
| Bcin08g02100 | Q10DK7.1 RecName: Full=1-aminocyclopropane-1-carboxylate synthase 1; Short=ACC synthase 1; AltName: Full=S-adenosyl-L-methionine methylthioadenosine-lyase 1 >A2XLL2.2 RecName: Full=1-aminocyclopropane-1-carboxylate synthase 1; Short=ACC synthase 1; A | 1.014197761 | -1.229714849 |
| Bcin16g02990 | - | 1.01501515 | -1.028963911 |
| novel.312 | - | 1.030465144 | -1.202625464 |
| Bcin06g04350 | - | 1.042763788 | -1.696390286 |
| Bcin01g04540 | O74631.1 RecName: Full=Protein FDD123; AltName: Full=CvHSP30/1 | 1.045169363 | -1.099092402 |
| Bcin09g05570 | O04036.3 RecName: Full=Sugar transporter ERD6; AltName: Full=Early-responsive to dehydration protein 6; AltName: Full=Sugar transporter-like protein 1 | 1.049481178 | -1.258055894 |
| Bcin05g00100 | - | 1.052764838 | -1.088982961 |
| Bcin01g05880 | Q9Y7K4.1 RecName: Full=Uncharacterized protein C2A9.02 | 1.053624821 | -1.772253 |
| Bcin13g02320 | O94218.1 RecName: Full=Xyloglucan-specific endo-beta-1,4-glucanase A; AltName: Full=Xyloglucanase A; AltName: Full=Xyloglucanendohydrolase A; Flags: Precursor | 1.061839738 | -1.309915892 |
| Bcin07g05480 | - | 1.062976986 | -1.009124596 |
| Bcin07g02390 | - | 1.065747378 | -1.680183181 |
| Bcin12g01620 | - | 1.069359892 | -1.132107939 |
| Bcin12g04870 | Q9P3V5.1 RecName: Full=Uncharacterized transporter C1348.05 | 1.074274664 | -1.111633993 |
| Bcin03g01580 | O16171.1 RecName: Full=Esterase-5C; Short=Est-5C; AltName: Full=Carboxylic-ester hydrolase 5C; Short=Carboxylesterase-5C; Flags: Precursor | 1.077909127 | -1.06582202 |
| Bcin01g08610 | Q6F6Y2.1 RecName: Full=FAD-dependent urate hydroxylase; AltName: Full=Flavoprotein urate hydroxylase | 1.092954521 | -2.304873274 |
| Bcin12g02830 | Q2UNR0.1 RecName: Full=Probable beta-glucosidase D; AltName: Full=Beta-D-glucoside glucohydrolase D; AltName: Full=Cellobiase D; AltName: Full=Gentiobiase D; Flags: Precursor | 1.146167332 | -1.105828192 |
| Bcin08g04590 | - | 1.148391826 | -1.319170953 |
| Bcin12g02360 | - | 1.155022748 | -1.214402579 |
| novel.268 | - | 1.156843332 | -1.033281266 |
| Bcin05g01660 | P29717.4 RecName: Full=Glucan 1,3-beta-glucosidase; AltName: Full=Exo-1,3-beta-glucanase; Flags: Precursor | 1.183503611 | -1.120076006 |
| Bcin04g05960 | - | 1.223027344 | -1.790735245 |
| Bcin06g04940 | O74628.1 RecName: Full=Uncharacterized oxidoreductase C162.03 | 1.23036542 | -1.472626291 |
| Bcin03g00480 | Q2LMP0.1 RecName: Full=Endo-1,4-beta-xylanase 11A; Short=Xylanase 11A; AltName: Full=1,4-beta-D-xylan xylanohydrolase 11A; Flags: Precursor | 1.230587488 | -1.052201764 |
| Bcin06g04950 | Q99385.1 RecName: Full=Vacuolar calcium ion transporter; AltName: Full=High copy number undoes manganese protein 1; AltName: Full=Manganese resistance 1 protein; AltName: Full=Vacuolar Ca(2+)/H(+) exchanger | 1.239281977 | -1.14405016 |
| Bcin15g03080 | Q00298.1 RecName: Full=Cutinase; AltName: Full=Cutin hydrolase; Flags: Precursor | 1.245642133 | -1.212742806 |
| Bcin05g01780 | - | 1.264743768 | -1.736053213 |
| Bcin12g03000 | - | 1.276455413 | -1.107675081 |
| Bcin02g09240 | - | 1.299308824 | -1.930555357 |
| Bcin06g04140 | P49374.1 RecName: Full=High-affinity glucose transporter | 1.305485164 | -1.011099548 |
| Bcin09g05970 | - | 1.346361342 | -1.025641452 |
| Bcin03g05810 | - | 1.35066875 | -1.096074683 |
| Bcin07g04120 | - | 1.352896087 | -1.120490222 |
| Bcin05g06610 | P39932.2 RecName: Full=Sugar transporter STL1 | 1.35379063 | -1.272974244 |
| Bcin10g05370 | O94562.1 RecName: Full=Uncharacterized aminotransferase C1771.03c | 1.358200526 | -1.778252724 |
| Bcin07g03780 | A1D2R3.1 RecName: Full=Probable quinate permease; AltName: Full=Quinate transporter | 1.363884976 | -1.075338735 |
| novel.238 | - | 1.378403342 | -1.178052715 |
| Bcin15g05080 | P0C7S9.1 RecName: Full=1,3-beta-glucanosyltransferase gel1; AltName: Full=Glucan elongating glucanosyltransferase 1; Flags: Precursor | 1.401815942 | -1.180906828 |
| Bcin13g00200 | B0XQS8.1 RecName: Full=Probable quinate permease; AltName: Full=Quinate transporter | 1.406402833 | -1.029197941 |
| Bcin05g03580 | - | 1.410089119 | -1.014089661 |
| Bcin13g01360 | O74923.1 RecName: Full=Uncharacterized transporter C757.13 | 1.425294353 | -2.064794573 |
| novel.607 | - | 1.432939209 | -1.504060197 |
| Bcin10g00310 | D4B0V1.1 RecName: Full=Probable glucan endo-1,3-beta-glucosidase ARB_02077; AltName: Full=(1->3)-beta-glucan endohydrolase ARB_02077; Short=(1->3)-beta-glucanase ARB_02077; Flags: Precursor | 1.473921617 | -1.221185661 |
| Bcin03g08110 | O14434.1 RecName: Full=Scytalone dehydratase arp1; AltName: Full=Conidial pigment biosynthesis oxidase arp1 | 1.475640153 | -1.050335287 |
| Bcin03g01520 | P42328.1 RecName: Full=Alcohol dehydrogenase; AltName: Full=ADH-HT | 1.477217557 | -1.471066481 |
| Bcin14g03170 | P80402.2 RecName: Full=2,3-dihydroxybenzoate decarboxylase; Short=2,3-DHBA decarboxylase; Short=DHBD; AltName: Full=o-pyrocatechuate decarboxylase | 1.481106338 | -1.065662486 |
| Bcin11g00940 | - | 1.492515869 | -2.093885662 |
| Bcin10g06060 | - | 1.494916541 | -1.000641136 |
| Bcin07g06820 | - | 1.504052818 | -1.158640234 |
| Bcin03g00280 | A1CYC2.2 RecName: Full=Probable pectin lyase A; Short=PLA; Flags: Precursor | 1.516376365 | -2.21829216 |
| Bcin06g06410 | G2QJ27.1 RecName: Full=Acetylesterase; AltName: Full=Carbohydrate esterase family 16 protein; Flags: Precursor | 1.55366742 | -1.570691954 |
| novel.865 | - | 1.556015832 | -1.319742845 |
| Bcin03g03200 | - | 1.563785901 | -1.319551114 |
| Bcin14g04260 | - | 1.568489367 | -1.452581286 |
| Bcin13g01380 | P21836.1 RecName: Full=Acetylcholinesterase; Short=AChE; Flags: Precursor | 1.585593702 | -1.853967346 |
| Bcin06g04290 | - | 1.626005321 | -1.056954122 |
| novel.1302 | - | 1.628046669 | -1.279408937 |
| novel.1042 | - | 1.637262552 | -1.28533742 |
| Bcin06g04270 | - | 1.637636891 | -1.145720211 |
| Bcin08g06830 | B8NJF4.2 RecName: Full=Probable beta-glucosidase D; AltName: Full=Beta-D-glucoside glucohydrolase D; AltName: Full=Cellobiase D; AltName: Full=Gentiobiase D; Flags: Precursor | 1.667536623 | -1.579867184 |
| Bcin12g01100 | Q00001.1 RecName: Full=Rhamnogalacturonase A; Short=RGase A; Short=RHG A; AltName: Full=Rhamnogalacturonan hydrolase A; Flags: Precursor | 1.713849018 | -1.420883533 |
| Bcin03g00370 | - | 1.748288582 | -2.185423346 |
| Bcin01g11040 | - | 1.783963738 | -1.094169662 |
| Bcin06g00520 | - | 1.796471623 | -1.676583604 |
| Bcin01g11290 | Q05031.1 RecName: Full=Mannan endo-1,6-alpha-mannosidase DFG5; AltName: Full=Endo-alpha-1->6-D-mannanase DFG5; Flags: Precursor | 1.805853931 | -1.433774892 |
| Bcin05g05020 | Q9UQY0.2 RecName: Full=Demethylsterigmatocystin 6-O-methyltransferase; AltName: Full=Aflatoxin biosynthesis protein O; AltName: Full=Methyltransferase B; AltName: Full=O-methyltransferase I; Short=mt-I | 1.828105253 | -1.116669908 |
| Bcin12g01950 | A7MBI7.1 RecName: Full=Catechol O-methyltransferase | 1.830607501 | -1.128003954 |
| Bcin15g03140 | - | 1.845643932 | -1.957134667 |
| Bcin01g10140 | - | 1.849167037 | -1.877432131 |
| Bcin05g02270 | - | 1.885167172 | -1.360202249 |
| Bcin15g04330 | Q9US44.1 RecName: Full=Uncharacterized transporter C1002.16c | 1.894061123 | -1.728711417 |
| Bcin09g02260 | D4AK17.1 RecName: Full=PI-PLC X domain-containing protein 1; Flags: Precursor | 1.955852653 | -1.152509266 |
| novel.507 | - | 2.033621909 | -2.502155753 |
| Bcin08g03760 | P11838.2 RecName: Full=Endothiapepsin; AltName: Full=Aspartate protease; Flags: Precursor | 2.053586365 | -1.073960944 |
| Bcin04g00460 | - | 2.279707277 | -1.88668098 |
| Bcin06g03740 | - | 2.283104387 | -1.380721967 |
| Bcin07g02730 | P49426.1 RecName: Full=Glucan 1,3-beta-glucosidase; AltName: Full=1,3-beta-D-glucanohydrolase; AltName: Full=Exo-beta 1,3 glucanase; Flags: Precursor | 2.293679451 | -1.141392148 |
| Bcin10g04860 | - | 2.296453747 | -1.571936489 |
| Bcin06g03730 | - | 2.351431196 | -1.35878574 |
| Bcin08g03830 | Q9LBG2.1 RecName: Full=Levodione reductase; AltName: Full=(6R)-2,2,6-trimethyl-1,4-cyclohexanedione reductase | 2.382721976 | -1.027367661 |
| Bcin16g02560 | Q2U4L7.2 RecName: Full=Glutaminase A; Flags: Precursor | 2.428217192 | -1.077050285 |
| Bcin10g01150 | - | 2.484199831 | -1.196114477 |
| Bcin03g00900 | O13752.1 RecName: Full=Uncharacterized TLC domain-containing protein C17A2.02c | 2.535573548 | -1.218128409 |
| Bcin01g07330 | A2QBB6.1 RecName: Full=Probable endopolygalacturonase E; Short=PGE; AltName: Full=Pectinase 4; AltName: Full=Pectinase E; AltName: Full=Polygalacturonase E; AltName: Full=Polygalacturonase IV; Short=PG-IV; Flags: Precursor | 2.540804701 | -1.30848218 |
| Bcin15g04800 | P38256.1 RecName: Full=Uncharacterized protein YBR096W | 2.620453466 | -2.156887743 |
| Bcin01g05680 | Q0D076.1 RecName: Full=Probable mannosyl-oligosaccharide alpha-1,2-mannosidase 1B; AltName: Full=Class I alpha-mannosidase 1B; AltName: Full=Man(9)-alpha-mannosidase 1B; Flags: Precursor | 2.649692884 | -1.208981675 |
| Bcin14g05510 | Q5B9Z8.2 RecName: Full=Probable alpha-L-arabinofuranosidase axhA-1; AltName: Full=Arabinoxylan arabinofuranohydrolase axhA-1; Flags: Precursor | 2.732277527 | -1.626997083 |
| Bcin07g00890 | Q12713.1 RecName: Full=Endochitinase 33; AltName: Full=33 kDa endochitinase; AltName: Full=Chitinase 33; Flags: Precursor | 2.743001431 | -1.23006152 |
| novel.220 | PF11807:Mycotoxin biosynthesis protein UstYa | 2.766902423 | -1.949964934 |
| Bcin08g05370 | - | 2.769952627 | -2.019642219 |
| Bcin15g04750 | P24458.1 RecName: Full=Cytochrome P450 52A3-B; Short=CYP52A3-B; AltName: Full=Alkane-inducible P450-ALK1-B; AltName: Full=CYPLIIA3 | 2.814531296 | -1.523067737 |
| Bcin01g07200 | - | 2.869355562 | -2.398125993 |
| Bcin01g10150 | Q5AK66.1 RecName: Full=Phosphatidylserine decarboxylase proenzyme 2; Contains: RecName: Full=Phosphatidylserine decarboxylase 2 beta chain; Contains: RecName: Full=Phosphatidylserine decarboxylase 2 alpha chain | 2.880330088 | -1.660020154 |
| Bcin01g07210 | D4ATR3.1 RecName: Full=Uncharacterized secreted glycosidase ARB_07629; Flags: Precursor | 2.912918936 | -1.343175661 |
| Bcin12g06760 | - | 2.947950074 | -1.67473258 |
| Bcin15g04770 | - | 2.952724933 | -1.546664123 |
| Bcin15g01700 | E9R876.1 RecName: Full=MFS gliotoxin efflux transporter gliA; AltName: Full=Gliotoxin biosynthesis protein A | 2.963259419 | -1.594832913 |
| Bcin07g06780 | Q0CCX6.1 RecName: Full=Dihydrogeodin oxidase; Short=DHGO; AltName: Full=Geodin synthesis protein J; Flags: Precursor | 2.966804685 | -2.146108081 |
| Bcin01g03520 | - | 2.990972927 | -1.762310879 |
| Bcin03g05200 | D4AZ24.1 RecName: Full=Probable endo-1,3(4)-beta-glucanase ARB_01444; Short=Endo-1,3-beta-glucanase; Short=Endo-1,4-beta-glucanase; AltName: Full=Laminarinase; Flags: Precursor | 3.020382294 | -1.197027006 |
| Bcin15g04780 | Q93VK5.1 RecName: Full=Protein LUTEIN DEFICIENT 5, chloroplastic; AltName: Full=Cytochrome P450 97A3; Flags: Precursor | 3.031404449 | -1.782955426 |
| Bcin03g03480 | Q96VB6.1 RecName: Full=Endo-1,4-beta-xylanase F3; Short=Xylanase F3; AltName: Full=1,4-beta-D-xylan xylanohydrolase F3; Flags: Precursor | 3.109320048 | -1.604939038 |
| Bcin03g00080 | - | 3.144430502 | -1.183377403 |
| Bcin15g04760 | - | 3.153932861 | -1.628440181 |
| Bcin06g00510 | - | 3.328952268 | -2.481461719 |
| Bcin06g02830 | P27121.1 RecName: Full=Ornithine decarboxylase; Short=ODC | 3.452896677 | -1.818852918 |
| Bcin14g00080 | - | 3.463064336 | -1.137611828 |
| novel.863 | - | 3.466864222 | -3.110714397 |
| Bcin11g02630 | Q0C8A0.1 RecName: Full=Dioxygenase trt7; AltName: Full=Terretonin synthesis protein 7 | 3.603666547 | -1.02140365 |
| Bcin11g06510 | - | 3.686112459 | -1.756668058 |
| Bcin15g04790 | P25358.1 RecName: Full=Elongation of fatty acids protein 2; AltName: Full=3-keto acyl-CoA synthase ELO2; AltName: Full=Fenpropimorph resistance protein 1; AltName: Full=Glucan synthesis protein 1; AltName: Full=Very-long-chain 3-oxoacyl-CoA synthase | 3.953026788 | -1.962644631 |
| Bcin02g02040 | O14405.1 RecName: Full=Endoglucanase-4; AltName: Full=Cellulase IV; AltName: Full=Cellulase-61A; Short=Cel61A; AltName: Full=Endo-1,4-beta-glucanase IV; Short=EGIV; AltName: Full=Endoglucanase IV; AltName: Full=Endoglucanase-61A; Flags: Precursor | 4.05252932 | -3.269928572 |
| Bcin06g07050 | Q7Z9M7.3 RecName: Full=Endoglucanase-7; AltName: Full=Cellulase-61B; Short=Cel61B; AltName: Full=Endo-1,4-beta-glucanase VII; Short=EGVII; AltName: Full=Endoglucanase VII; AltName: Full=Endoglucanase-61B; Flags: Precursor | 4.164364472 | -2.236023471 |
| Bcin04g06380 | - | 5.026362706 | -3.163942742 |
| Bcin16g04060 | Q4X084.1 RecName: Full=Probable endo-1,3(4)-beta-glucanase AFUA_2G14360; AltName: Full=Mixed-linked glucanase AFUA_2G14360; Flags: Precursor >B0XTU6.1 RecName: Full=Probable endo-1,3(4)-beta-glucanase AFUB_029980; AltName: Full=Mixed-linked glucanase | 5.173124528 | -1.654865351 |
| novel.957 | - | 6.509466954 | -3.296161968 |
