# Supplementary material for: The GATA transcription factor BcWCL2 regulates citric acid secretion to maintain redox homeostasis and full virulence in Botrytis cinerea
Source: mBio. 2024 May 30;15(7):e00133-24. doi: 10.1128/mbio.00133-24 (PMC11253612; doi:10.1128/mbio.00133-24)
Supplement: Table S7 — FPKM values of differentially expressed genes in Fig. 6A, 8G, and S5A. [file mbio.00133-24-s0008.docx]

**Table S7: FPKM values of differentially expressed genes in Figure 6A, 8G, and S5A.**

| **Feature ID (fungi.ensembl.org)** | **Gene name** | **WT mean FPKM 48h in planta** | **Δ*bcwcl2* mean FPKM 48h in planta** | **Δ*bcwcl2* +CA mean FPKM 48h in planta** | **log2FoldChange:Δ*bcwcl2* vs WT** | **padj adjusted for multiple testing with the Benjamini-Hochberg procedure** | **log2FoldChange:Δ*bcwcl2*+CA vs Δ*bcwcl2*** | **padj adjusted for multiple testing with the Benjamini-Hochberg procedure** |
| --- | --- | --- | --- | --- | --- | --- | --- | --- |
| **Fig 6A: Expression profiles derived from the transcriptome data of the key genes associated with citric acid (CA) synthesis in *B. cinerea*.** | | | | | | | | |
| Bcin02g02750 | Bccit1 | 702.6713789 | 632.3853061 | 579.8160117 | -0.156999901 | 4.99E-08 | -0.123560191 | 4.22E-05 |
| Bcin09g00650 | Bccit3 | 400.9369714 | 238.2377882 | 324.7343417 | -0.755863483 | 2.29E-85 | 0.448413233 | 4.80E-31 |
| Bcin01g09950 | Bcpyc | 304.0540757 | 211.16598 | 486.2922185 | -0.530856062 | 1.20E-71 | 1.204973571 | 0 |
| **Fig 6A: Expression profiles derived from the transcriptome data of the key genes associated with citric acid (CA) transport in *B. cinerea*.** | | | | | | | | |
| Bcin03g05230 | MFS_1 | 2.904406897 | 1.733257202 | 8.46835287 | -0.750212559 | 0.008455226 | 2.291023195 | 3.65E-27 |
| Bcin10g04810 | MFS_1 | 2.55191914 | 0.842851629 | 4.655468119 | -1.599104512 | 0.000132873 | 2.461668065 | 3.27E-13 |
| Bcin14g00870 | MFS_1 | 109.4318035 | 23.69749833 | 58.34481066 | -2.211345186 | 5.04E-250 | 1.300841642 | 4.39E-64 |
| Bcin02g07440 | MFS_1 | 106.9458338 | 10.02196434 | 80.77907858 | -3.419256623 | 0 | 3.011219994 | 1.88E-281 |
| Bcin05g00390 | MFS_1 | 5.685389534 | 2.689256601 | 5.950188555 | -1.08543383 | 4.98E-07 | 1.148055548 | 4.53E-08 |
| Bcin16g03420 | Mito_carr | 378.1371559 | 151.2574039 | 426.1104603 | -1.326971394 | 1.42E-230 | 1.495979421 | 1.31E-253 |
| Bcin02g08460 | CTP1 | 30.97543702 | 38.86000836 | 33.85163936 | 0.321952986 | 0.000892658 | -0.19700932 | 0.041986828 |
| Bcin03g05760 | CTP1 | 1.178087567 | 2.475801914 | 3.773381329 | 1.068265574 | 0.059164361 | 0.605960818 | 0.167664031 |
| **Fig 8G: Relative expression of ROS generation- and scavenging-associated genes in the WT, Δ*bcwcl2* and Δ*bcwcl2*+CA strains.** | | | | | | | | |
| Bcin06g03980 | Bchem15 | 5.768297199 | 9.854533765 | 7.470794669 | 0.767806036 | 6.63E-07 | -0.396889312 | 1.20E-02 |
| Bcin03g06840 | BcnoxR | 1.118818042 | 4.168616805 | 2.140345093 | 1.891358323 | 3.47E-08 | -0.958379652 | 0.001689304 |
| Bcin12g04240 | Bcdao1 | 17.73171592 | 30.36029256 | 22.38843179 | 0.77116191 | 2.05E-10 | -0.438358565 | 0.000190995 |
| Bcin01g00260 | Bcaao1 | 17.20317646 | 22.00543539 | 13.63610576 | 0.350358575 | 0.001312286 | -0.689250119 | 7.69E-10 |
| Bcin09g05400 | Bcdao8 | 9.501647812 | 12.46375908 | 9.59594335 | 0.387220286 | 0.019059573 | -0.376820045 | 0.025971086 |
| Bcin07g01080 | Bcdao11 | 7.613485593 | 10.46885139 | 7.364821989 | 0.454832738 | 0.010456948 | -0.506347049 | 0.005814984 |
| **Fig 8G: Relative expression of ROS generation- and scavenging-associated genes in the WT, Δ*bcwcl2* and Δ*bcwcl2*+CA strains.** | | | | | | | | |
| Bcin09g03930 | Bcprx8 | 621.9663057 | 274.4053632 | 345.2147405 | -1.185634082 | 4.91E-191 | 0.332880875 | 1.06E-14 |
| Bcin02g03060 | BcppoA80 | 60.70177027 | 56.02036984 | 76.38310337 | -0.120837623 | 0.011009148 | 0.448832908 | 2.43E-21 |
| Bcin12g00520 | Bcprx2 | 1.456341027 | 1.161108764 | 1.588838525 | -0.328797306 | 0.568688461 | 0.450772662 | 0.418896712 |
| Bcin07g05810 | Bcccp2 | 48.89992401 | 36.06707914 | 53.33535974 | -0.443473809 | 1.73E-05 | 0.565435106 | 2.21E-08 |
| Bcin09g04400 | Bccat7 | 0.402692099 | 0.16832753 | 0.449907417 | -1.259333421 | 0.292016951 | 1.416000024 | 0.221986463 |
| **Fig S5A: Expression profiles derived from the transcriptome data of acid hydrolases in *B. cinerea*.** | | | | | | | | |
| Bcin15g04670 | Serine peptidase S8_S53 | 310.4591466 | 216.5697244 | 284.3278667 | -0.524725147 | 3.99E-50 | 0.394516676 | 7.08E-26 |
| Bcin11g02900 | Serine peptidase (trypsin-like) | 608.7406169 | 15.09028132 | 419.3735506 | -5.340616713 | 0 | 4.79960851 | 0 |
| Bcin09g00190 | mannan endo-1,6-alpha-mannosidase precursor | 18.14926145 | 13.75697099 | 18.06049284 | -0.404926804 | 9.28E-05 | 0.394155839 | 0.00012035 |
| Bcin12g06300 | Zn metallopeptidase M35 | 313.754567 | 18.72345626 | 360.2641548 | -4.070672819 | 0 | 4.266672236 | 0 |
| Bcin09g01190 | Serine peptidase S53 | 60.57577435 | 6.099434134 | 142.3459742 | -3.318166908 | 9.19E-22 | 4.547241816 | 2.40E-36 |
| Bcin13g02960 | Serine peptidase (trypsin-like) | 46.71840456 | 1.92644551 | 121.7178562 | -4.60327289 | 1.40E-158 | 5.981239957 | 0 |
| Bcin06g01000 | spherulin 4-like cell surface protein | 6.234711032 | 4.118644939 | 6.780584399 | -0.604125286 | 0.022700526 | 0.72191628 | 0.00551109 |
| Bcin02g04800 | Zn metallopeptidase M14 | 53.21652025 | 4.5352053 | 77.67069369 | -3.559178218 | 1.42E-171 | 4.101496539 | 8.80E-264 |
| Bcin16g02770 | Zn metallopeptidase M35 Bcmpl | 460.2894226 | 92.03414849 | 601.6113514 | -2.3275568 | 0 | 2.710509315 | 0 |
| Bcin04g03510 | barwin-like endoglucanase | 51.51723449 | 41.54984213 | 66.53471141 | -0.314958194 | 9.95E-06 | 0.680585243 | 1.18E-18 |
| Bcin03g03840 | glucan 1,3-beta-glucosidase precursor | 16.37841514 | 13.19604466 | 18.43959637 | -0.316416313 | 0.002071306 | 0.483833305 | 4.94E-07 |
| Bcin02g07630 | predicted protein | 1.091548561 | 0.655940196 | 1.435057965 | -0.743306448 | 0.199186738 | 1.134902303 | 0.031781548 |
| Bcin06g00330 | Serine peptidase S8_S53 | 61.37204334 | 38.03530185 | 85.27876014 | -0.694984196 | 6.60E-25 | 1.16611604 | 3.92E-76 |
