# Supplementary material for: The GATA transcription factor BcWCL2 regulates citric acid secretion to maintain redox homeostasis and full virulence in Botrytis cinerea
Source: mBio. 2024 May 30;15(7):e00133-24. doi: 10.1128/mbio.00133-24 (PMC11253612; doi:10.1128/mbio.00133-24)
Supplement: Table S8 — FPKM values of differentially expressed genes in ROS scavenging-associated genes. [file mbio.00133-24-s0009.docx]

**Table S8: FPKM values of differentially expressed genes in ROS scavenging-associated genes.**

| **Feature ID (fungi.ensembl.org)** | **Gene name** | **WT mean FPKM 48h in planta** | **Δ*bcwcl2* mean FPKM 48h in planta** | **Δbcwcl2 +CA mean FPKM 48h in planta** | **log2FoldChange:Δ*bcwcl2* vs WT** | **padj adjusted for multiple testing with the Benjamini-Hochberg procedure** | **log2FoldChange:Δ*bcwcl2*+CA vs Δ*bcwcl2*** | **padj adjusted for multiple testing with the Benjamini-Hochberg procedure** |
| --- | --- | --- | --- | --- | --- | --- | --- | --- |
| **Peroxiredoxin genes** | | | | | | | | |
| Bcin04g00570 | Bcprx1 | 29.21215378 | 20.68063495 | 29.65500282 | -0.503247048 | 4.42E-06 | 0.521718131 | 2.00E-06 |
| Bcin12g00520 | Bcprx2 | 1.456341027 | 1.161108764 | 1.588838525 | -0.328797306 | 5.69E-01 | 0.450772662 | 4.19E-01 |
| Bcin16g00120 | Bcprx3 | 19.71078039 | 21.10060739 | 13.81956727 | 0.093799432 | 4.78E-01 | -0.608552632 | 2.0114E-05 |
| Bcin01g08520 | Bcprx4 | 106.4549169 | 89.67682233 | 122.1018594 | -0.25252197 | 0.000307074 | 0.446788479 | 2.17E-08 |
| Bcin08g01880 | Bcprx5 | 3.040151291 | 3.272863344 | 2.442911128 | 0.100865994 | 0.756278622 | -0.419949598 | 2.27E-01 |
| Bcin10g05930 | Bcprx6 | 16.32162678 | 21.37445496 | 14.98031951 | 0.383312813 | 3.31E-02 | -0.510929582 | 5.50E-03 |
| Bcin02g08370 | Bcprx7 | 41.00871661 | 46.17096539 | 40.49636814 | 0.166020932 | 0.164384682 | -0.187731212 | 7.79E-02 |
| Bcin09g03930 | Bcprx8 | 621.9663057 | 274.4053632 | 345.2147405 | -1.185634082 | 4.91E-191 | 0.332880875 | 1.06E-14 |
| Bcin10g01030 | Bcprx9 | 810.0380629 | 1019.412574 | 637.5572557 | 0.326542841 | 1.06E-21 | -0.675358398 | 4.43E-81 |
| **Superoxide Dismutase genes** | | | | | | | | |
| Bcin03g03390 | Bcsod1 | 1126.168489 | 1552.083181 | 1116.119358 | 0.457702422 | 2.40745E-55 | -0.474044439 | 2.41878E-54 |
| Bcin06g03160 | Bcsod2 | 16.15824095 | 20.69519856 | 7.993260287 | 0.352050976 | 0.014077875 | -1.3703576 | 4.29601E-13 |
| Bcin01g03830 | Bcsod3 | 73.09921164 | 94.37629043 | 77.82257095 | 0.363694138 | 8.35E-09 | -0.276672017 | 5.84E-06 |
| Bcin05g05040 | Bcsod4 | 323.8864315 | 406.0553646 | 310.7127108 | 0.32106704 | 7.78E-15 | -0.384330227 | 8.67608E-20 |
| **Catalase gene** | | | | | | | | |
| Bcin06g01180 | BccatA | 0.104428039 | 0.095831755 | 0.169072228 | -0.12408836 | 9.49E-01 | 0.818252452 | 0.606431015 |
| Bcin11g06450 | Bccat2 | 3.020037548 | 3.805068783 | 3.536934654 | 0.329428418 | 0.18375513 | -0.105270791 | 6.81E-01 |
| Bcin06g04520 | Bccat3 | 1.76111887 | 5.024738583 | 5.888687965 | 1.507504964 | 8.4782E-11 | 0.230244793 | 0.229591573 |
| Bcin05g00730 | Bccat4 | 0.162045369 | 0.065892868 | 0.285554928 | -1.295968834 | 0.421158428 | 2.107021246 | 0.138040768 |
| Bcin03g01920 | Bccat5 | 284.0877248 | 226.9186378 | 106.9425644 | -0.329213279 | 2.34E-24 | -1.083524712 | 8.12E-145 |
| Bcin05g04580 | Bccat6 | 0.500176607 | 0.232085147 | 0.916724383 | -1.114173146 | 0.150865953 | 1.985981896 | 3.34E-03 |
| Bcin09g04400 | Bccat7 | 0.402692099 | 0.16832753 | 0.449907417 | -1.259333421 | 0.292016951 | 1.416000024 | 0.221986463 |
| Bcin04g02010 | Bccat8 | 1.109441714 | 1.119436107 | 1.111221175 | 0.007027948 | 9.91E-01 | -0.005710489 | 9.93E-01 |
| **Cytochrome c peroxidase gene** | | | | | | | | |
| Bcin07g05810 | Bcccp2 | 48.89992401 | 36.06707914 | 53.33535974 | -0.443473809 | 1.72711E-05 | 0.565435106 | 2.2054E-08 |
| Bcin01g09360 | Bcccp1 | 632.8699401 | 507.2590875 | 398.3697076 | -0.324133214 | 9.37E-23 | -0.34704402 | 4.38E-30 |
| **Peroxidase genes** | | | | | | | | |
| Bcin13g05720 | Bcprd1 | 50.66061659 | 65.37277648 | 60.19233031 | 0.362399019 | 8.36511E-07 | -0.11709375 | 0.107078586 |
| Bcin05g01450 | Bcprd2 | 2.84687163 | 2.203923099 | 2.120570532 | -0.375048985 | 2.14E-01 | -0.053554087 | 0.866877928 |
| Bcin11g03930 | Bcprd3 | 3.299766242 | 4.300164411 | 2.748259448 | 0.378955923 | 0.246686889 | -0.645783079 | 0.052614099 |
| Bcin05g00590 | Bcprd4 | 0.848661593 | 1.016547152 | 1.006686067 | 0.255378157 | 6.23E-01 | -0.010656776 | 9.83E-01 |
| Bcin03g00320 | Bcprd5 | 16.08348259 | 21.25848545 | 22.82625059 | 0.398298294 | 9.64E-03 | 0.103290788 | 0.427652728 |
| Bcin02g06340 | Bcprd6 | 0.431145727 | 1.014130763 | 0.579864523 | 1.225629293 | 0.095956777 | -0.801071138 | 0.256232872 |
| Bcin10g02560 | Bcprd7 | 2.588247706 | 5.896620184 | 3.785185383 | 1.183219674 | 1.79E-07 | -0.63803195 | 3.21E-03 |
| Bcin05g02430 | Bcprd8 | 1.565979061 | 2.037420202 | 1.776024116 | 0.375641434 | 0.27530609 | -0.198371852 | 0.595856345 |
| Bcin03g07850 | Bcprd10 | 0.285782545 | 0.460415389 | 0.529702613 | 0.684531486 | 3.36E-01 | 0.202094249 | 7.35E-01 |
| Bcin13g03680 | Bcprd11 | 48.15145799 | 47.08894566 | 41.30153825 | -0.037418284 | 0.60762507 | -0.187475787 | 1.06E-02 |
